# Total synthesis of tetraacylated phosphatidylinositol hexamannoside and evaluation of its immunomodulatory activity

**DOI:** 10.1038/ncomms8239

**Published:** 2015-06-03

**Authors:** Pratap S. Patil, Ting-Jen Rachel Cheng, Medel Manuel L. Zulueta, Shih-Ting Yang, Larry S. Lico, Shang-Cheng Hung

**Affiliations:** 1 Genomics Research Center, Academia Sinica, No. 128, Section 2, Academia Road, Taipei 115, Taiwan

## Abstract

Tuberculosis, aggravated by drug-resistant strains and HIV co-infection of the causative agent *Mycobacterium tuberculosis*, is a global problem that affects millions of people. With essential immunoregulatory roles, phosphatidylinositol mannosides are among the cell-envelope components critical to the pathogenesis and survival of *M. tuberculosis* inside its host. Here we report the first synthesis of the highly complex tetraacylated phosphatidylinositol hexamannoside (Ac_2_PIM_6_), having stearic and tuberculostearic acids as lipid components. Our effort makes use of stereoelectronic and steric effects to control the regioselective and stereoselective outcomes and minimize the synthetic steps, particularly in the key desymmetrization and functionalization of *myo*-inositol. A short synthesis of tuberculostearic acid in six steps from the Roche ester is also described. Mice exposed to the synthesized Ac_2_PIM_6_ exhibit increased production of interleukin-4 and interferon-γ, and the corresponding adjuvant effect is shown by the induction of ovalbumin- and tetanus toxoid-specific antibodies.

## Abstract (web summary)

Phosphatidylinositol mannosides are cell envelope components vital for the survival of *M. tuberculosis*. Here, the authors report an elegant and convergent total synthesis of the complex glycolipid tetraacylated phosphatidylinositol hexamannoside (Ac2PIM6) and study the immunological effects in mice.

## Graphical abstract

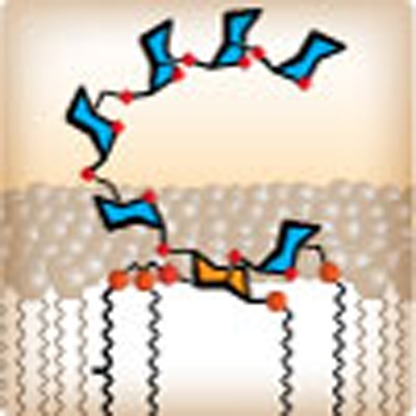

M*ycobacterium tuberculosis* is a dreaded pathogen that causes tuberculosis, one of the leading causes of death in the world. Although the disease becomes active for only 5–10% of infected individuals, 1.5 million people died of tuberculosis in 2013 alone despite progress in the global effort for diagnosis, treatment and prevention^1^. Moreover, it is estimated that one-third of the human population is latently infected with *M. tuberculosis* and is highly vulnerable if immunocompromised. The antituberculosis vaccine bacillus Calmette-Guérin, made by using an attenuated strain of *M. bovis*, only gives protection to children but is highly variable in adults^2^. Co-infection with HIV and the rising cases of multidrug and extensive drug resistance also add to the high morbidity and mortality of the disease^3^. Clearly, fresh insights on the character of the causative agent and its pathogenesis are needed to help alleviate this human condition^4^.

The thick glycolipid-containing cell envelope^5^ of *M. tuberculosis* is critical for bacterial survival and growth. It is involved in sabotaging immunoregulatory responses^6^,^7^,^8^ and it forms a protective barrier for various drugs^9^,^10^. Among the vital cell-envelope components, phosphatidylinositol mannosides (PIMs) and their hypermannosylated structural relatives (lipomannans and lipoarabinomannans) are found noncovalently anchored to the plasma membrane and the outer capsule through palmitate, stearate and tuberculostearate lipid chains^11^. PIMs, in particular, dictate the intercellular fate of mycobacteria by binding to macrophages^12^, regulate cytokines and reactive radical species and stimulate early endosomal fusion by acting as ligands to Toll-like receptors, C-type lectins and DC-SIGN^13^. PIMs can also act as CD1d antigen to activate natural killer T cells for the production of interferon-γ (ref. 14), indicating their potential as vaccine or adjuvant candidates. In addition, PIMs interact with α_5_β_1_ integrin on CD4^+^ lymphocytes, which can either promote granuloma formation and enhance host immune response or help in bacterial survival^15^.

Structurally, *myo*-inositol is the central support unit of PIMs with a diacylated glycerophospholipid moiety at O1 and α-mannosylation sites at O2 and O6 (ref. 16). Additional lipid chains may be linked at the primary hydroxyl of the 2-*O*-mannosyl unit and at the O3 position of *myo*-inositol to form triacylated PIMs (AcPIMs) and tetraacylated PIMs (Ac_2_PIMs), respectively. Higher PIMs (for example, Ac_*n*_PIM_3_−Ac_*n*_PIM_6_) are formed by elongation at the mannose residue linked at O6 of *myo*-inositol. The number of mannose residues and the degree and type of the fatty acyl groups present in the PIM molecules determine their unique role in immunoregulation^11^. As a result, elegant synthetic strategies have been developed for PIMs and their related compounds^17^,^18^,^19^,^20^,^21^,^22^,^23^,^24^,^25^,^26^,^27^,^28^,^29^,^30^,^31^,^32^,^33^,^34^. Nevertheless, the synthesis of a tetraacylated phosphatidylinositol hexamannoside (Ac_2_PIM_6_), the most complex among this class of compounds, is yet to be reported. Thus far, previous disclosures explored the synthesis of Ac_2_PIM_2_ (ref. 34), PIM_4_ (ref. 24), PIM_6_ (ref. 32) and AcPIM_6_ (ref. 25) to name a few.

We describe herein the first synthesis of Ac_2_PIM_6_, using stearic and tuberculostearic acids as the lipid components. The immunomodulatory activity of the synthesized Ac_2_PIM_6_ was also evaluated.

## Results

### Synthetic strategy

**Figure 1:**
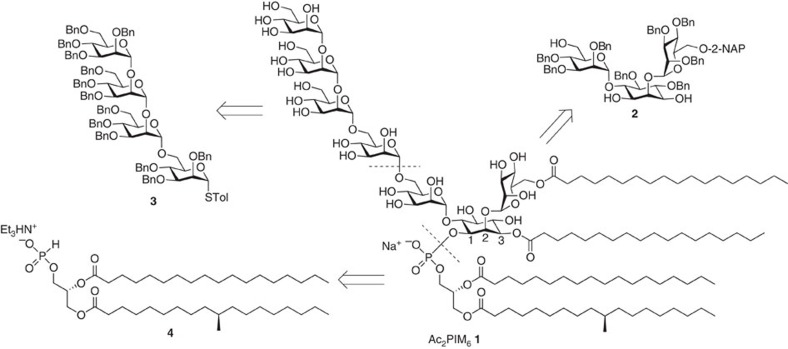
Our target tetraacylated phosphatidylinositol hexamannoside (Ac_2_PIM_6_) and the main blocks designed to represent each segment. 2-NAP, 2-naphthylmethyl; Bn, benzyl; Tol, *p*-tolyl.

Compound **1** possesses multiple components and functionalizations. To arrive at this molecule, one rational synthetic design would be to fragment this sizeable structure into separate segments, which could later be assembled in a convergent manner. For this purpose, we conceived the pseudotrisaccharide **2**, tetramannoside donor **3** and phosphonate **4** as the primary targets (Fig. 1). The readily perceptible synthetic issues include the transformation of the ordinarily meso *myo*-inositol into the unsymmetrical counterpart in **2** as well as the regioselective protection to afford the mannosyl-building blocks useful enough to deliver the necessary α1→2 and α1→6 linkages and the acylation of one mannosyl unit. Accordingly, along with benzyl groups for the global protection of hydroxyls that would be free in the desired product, we selected two additional orthogonal protecting groups for the primary positions of the mannosyl residues in intermediates leading to compound **2**. The *tert*-butyldiphenylsilyl (TBDPS) group should allow, on deprotection, the subsequent coupling with the tetramannoside **3**, whereas the 2-naphthylmethyl (2-NAP) group protects the position that would later be acylated. Being a core constituent of inositol phosphates and other phosphatidyl lipoglycans, various methods have been published for the *myo*-inositol resolution and desymmetrization^35^,^36^. However, the suitably protected chiral *myo*-inositol derivative required for PIMs and glycosyl phosphatidylinositide synthesis were mainly achieved through multistep synthesis from D-glucose via the Ferrier reaction^37^. Recently, we have shown that a mannosyl donor can directly act as chiral auxiliary to differentiate the enantiotopic hydroxyls of meso *myo*-inositol derivatives^30^,^31^. This direct approach bypasses many transformation steps in affording an appropriately mannosylated and desymmetrized *myo*-inositol. We intend to apply this capacity towards diol **2** while also relying on the steric hindrance created by the installed mannosyl residues to strategically position the 4,5-di-*O*-benzyl moieties on the *myo*-inositol unit and sufficiently favour regioselective acylation at the free 3-OH. An extra temporary protecting group and participating moiety is also needed to permit the construction of the tetramannoside **3**, in which case we chose a 2-*O*-benzoyl group. We planned to assemble **3** through linear glycosylation from the reducing end to the nonreducing end using a single elongation unit. For the phosphonate **4**, access to the rare tuberculostearic acid is the main concern, and this fatty acid should be synthesized to complete the desired phospholipid moiety.

### Mannosyl-building blocks

**Figure 2:**
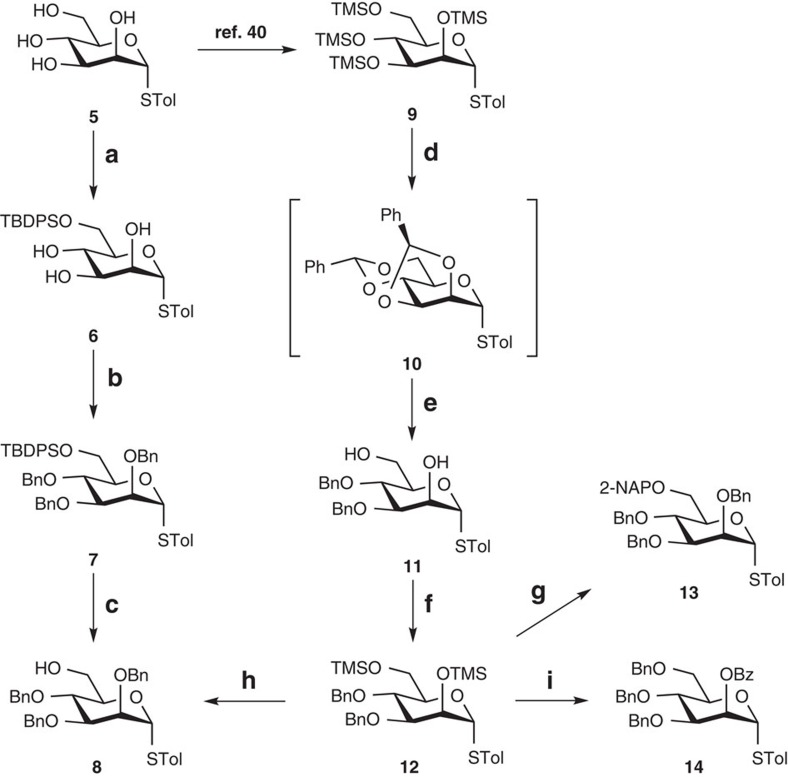
Preparations of the mannosyl-building blocks. Reagents and conditions: (**a**) *tert*-butyldiphenylchlorosilane, Et_3_N, DMAP, 93%; (**b**) NaH, BnBr, DMF, 94%; (**c**) PTSA, MeOH, CH_2_Cl_2_, 92%; (**d**) benzaldehyde (2.1 equivalents), TMSOTf, MeCN, 0 °C, 30 min; (**e**) BH_3_·THF, Cu(OTf)_2_, CH_2_Cl_2_, 87% (one pot from **9**); (**f**) trimethylchlorosilane, Et_3_N, quantitative; (**g**) 2-naphthaldehyde, Et_3_SiH, TMSOTf, CH_2_Cl_2_, −78 to −40 °C, 2 h, then, NaH, BnBr, DMF, 81% (one pot); (**h**) 2-naphthaldehyde, Et_3_SiH, TMSOTf, CH_2_Cl_2_, −78 to −40 °C, 2 h, then, NaH, BnBr, DMF, then, DDQ, H_2_O, 73% (one pot); (**i**) benzaldehyde, Et_3_SiH, TMSOTf, −78 °C, 1.5 h, then, BF_3_·Et_2_O, MeCN, −78 to −20 °C, 30 min, then, Bz_2_O, Et_3_N, 93% (one pot). Bz, benzoyl; Bz_2_O, benzoic anhydride; Cu(OTf)_2_, copper(II) trifluoromethanesulfonate; DDQ, 2,3-dichloro-5,6-dicyano-1,4-benzoquinone; DMAP, 4-(*N*,*N*-dimethylamino)pyridine; DMF, *N*,*N*-dimethylformamide; Ph, phenyl; PTSA, *p*-toluenesulfonic acid; TBDPS, *tert*-butyldiphenylsilyl; THF, tetrahydrofuran; TMS, trimethylsilyl, TMSOTf, trimethylsilyl trifluoromethanesulfonate.

Considering the stability of the thiotolyl leaving group on various functional group interconversions, we selected the thiomannoside **5** (ref. 38) as a starting point in our transformations towards several mannosyl building blocks (Fig. 2). In general, the building blocks needed for the assembly of our target structure required differentiation at either O6 or O2. With a bulky functionality such as the TBDPS group, the protection sequence aimed regioselectively at the primary O6 position seems clear-cut. Thus, the 6-*O*-silylation of **5** using *tert*-butyldiphenylchlorosilane, triethylamine and 4-(*N*,*N*-dimethylamino)pyridine gave compound **6** in excellent yield. Subsequent benzylation under Williamson condition supplied the necessary thioglycoside **7**. The 6-alcohol **8**, intended as the starting acceptor in generating the tetramannoside **3**, was also readily acquired from **7** by acidic desilylation.

In contrast to the route above, traditional approaches concerning the effective acquisitions of the 6-*O*-naphthylmethylated thiomannoside **13** and the 2-benzoate **14** do not appear to be straightforward. The complexity arises from the desire to carry out the fully regioselective installations of the vital ether groups. Apparently, the regioselective one-pot protection strategy that we introduced^39^,^40^ and further expanded to other sugars^41^,^42^,^43^,^44^ could simplify such preparations. Our recent work on the stereoselective dioxolane-type benzylidene formation on thiomannosides^43^ should provide a convenient gateway to the 2,6-diol **11**, a potential common intermediate towards compounds **13** and **14**. It was envisioned that, with benzyl groups permanently protecting O3 and O4, the primary 6-hydroxyl could be easily differentiated from the secondary and axial 2-hydroxyl. We were also keen to check whether stereoselective dibenzylidenation and simultaneous regioselective ring opening could be achieved in one pot.

Starting from the tetrakis-trimethylsilyl ether **9** acquired in one step from **5** (ref. 43), treatment with 2.1 equivalents of benzaldehyde along with catalytic trimethylsilyl trifluoromethanesulfonate (TMSOTf) in acetonitrile at 0 °C exclusively delivered the *exo*-product **10** as evidenced by NMR spectroscopy and X-ray crystallography (Supplementary Fig. 1, Supplementary Data 1). This fully stereoselective transformation is beneficial because unlike the regioselectivity in the 4,6-*O*-benzylidene ring opening, which is determined by the choice of reducing agent^45^, the opening of the dioxolane-type 2,3-*O*-benzylidine moiety is guided by the orientation of the phenyl group (that is, *exo*-isomers generally open at the axial position). Delightfully, the subsequent exposure of **10** to BH_3_·tetrahydrofuran in the same vessel provided the diol **11** in a two-stage one-pot yield of 87%. Anticipating a smooth regioselective reductive etherification at O6 (ref. 46), we subjected the diol **11** to trimethylsilylation. Consequently, treatment of the so-formed **12** with 2-naphthaldehyde, triethylsilane and TMSOTf in subzero temperature supplied the intermediate that was benzylated in one pot using the typical etherification method to afford compound **13**. An X-ray single crystal analysis fully supported the desired structure (Supplementary Fig. 1, Supplementary Data 2). Continuing further, addition of 2,3-dichloro-5,6-dicyano-1,4-benzoquinone to the *in situ*-generated **13** successfully delivered the same acceptor **8**, thus providing an alternative pathway for its acquisition. Similarly, the corresponding reductive 6-O-benzylation of **12** was carried out, followed by desilylation at O2 with tetrabutylammonium fluoride (TBAF) and basic benzoylation in the same flask. Unfortunately, the yield for compound **14** was less than satisfactory even when benzoic acid and TBAF were used together^41^ to perform the desilylation, probably due to the interference of TBAF to the benzoylation stage. Desilylation with BF_3_·Et_2_O apparently solved this issue and furnished **14** in an excellent 93% yield from **12**.

### Synthesis of the pseudotrisaccharide 2

**Figure 3:**
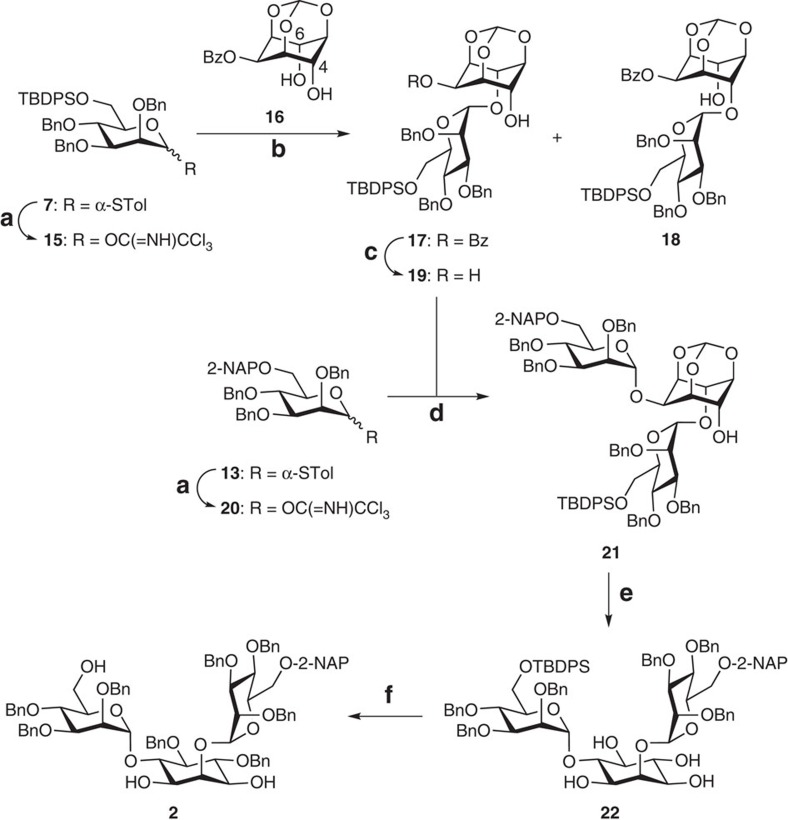
Preparation of the pseudotrisaccharide 2. Reagents and conditions: (**a**) (1) NBS, acetone, H_2_O; (2) K_2_CO_3_, CCl_3_CN, **15**: 94% (two steps), **20**: 90% (two steps); (**b**) silver trifluoromethanesulfonate, 1,4-dioxane, CH_2_Cl_2_, **17**: 68%, **18**: 20%; (**c**) NaOMe, MeOH, CH_2_Cl_2_, quantitative; (**d**) BF_3_·Et_2_O, CH_2_Cl_2_, −60 to −20 °C, 72%; (**e**) (1) PTSA, MeOH, CH_2_Cl_2_, 84%; (2) *tert*-butyldiphenylchlorosilane, Et_3_N, DMAP, 82%; (**f**) (1) trimethylchlorosilane, Et_3_N, quantitative; (2) benzaldehyde (3 equivalent), Et_3_SiH, TMSOTf, CH_2_Cl_2_, −40 °C, then, tetrabutylammonium fluoride, 72%. NBS, *N*-bromosuccinimide.

For the desymmetrization of *myo*-inositol, we evaluated the coupling of the mannosyl donor **7** with the meso diol **16** (Fig. 3), which can be easily prepared in one step^30^ from the commercially available Kishi's triol. The asymmetric nature of the mannosyl donor itself should provide certain preferences between the axial hydroxyls of **16** as we have demonstrated previously^30^ but without the TBDPS group on the sugar. Because of the wider opening available for a nucleophilic attack on the half-chair mannosyl oxocarbenium ion intermediate by O6 as compared with O4 (Supplementary Fig. 2), it is expected that the required 6*-O*-mannosylation would be more favoured. Our attempts at coupling of **7** and **16** using *N*-iodosuccinimide and TMSOTf in CH_2_Cl_2_ and 1,4-dioxane to improve the solubility of diol **16**, unfortunately, led only to donor hydrolysis and full recovery of the acceptor. We suspected that the axial hydroxyls are too unreactive for mannosyl thioglycoside to foster productive couplings. With strong activators avoided to maintain the acid-sensitive orthoformate group, focus was shifted to the imidate versions of the donor. After some optimization (see the Supplementary Table 1), silver trifluoromethanesulfonate promoted the glycosylation step at room temperature, supplying the desired pseudodisaccharide **17** at 68% yield, along with its regioisomer **18** (20%). Here and in the succeeding glycosylations, we verified the α-orientations of the mannosidic bonds through the coupling constants of the anomeric carbons and protons (∼170 Hz, see Supplementary Methods)^47^,^48^. Distinguishing the structures of **17** and **18** with confidence is not possible with NMR analysis alone. We, therefore, resorted to exchange the primary TBDPS with benzyl group (Supplementary Fig. 3) and compare agreement with the NMR spectra from previously published data^30^.

For the preparation of the key intermediate **21**, the pseudodisaccharide **17** was subjected to Zemplén deacylation to generate the diol **19**. Regioselective mannosylation at the equatorial hydroxy group should be more likely because of steric reasons. A thorough evaluation of the subsequent coupling also made us consider the application of the imidate **20** over the thioglycoside **13**. Under BF_3_·Et_2_O promotion, compound **21** was, therefore, acquired in 72% yield with complete regioselectivity and stereoselectivity. The orthoformate group was cleaved using *p*-toluenesulfonic acid, which also removed the TBDPS group. The tetraol **22** was obtained after re-installation of the silyl group. With **22** in hand, regioselective benzylation at O4 and O5 of the inositol unit is the next challenge. Williamson condition and acidic benzylation using benzyl imidate are not sufficiently selective, whereas the reductive benzylation of the trimethylsilylated substrate showed greater promise. True enough, excellent selectivity was achieved by using 3 equivalents of benzaldehyde, furnishing, after further full desilylation with TBAF, the desired compound **2** in 72% yield from **22**.

It should be stated that other less successful means in acquiring the pseudotrisaccharide backbone have been studied. Our effort at condensation of the imidate donor **20** with the diol **16** led to mixtures of inseparable regioisomers and stereoisomers, a demonstration of the known potential of the bulky 6-*O*-TBDPS group at enhancing α-selectivity^49^. A participating moiety at O2 of the mannosyl donor was ruled out to avoid complications that may be encountered in later reactions. Sequential dimannosylation of Kishi's triol also seemed feasible under our synthetic design, with glycosylation at the more reactive O2 using donor **20** followed by asymmetric 6-*O*-mannosylation with donor **15**. Unfortunately, poor yields for both couplings were observed. Another procedure we have tried included the 2-*O*-mannosylation of **19** with a donor already carrying the fatty acyl functionality at the primary position. While the glycosylation step worked as intended, the acyl moiety was also removed along with the TBDPS group on acid treatment intended to cleave the orthoformate function.

### Synthesis of tuberculostearic acid and the *H*-phosphonate 4

**Figure 4:**
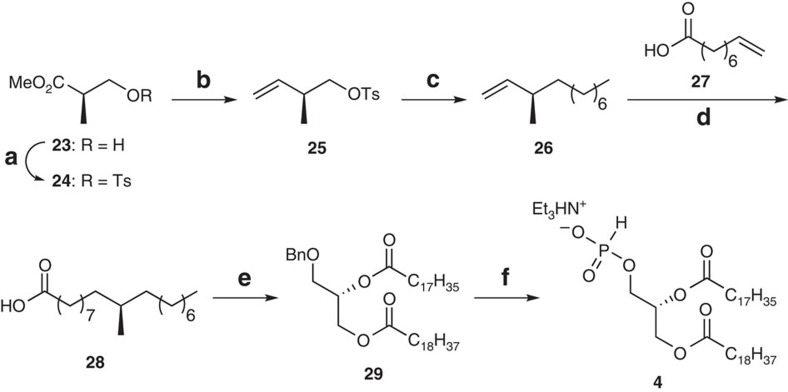
Preparation of tuberculostearic acid (28) and the *H*-phosphonate 4. Reagents and conditions: (**a**) TsCl, Et_3_N, DMAP, 94%; (**b**) (1) diisobutylaluminium hydride, −78 °C; (2) Ph_3_P=CH_2_, 72% (two steps); (**c**) C_7_H_15_MgBr, Li_2_CuCl_4_, −78 to 0 °C, 92%; (**d**) (1) Grubbs second-generation catalyst, CH_2_Cl_2_, reflux; (2) H_2_, Pd/C, 75% (two steps); (**e**) (1) 3-*O*-benzyl-*sn*-glycerol, DCC, DMAP, 75%; (2) stearic acid, DCC, DMAP, 88%; (**f**) (1) H_2_, Pd/C, 92%; (2) PCl_3_, imidazole, Et_3_N, −10 °C, 69%. DCC, dicyclohexylcarbodiimide; Ts, Tosyl.

Tuberculostearic acid was first isolated from *M. tuberculosis* in 1927 (ref. 50) and several methods for its synthesis have been reported^25^,^26^,^51^,^52^,^53^. Nevertheless, an updated, shorter and more effective method for accessing this important fatty acid is still desirable. We decided to acquire the chiral carbon of tuberculostearic acid from the commercially available Roche ester (**23**). Tosylation of **23** to afford compound **24**, followed by reduction with diisobutylaluminium hydride and methylene insertion by Wittig reaction furnished the olefin **25** (ref. 54; Fig. 4). The first long-chain elongation of **25** towards compound **26** was achieved by Grignard reaction under catalytic Li_2_CuCl_2_. Grubbs metathesis of olefin **26** with the olefinic acid **27** provided the *E*/*Z* olefin mixture, which was exposed to palladium-catalysed hydrogenation to finally secure tuberculostearic acid (**28**). Accomplished in just six steps, this acquisition is the shortest synthetic preparation reported, thus far, for this compound.

Elaborations of the commercially available 3-*O*-benzyl-*sn*-glycerol were performed next. Under dicyclohexylcarbodiimide and 4-(*N*,*N*-dimethylamino)pyridine, the fatty acid **28** was first condensed with the primary hydroxyl followed by stearic acid esterification at the secondary position in good yields. Cleavage of the benzyl group was achieved through hydrogenolysis and the generated alcohol was phosphorylated using PCl_3_, imidazole and Et_3_N to afford the *H*-phosphonate **2**.

### Ac_2_PIM_6_ assembly and final transformations

**Figure 5:**
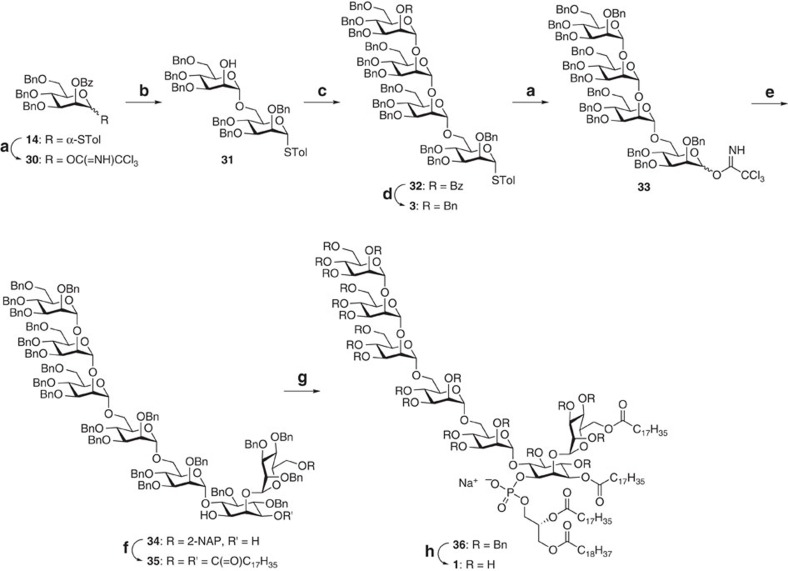
Synthesis of compound 1. Reagents and conditions: (**a**) (1) NBS, acetone, H_2_O; (2) CCl_3_CN, 1,8-diazabicyclo[5.4.0]undec-7-ene, **30**: 96% (two steps), **33**: 86% (two steps); (**b**) **8**, TMSOTf, CH_2_Cl_2_, −78 °C, then, NaOMe, MeOH, 87% (one pot); (**c**) (1) **30**, TfOH, CH_2_Cl_2_, −60 to −40 °C, then, NaOMe, MeOH, 70% (one pot); (2) **30**, TfOH, CH_2_Cl_2_, −60 to −40 °C, 74%; (**d**) BnBr, NaH, 98%; (**e**) **2**, TMSOTf, Et_2_O, −40 °C, 52% (89% yield based on the recovered **2**); (**f**) (1) DDQ, CH_2_Cl_2_, H_2_O, 71%; (2) stearic acid, DCC, DMAP, 86%; (**g**) (1) **4**, pivaloyl chloride, pyridine; (2) I_2_, pyridine, H_2_O; (3) DOWEX 50WX8 Na^+^ form, 77% from **35**; (**h**) H_2_, Pd/C, 88%. TfOH, trifluoromethanesulfonic acid.

Our planned sugar assembly towards the tetramannoside **3** hinges on the chemoselective activation of a trichloroacetimidate donor in the presence of a thioglycoside acceptor^55^ (Fig. 5). Accordingly, the elongation unit is formed by converting compound **14** to **30** under the usual procedures. Glycosylation of the thioglycoside **8** with **30** followed by debenzoylation in the same flask supplied the disaccharide acceptor **31** in 87% yield. Two more elongation cycles easily formed the tetramannoside **32**. Knowing that the benzoate group is base-sensitive, the benzoyl-to-benzyl exchange was achieved in one step by NaH and benzyl bromide treatment, smoothly offering the target compound **3** in 98% yield.

With all segment backbones available, we moved forward in putting all these pieces together. The glycosylation of the pseudotrisaccharide acceptor **2** by the thioglycoside **3**, however, produced only a meager 10% yield for compound **34** despite our best efforts, prompting us to use the more reactive imidate counterpart **33** instead (see Supplementary Table 2). With Et_2_O as solvent and TMSOTf as activator, we eventually obtained the desired **34** in 52% yield (89%, if the recovered acceptor is considered). The 2-NAP ether was then cleaved, which paved the way for the concurrent installation of two stearate esters at the mannosyl and the inositol units, leading to the alcohol **35**. This reaction exhibited no regioselectivity issues, with O1 of the inositol unit spared because it experiences the highest steric hindrance among the three free hydroxyls. The attachment of the *H*-phosphonate **4** and the pseudoheptasaccharide **35** was carried out by using pivaloyl chloride, followed by iodine-mediated *in situ* oxidation and cation exchange, delivering the derivative **36**. Global hydrogenolysis of the benzyl ethers provided the Ac_2_PIM_6_ construct **1** in 82% yield.

### Evaluation of immunomodulatory activity

**Figure 6:**
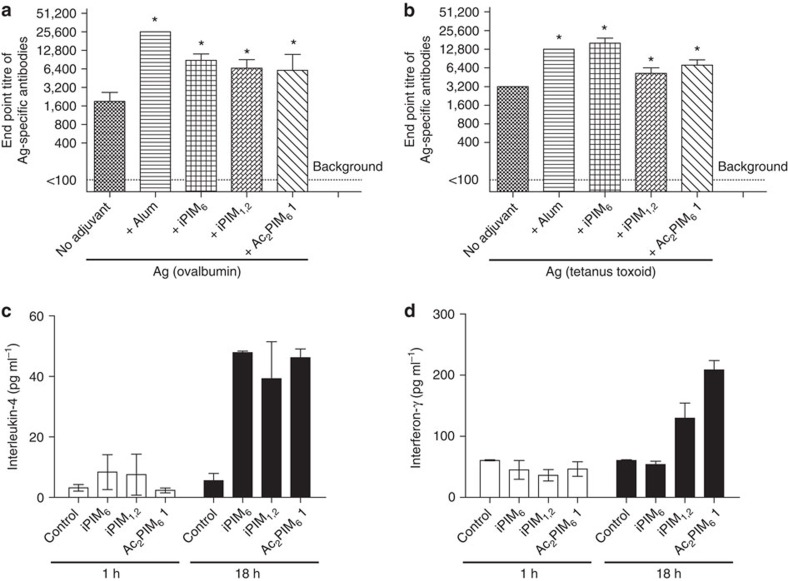
Immunological evaluation in BALB/c mice. (**a**,**b**) Induction of antigen (Ag)-specific antibodies in mice immunized with ovalbumin or tetanus toxoid adjuvanted with alum or various PIMs; (**c**,**d**) secreted cytokines (interleukin-4 and interferon-γ) in mice 1 and 18 h after injection with various PIMs (control represents injection only with PBS). Both end point antibody titres and the cytokine levels were measured by using enzyme-linked immunosorbent assay. The results displayed represent the mean+s.d.'s (*n*=5). Data were analysed by using one-way analysis of variance, and differences were considered significant at *P<0.05. iPIM_6_ and iPIM_1,2_, isolated PIM_6_ and mixture of PIM_1_ and PIM_2_, respectively, from *M. tuberculosis* strain H37Rv.

The adjuvant effects of compound **1** were examined through co-administration with ovalbumin (Fig. 6a) or tetanus toxoid (Fig. 6b) antigen in BALB/c mice. PIMs isolated from *M. tuberculosis* strain H37Rv (iPIM_1,2_ and iPIM_6_) and alum were also investigated in parallel for comparison. It was observed that compound **1** induced an approximately two to fourfold increase in the level of antigen-specific antibodies. The adjuvant activity of **1** is similar to the bacteria-derived PIMs and slightly lower than alum.

Furthermore, we evaluated the cytokine-producing activity of compound **1** as well as iPIM_1,2_ and iPIM_6_ (Fig. 6c,d). The level of interleukin-4 and interferon-γ was not detectable in mouse sera at 1 h after injection of Ac_2_PIM **1** and the bacteria-derived PIMs. At 18 h after injection, the cytokine levels increased. Lipid and glycolipid molecules derived from *M. tuberculosis* are presented to T cells by CD1 antigen-presenting molecules, specifically CD1d^14^,^56^. Compared with the well-known CD1d-targeting α-galactosylceramide, which can activate the invariant natural killer T cells and induce high levels of interleukin-4 and interferon-γ within 24 h (ref. 57), Ac_2_PIM_6_ **1** appeared to have moderate effects.

## Discussion

We have successfully developed a convenient route to synthesize an Ac_2_PIM_6_ construct in the form of compound **1** containing tuberculostearic acid and stearic acid as the fatty acid components. This is the first time that an Ac_2_PIM_6_ molecule was synthesized. Further, a novel and short synthetic route towards tuberculostearic acid was developed, with only six synthetic steps from the commercially available Roche ester and four purification stages. Our synthetic approach benefitted from the use of shared mannoside-building blocks, the carefully chosen orthogonal protecting groups and the features of the regioselective one-pot transformations from trimethylsilylated starting materials previously developed by us. The trichloroacetimidate donor types^58^ are vital factors in achieving the successful assembly processes. Regioselectivity and stereoselectivity were achieved through the aid of steric and stereoelectronic effects. Steric effects were also exploited in the direct desymmetrization of *myo*-inositol by mannosyl donors and in minimizing the number of protecting groups used in the synthesis. With practical access and functional group flexibility, the key intermediates such as the pseudotrisaccharide **2** possess good potential in supplying PIMs of different mannosylation and lipidation patterns as well. Our synthesized Ac_2_PIM_6_ has comparable adjuvant activity with the natural PIMs against ovalbumin and tetanus toxoid antigens and induced the production of interleukin-4 and interferon-γ, thus, validating the immunological qualities of PIM molecules and its value in vaccine research.

## Methods

### Chemical synthesis

The complete experimental details and compound characterization data can be found in the Supplementary Methods. For the NMR spectra of the compounds in this article, see Supplementary Figs 4–120. The mass spectrum of the synthesized Ac_2_PIM_6_ **1** is shown in Supplementary Fig. 121.

### Materials for immunological evaluation

All BALB/c mice were housed at the animal facility in the Institute of Cell Biology, Academia Sinica, Taiwan in accordance with the Institutional Animal Care Committee guidelines. Purified iPIM_1,2_ (NR-14846) and iPIM_6_ (NR-14847) were obtained through BEI Resources, National Institute of Allergy and Infectious Diseases, National Institutes of Health (USA). Ovalbumin and tetanus toxoid were purchased from InvivoGen (San Diego, CA, USA) and Adimmune Inc. (Taichung, Taiwan), respectively.

### Evaluation of adjuvant activity

Five- to six-week-old female BALB/c mice were immunized with ovalbumin (100 μg) or tetanus toxoid (2 μg) adjuvanted with 10 μg of PIM compounds (Ac_2_PIM_6_ **1**, iPIM_1,2_ or iPIM_6_) or alum in PBS for three times at 2-week intervals by intramuscular injection. Two weeks after the third immunization, the immunized mice were bled for antigen-specific antibody analysis.

Ovalbumin- and tetanus toxoid-specific antibodies in heat-inactivated serum were monitored with direct enzyme-linked immunosorbent assay (ELISA). The ovalbumin- or tetanus toxoid-coated plates were incubated with mouse serum in twofold serial dilutions for 1 h. Antigen-specific IgG was monitored by using horseradish peroxidase-conjugated anti-mouse antibodies and 3,3*′*,5,5*′*-tetramethylbenzidine substrate (Thermo Scientific Inc). After colour development, absorbance at 450 nm was recorded by using a plate reader (SpectraMax M5, Molecular Device). The end point antibody titre was defined as the highest dilution of serum to produce an absorbance 2.5 times higher than the optical absorbance produced by the pre-immune serum. The background end point antibody titre was assigned as <1:100.

### Evaluation of cytokine-producing activity

Five- to six-week-old female BALB/c mice were intramuscularly injected with 10 μg of the PIM compounds (Ac_2_PIM_6_ **1**, iPIM_1,2_ or iPIM_6_) in PBS and bled at 1 or 18 h after injection (five mice per group). The cytokines in the sera were measured with sandwich ELISA using paired anti-interleukin-4 and anti-interferon-γ monoclonal antibodies (R&D Systems).

### Statistical analysis

The response of each mouse was counted as an individual data point for statistical analysis. Data obtained from animal studies were analysed by using one-way analysis of variance from Graphpad and differences were considered significant at *P*<0.05.

## Additional information

**Accession codes:** The X-ray crystallographic coordinates for compounds **10** and **13** in this study have been deposited at the Cambridge Crystallographic Data Centre (CCDC), under deposition numbers CCDC 1040371 and CCDC 1040372, respectively. These data can be obtained free of charge from the CCDC via www.ccdc.cam.ac.uk/data_request/cif.

**How to cite this article:** Patil, P. S. *et al*. Total synthesis of tetraacylated phosphatidylinositol hexamannoside and evaluation of its immunomodulatory activity. *Nat. Commun*. 6:7239 doi: 10.1038/ncomms8239 (2015).

## Supplementary Material

Supplementary Information Supplementary Figures 1-121, Supplementary Tables 1-2, Supplementary Methods and Supplementary References

Supplementary Data 1 Crystallographic information file for compound 10

Supplementary Data 2 Crystallographic information file for compound 13
