# Supplementary material for: Total synthesis of tetraacylated phosphatidylinositol hexamannoside and evaluation of its immunomodulatory activity
Source: Nat Commun. 2015 Jun 3;6:7239. doi: 10.1038/ncomms8239 (PMC4468851; doi:10.1038/ncomms8239)
Supplement: Supplementary Information — Supplementary Figures 1-121, Supplementary Tables 1-2, Supplementary Methods and Supplementary References [file ncomms8239-s1.pdf]

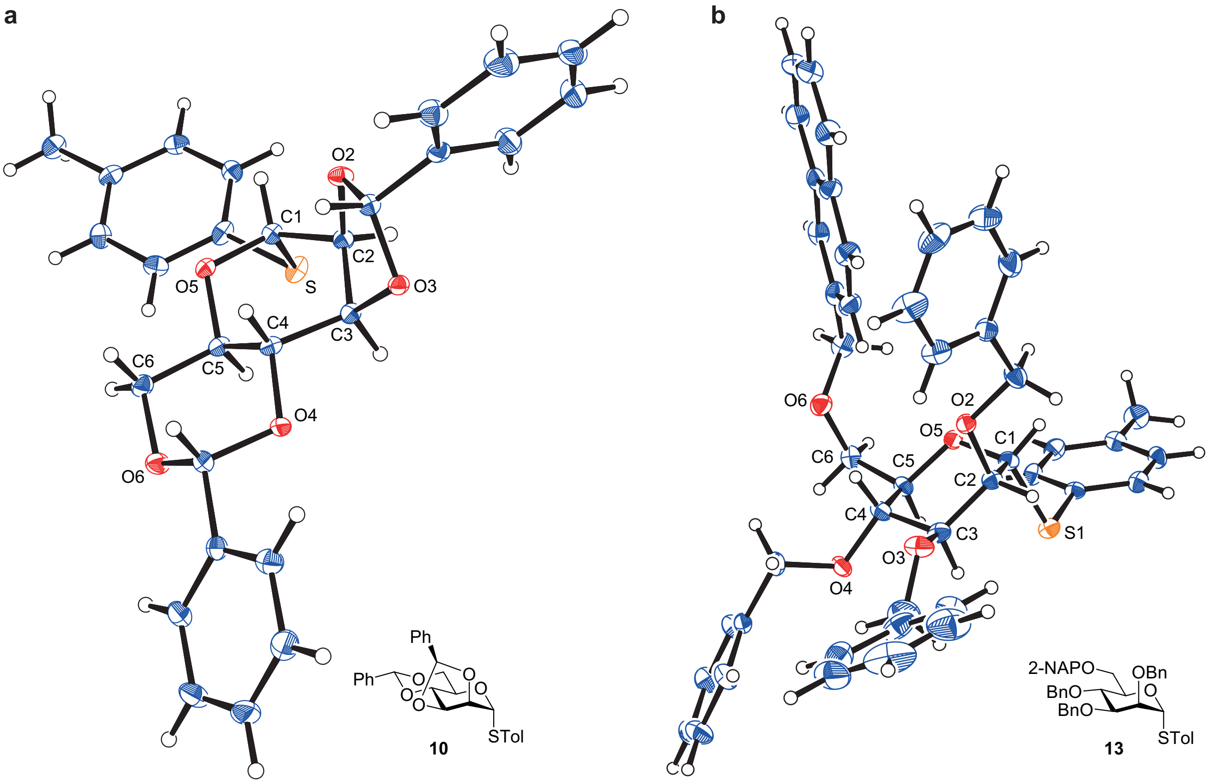

Supplementary Figure 1. X-ray crystal structures of thiomannosides. a, Compound **10**; **b**, compound **13**.

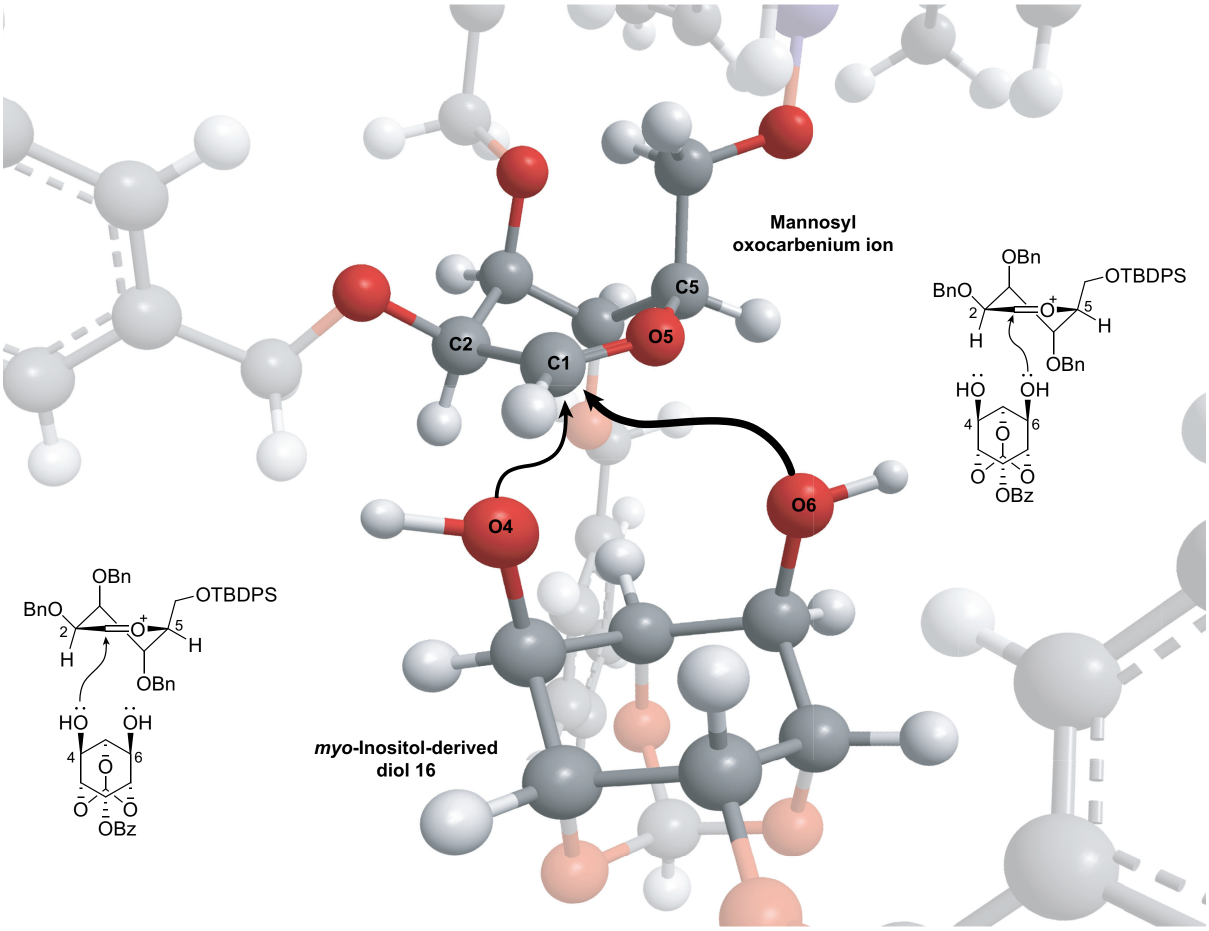

Supplementary Figure 2. Regioselective aspects in the glycosylation of the mannosyl donor 15 and the diol 16. The oxocarbenium ion adopting the low energy $^{3}H_{4}$ conformation defined by the MM2 field displays axial/pseudoaxial C3, C4 and C5 substituents and a pseudoequatorial substituent at C2[1]. The approach of the diol **16** from the back portion of the oxocarbenium ion is blocked by C4 and its benzyloxy substituent, whereas the β-face is quite crowded. While both O4 and O6 of diol **16** have opportunities in attacking the α-face of the oxocarbenium ion from the front portion, O6 has a much wider free area of approach than O4. The O4 attack may be hindered by the proton attached to the neighboring C2 and may place O6 in an unfavoured position near the proton attached to C5. It is worth mentioning that as a result of the pseudoaxial/pseudoequatorial orientations of the substituents at C2 and C5 of the oxocarbenium ion, their corresponding protons are situated at nearly similar locations with respect to C1 and O5 if $^{4}H_{3}$—another likely conformation—is adopted by the oxocarbenium ion.

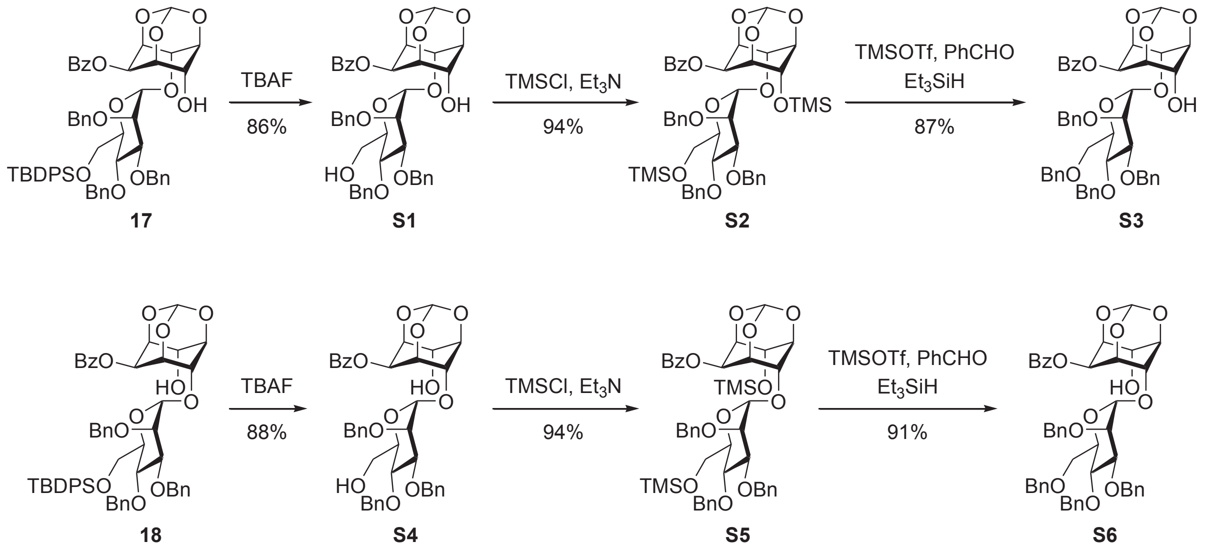

Supplementary Figure 3. Derivatisation of the pseudodisaccharides 17 and 18 for to enable structure determination by comparison with literature data[2]. TMSCl, trimethylchlorosilane.

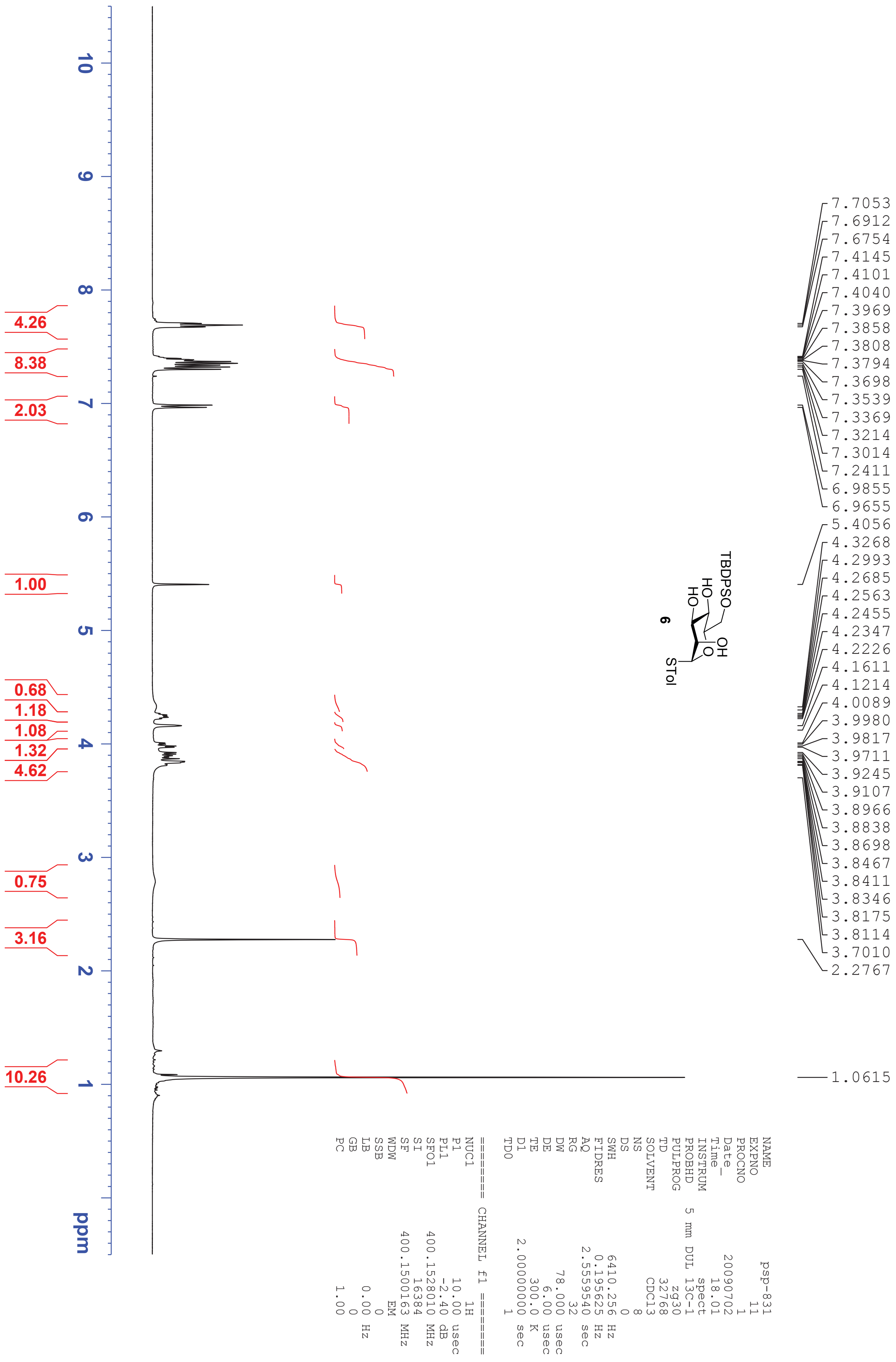

Supplementary Figure 4. ^{1}H NMR spectrum of compound 6.

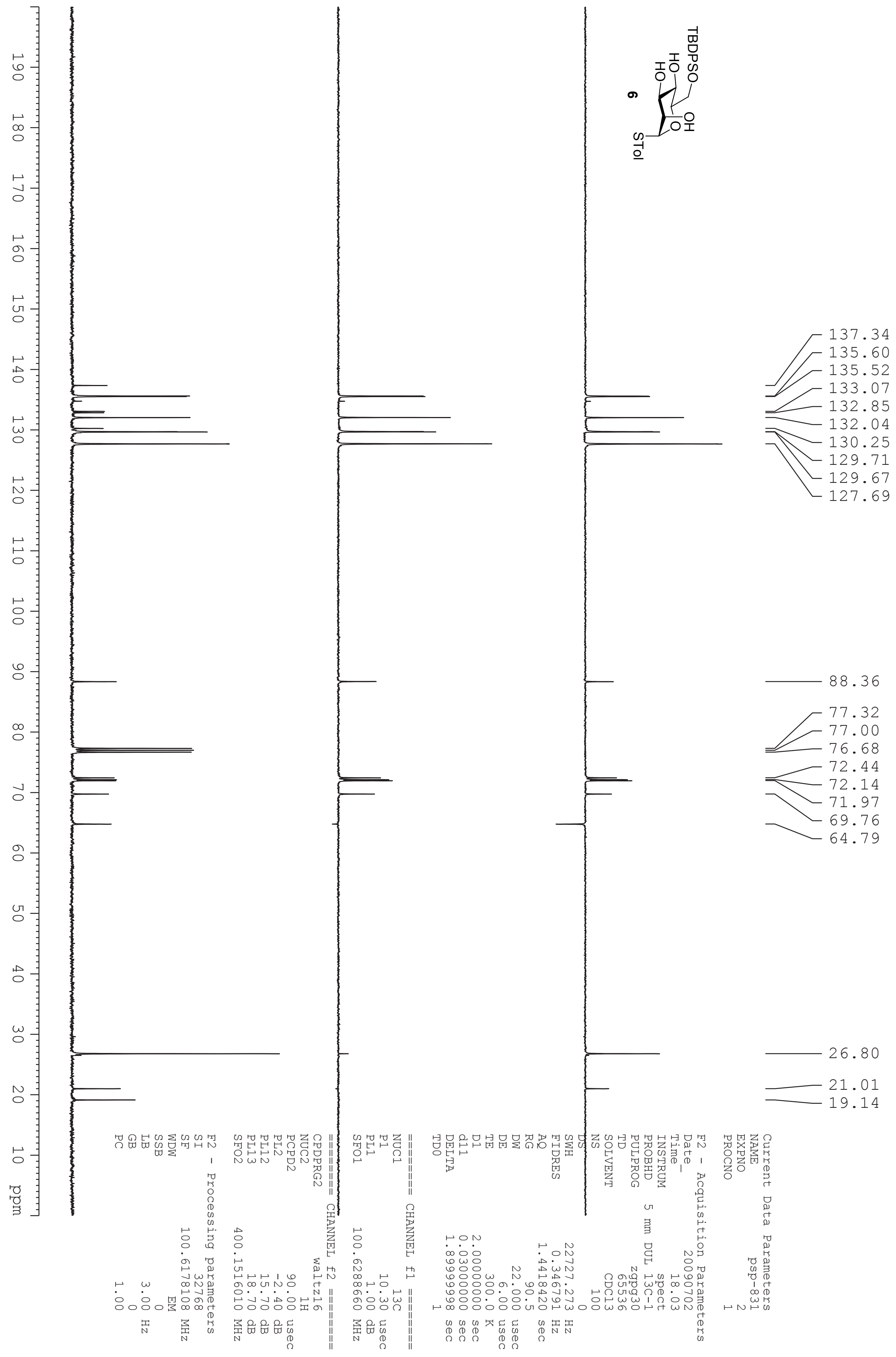

Supplementary Figure 5. ^{13}C and DEPT NMR spectra of compound 6.

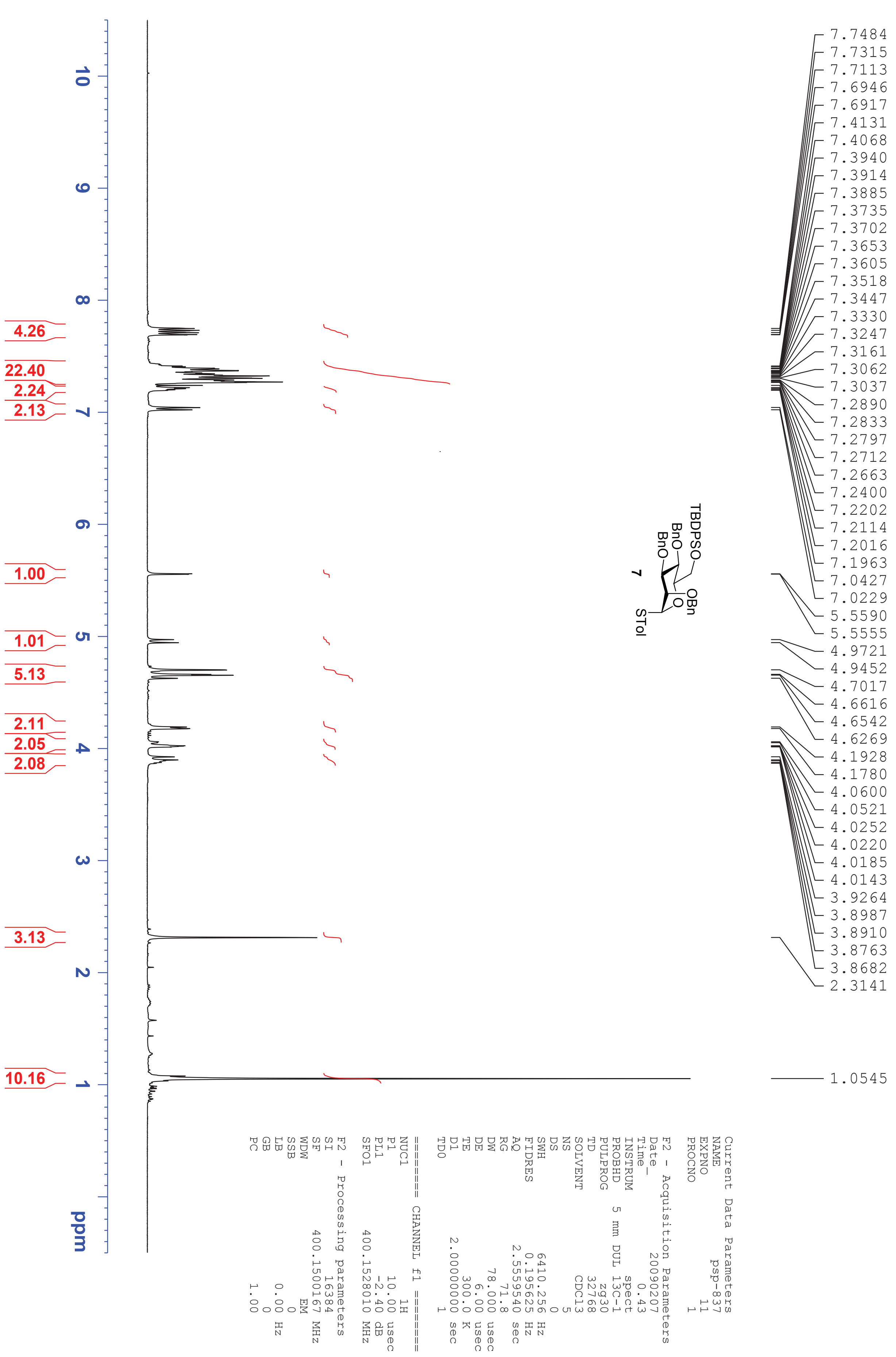

Supplementary Figure 6. ^{1}H NMR spectrum of compound 7.

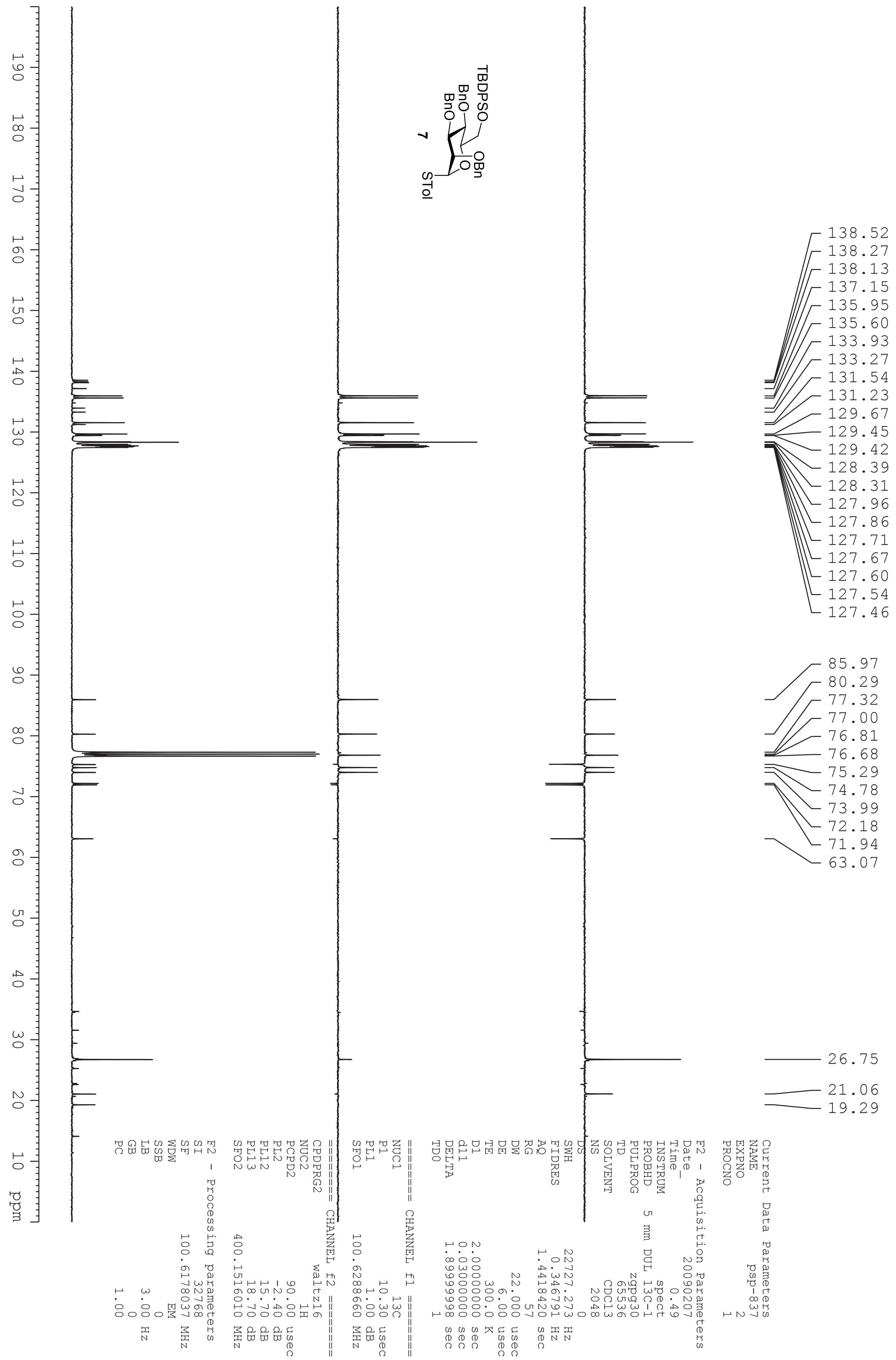

Supplementary Figure 7. ^{13}C and DEPT NMR spectra of compound 7.

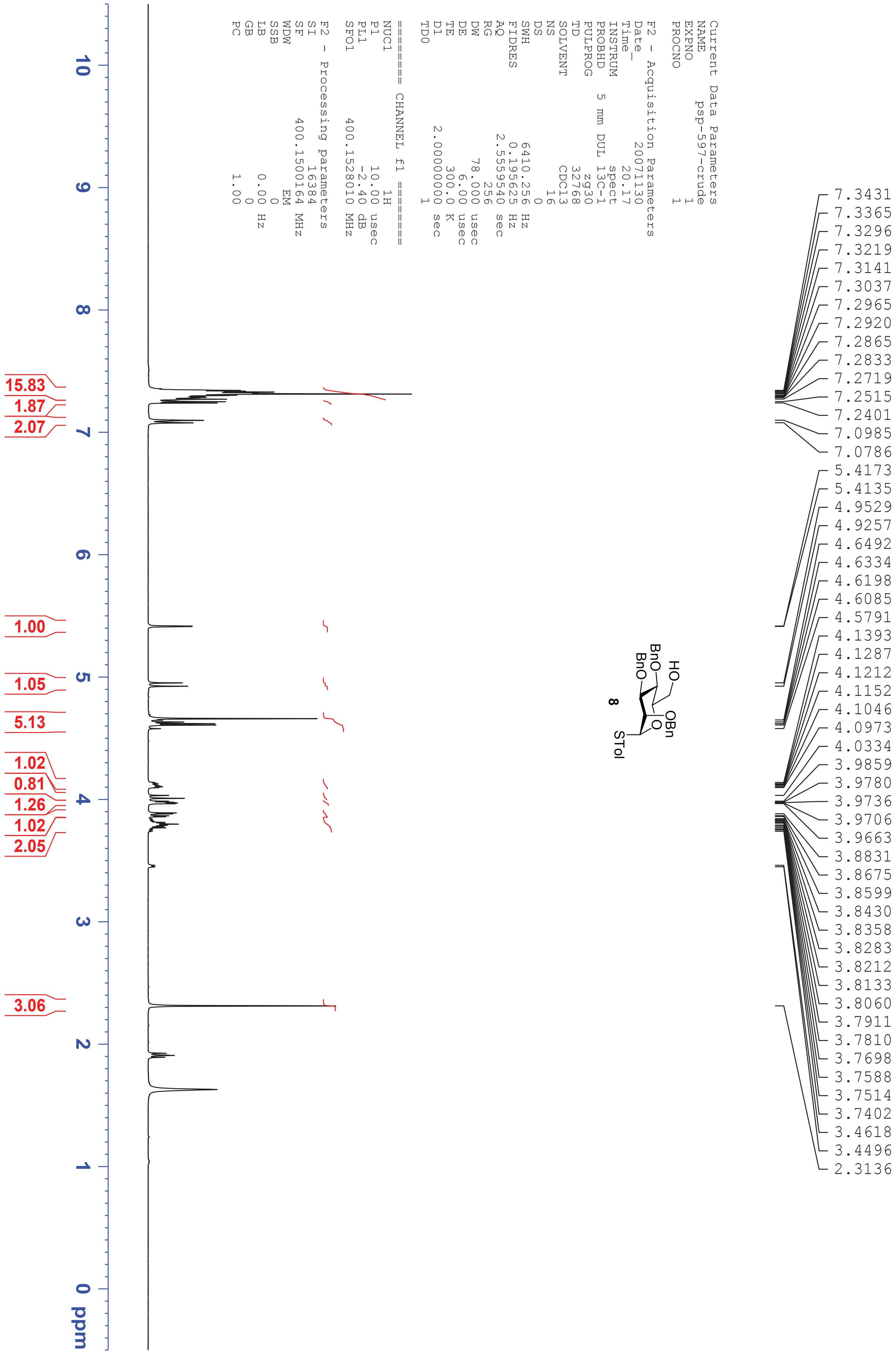

Supplementary Figure 8. ^{1}H NMR spectrum of compound 8.

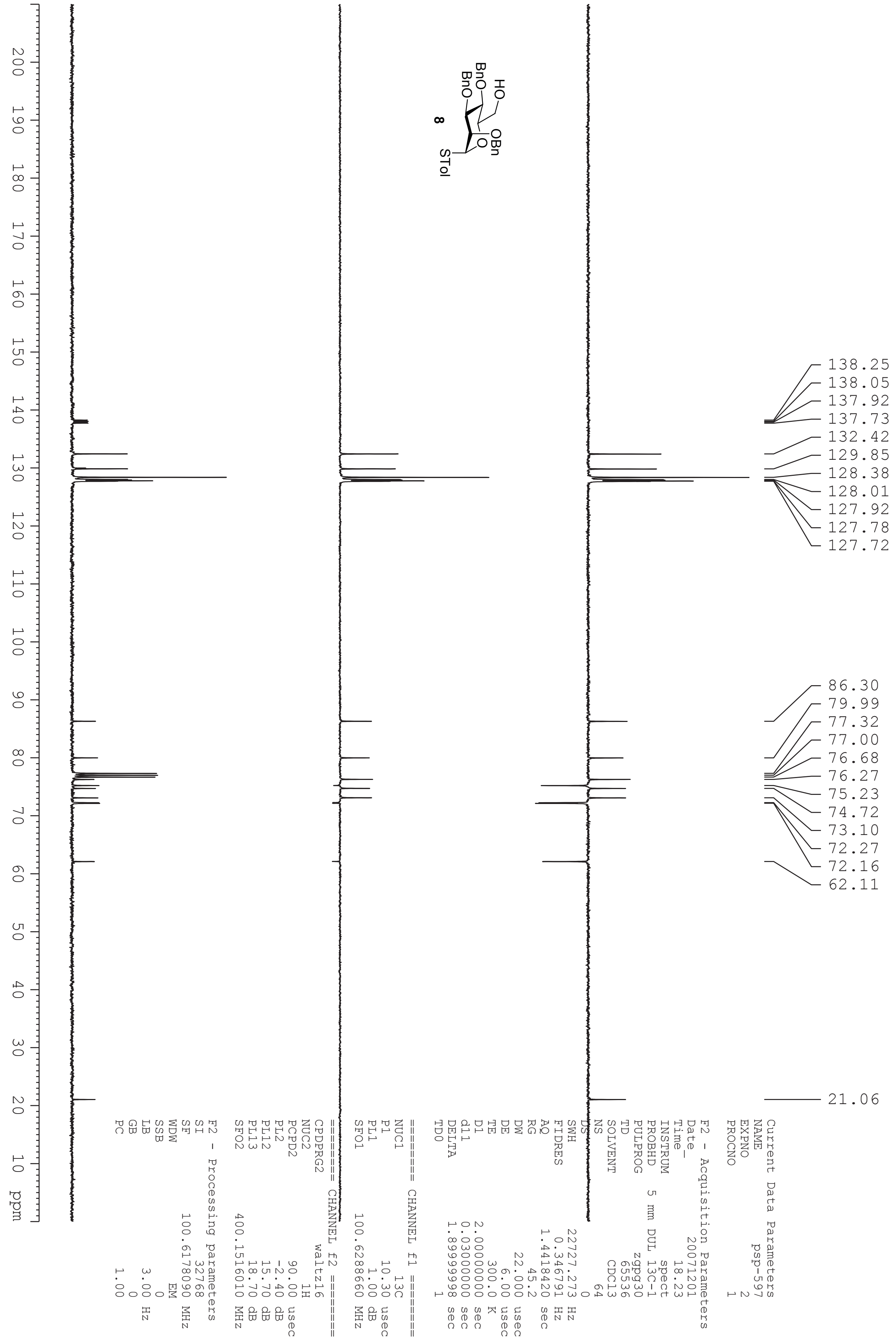

Supplementary Figure 9. ^{13}C and DEPT NMR spectra of compound 8.

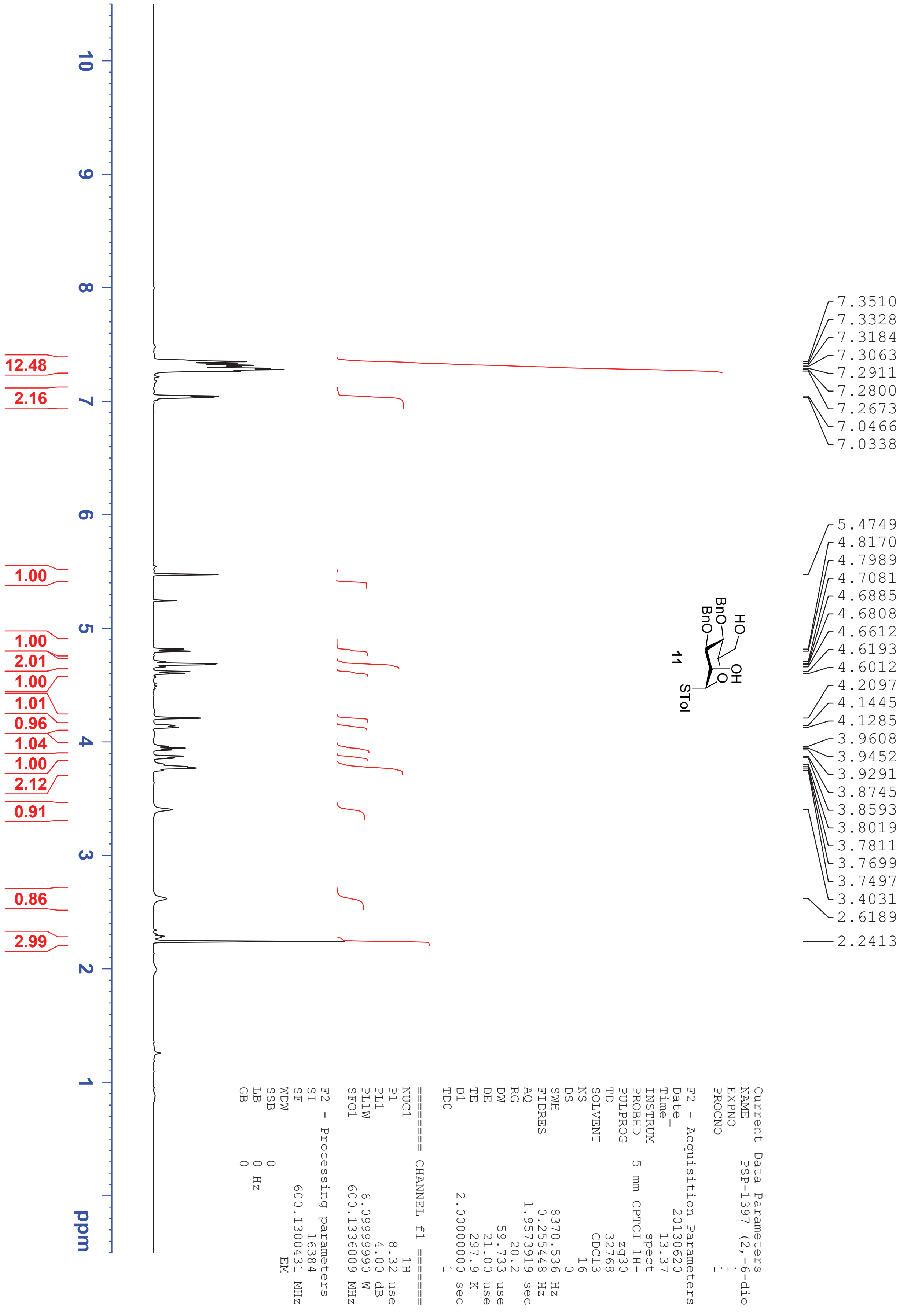

Supplementary Figure 10. **^{1}H NMR spectrum of compound 11.**

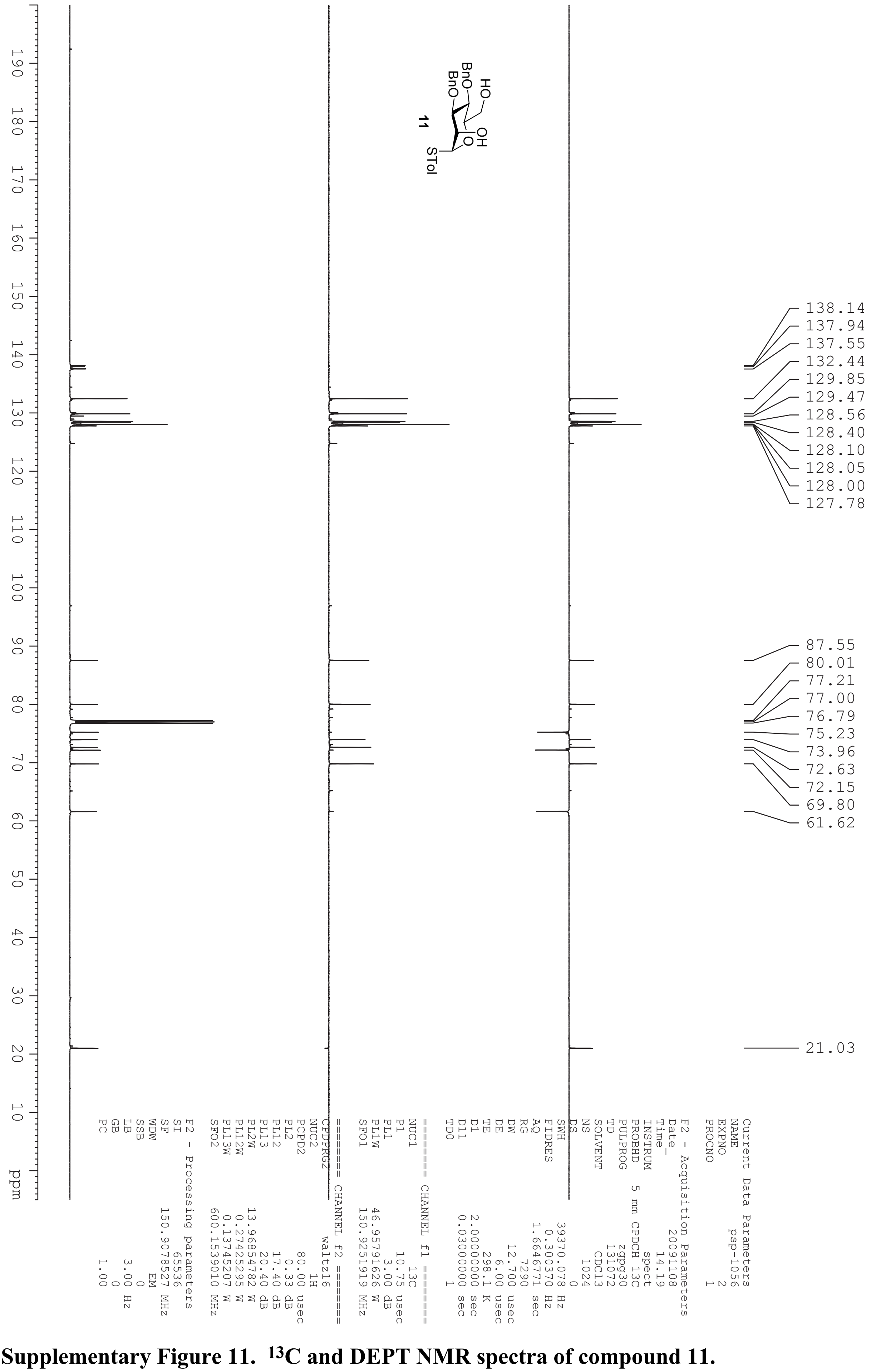

Supplementary Figure 11. ^{13}C and DEPT NMR spectra of compound 11.

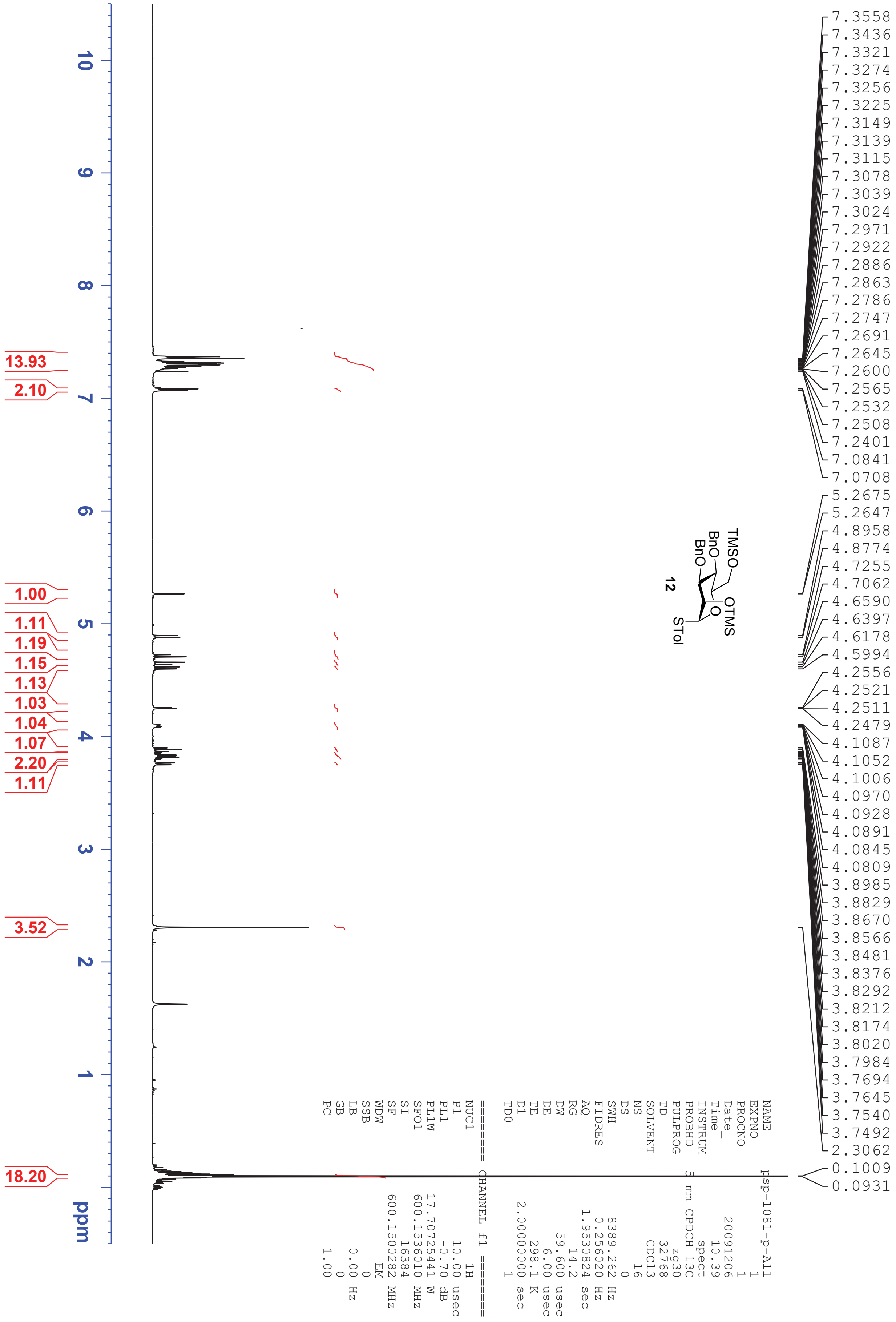

Supplementary Figure 12. ^{1}H NMR spectrum of compound 12.

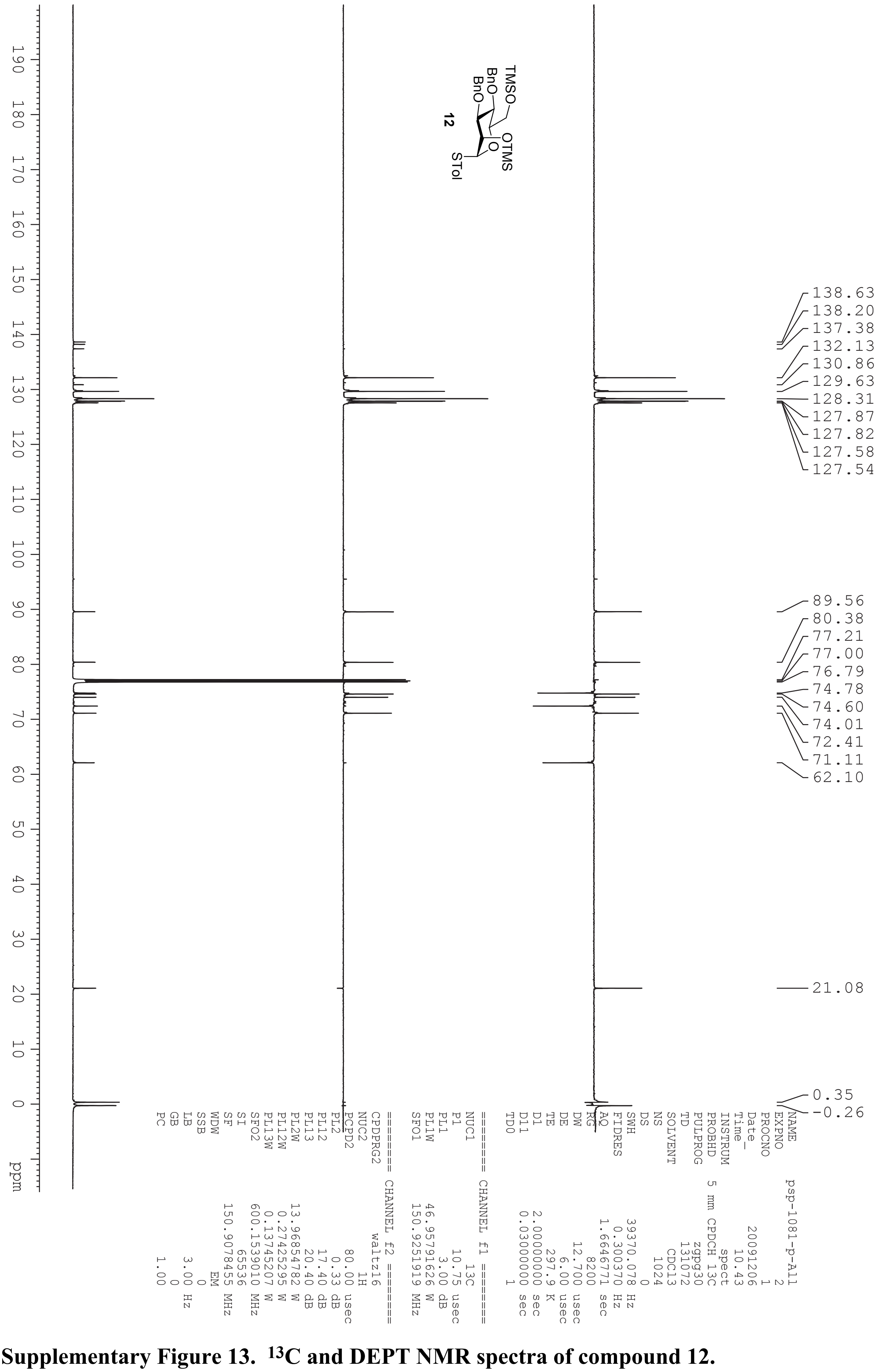

Supplementary Figure 13. ^{13}C and DEPT NMR spectra of compound 12.

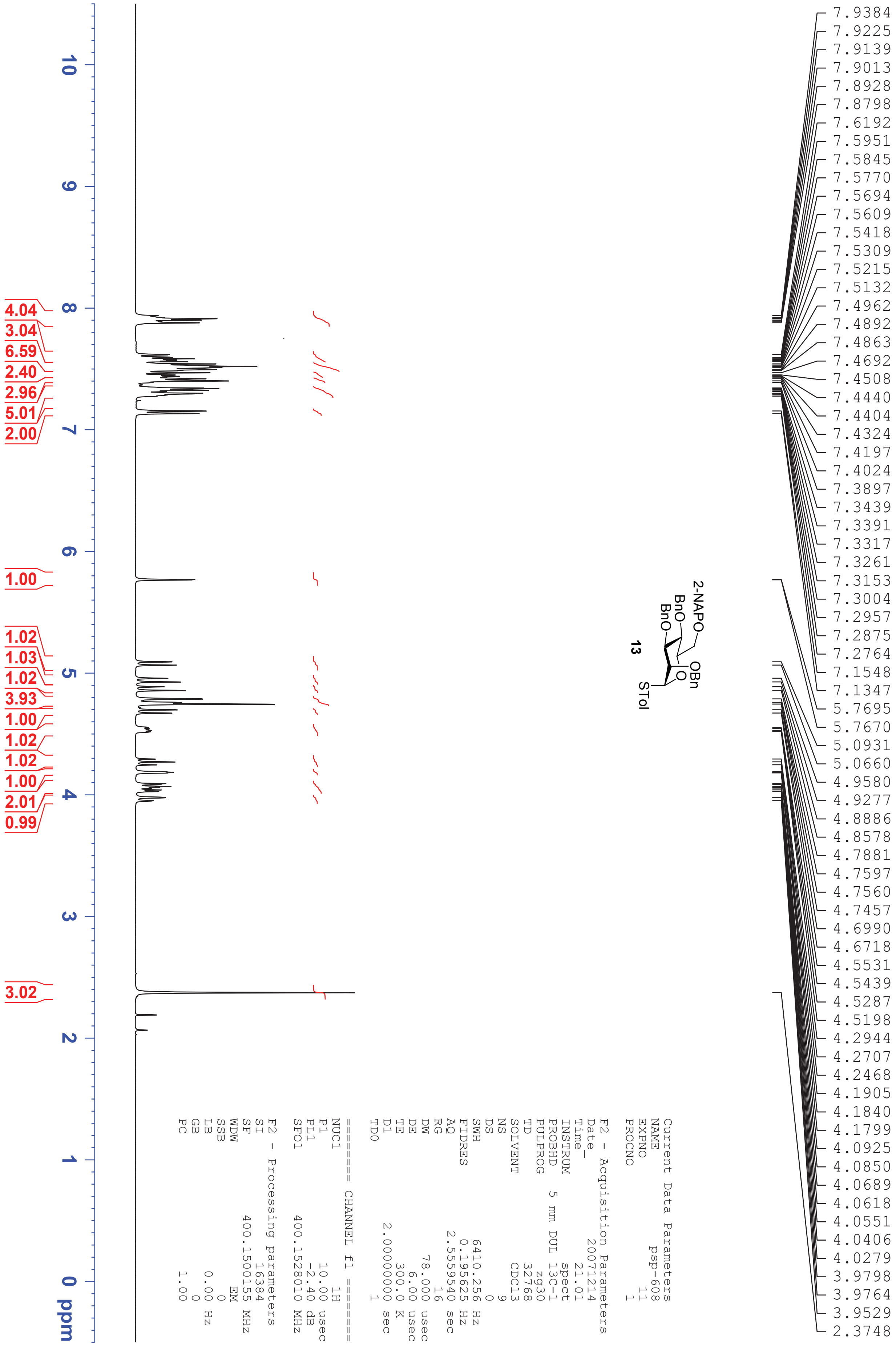

Supplementary Figure 14. **^{1}H NMR spectrum of compound 13.**

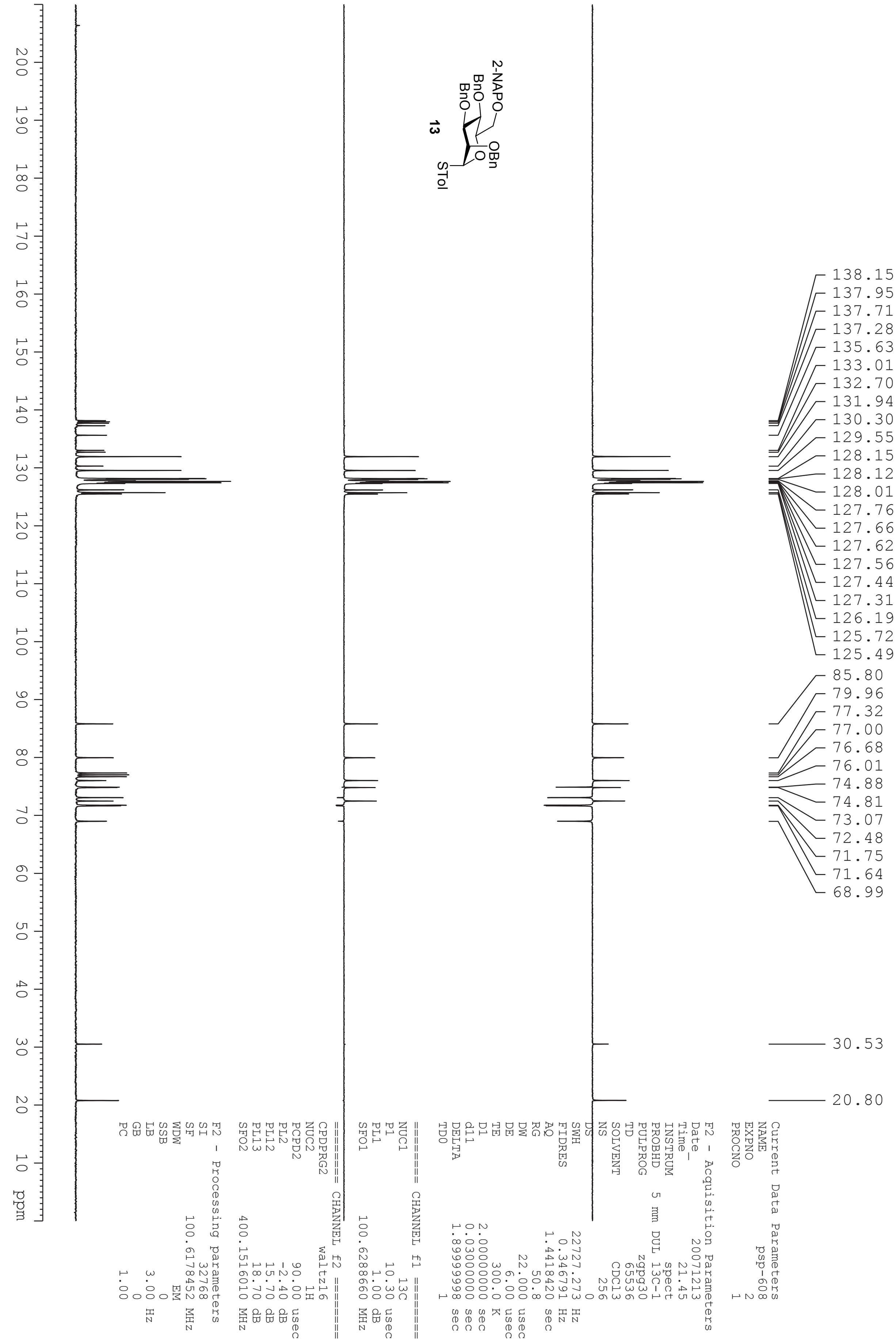

Supplementary Figure 15. ^{13}C and DEPT NMR spectra of compound 13.

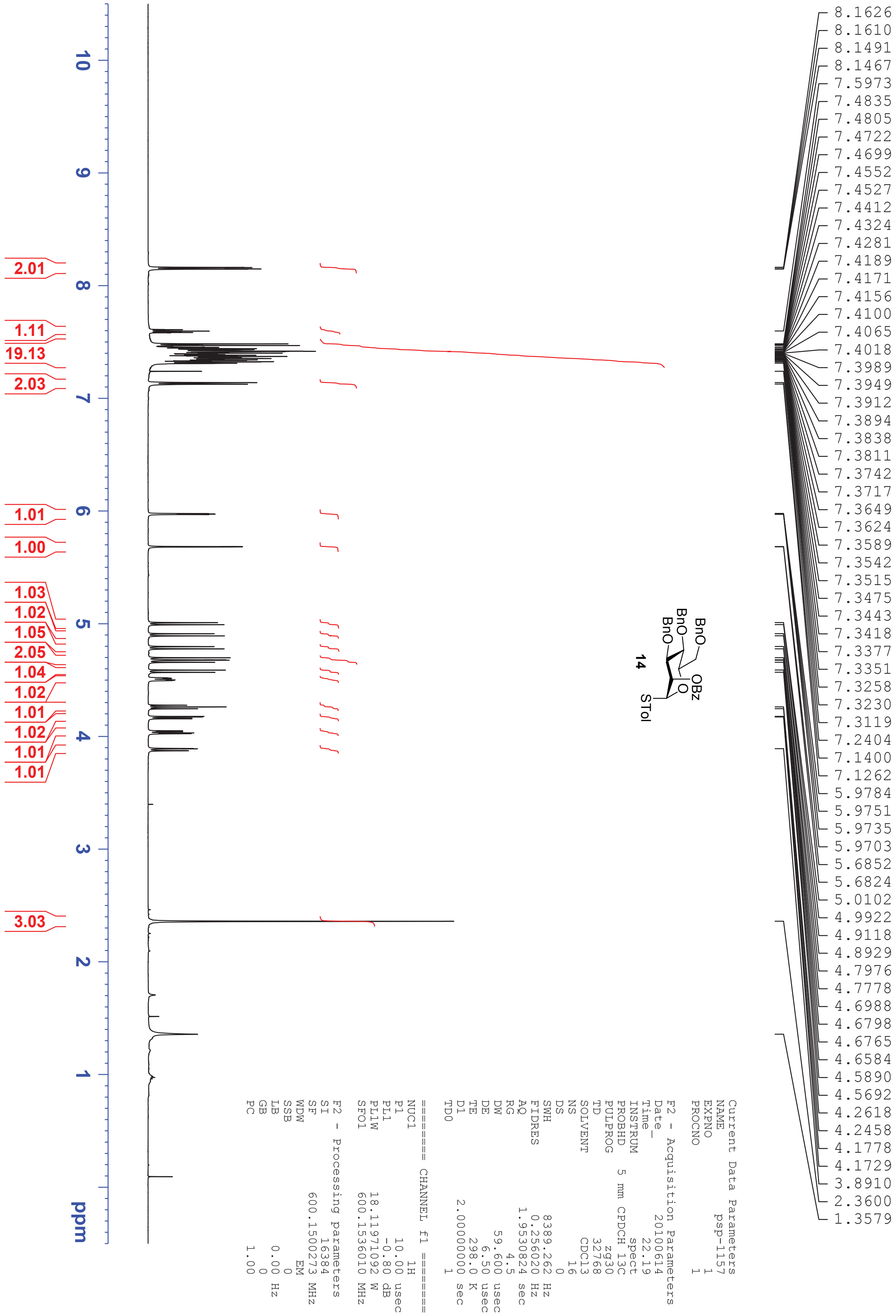

Supplementary Figure 16. ^{1}H NMR spectrum of compound 14.

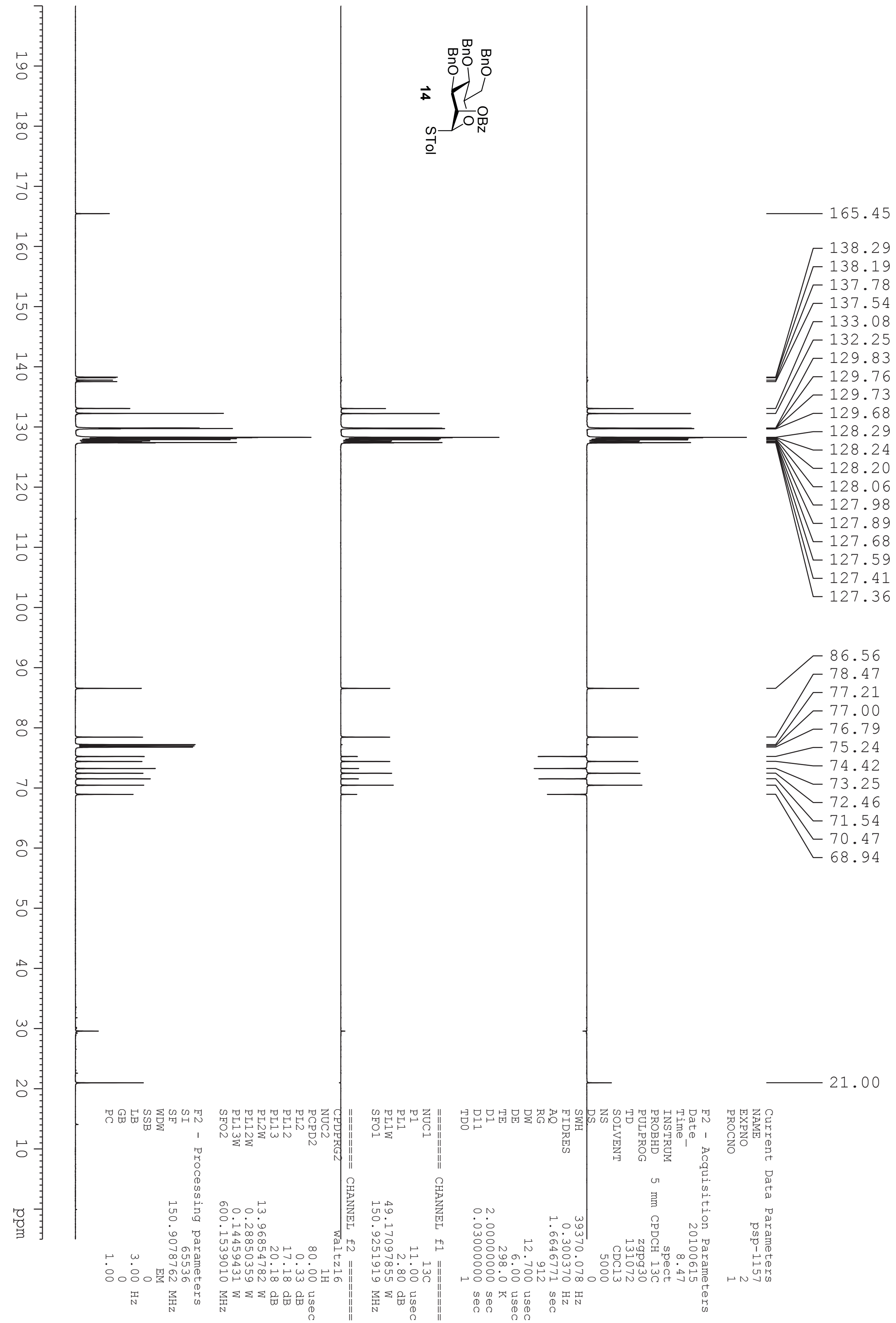

Supplementary Figure 17. ^{13}C and DEPT NMR spectra of compound 14.

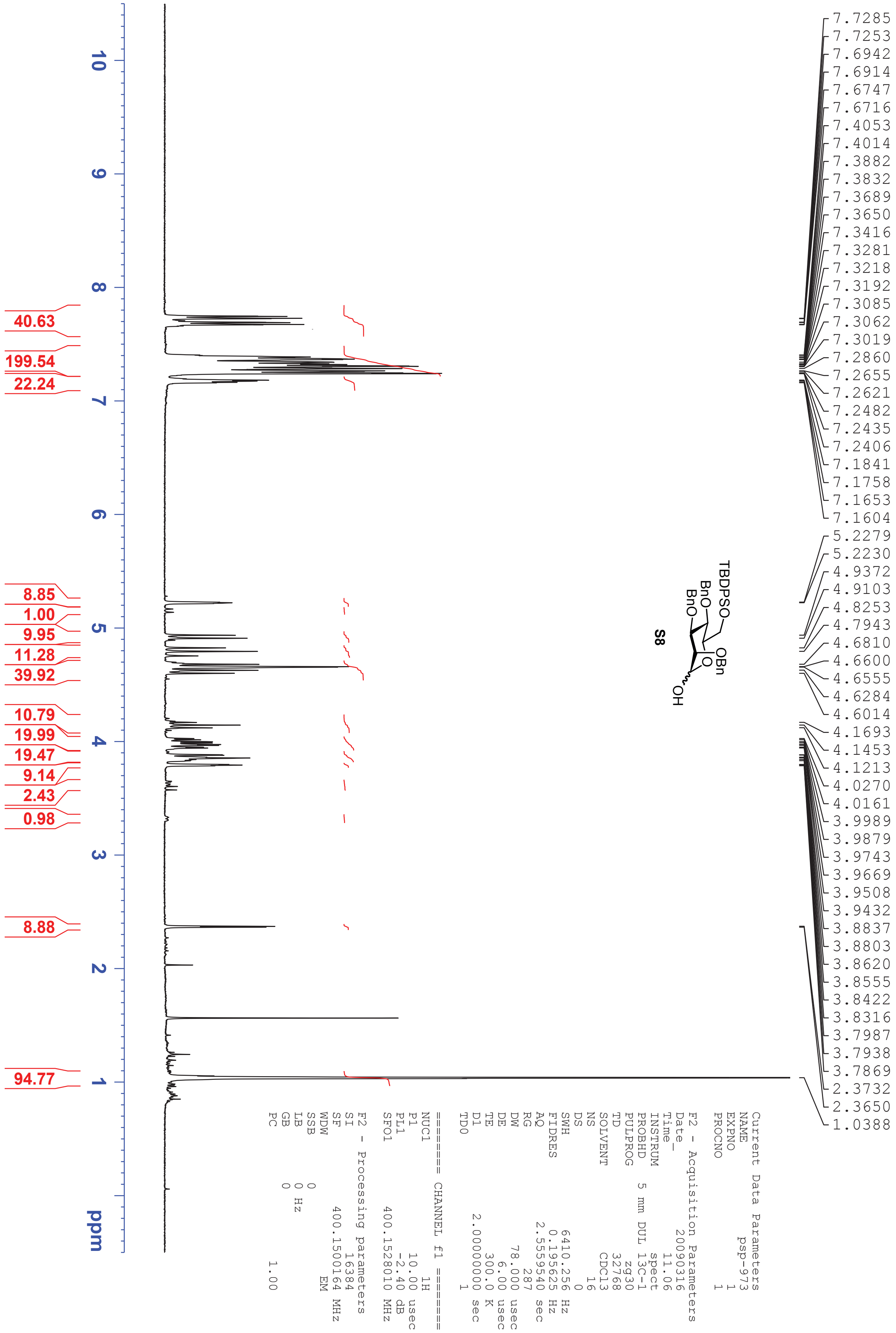

Supplementary Figure 18. ^{1}H NMR spectrum of compound S8.

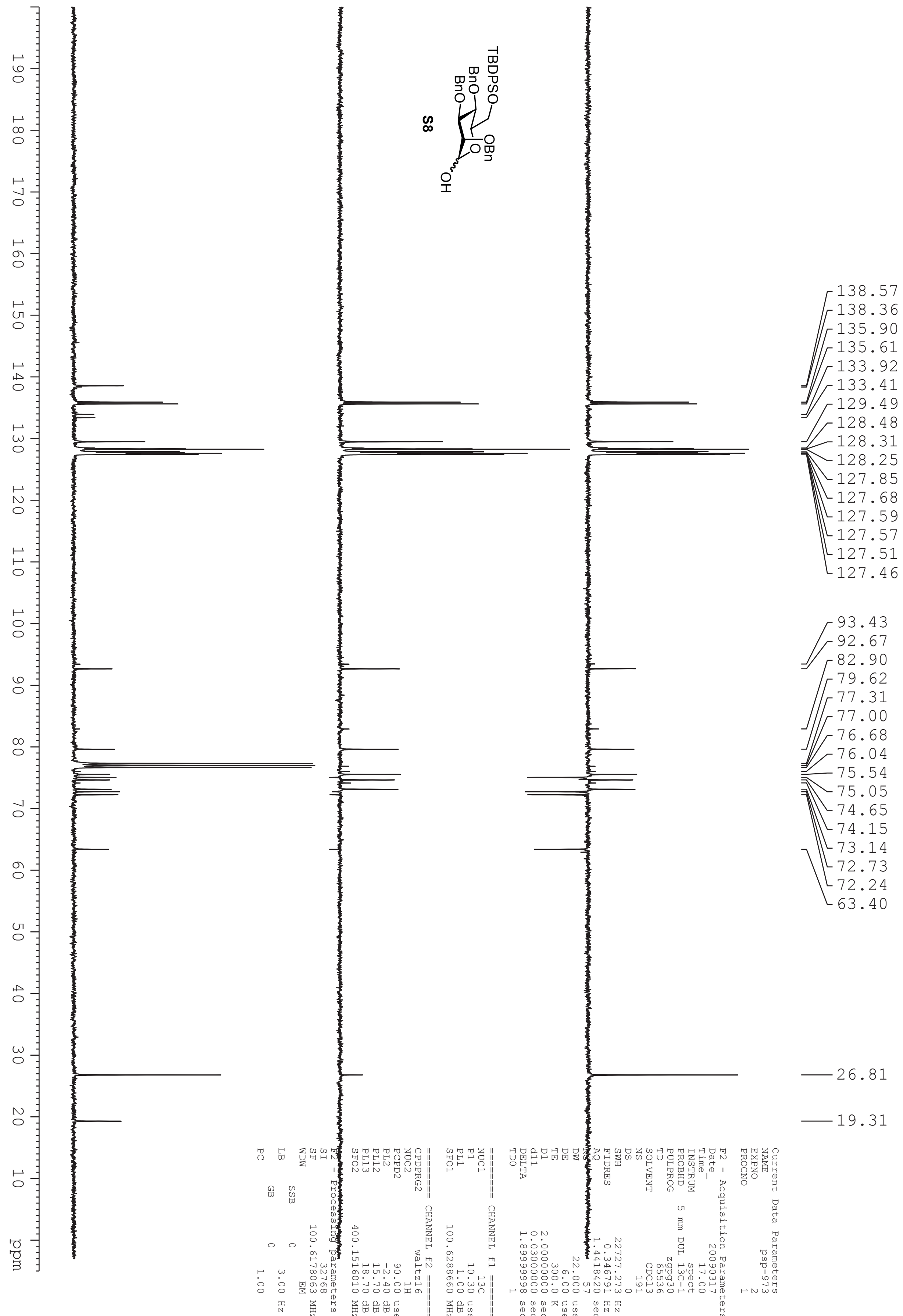

Supplementary Figure 19. ^{13}C and DEPT NMR spectra of compound S8.

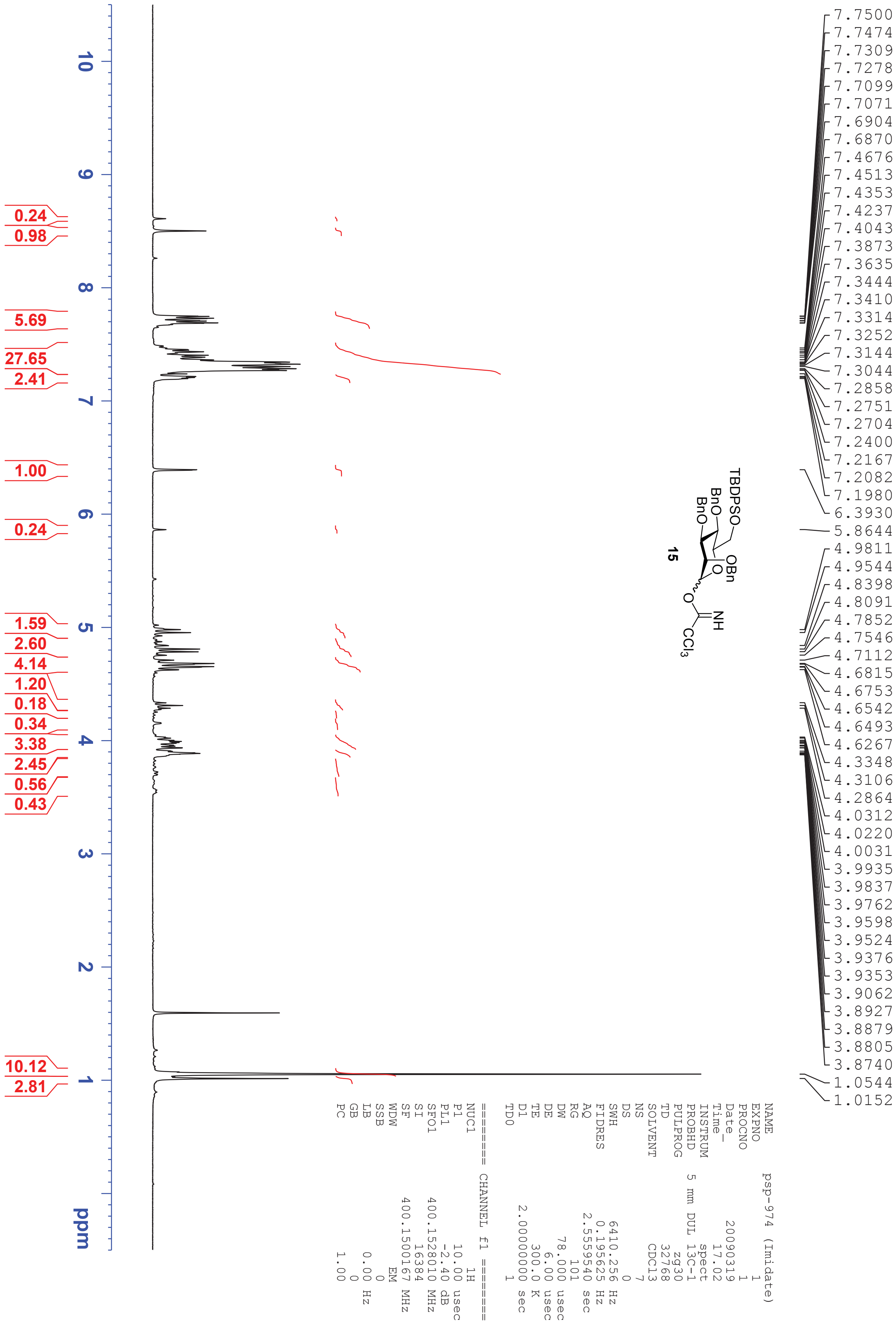

Supplementary Figure 20. **^{1}H NMR spectrum of compound 15.**

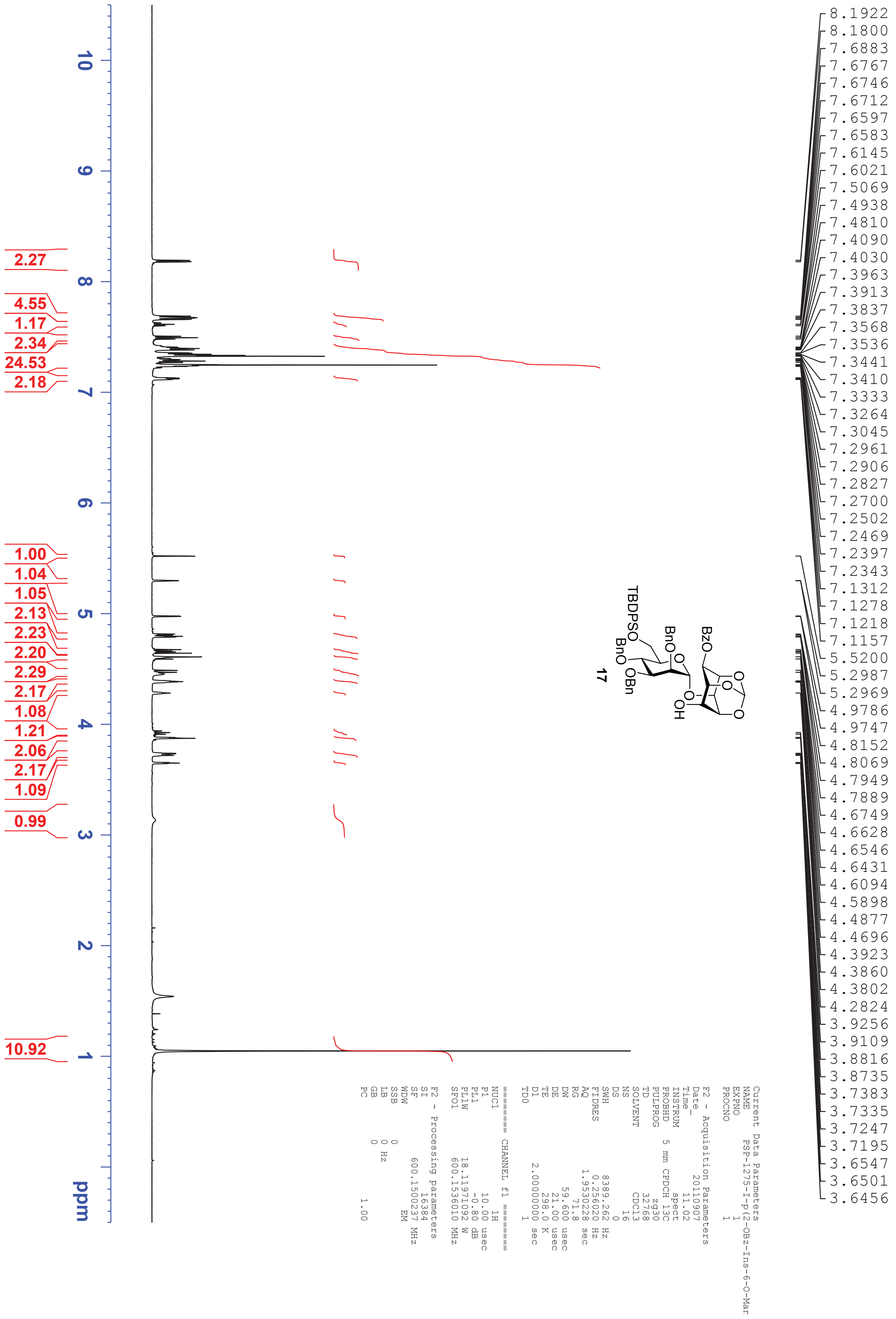

Supplementary Figure 21. ^{1}H NMR spectrum of compound 17.

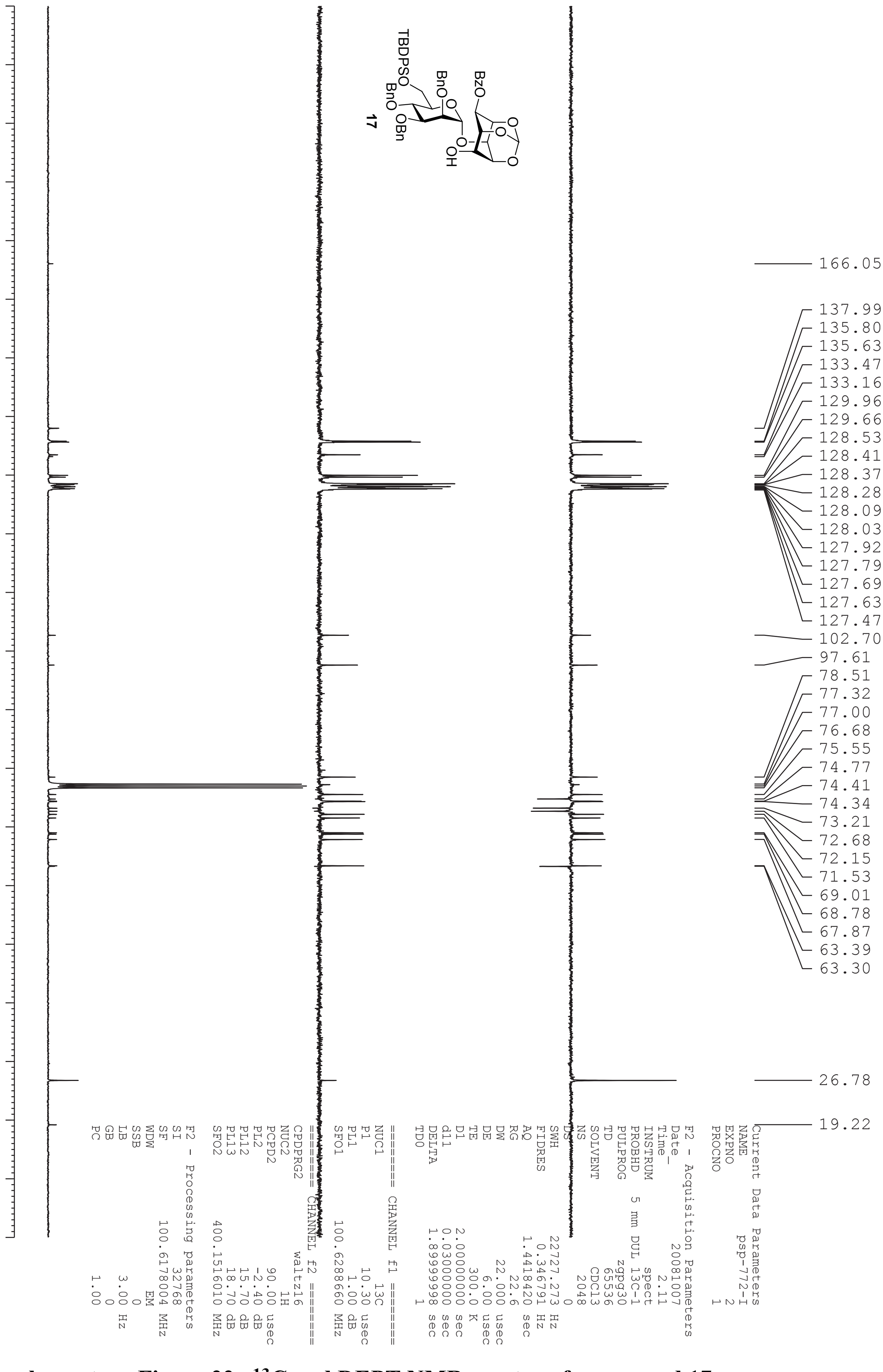

Supplementary Figure 22. ^{13}C and DEPT NMR spectra of compound 17.

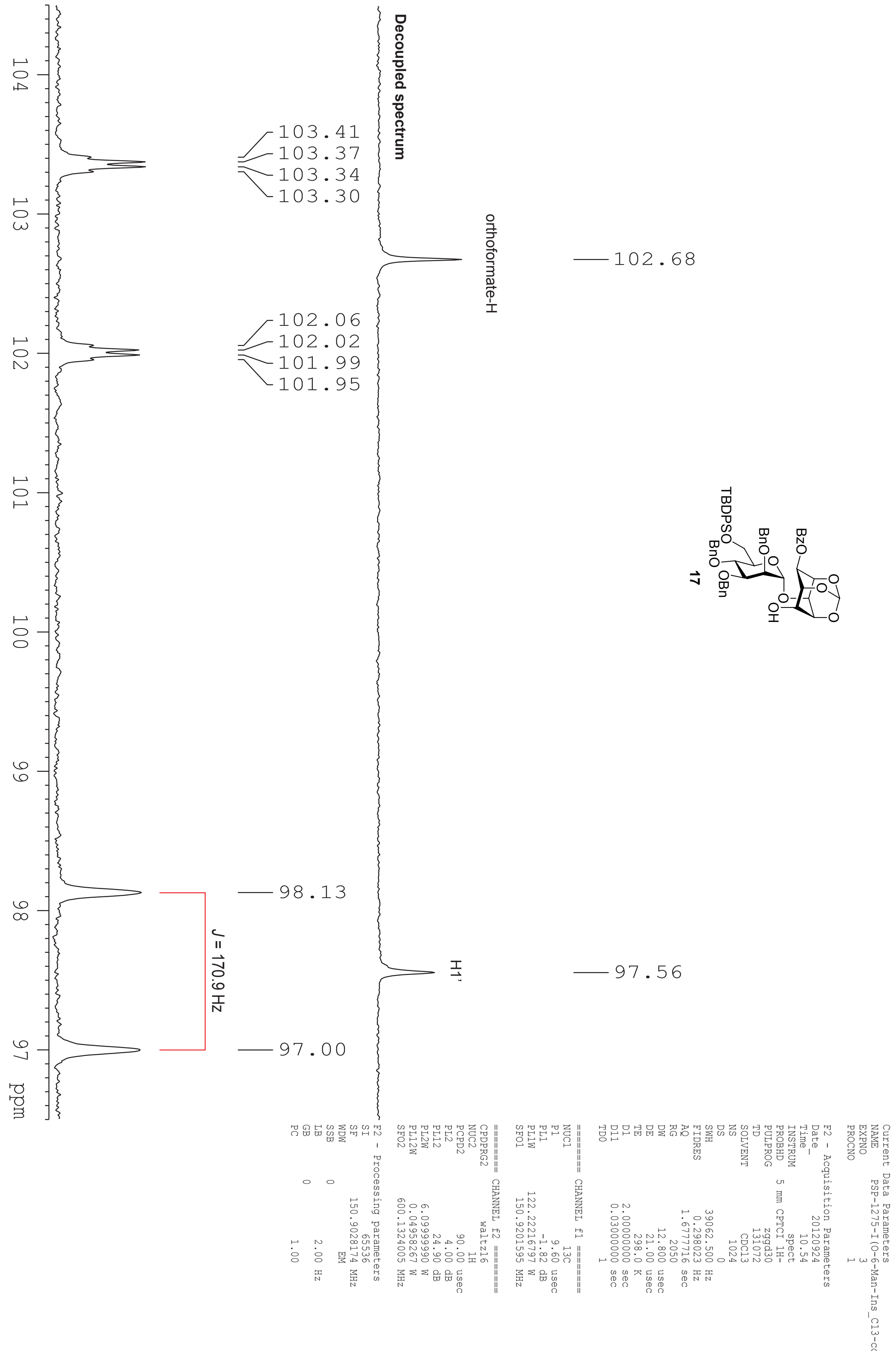

Supplementary Figure 23. Non-decoupled ^{13}C NMR spectrum of compound 17.

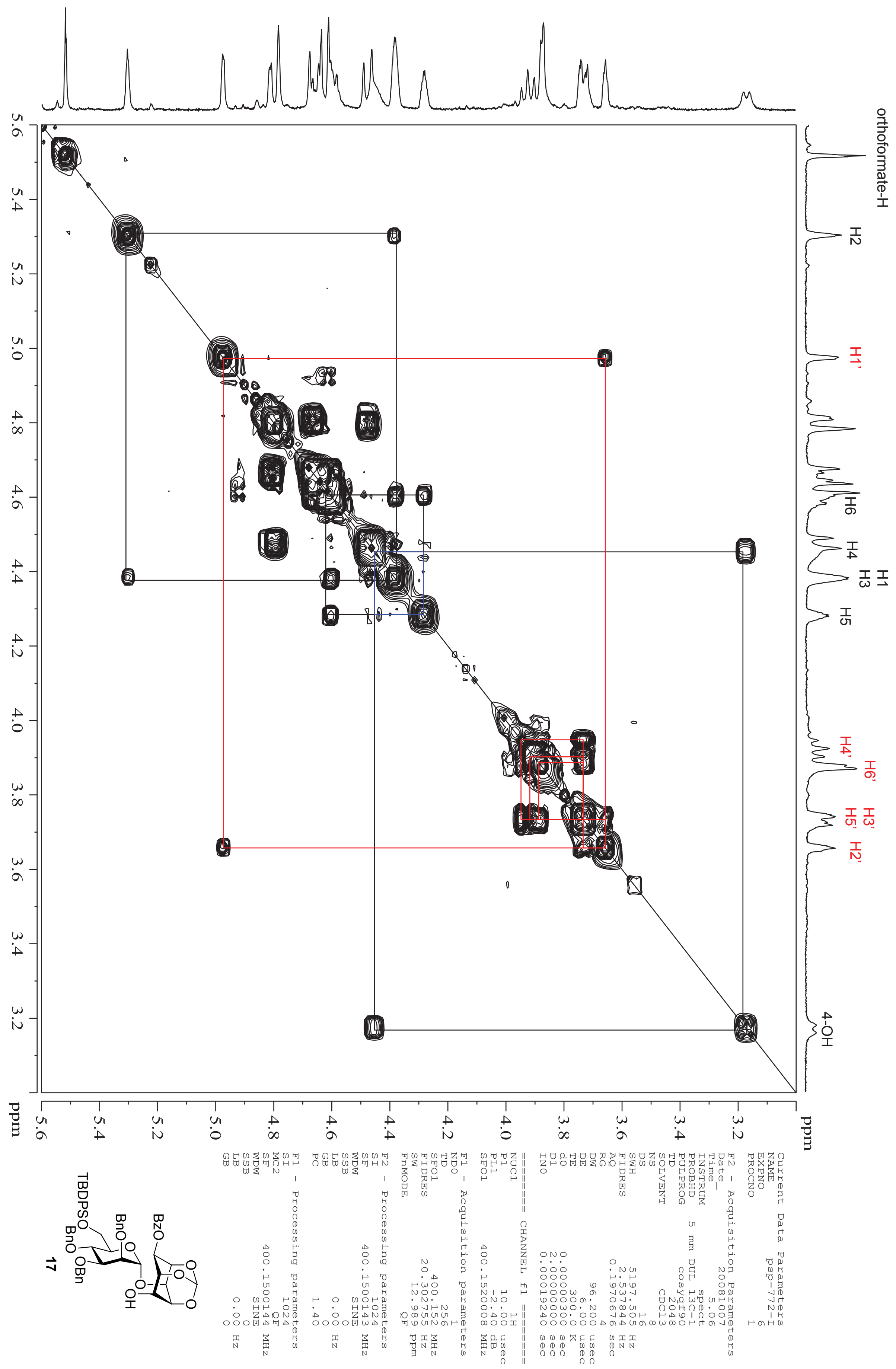

Supplementary Figure 24. COSY NMR spectrum of compound 17.

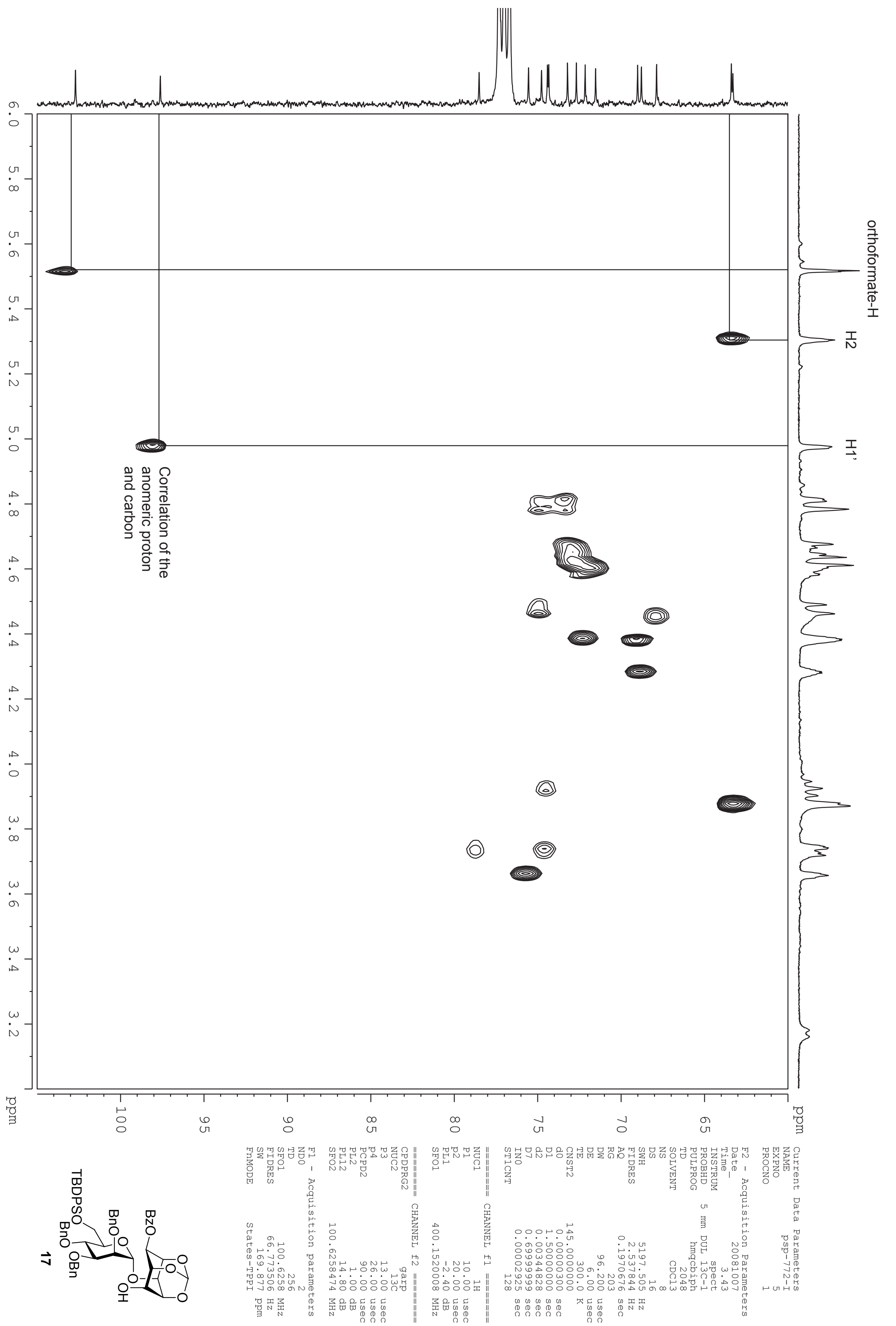

Supplementary Figure 25. HMQC NMR spectrum of compound 17.

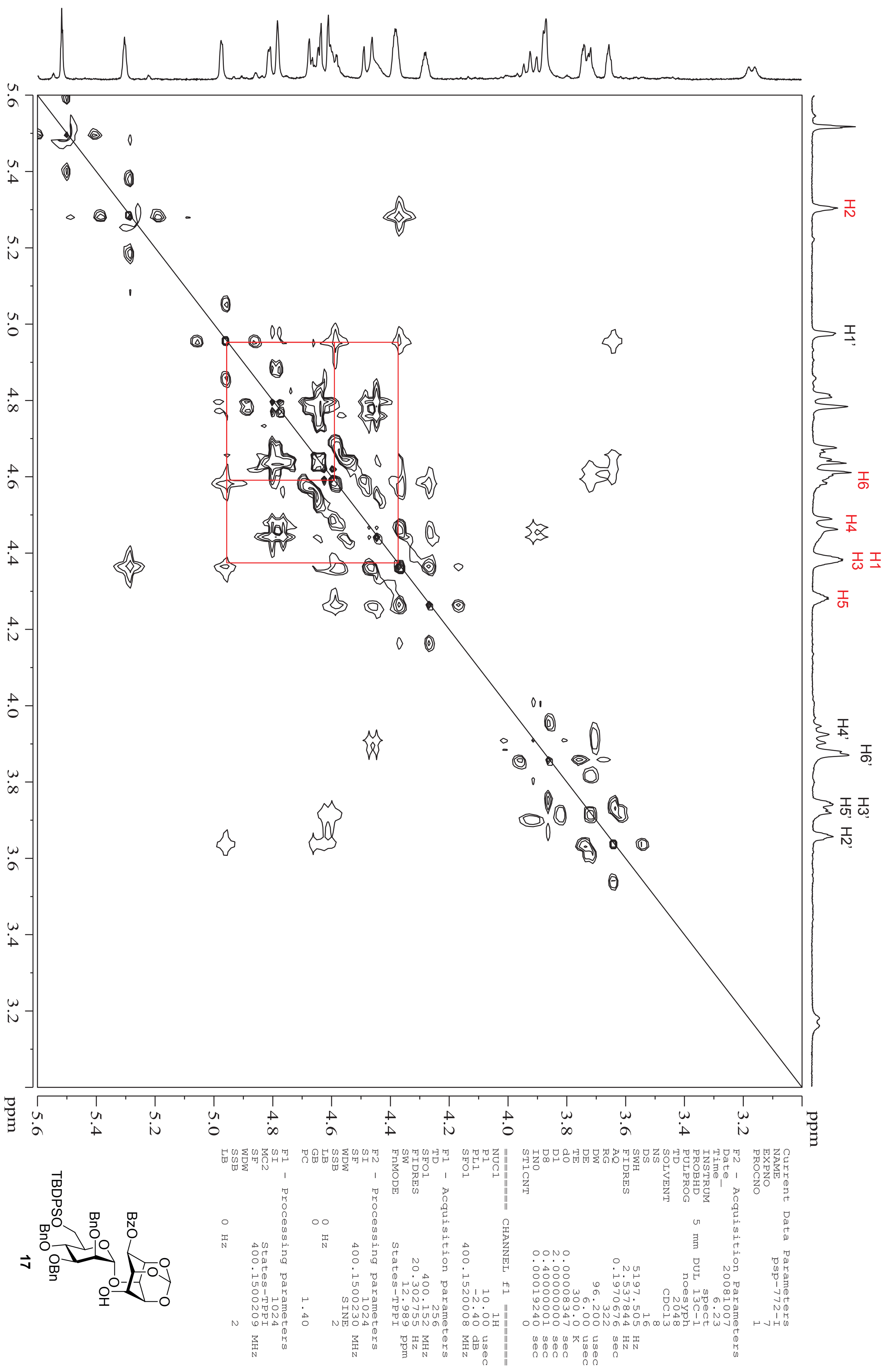

Supplementary Figure 26. NOESY NMR spectrum of compound 17.

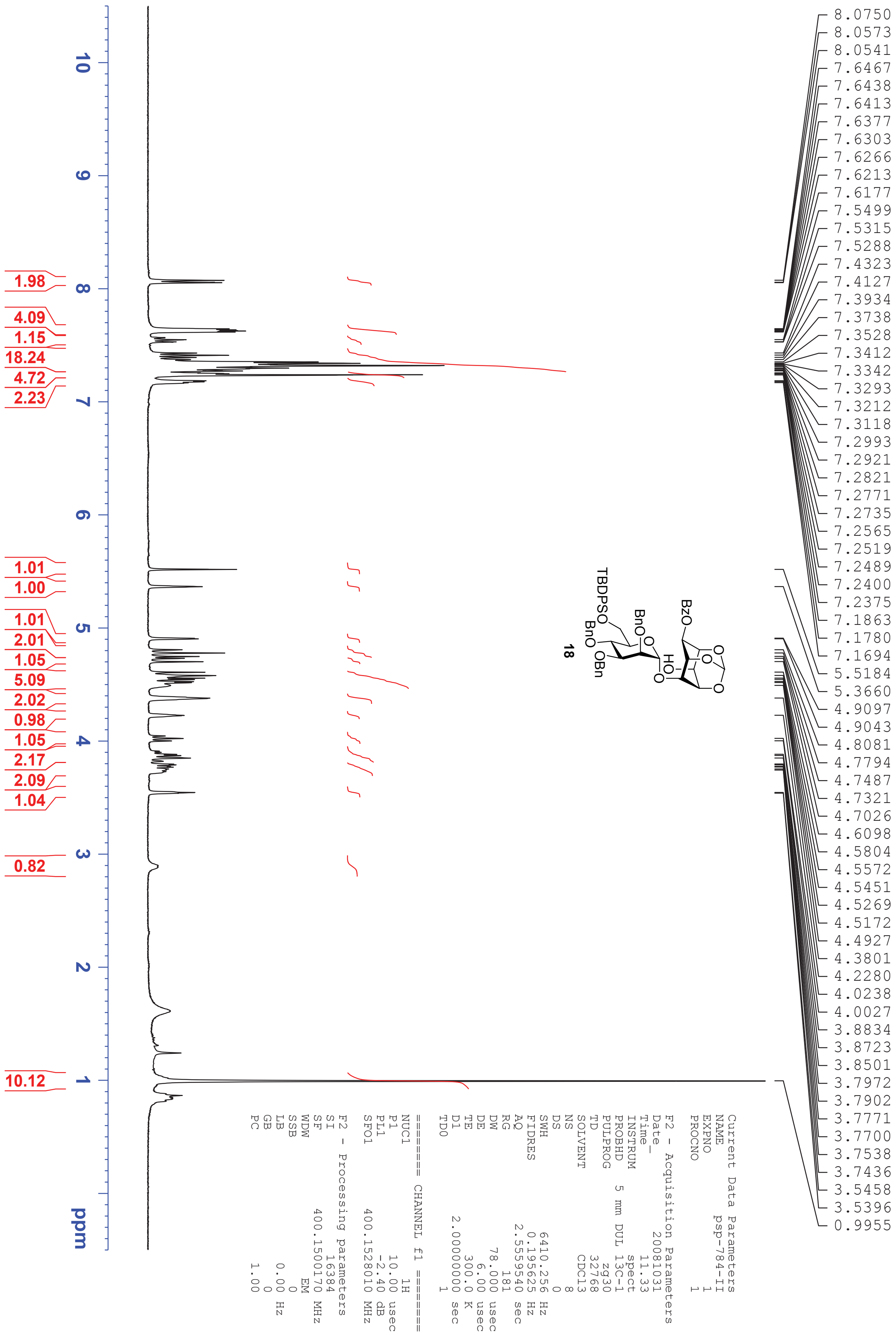

Supplementary Figure 27. ^{1}**H NMR spectrum of compound 18.**

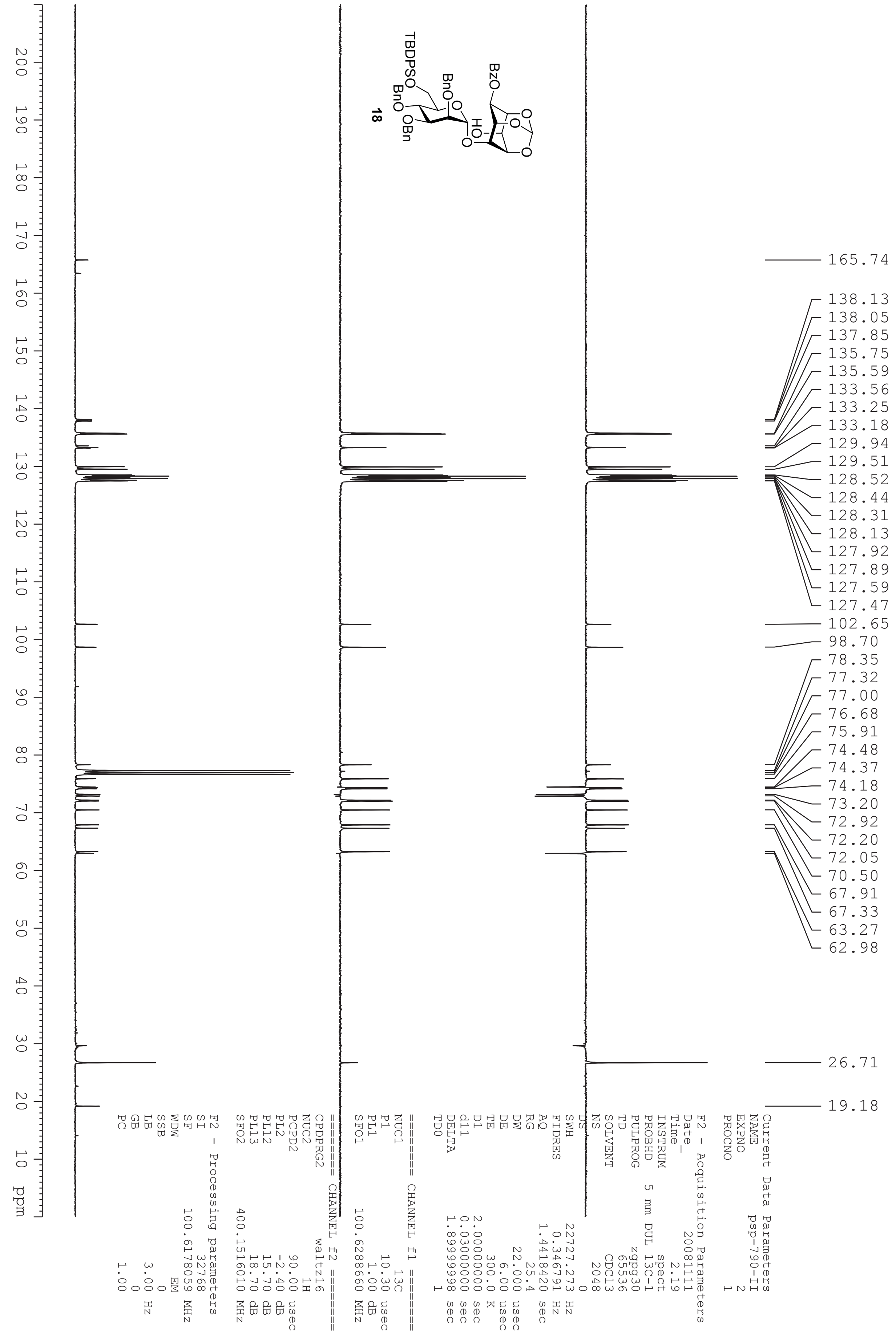

Supplementary Figure 28. ^{13}C and DEPT NMR spectra of compound 18.

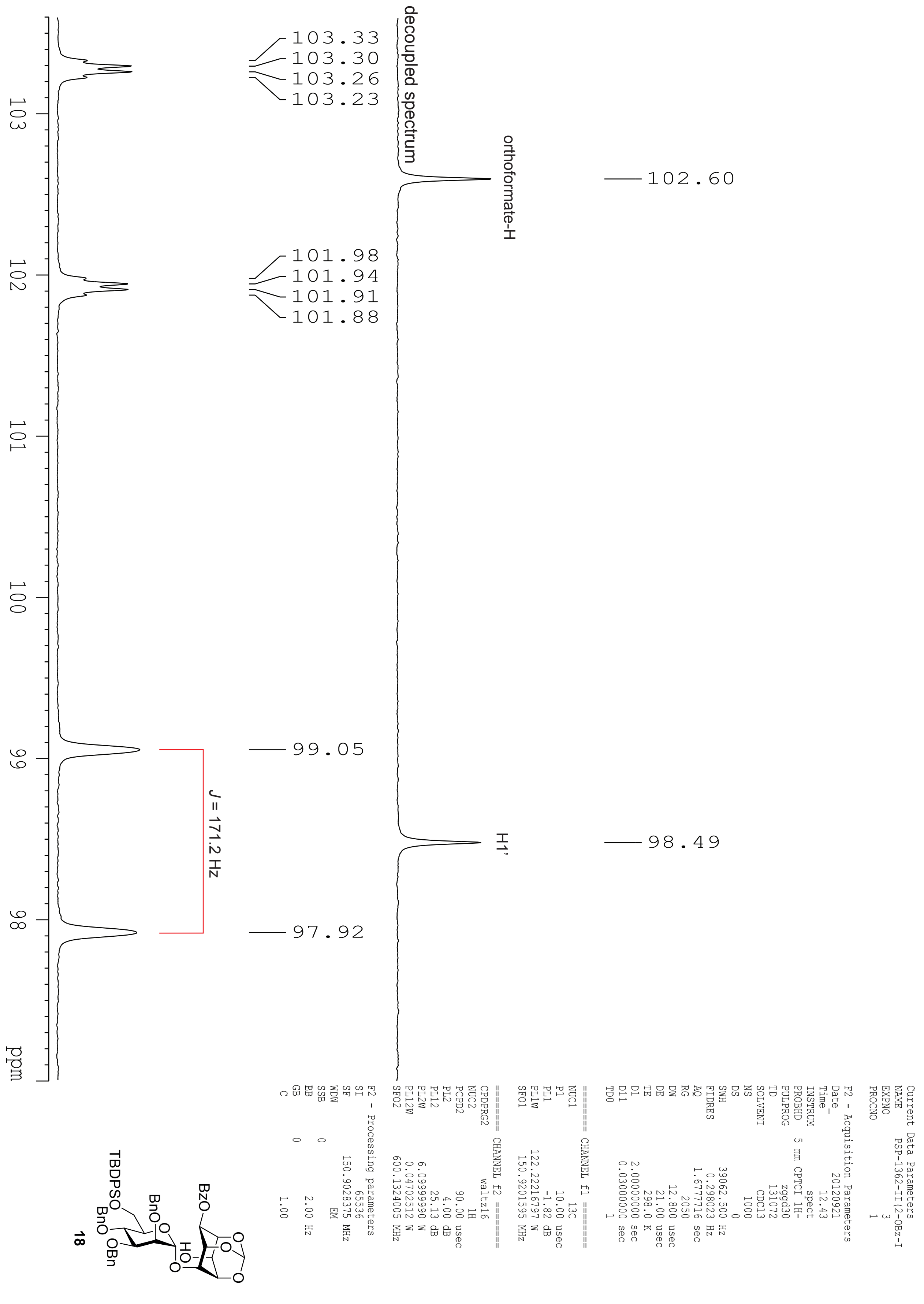

Supplementary Figure 29. Non-decoupled ^{13}C NMR spectrum of compound 18.

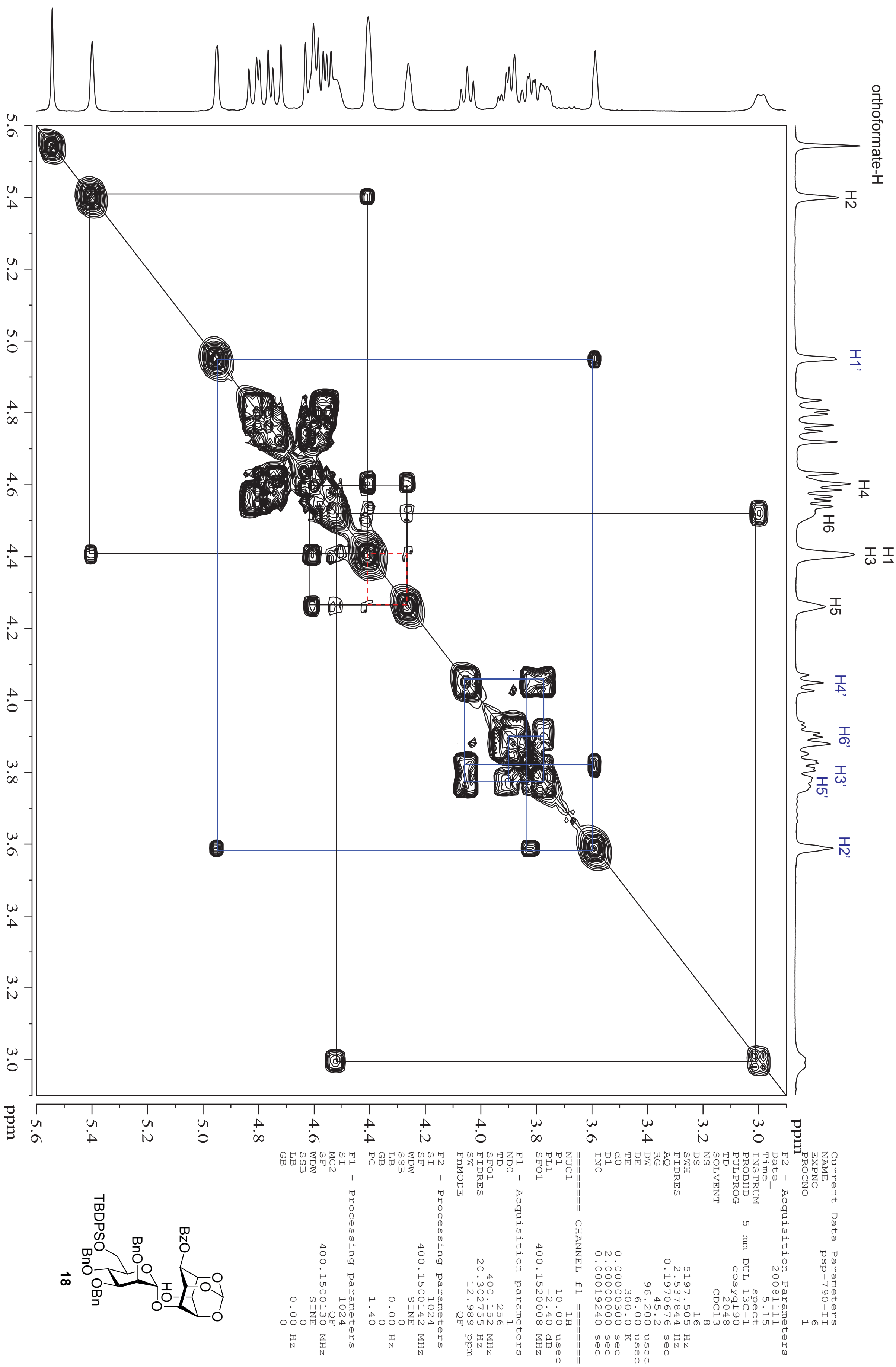

Supplementary Figure 30. COSY NMR spectrum of compound 18.

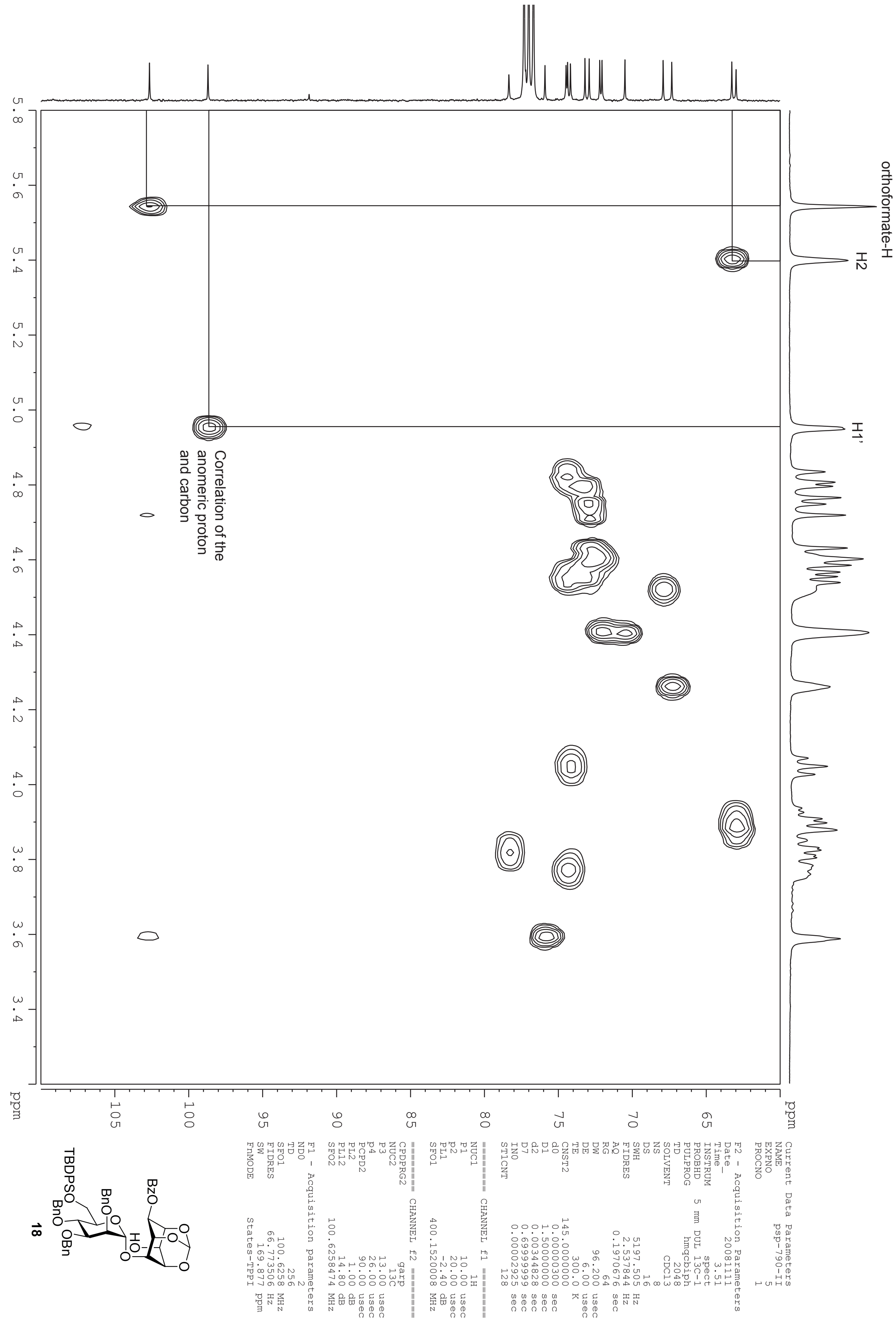

Supplementary Figure 31. HMQC NMR spectrum of compound 18.

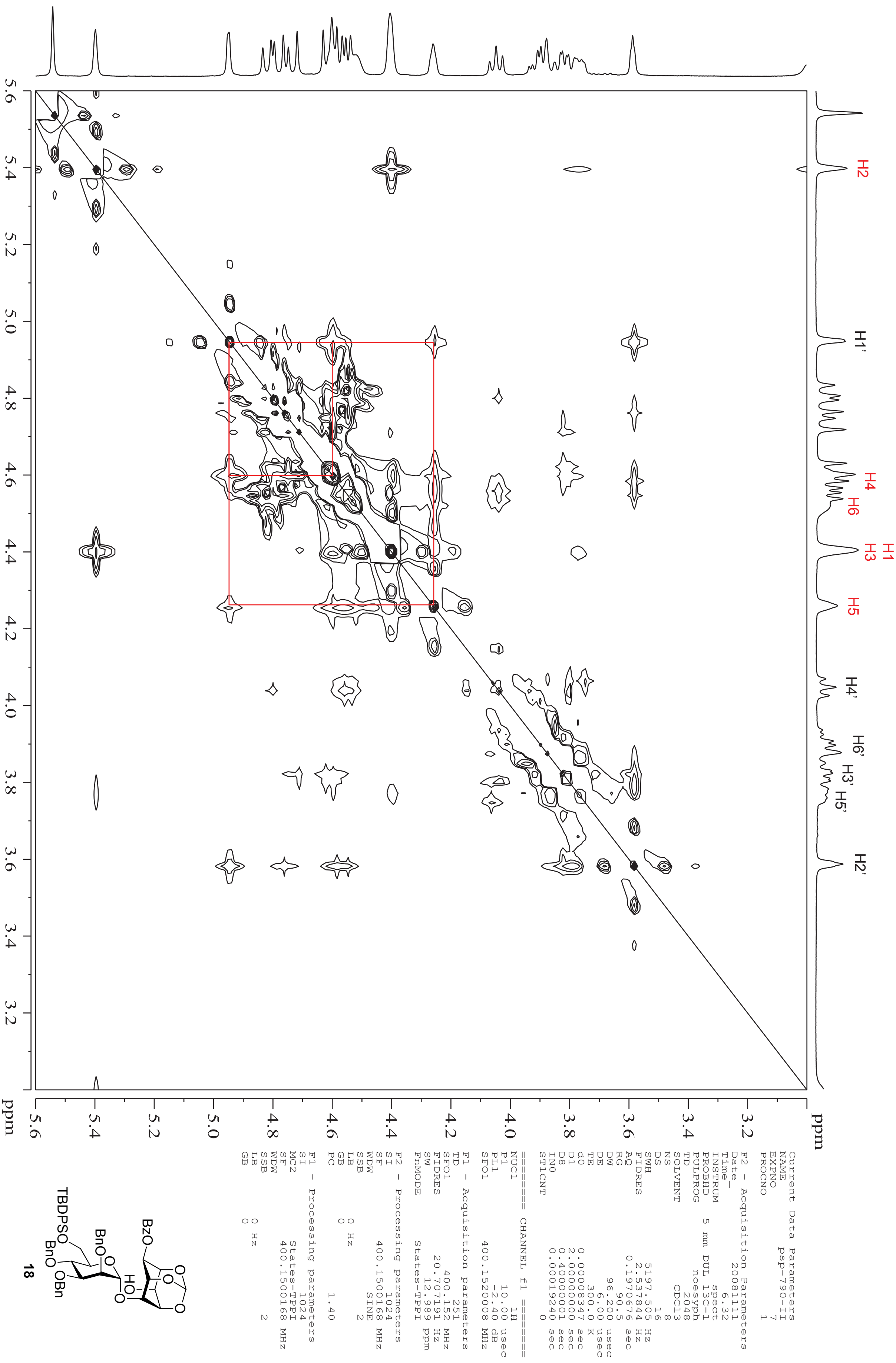

Supplementary Figure 32. NOESY NMR spectrum of compound 18.

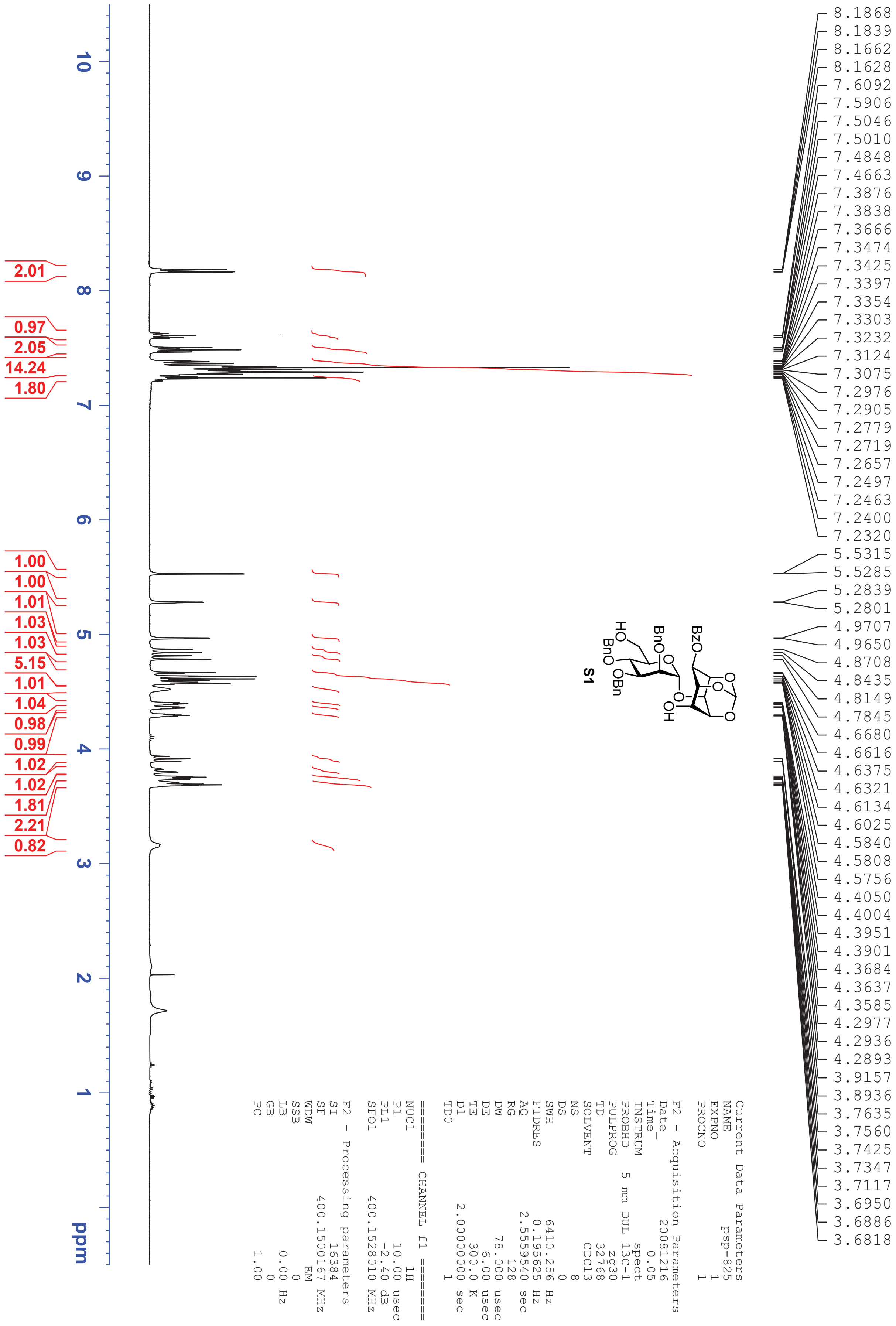

Supplementary Figure 33. **^{1}H NMR spectrum of compound S1.**

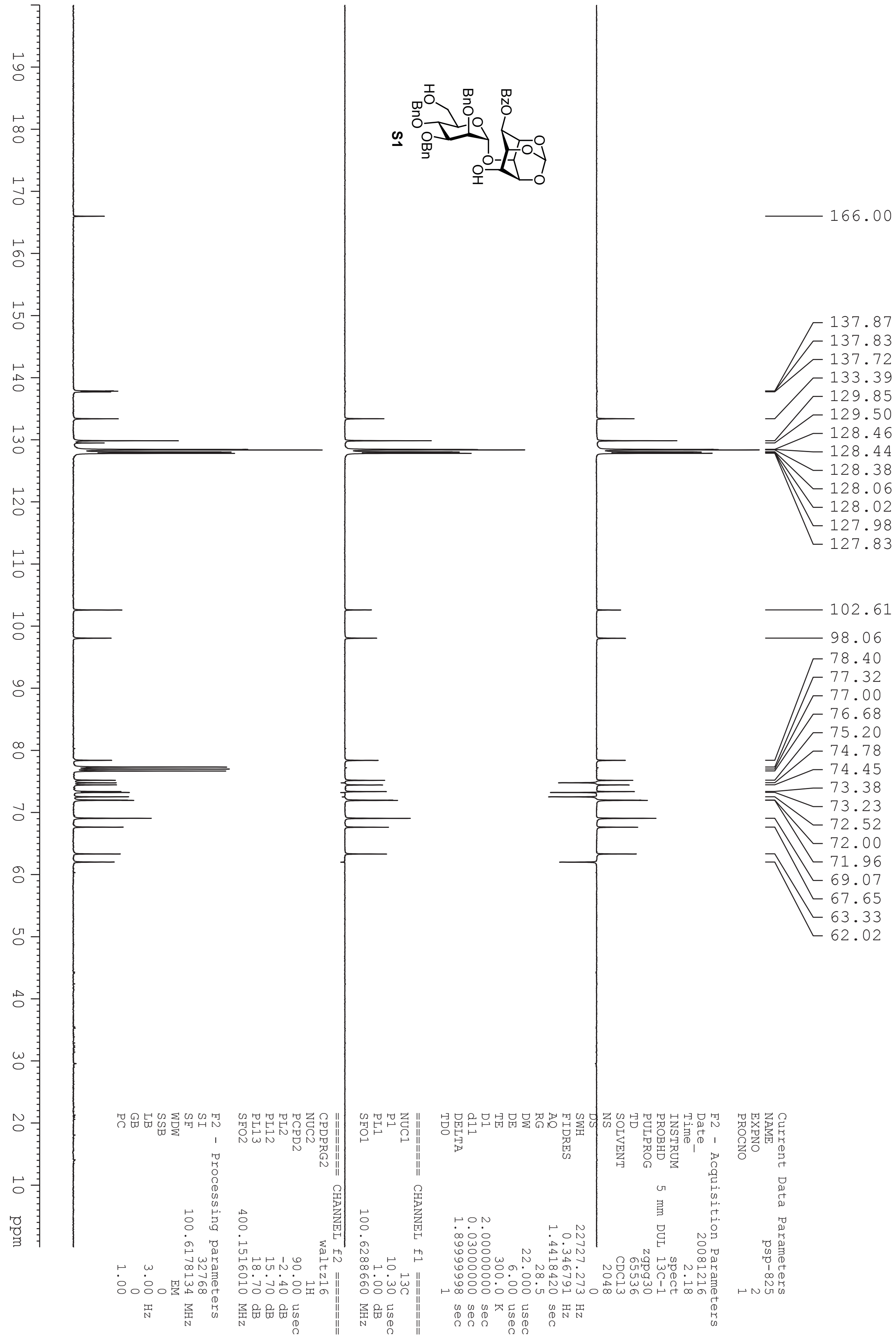

Supplementary Figure 34. ^{13}C and DEPT NMR spectra of compound S1.

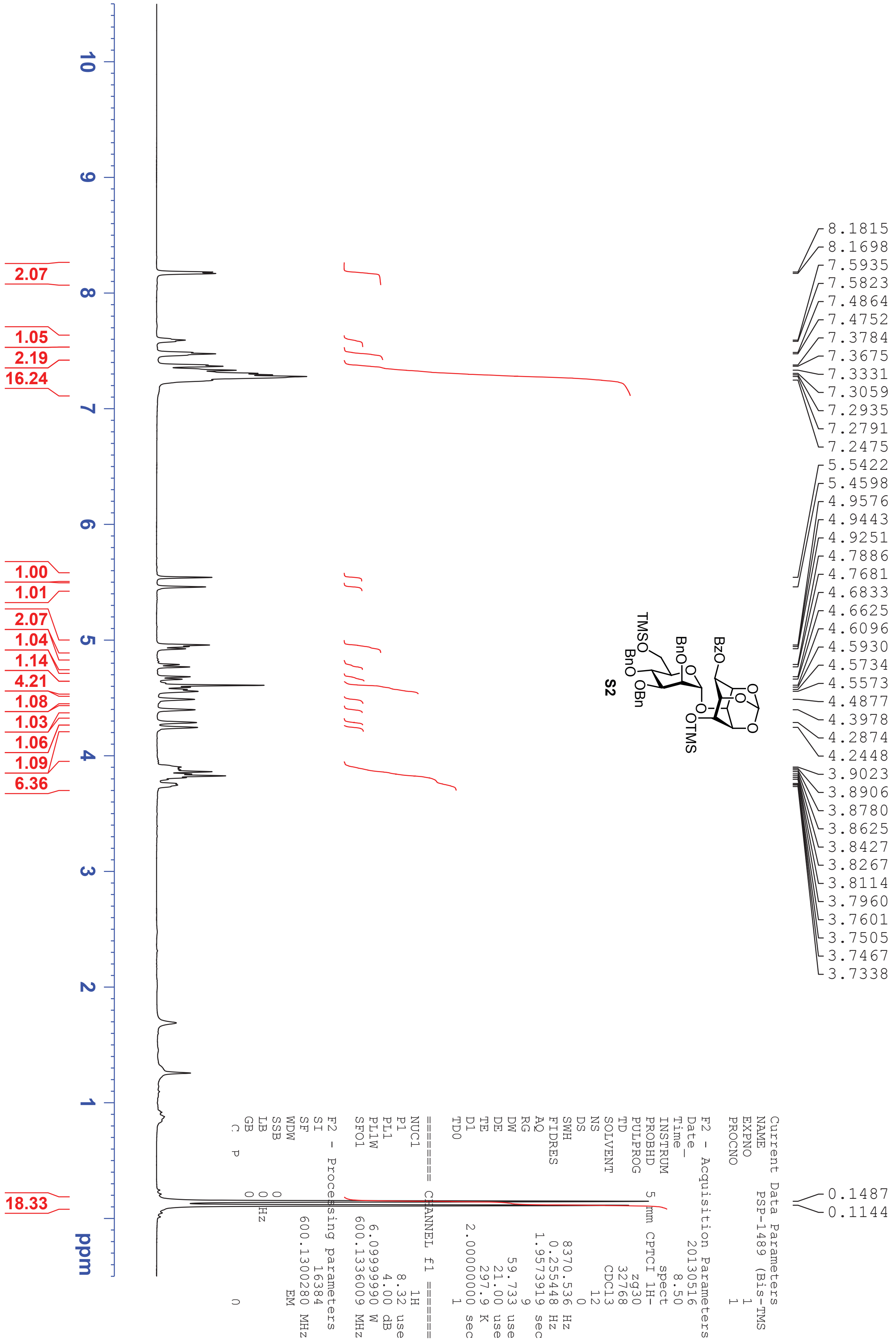

Supplementary Figure 35. ^{1}**H NMR spectrum of compound S2.**

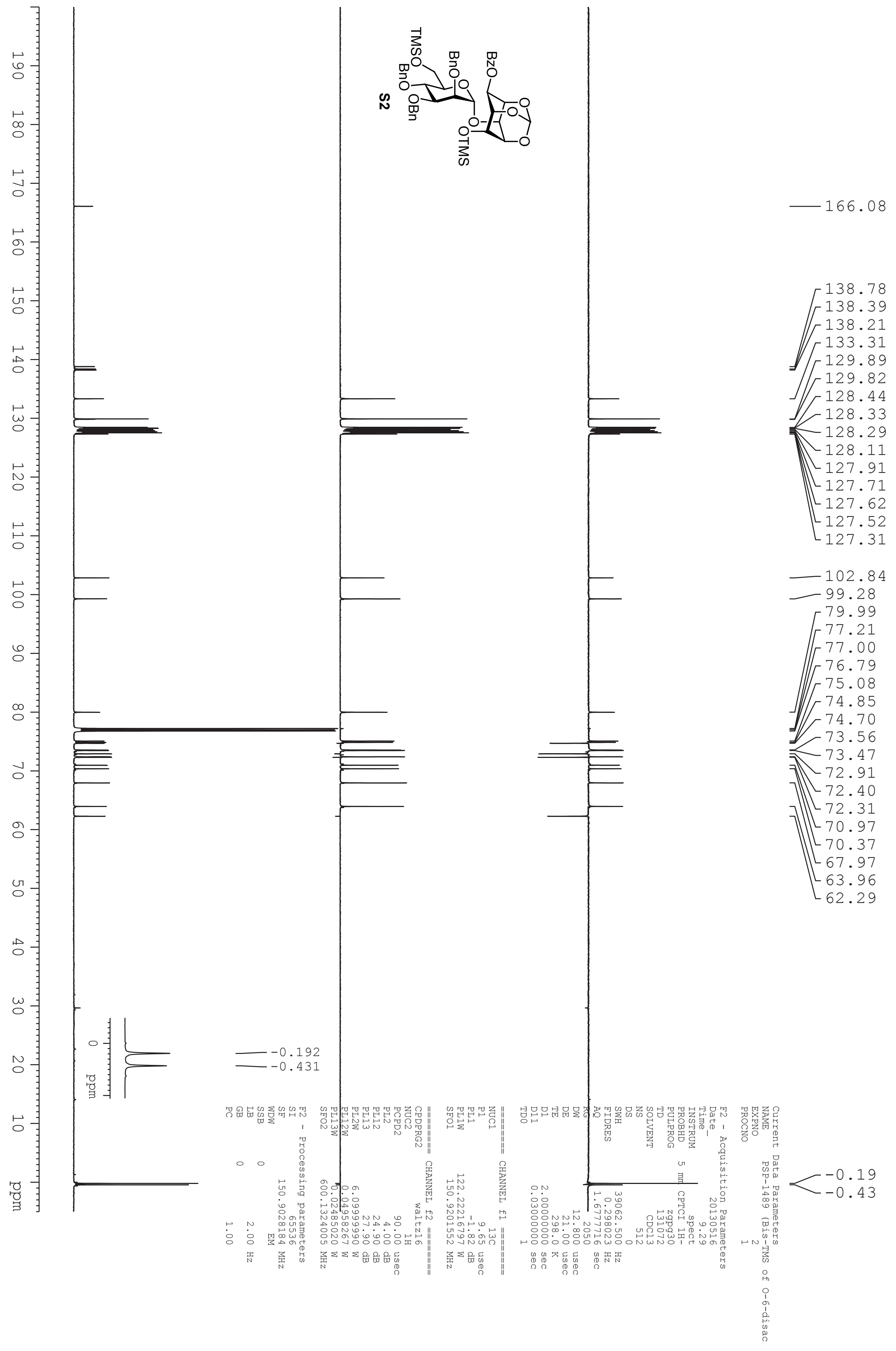

Supplementary Figure 36. ^{13}C and DEPT NMR spectra of compound S2.

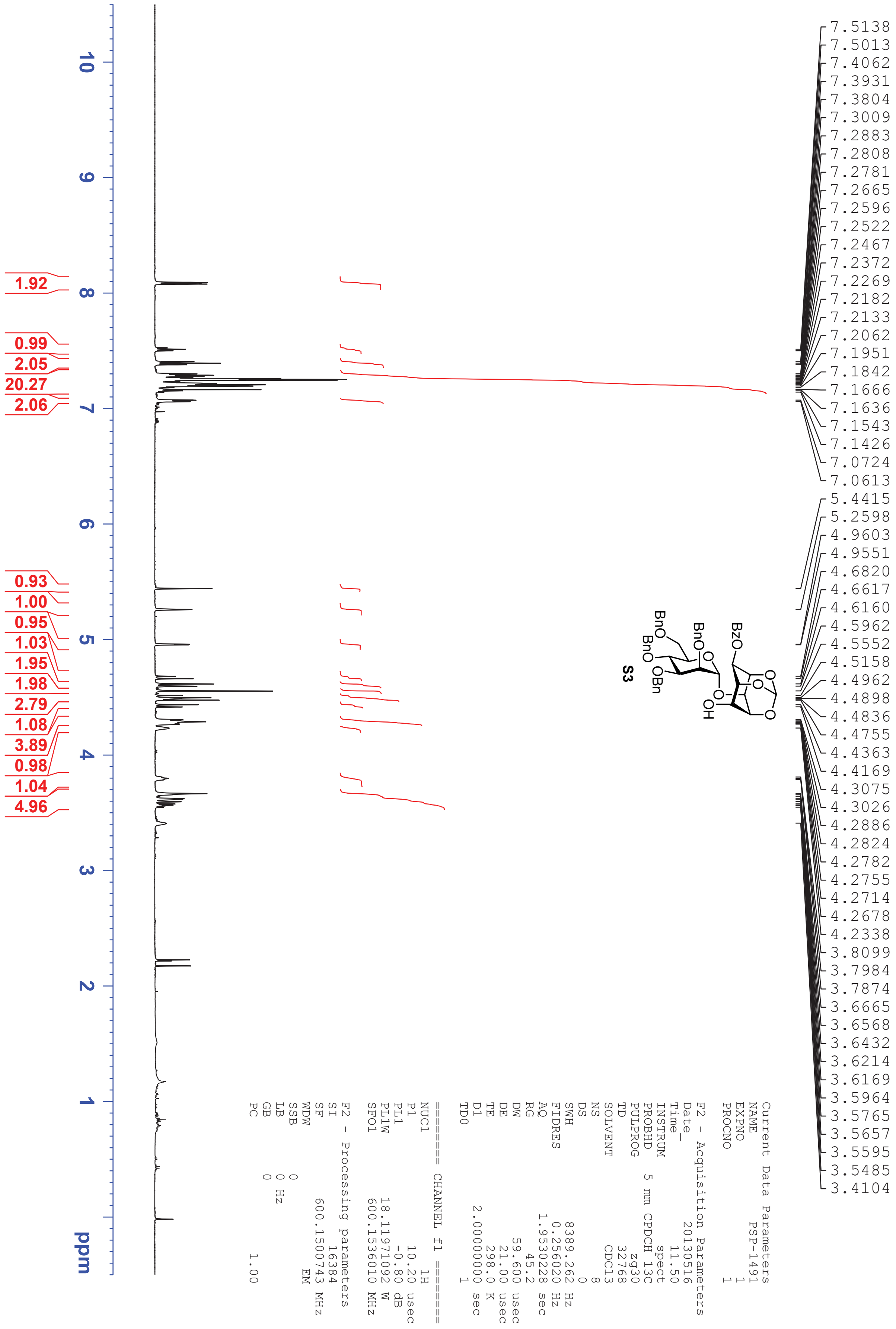

Supplementary Figure 37. ^{1}H NMR spectrum of compound S3.

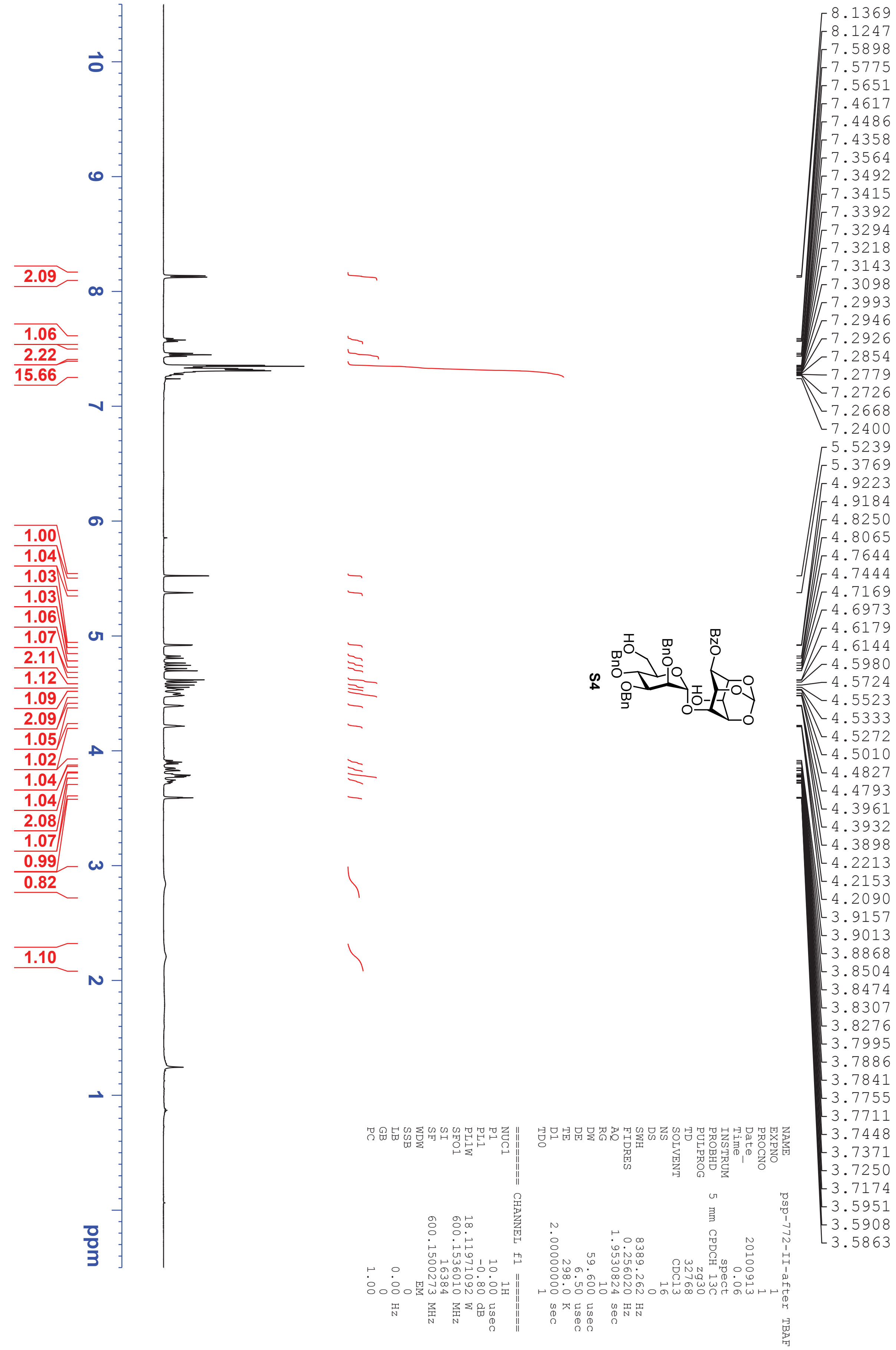

Supplementary Figure 38. ^{1}H NMR spectrum of compound S4.

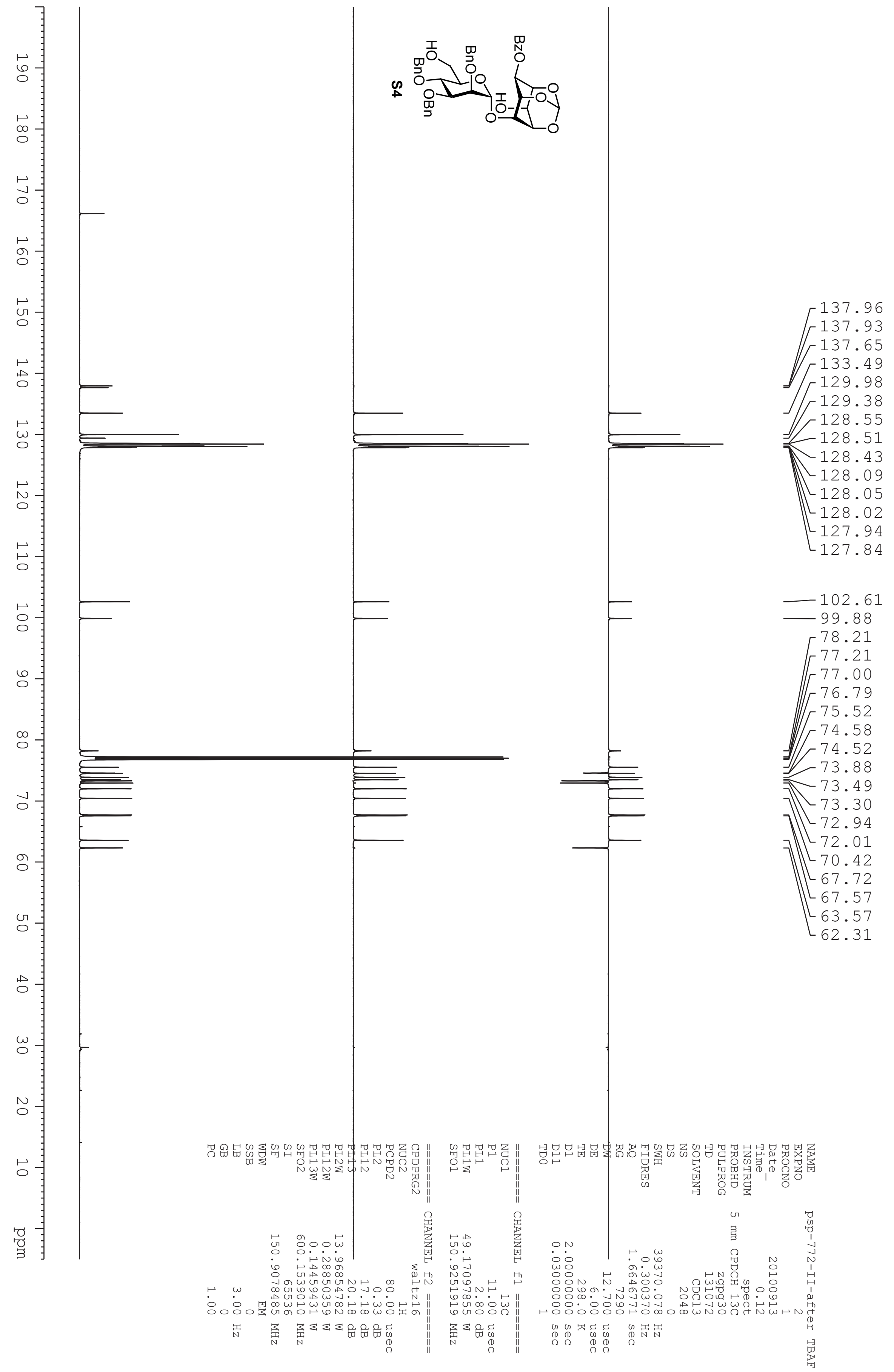

Supplementary Figure 39. ^{13}C and DEPT NMR spectra of compound S4.

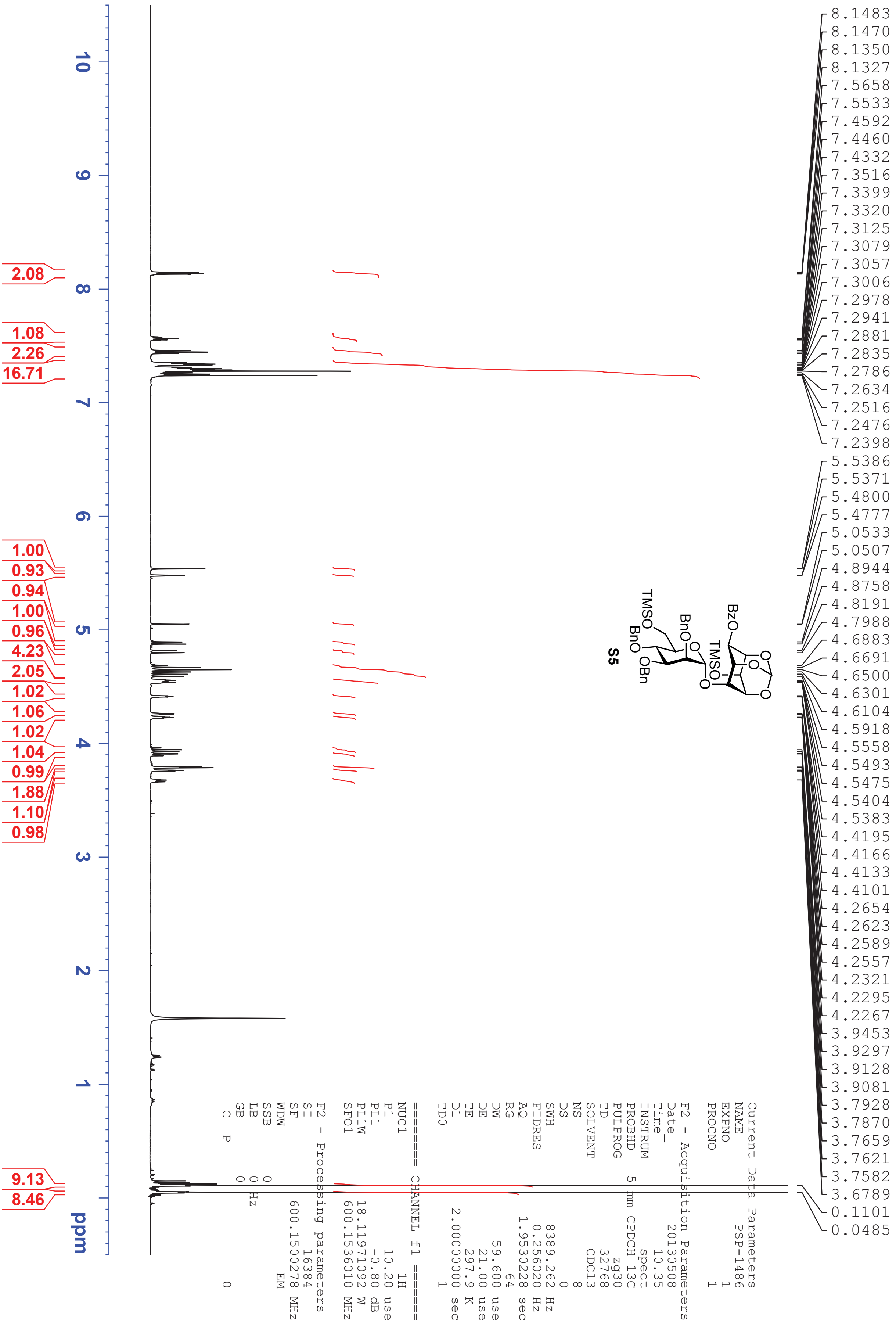

Supplementary Figure 40. ^{1}H NMR spectrum of compound S5.

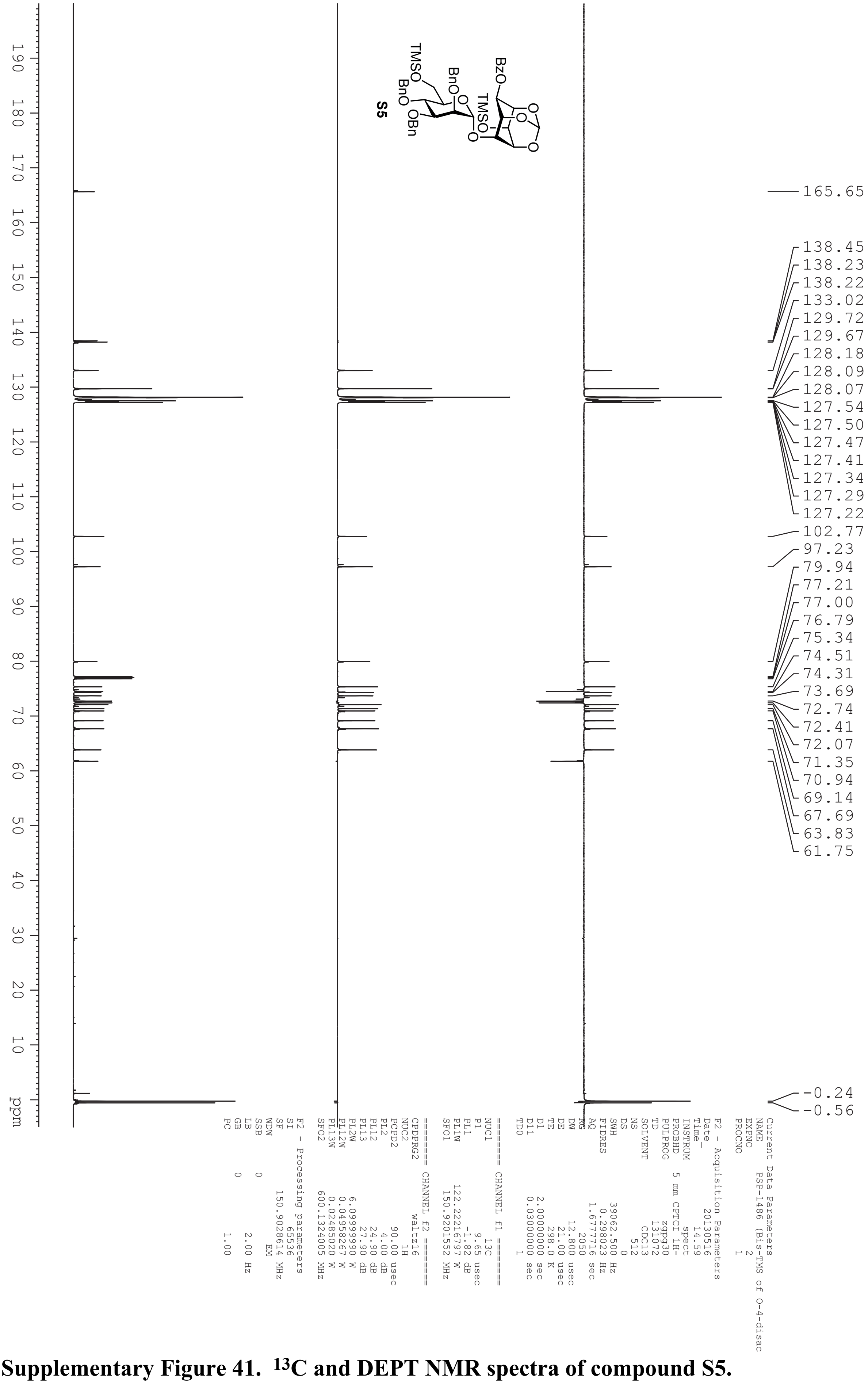

Supplementary Figure 41. ^{13}C and DEPT NMR spectra of compound S5.

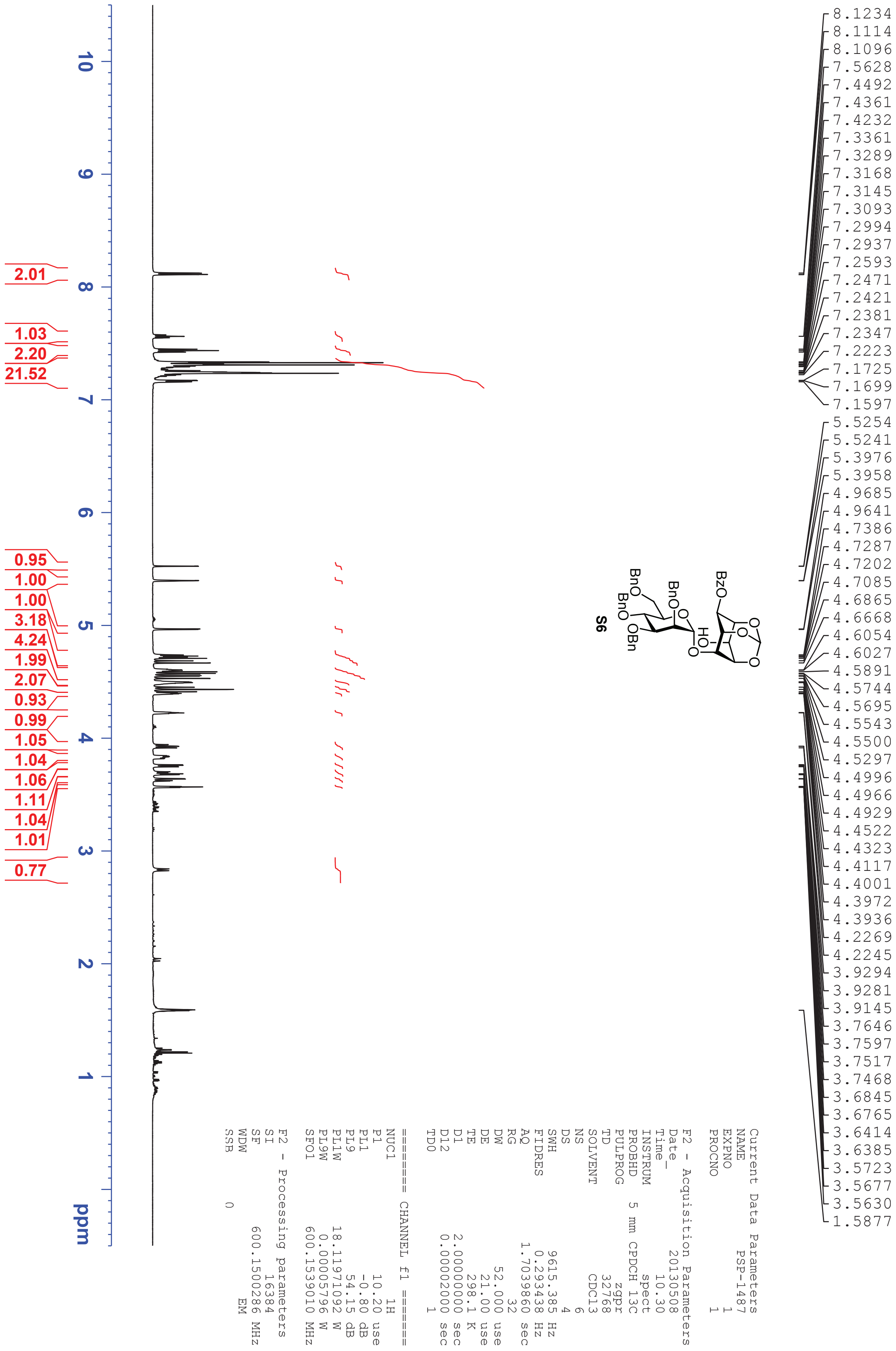

Supplementary Figure 42. ^{1}H NMR spectrum of compound S6.

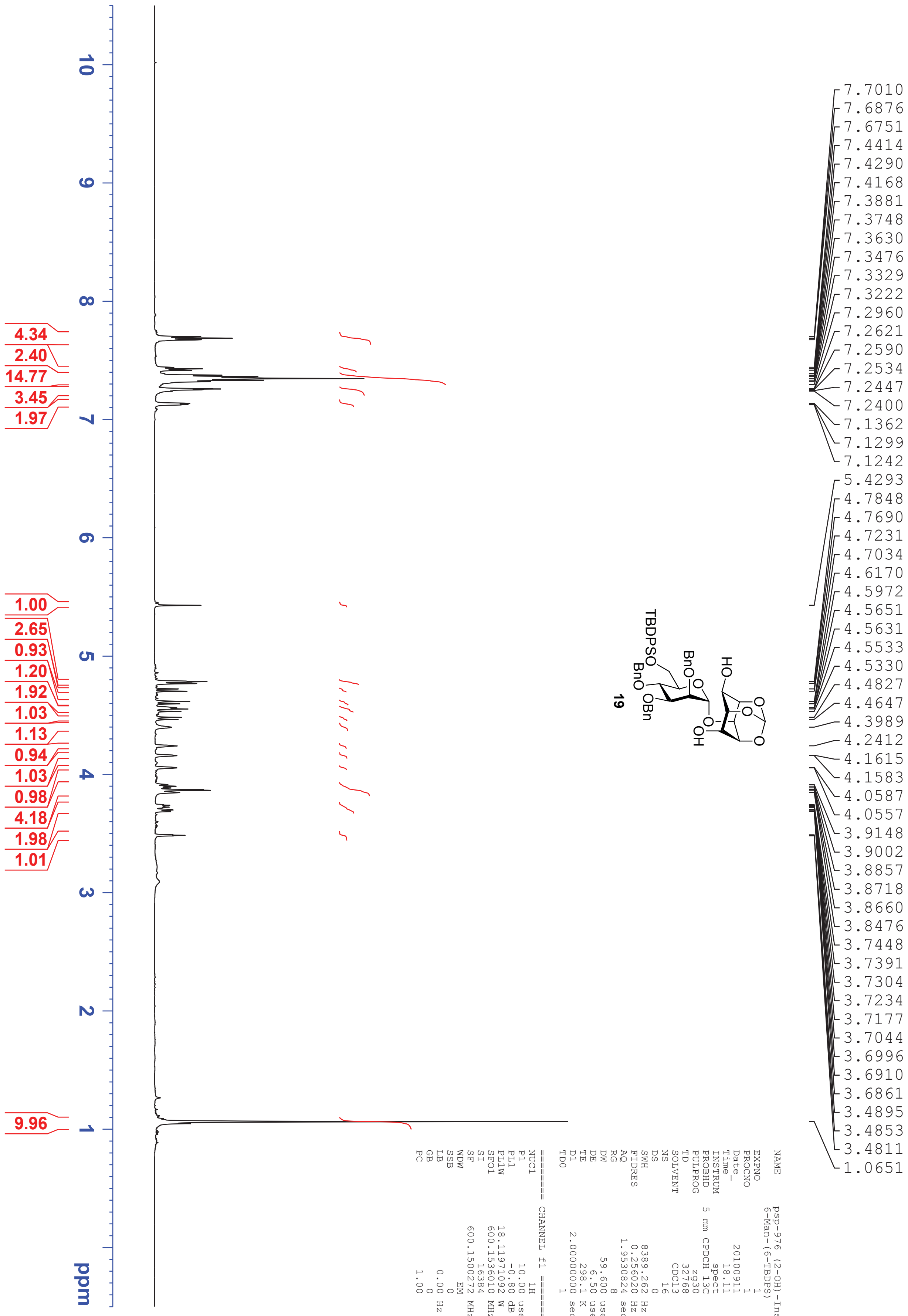

Supplementary Figure 43. **^{1}H NMR spectrum of compound 19.**

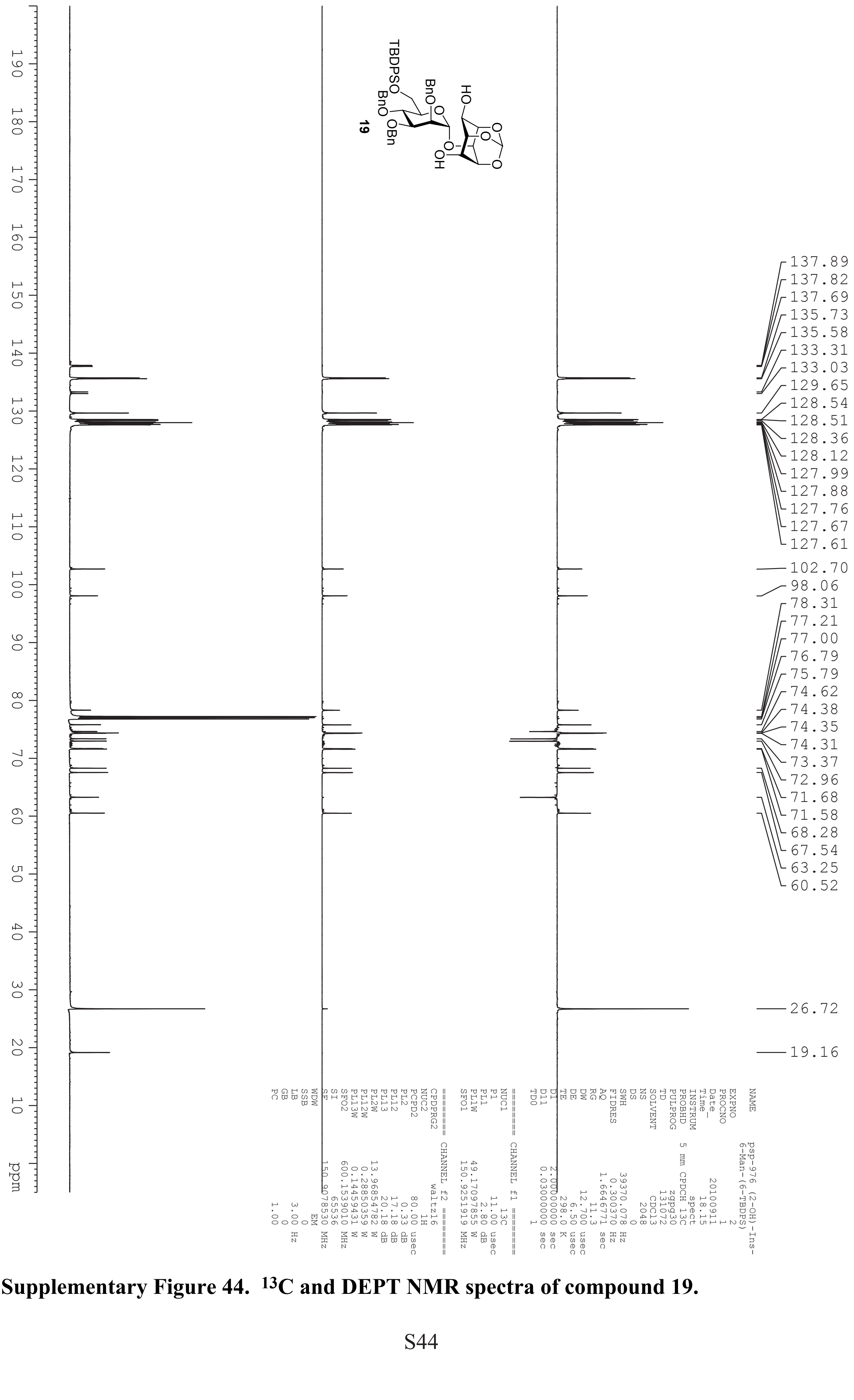

Supplementary Figure 44. ^{13}C and DEPT NMR spectra of compound 19.

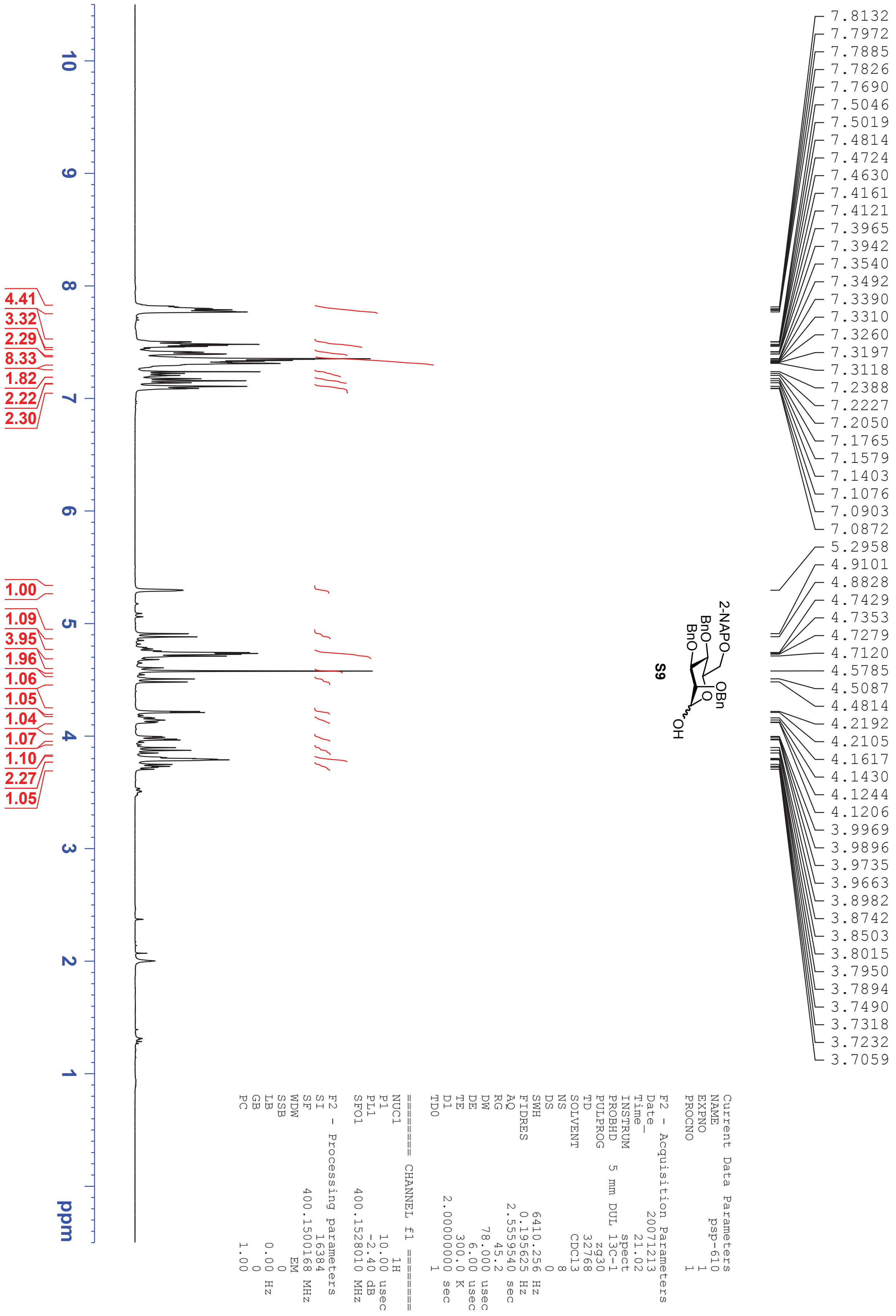

Supplementary Figure 45. ^{1}H NMR spectrum of compound S9.

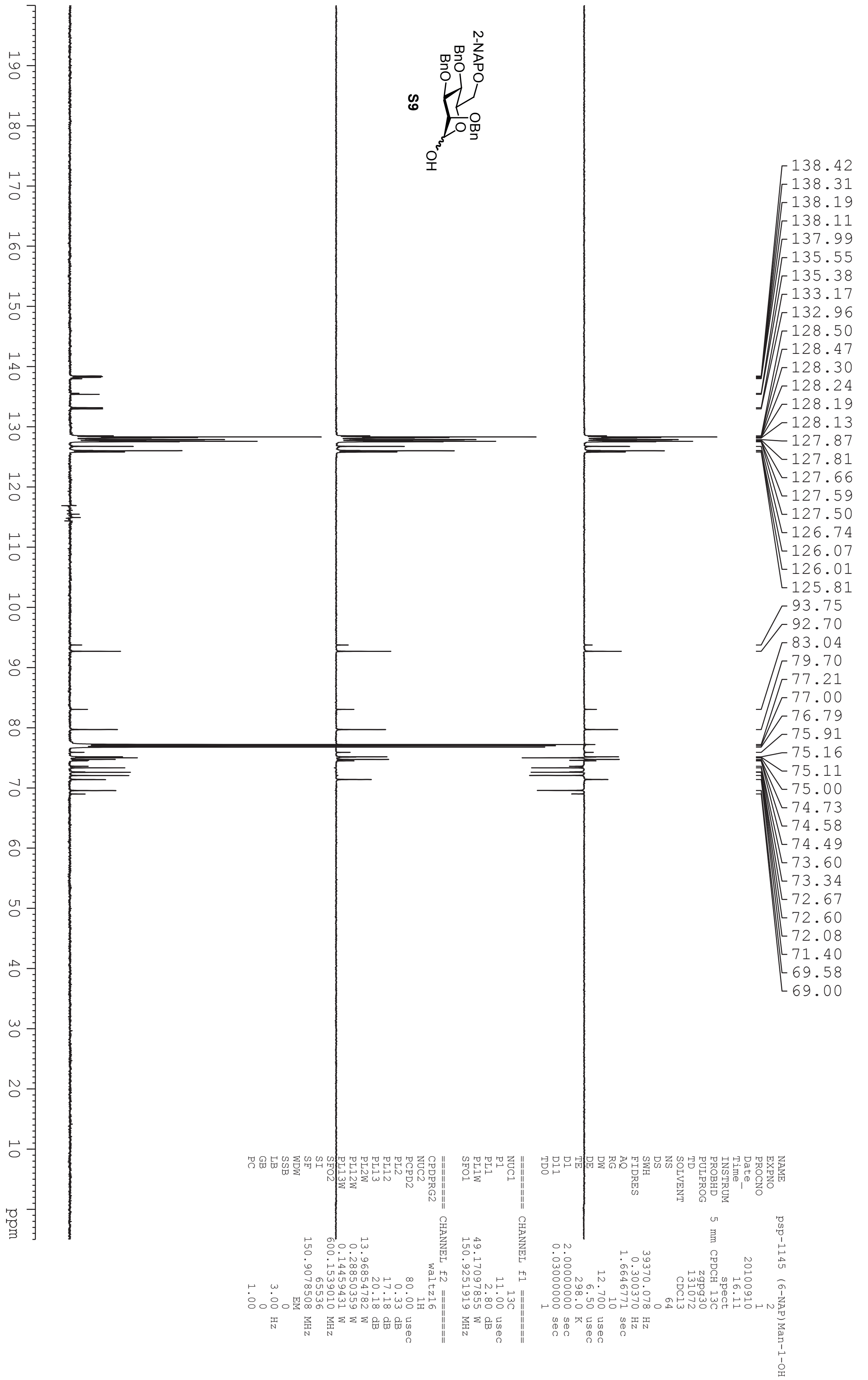

Supplementary Figure 46. ^{13}C and DEPT NMR spectra of compound S9.

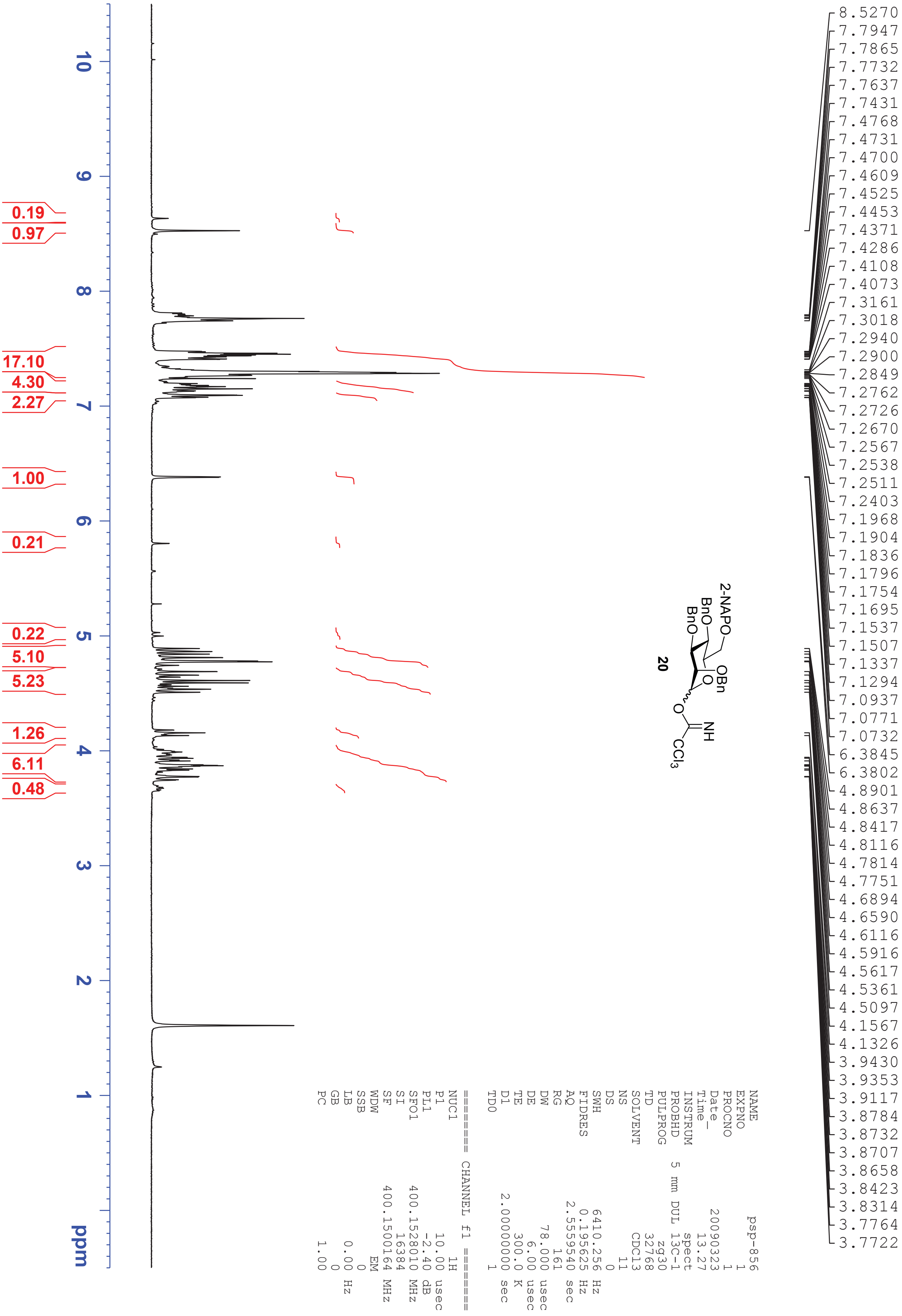

Supplementary Figure 47. ¹H NMR spectrum of compound 20.

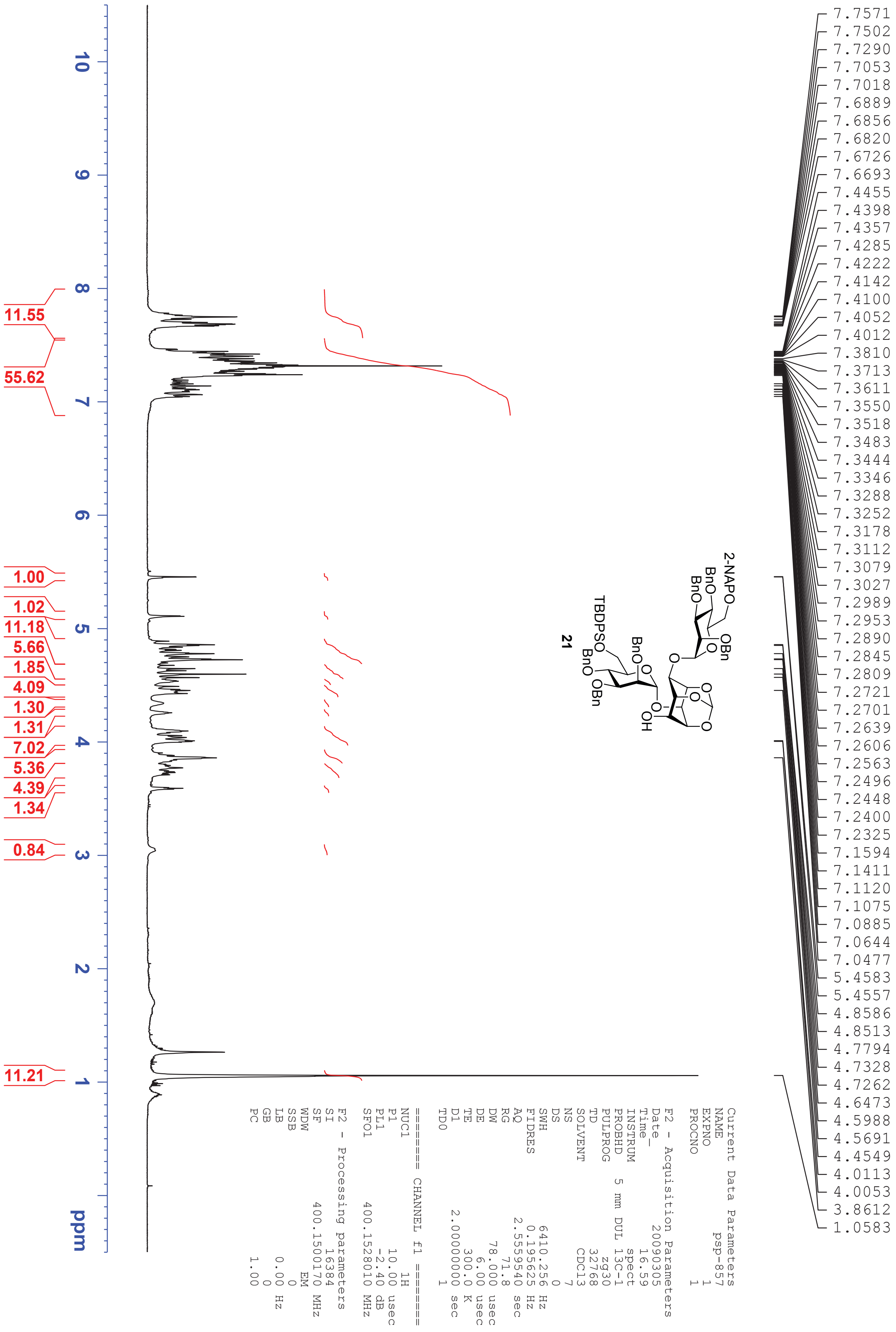

Supplementary Figure 48. ¹H NMR spectrum of compound 21.

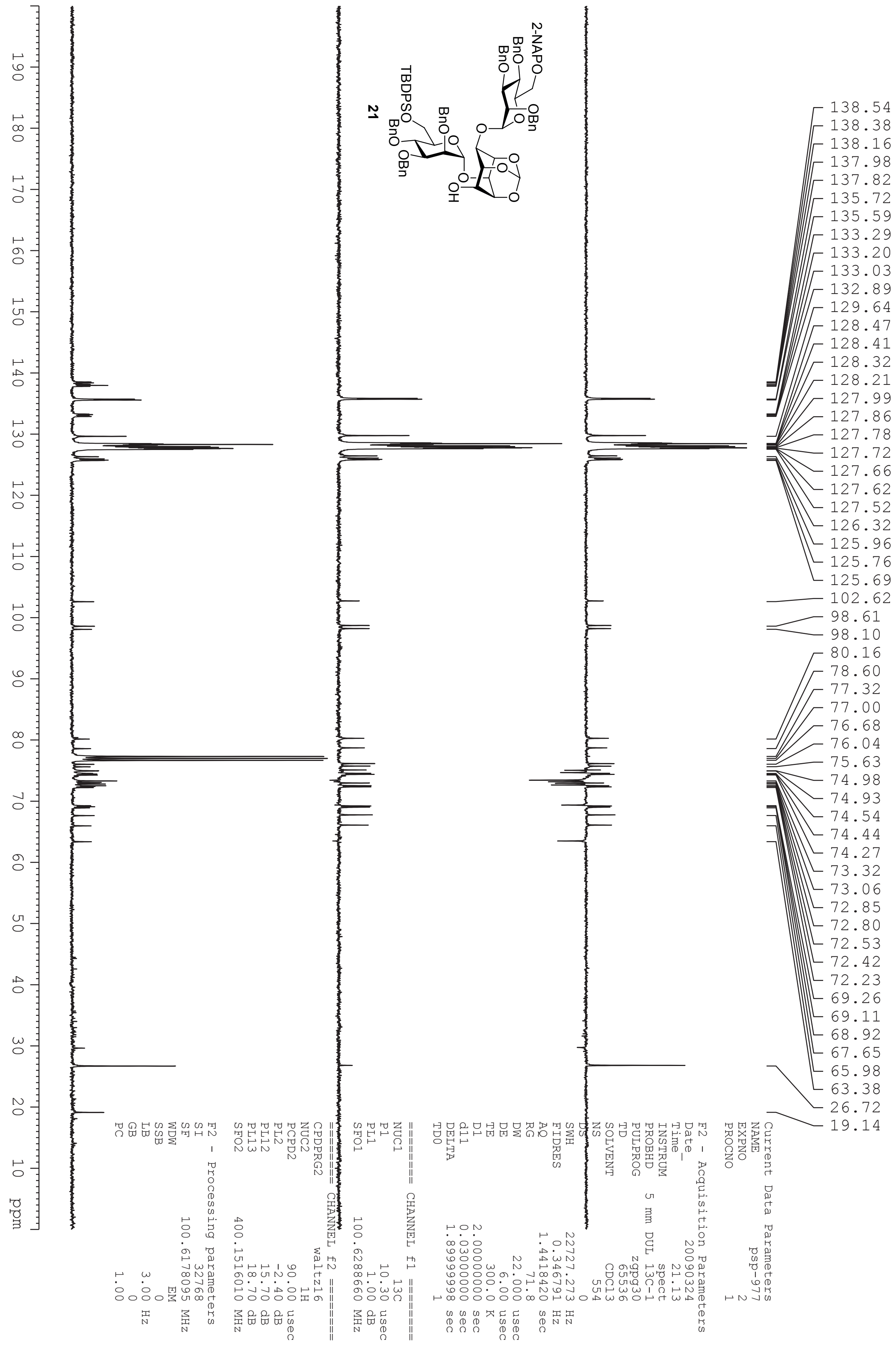

Supplementary Figure 49. ^{13}C and DEPT NMR spectra of compound 21.

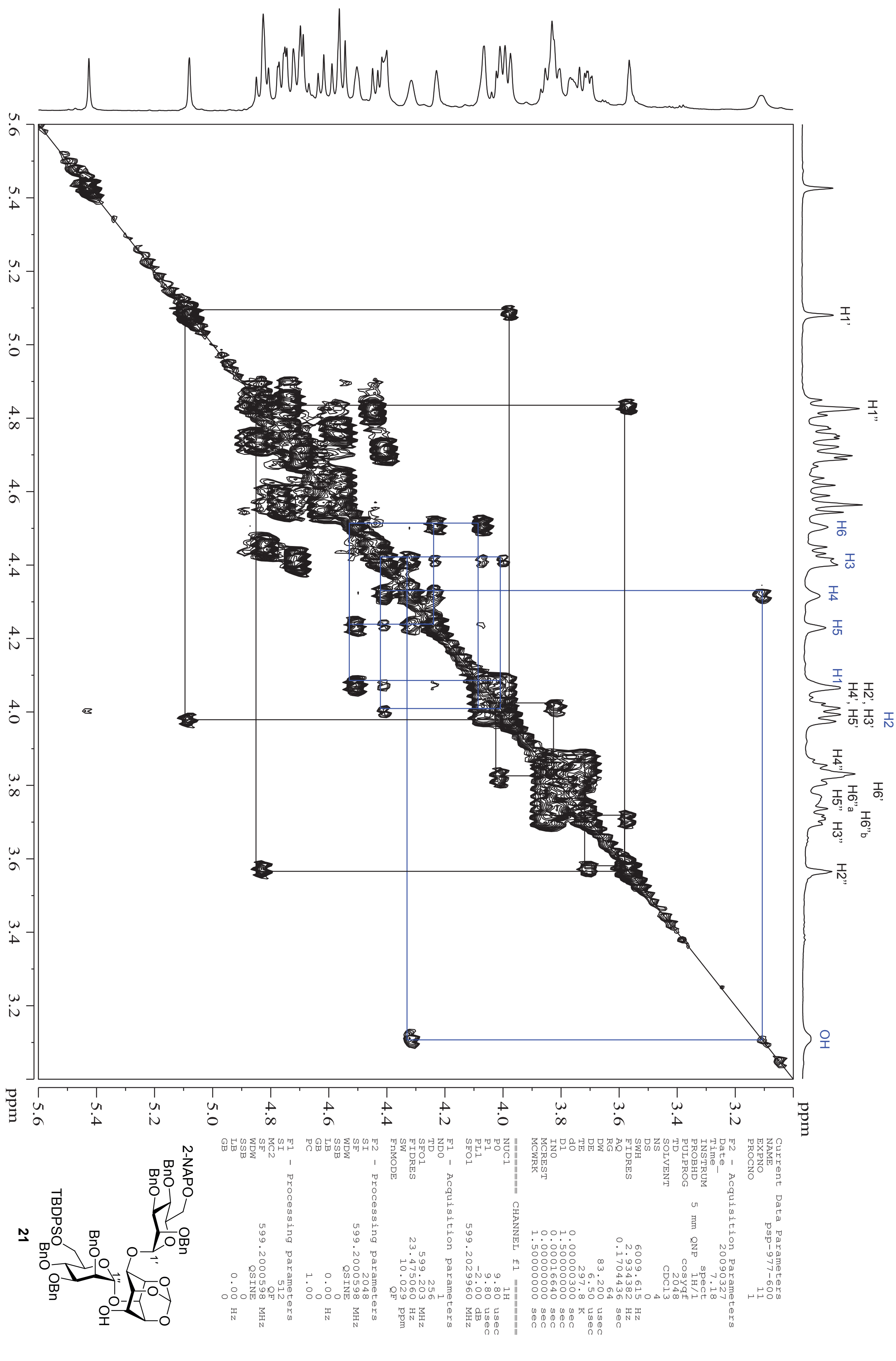

Supplementary Figure 50. COSY NMR spectrum of compound 21.

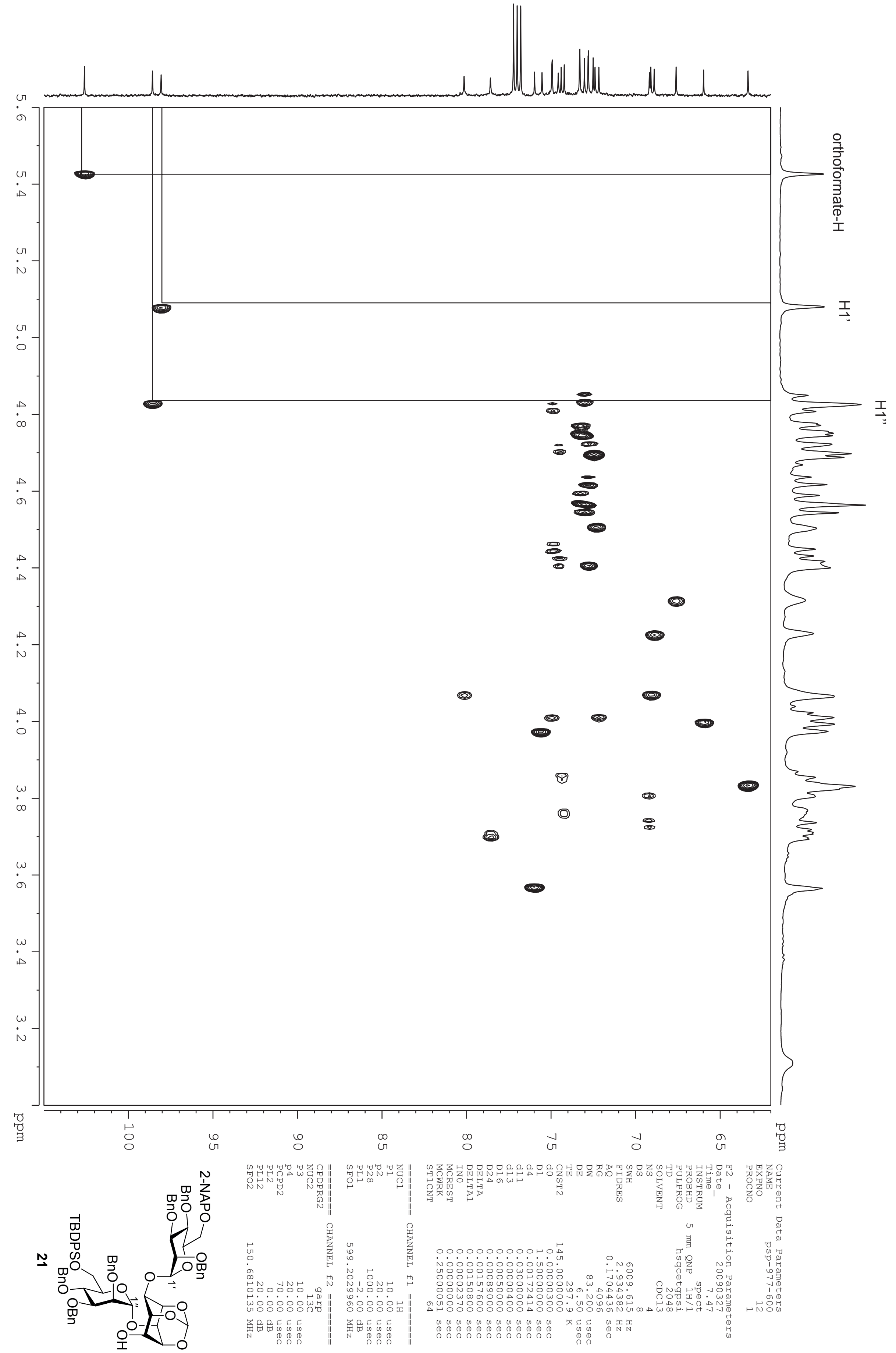

Supplementary Figure 51. HMQC NMR spectrum of compound 21.

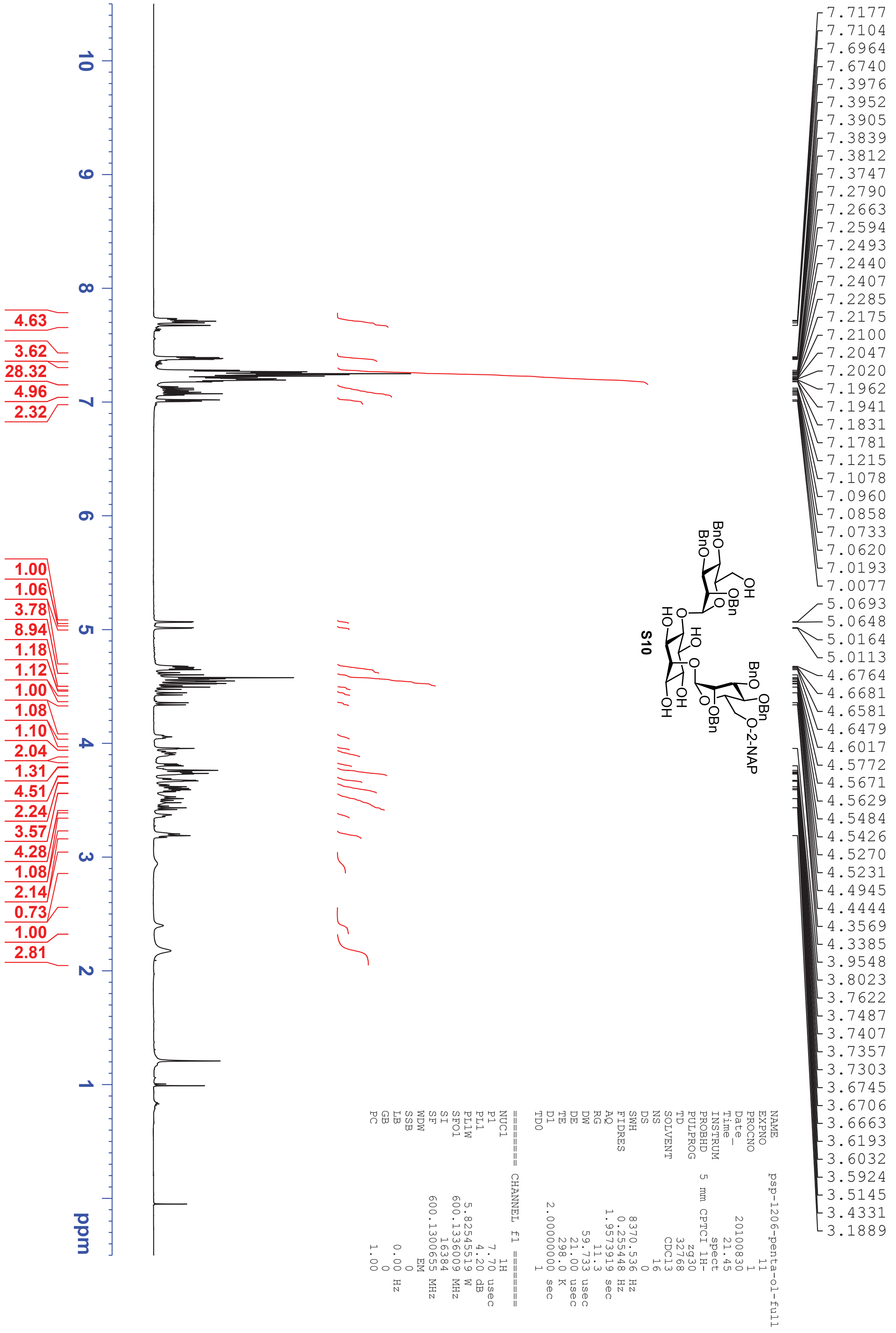

Supplementary Figure 52. ^{1}**H NMR spectrum of compound S10.**

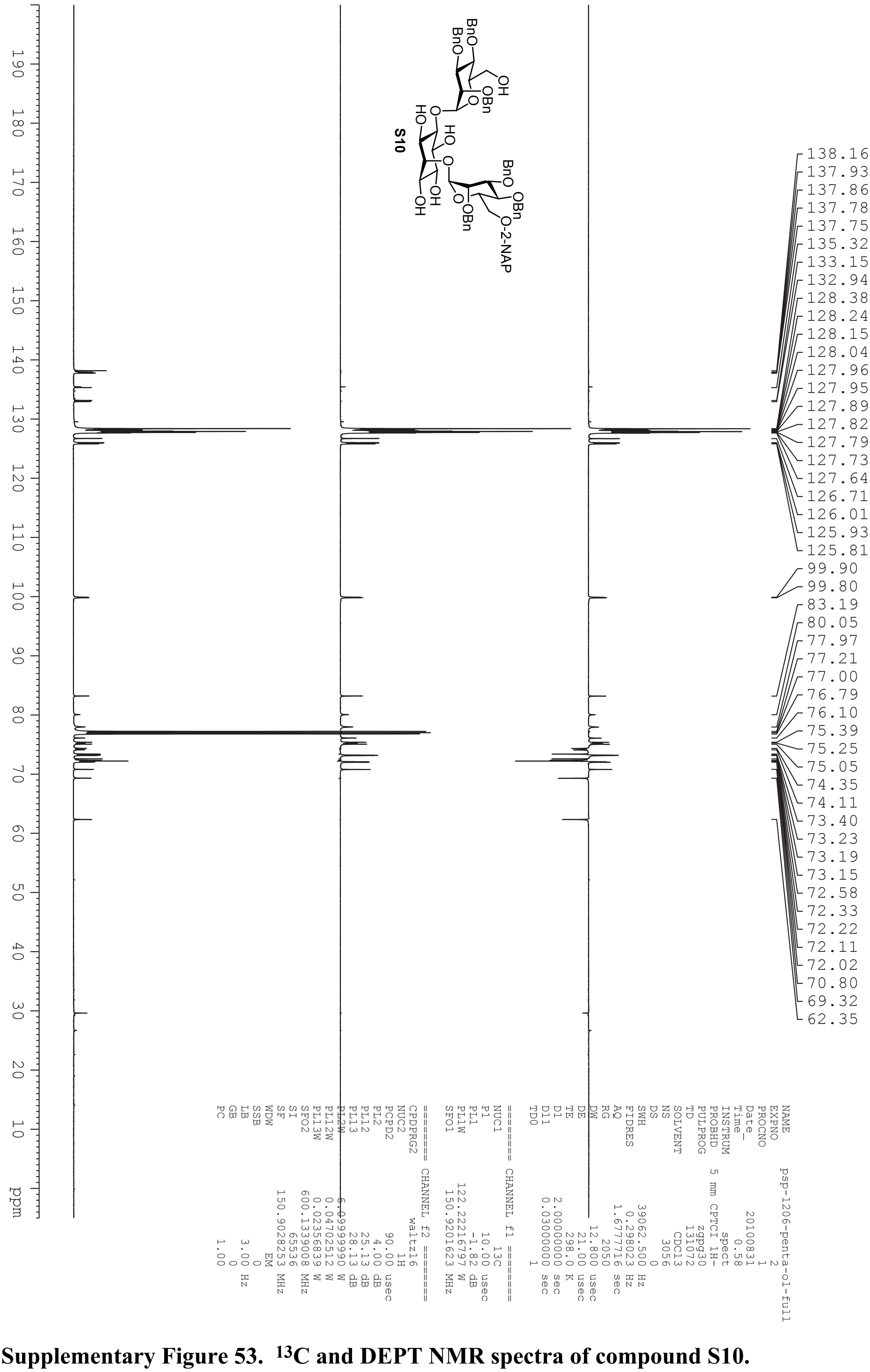

Supplementary Figure 53. ^{13}C and DEPT NMR spectra of compound S10.

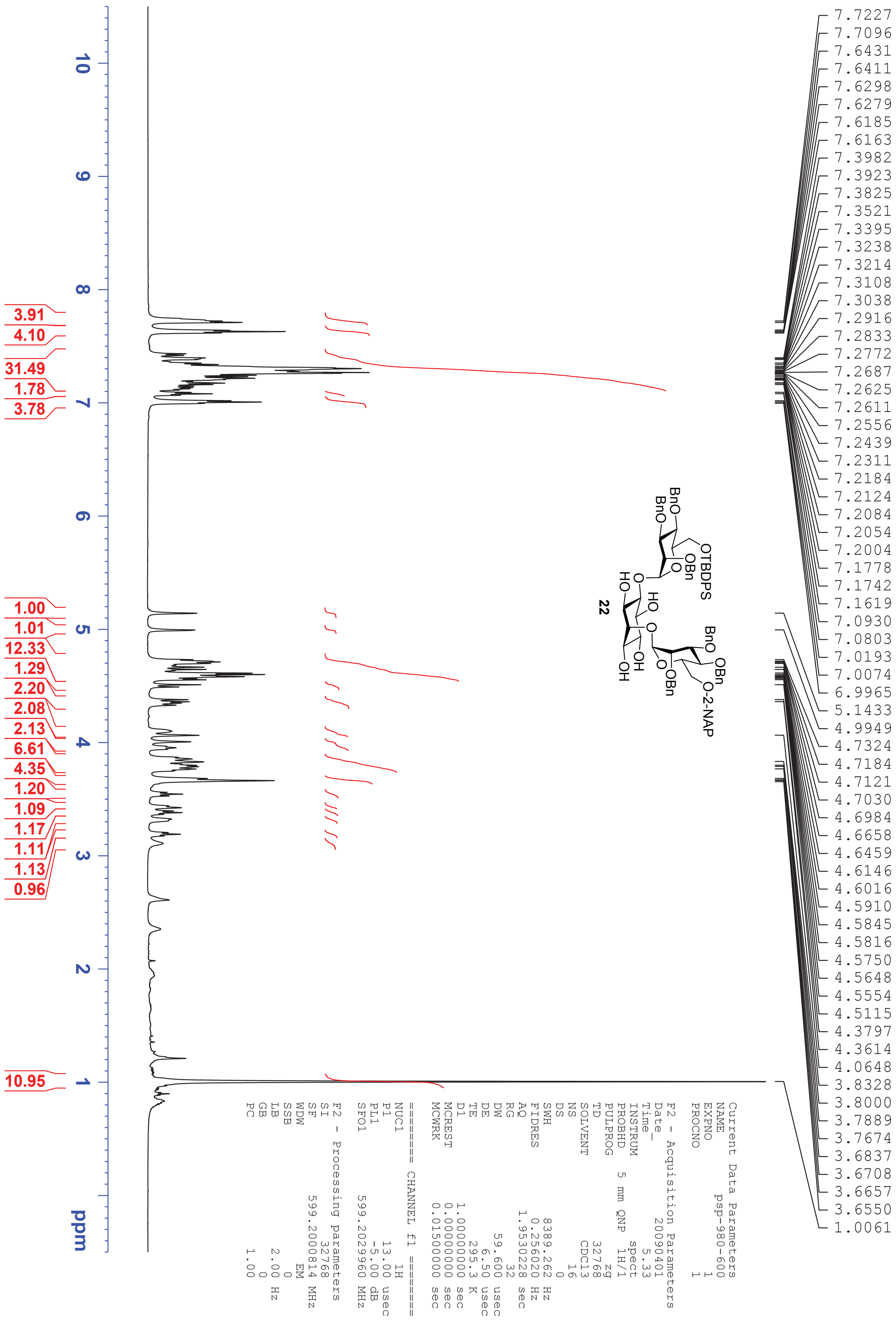

Supplementary Figure 54. ^{1}H NMR spectrum of compound 22.

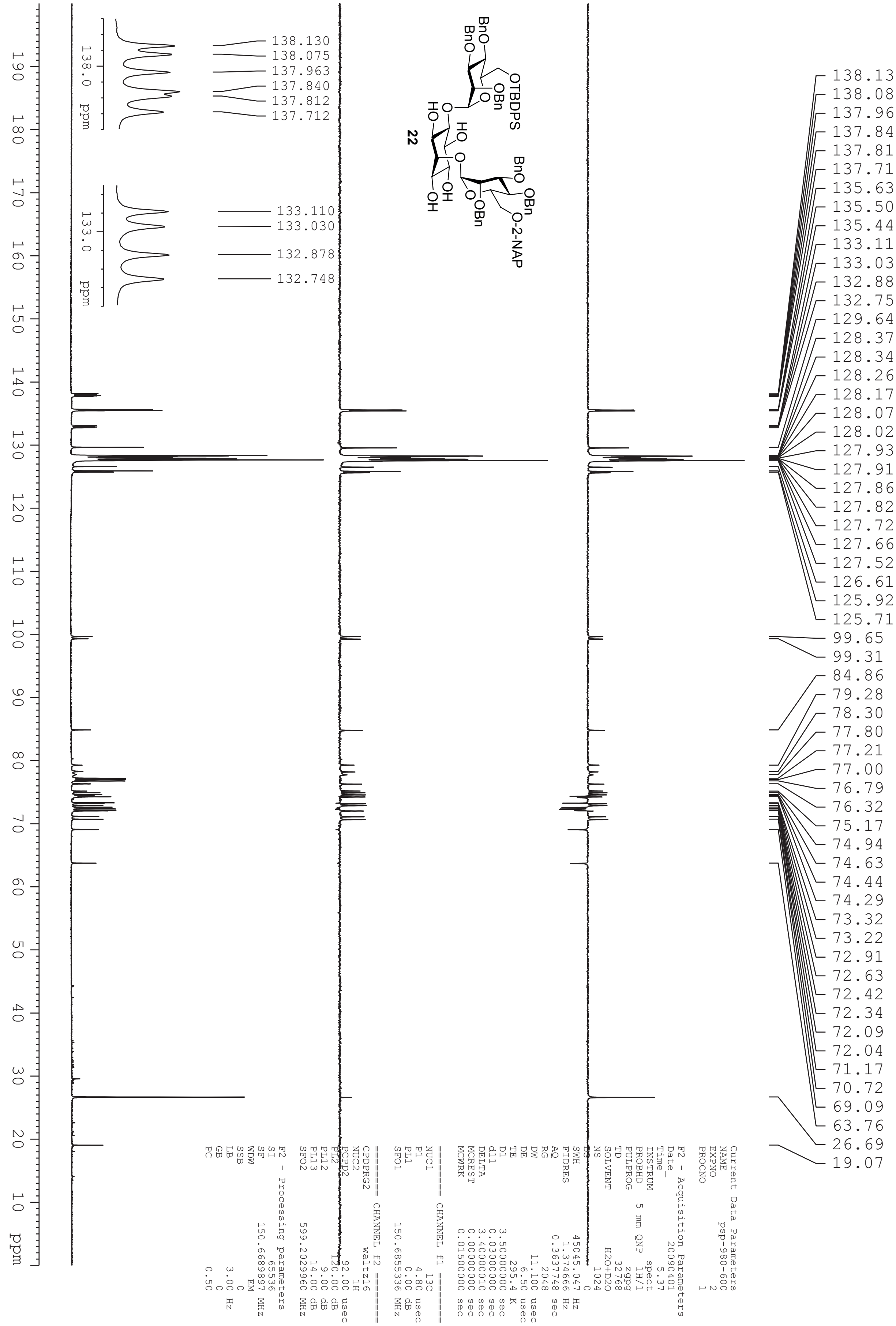

Supplementary Figure 55. ^{13}C and DEPT NMR spectra of compound 22.

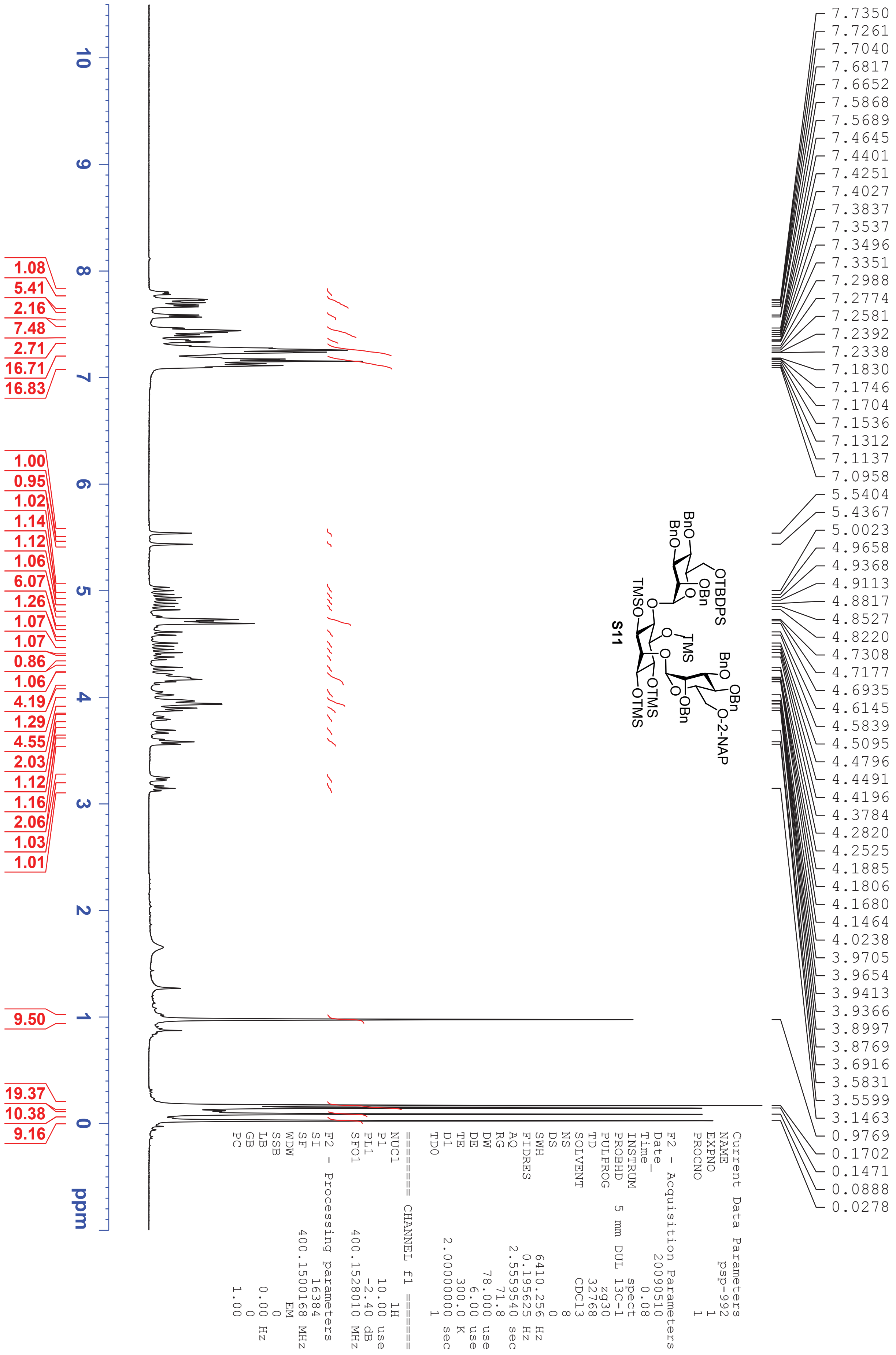

Supplementary Figure 56. **^{1}H NMR spectrum of compound S11.**

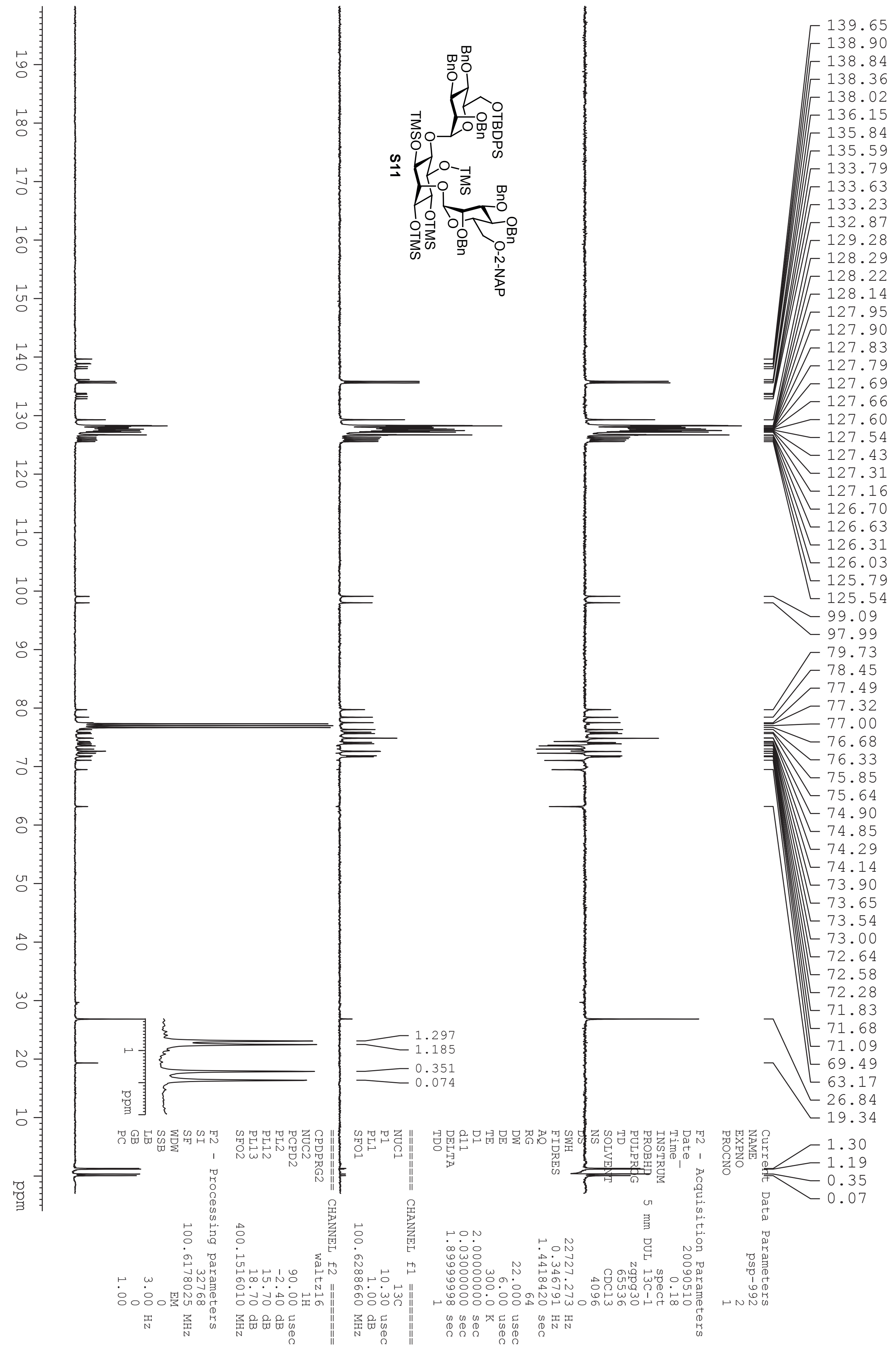

Supplementary Figure 57. ^{13}C and DEPT NMR spectra of compound S11.

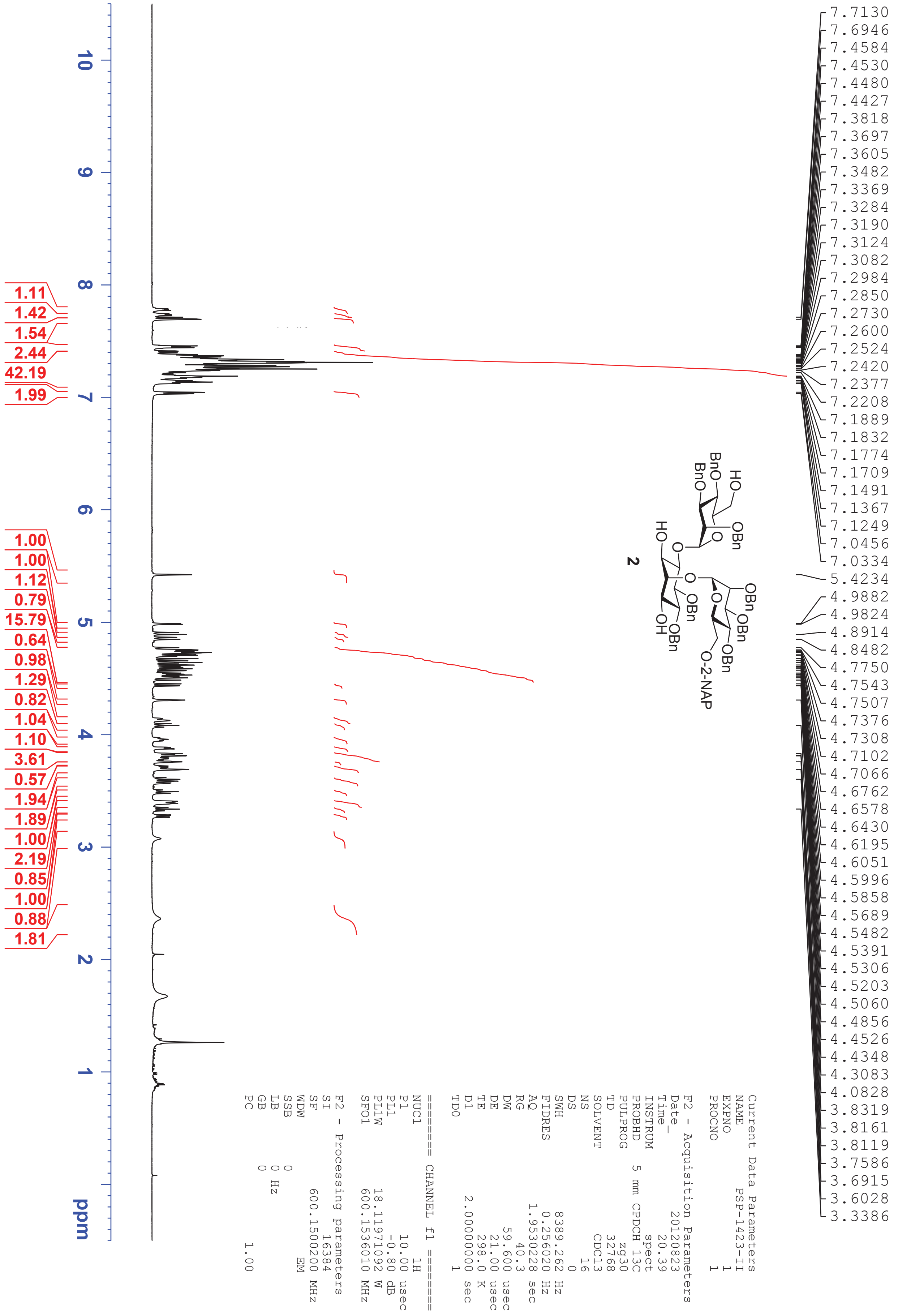

Supplementary Figure 58. ^{1}**H NMR spectrum of compound 2.**

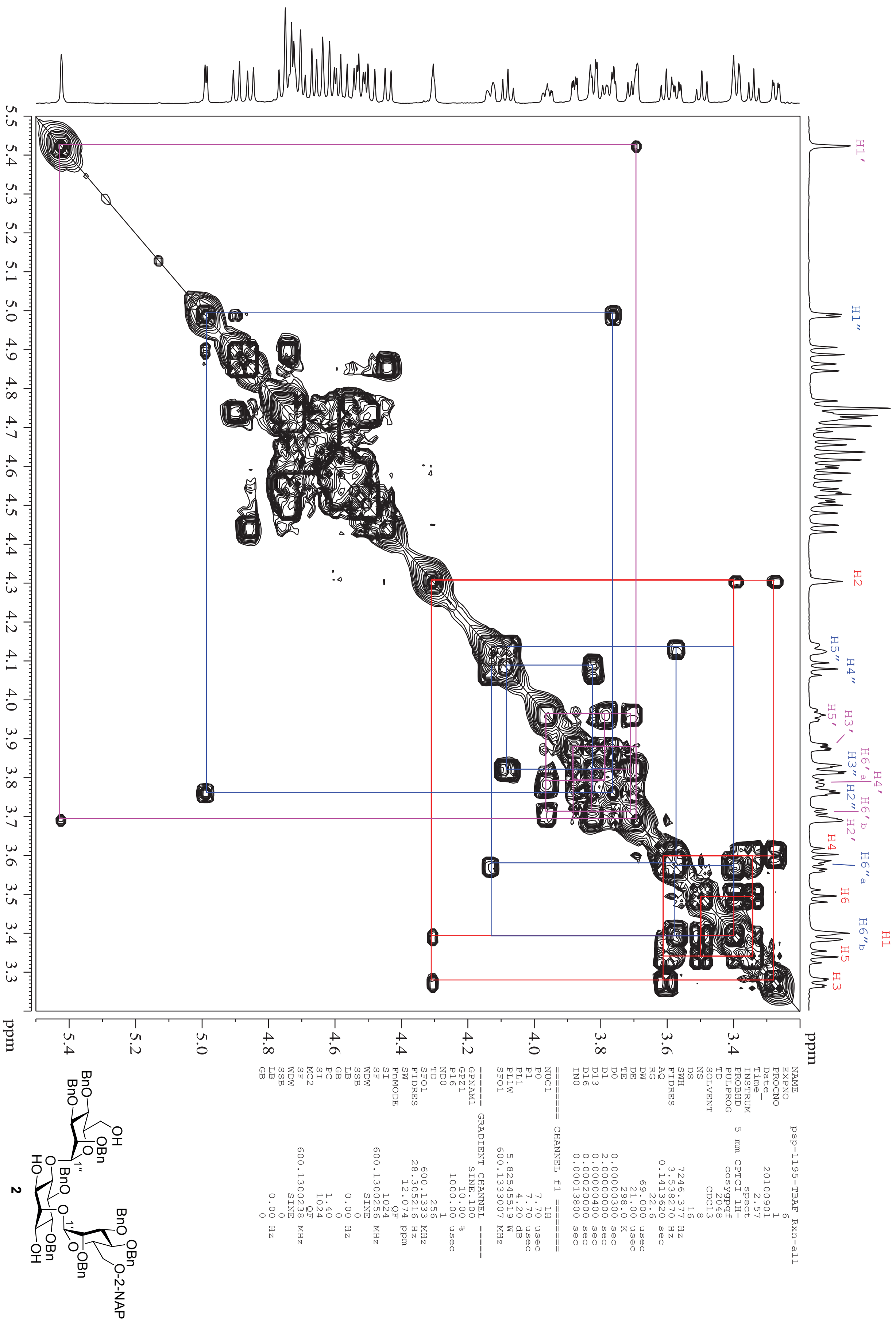

Supplementary Figure 59. COSY NMR spectrum of compound 2.

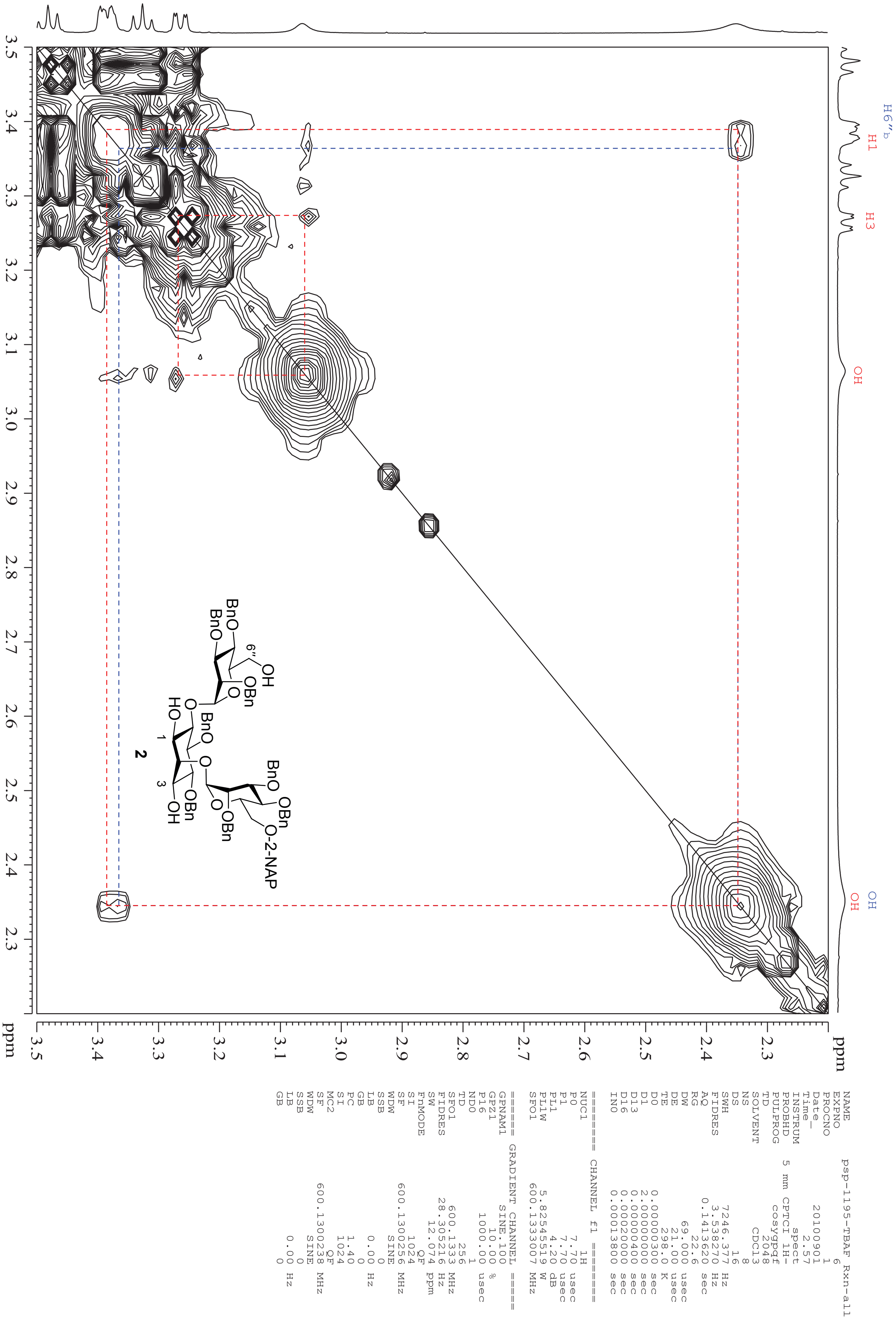

Supplementary Figure 60. COSY NMR spectrum of compound 2 identifying the location of the hydroxy groups.

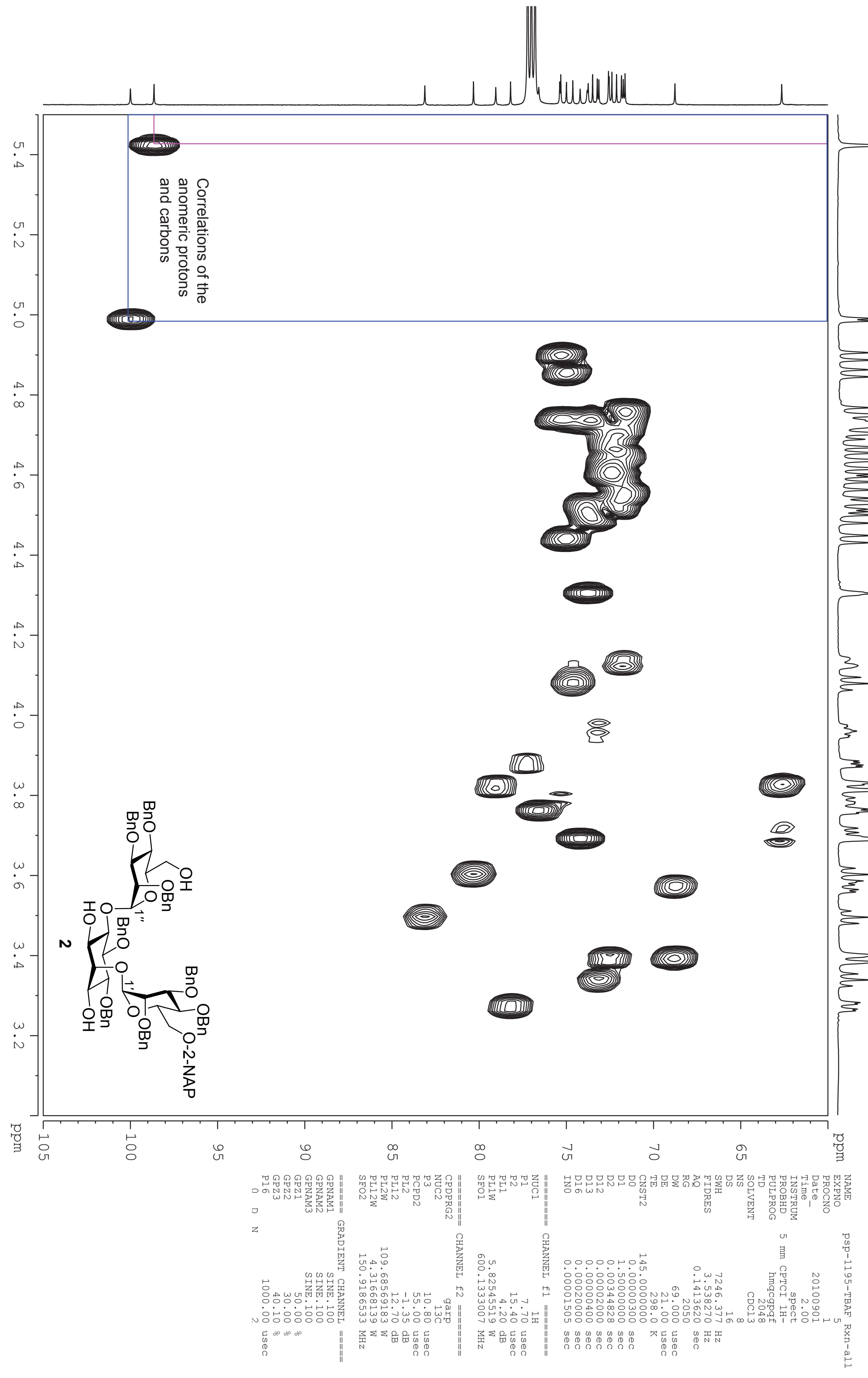

Supplementary Figure 61. HMQC NMR spectrum of compound 2.

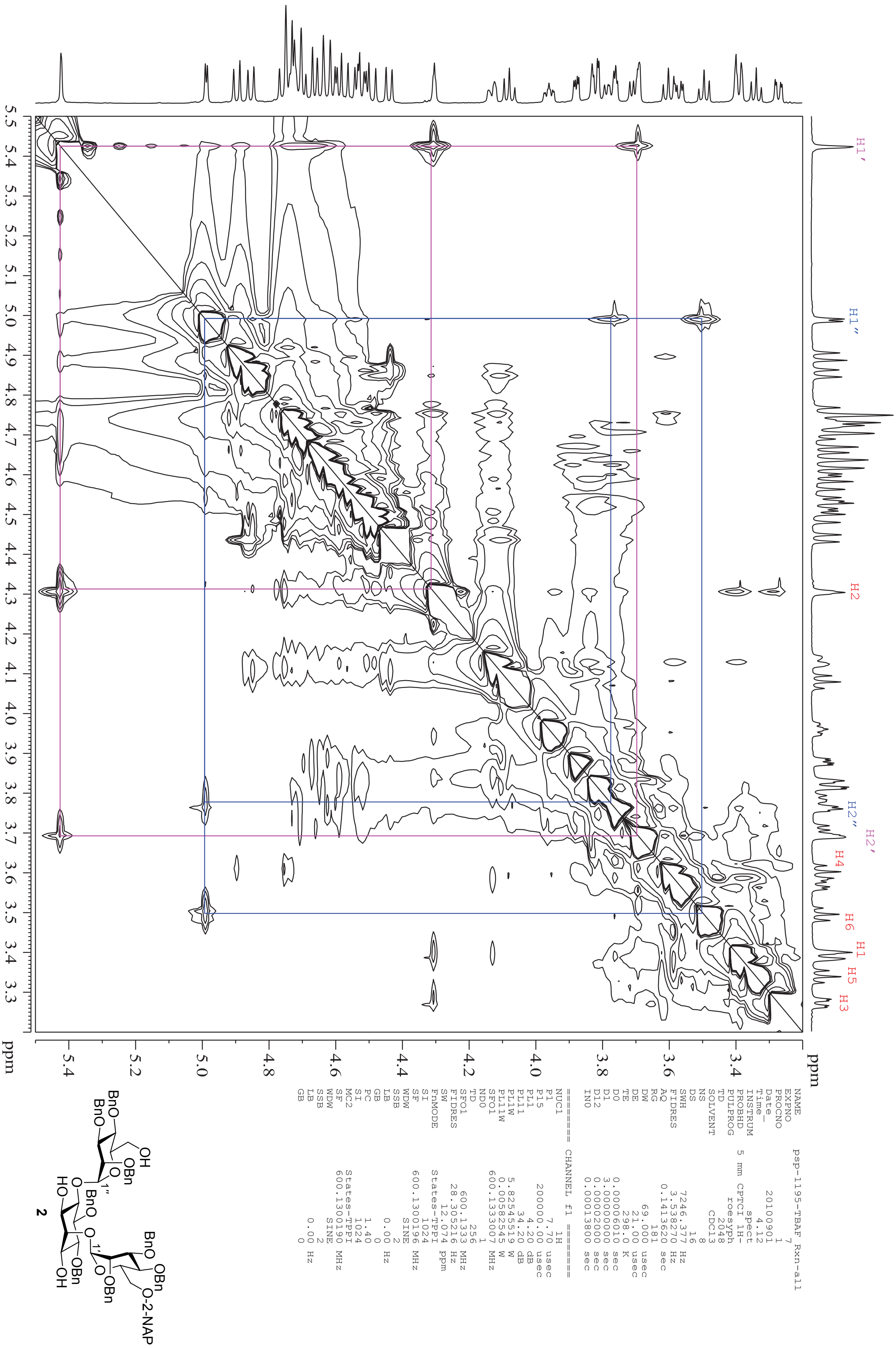

Supplementary Figure 62. ROESY NMR spectrum of compound 2.

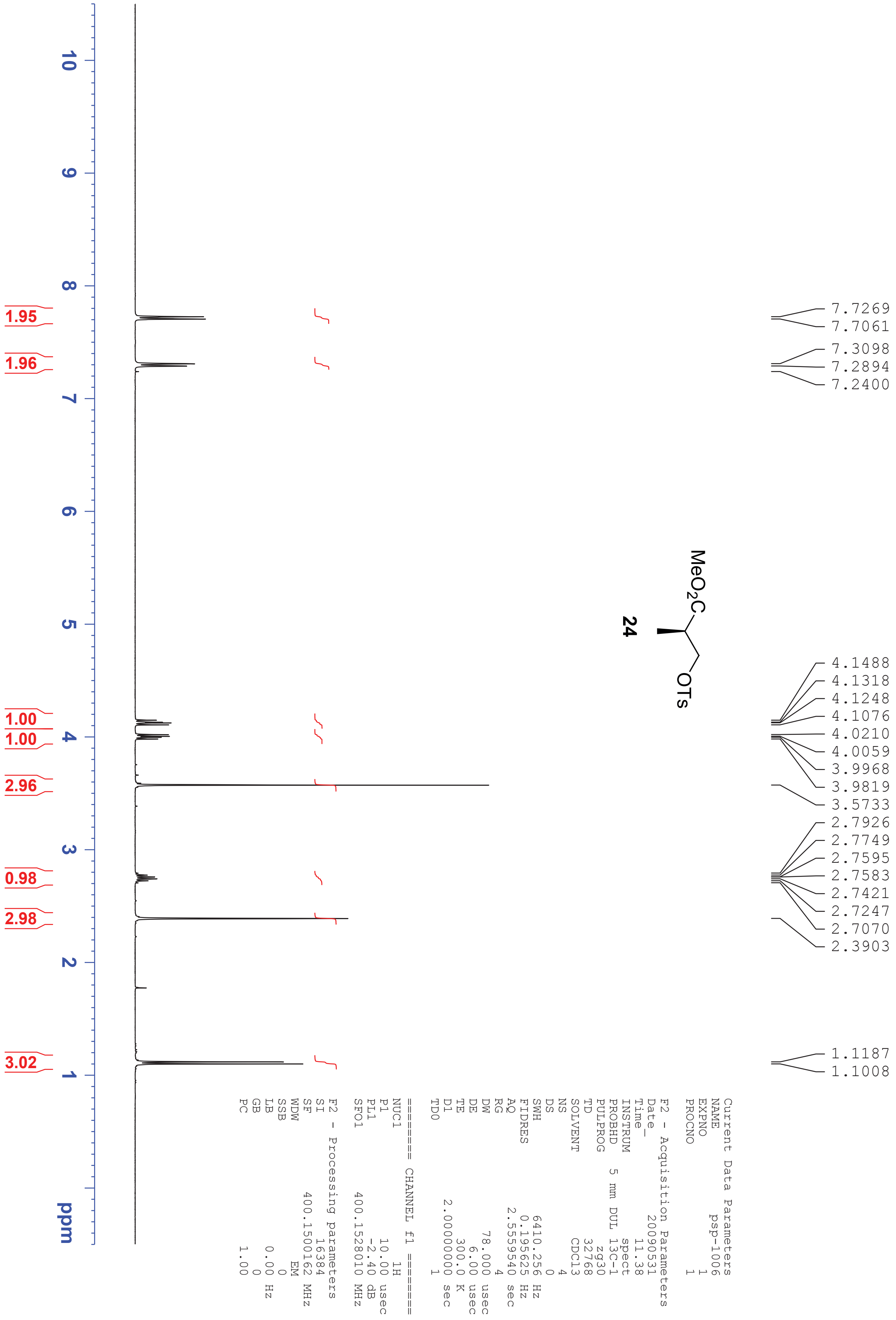

Supplementary Figure 63. **^{1}H NMR spectrum of compound 24.**

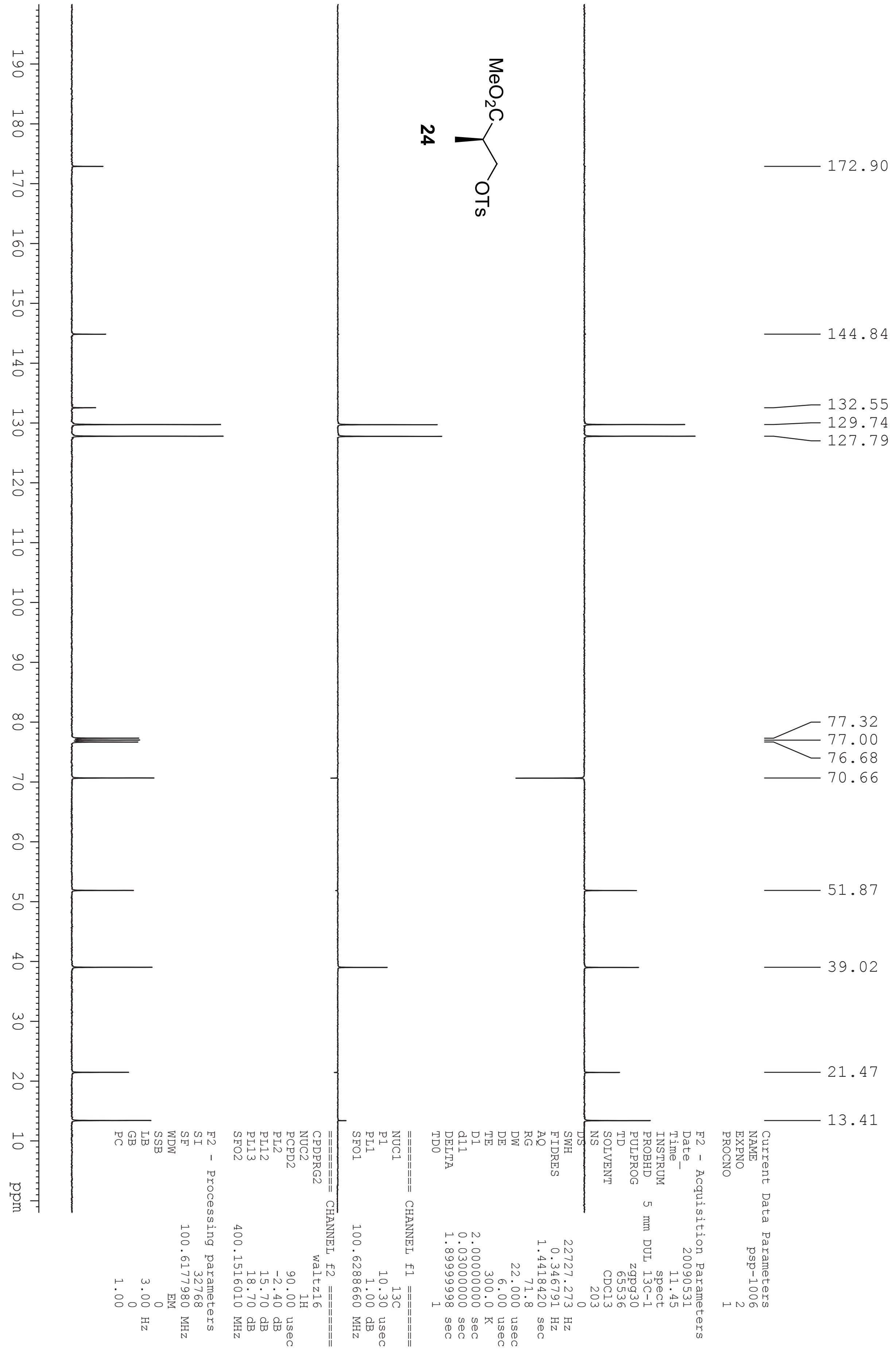

Supplementary Figure 64. ^{13}C and DEPT NMR spectra of compound 24.

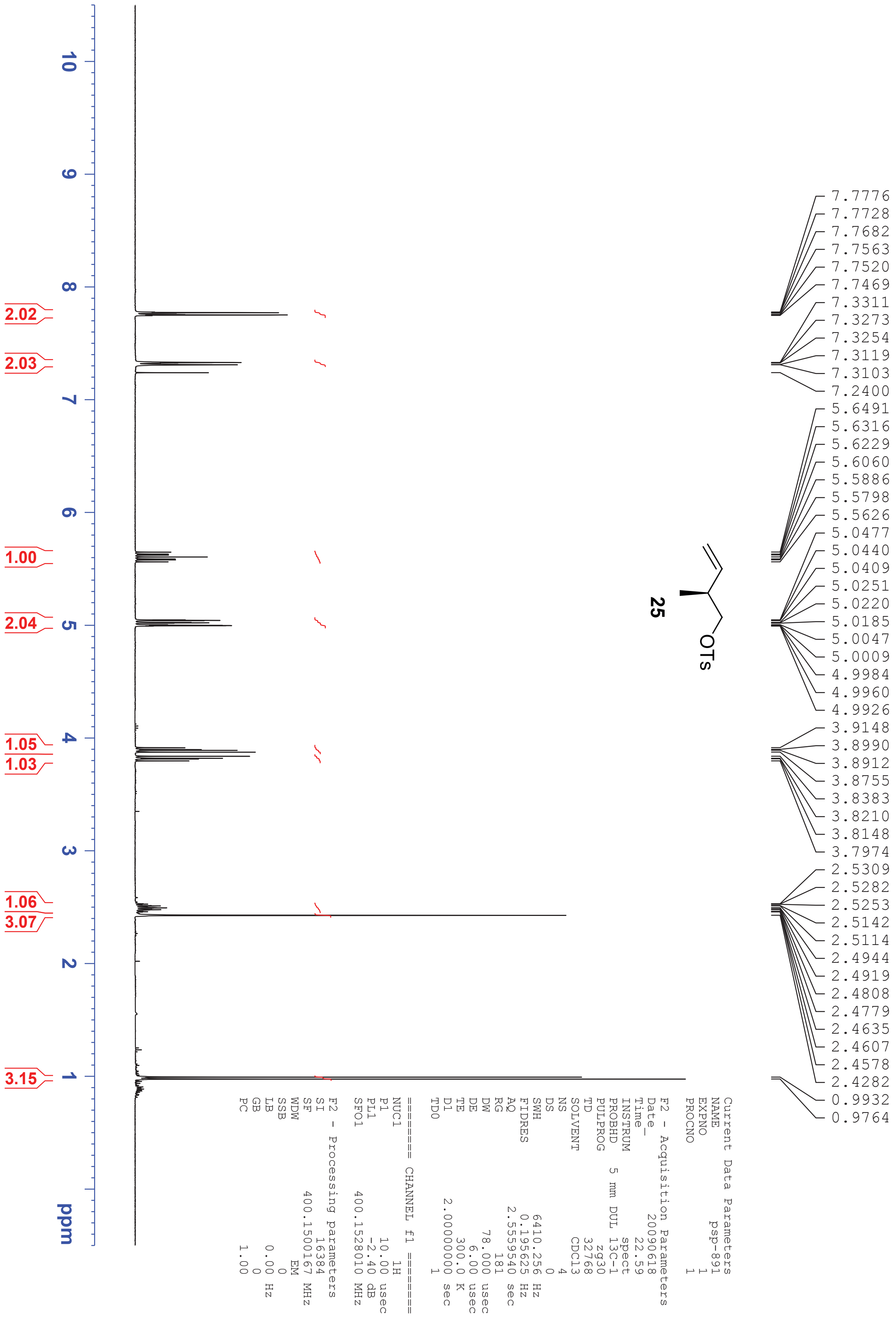

Supplementary Figure 65. ^{1}**H NMR spectrum of compound 25.**

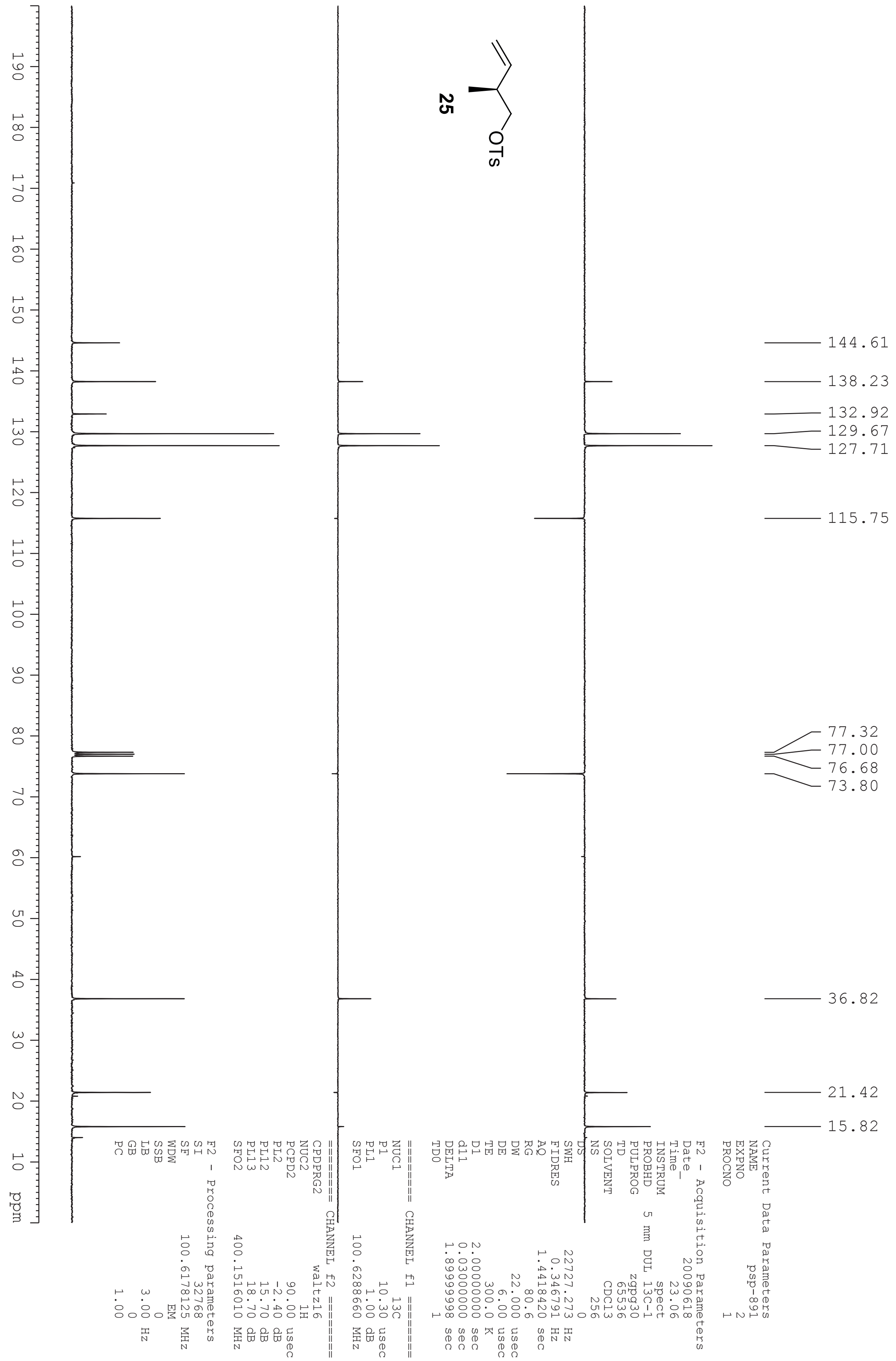

Supplementary Figure 66. ^{13}C and DEPT NMR spectra of compound 25.

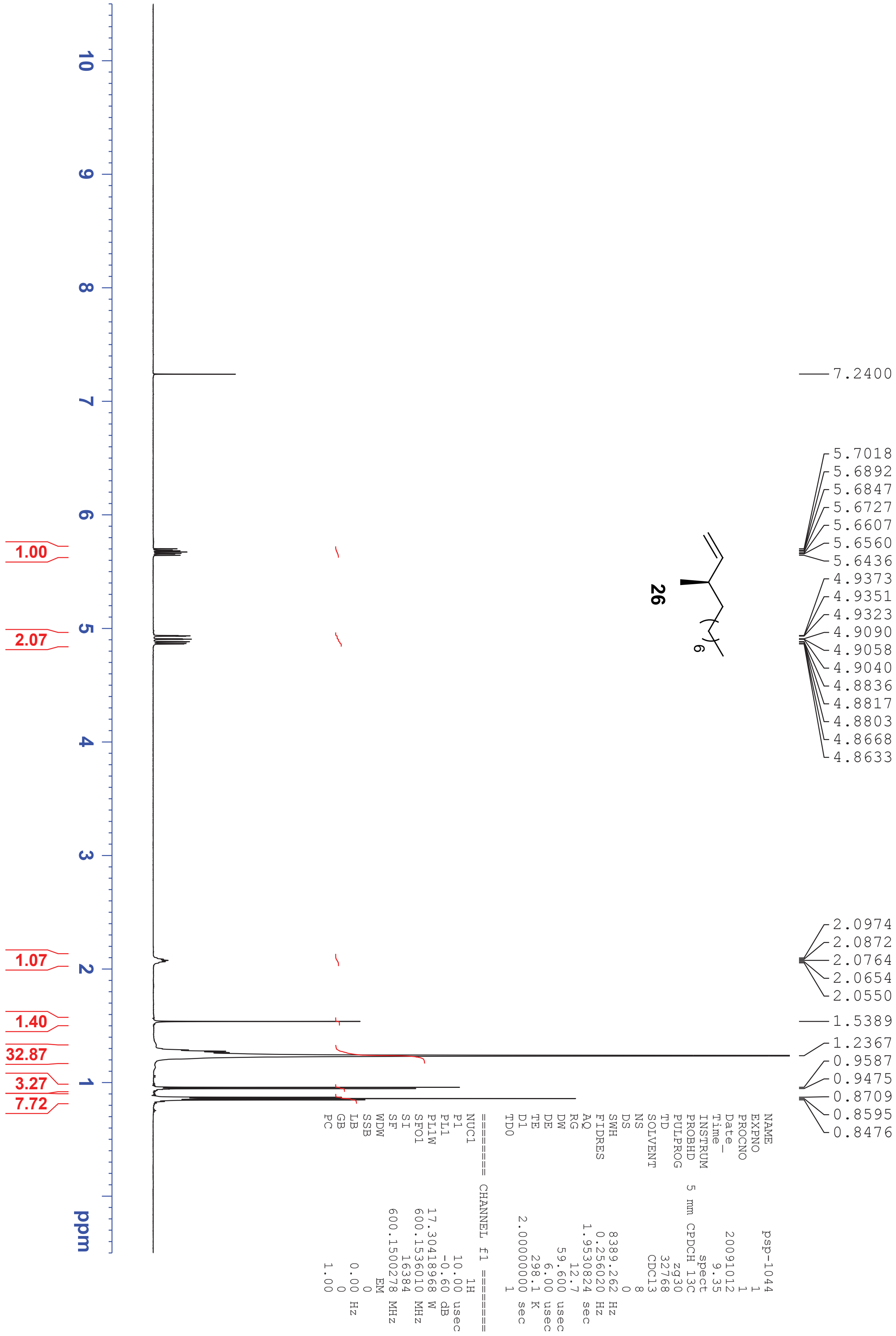

Supplementary Figure 67. ^{1}H NMR spectrum of compound 26.

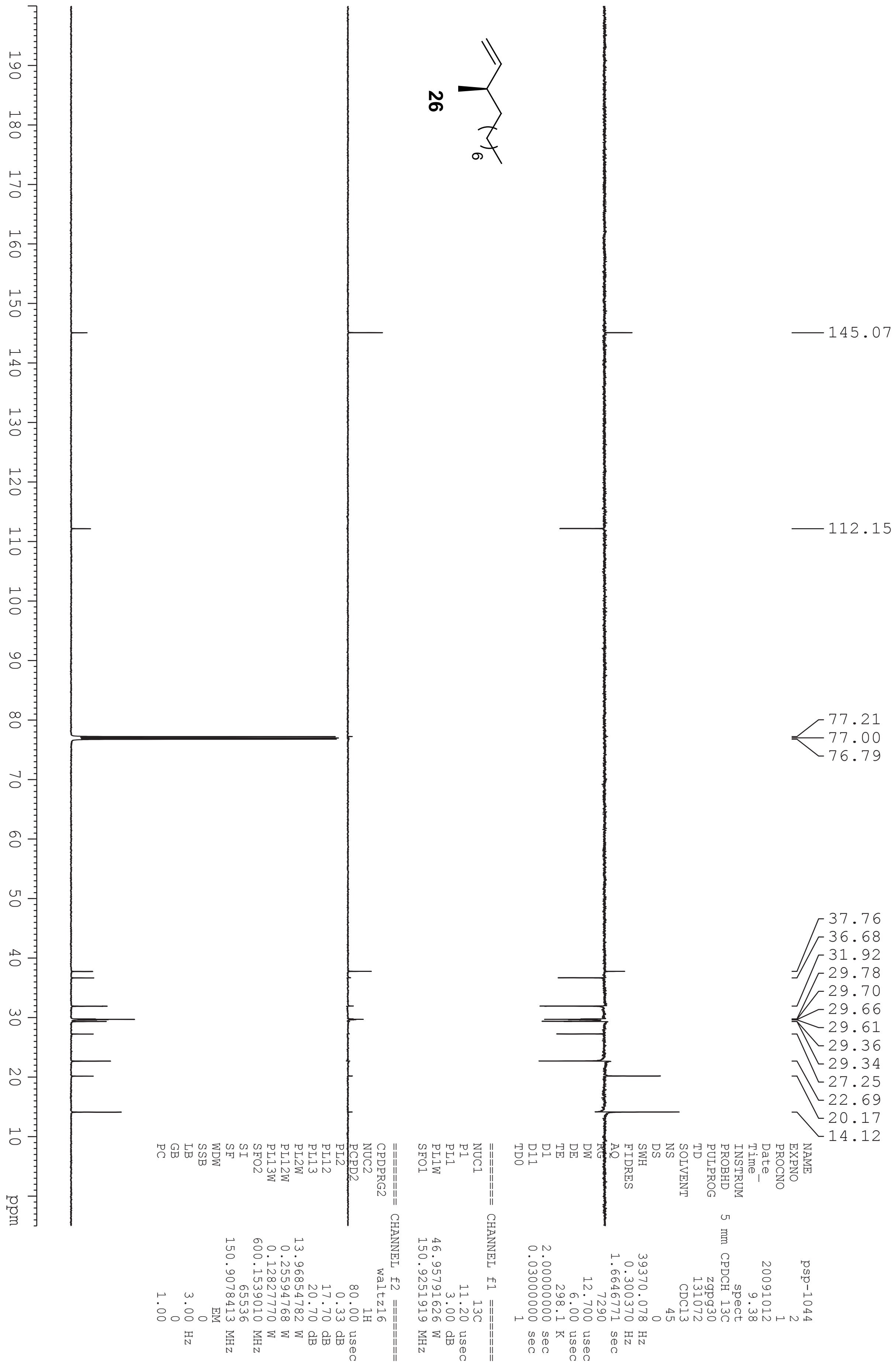

Supplementary Figure 68. ^{13}C and DEPT NMR spectra of compound 26.

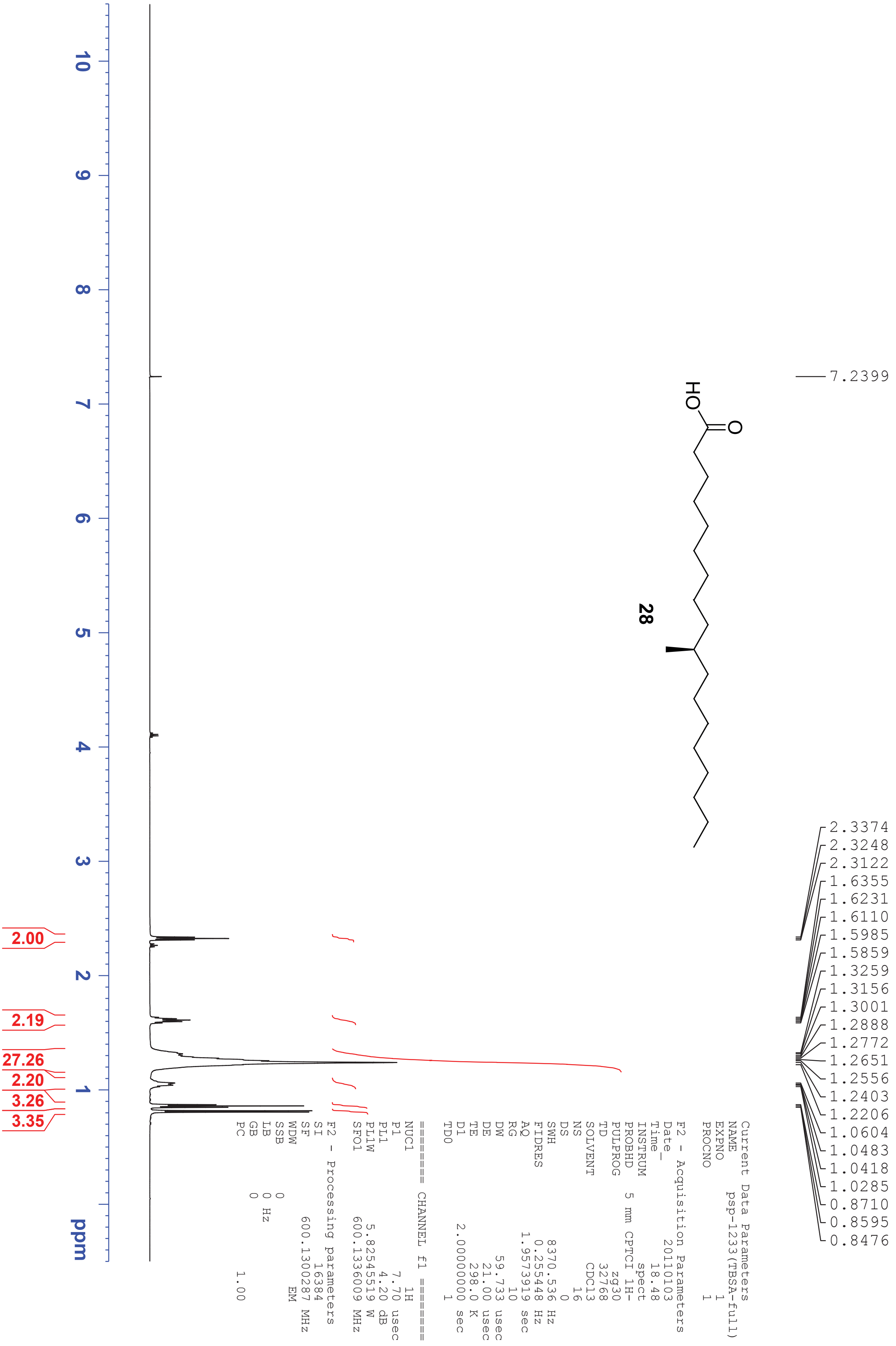

Supplementary Figure 69. ^{1}H NMR spectrum of compound 28.

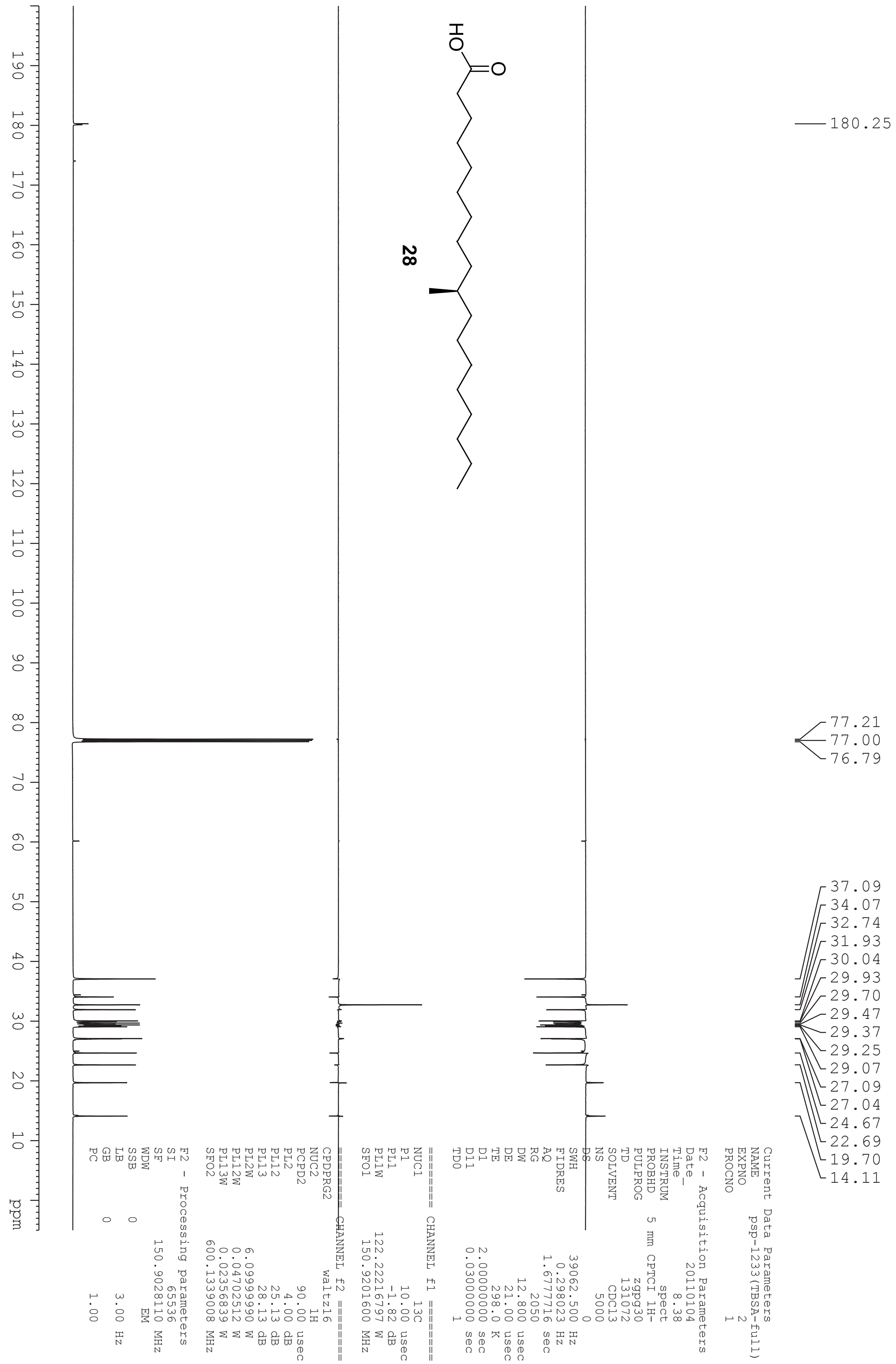

Supplementary Figure 70. ^{13}C and DEPT NMR spectra of compound 27.

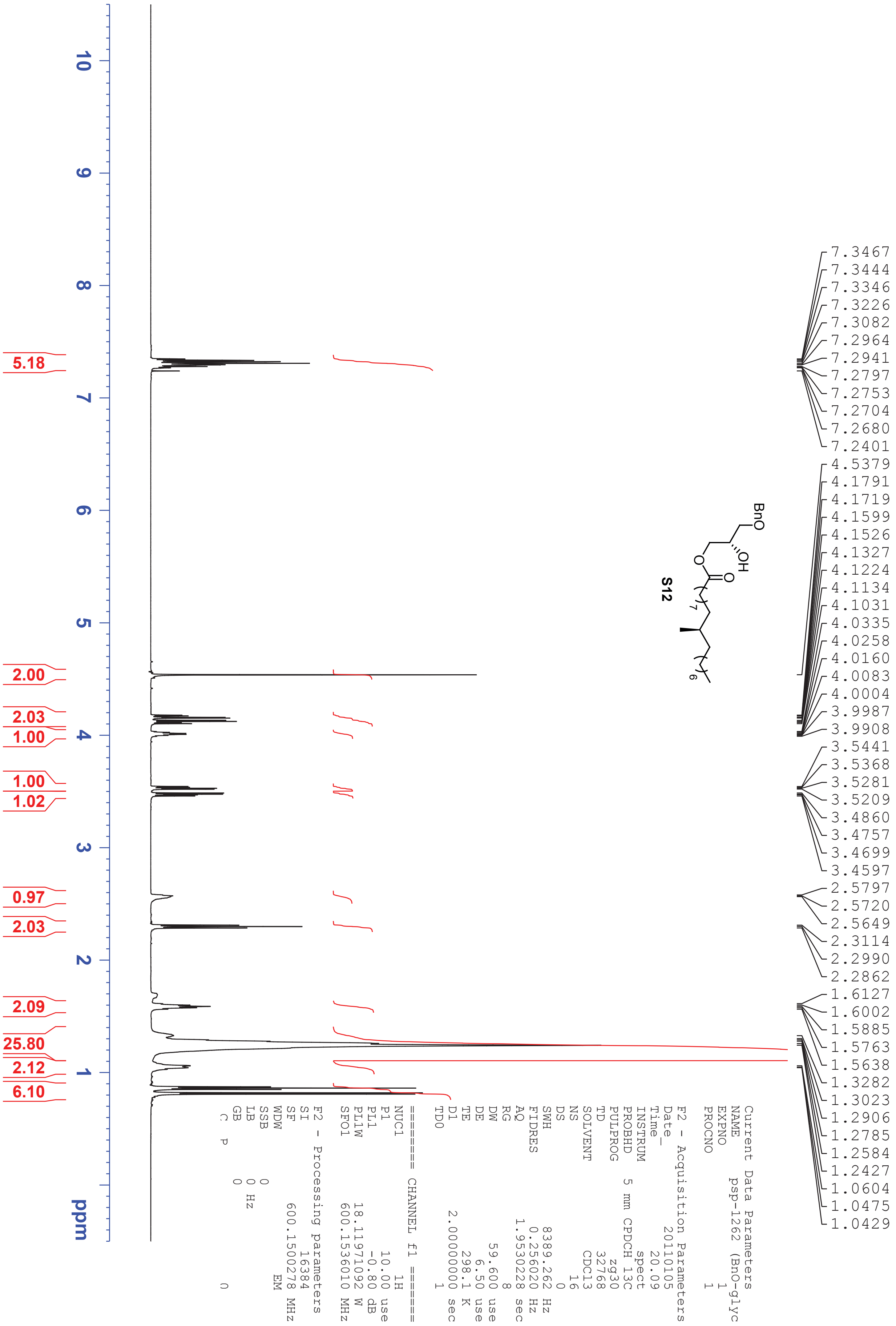

Supplementary Figure 71. ^{1}H NMR spectrum of compound S12.

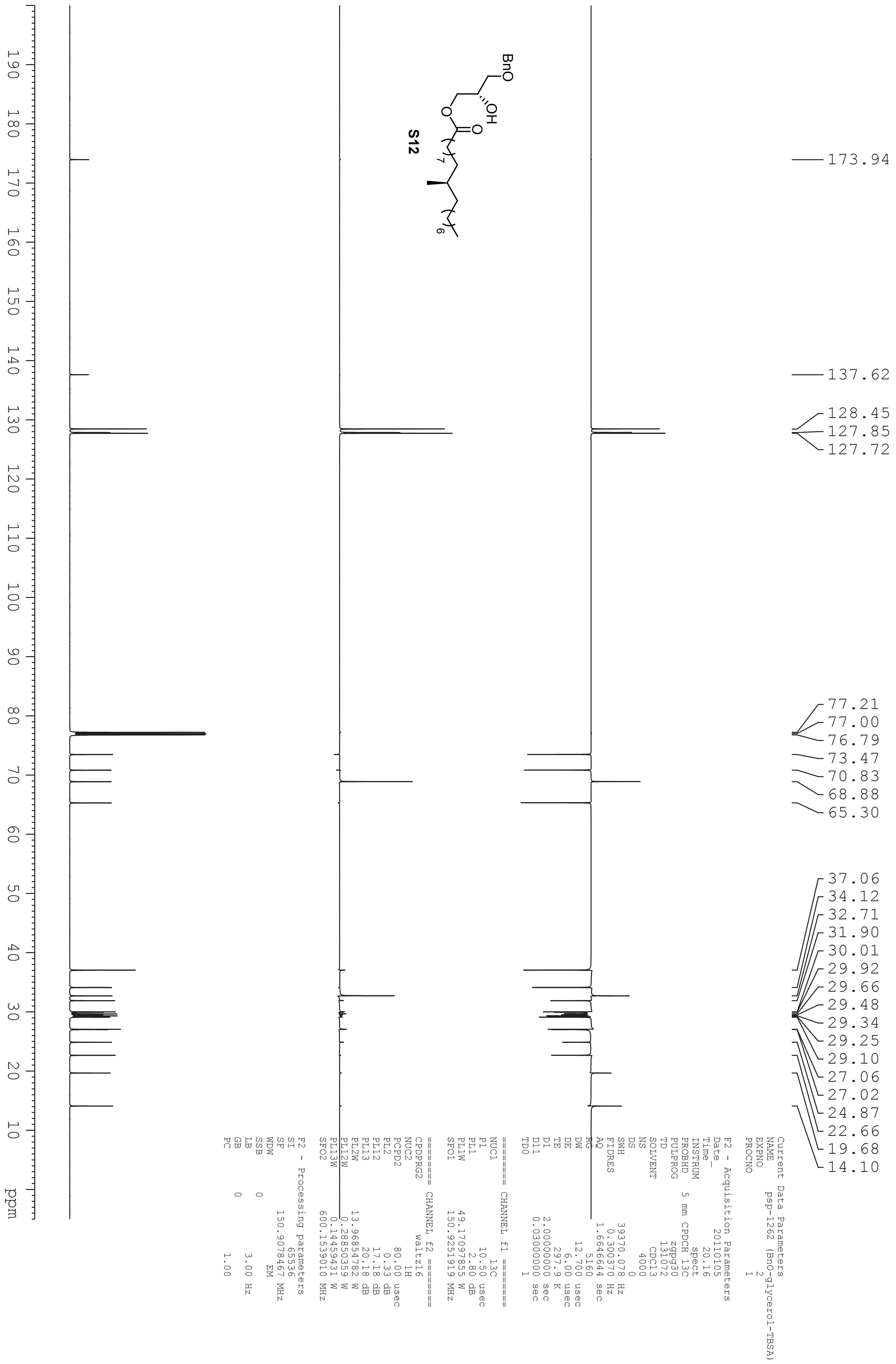

Supplementary Figure 72. **^{13}C and DEPT NMR spectra of compound S12.**

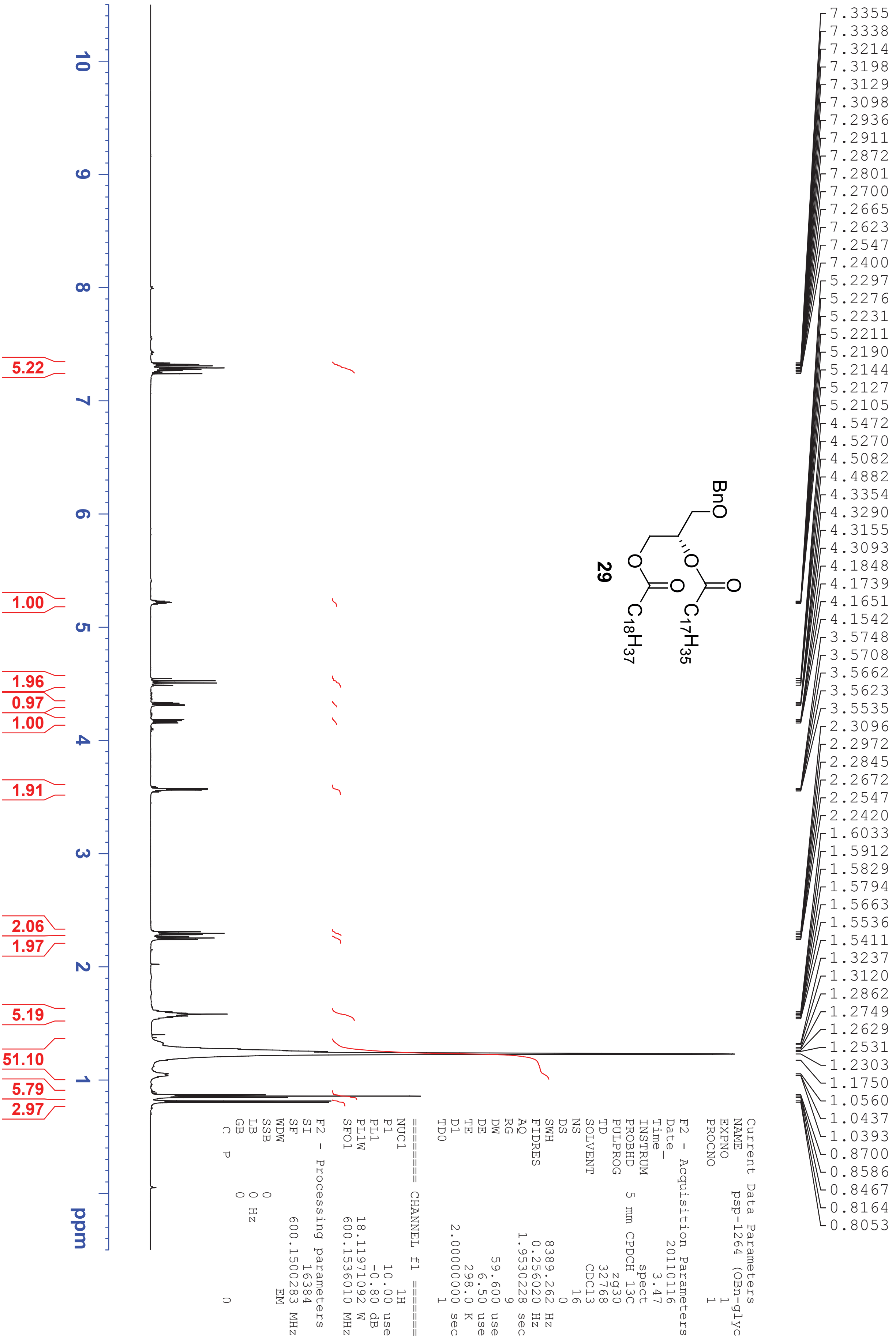

Supplementary Figure 73. ^{1}**H NMR spectrum of compound 29.**

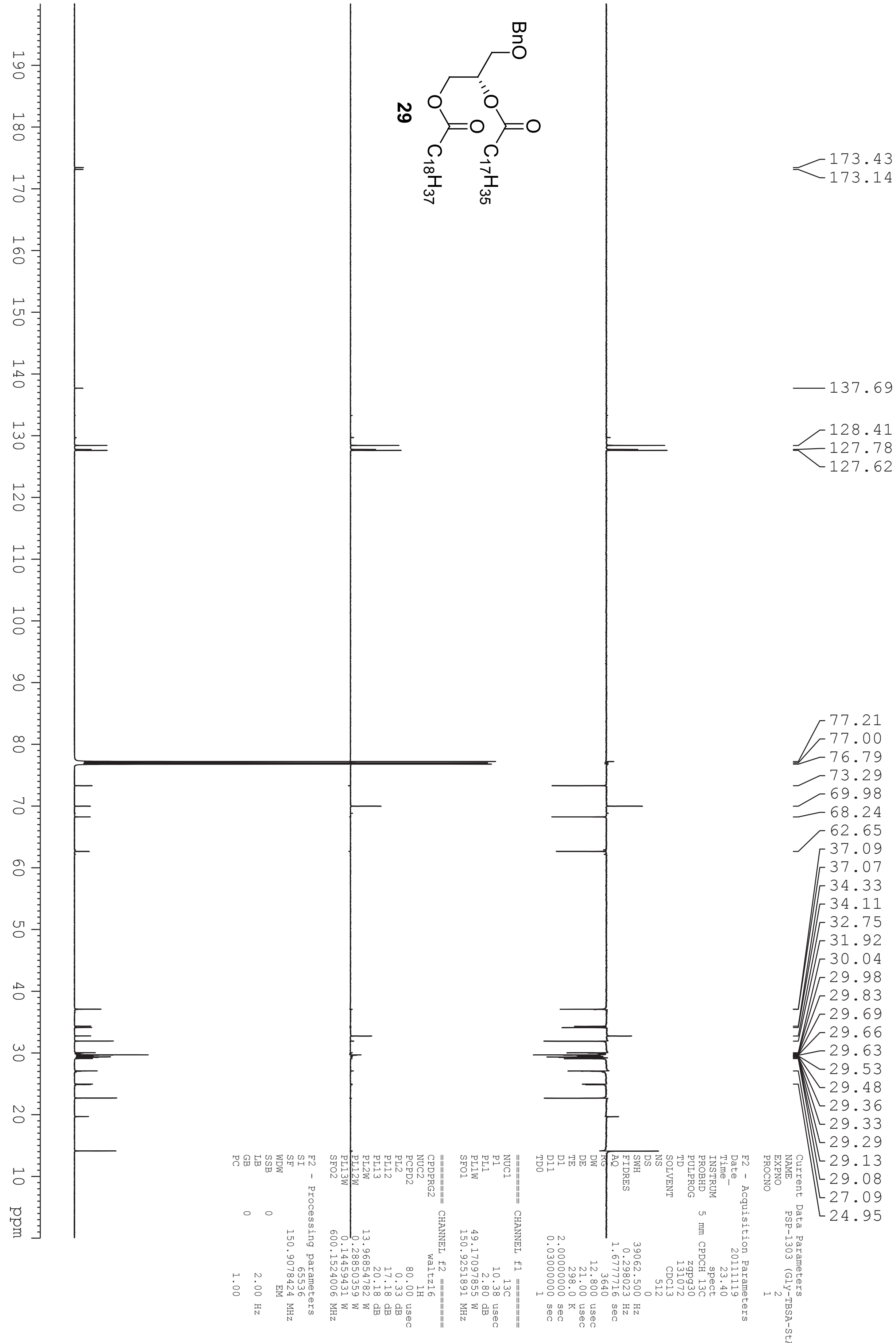

Supplementary Figure 74. ^{13}C and DEPT NMR spectra of compound 29.

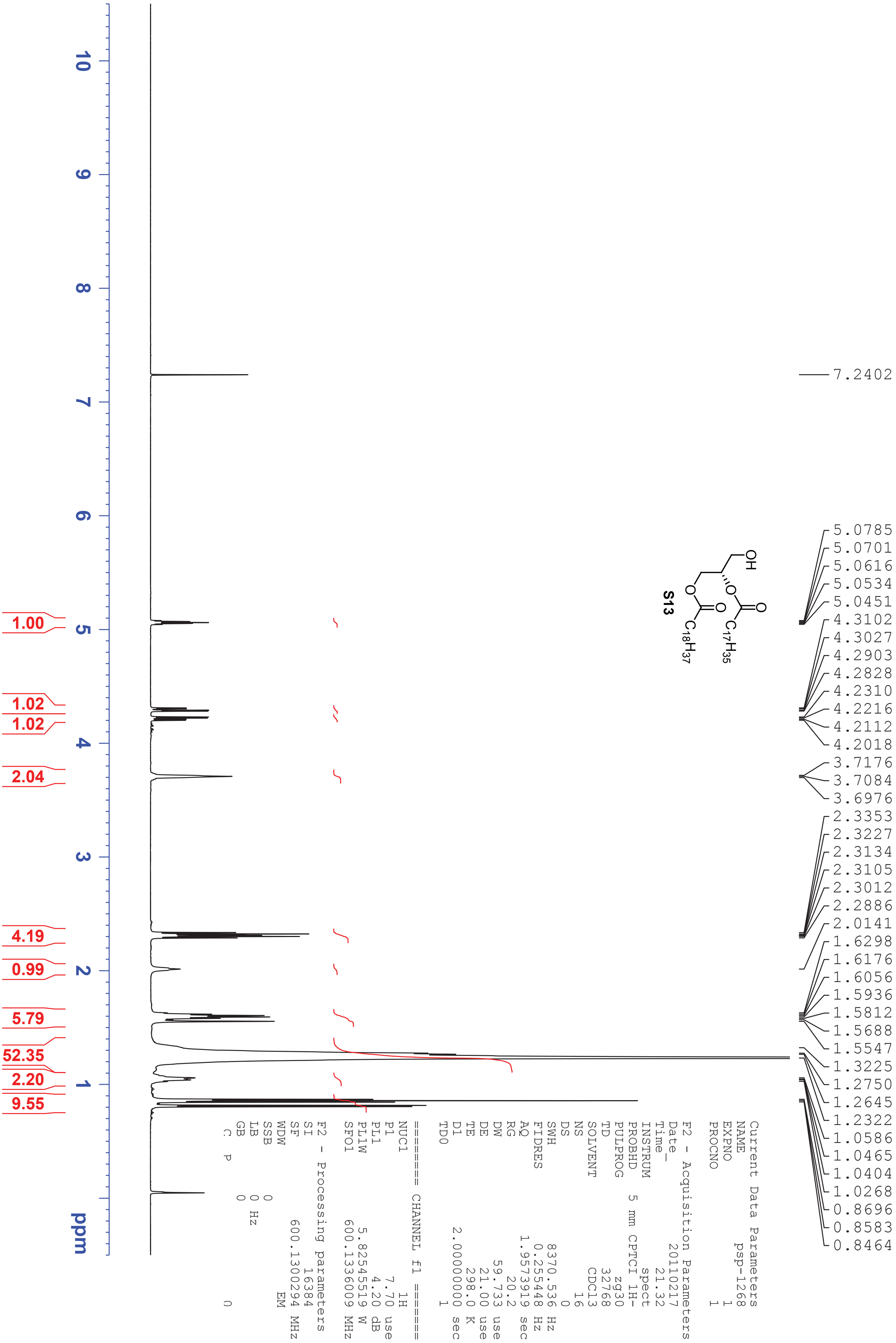

Supplementary Figure 75. ^{1}**H NMR spectrum of compound S13.**

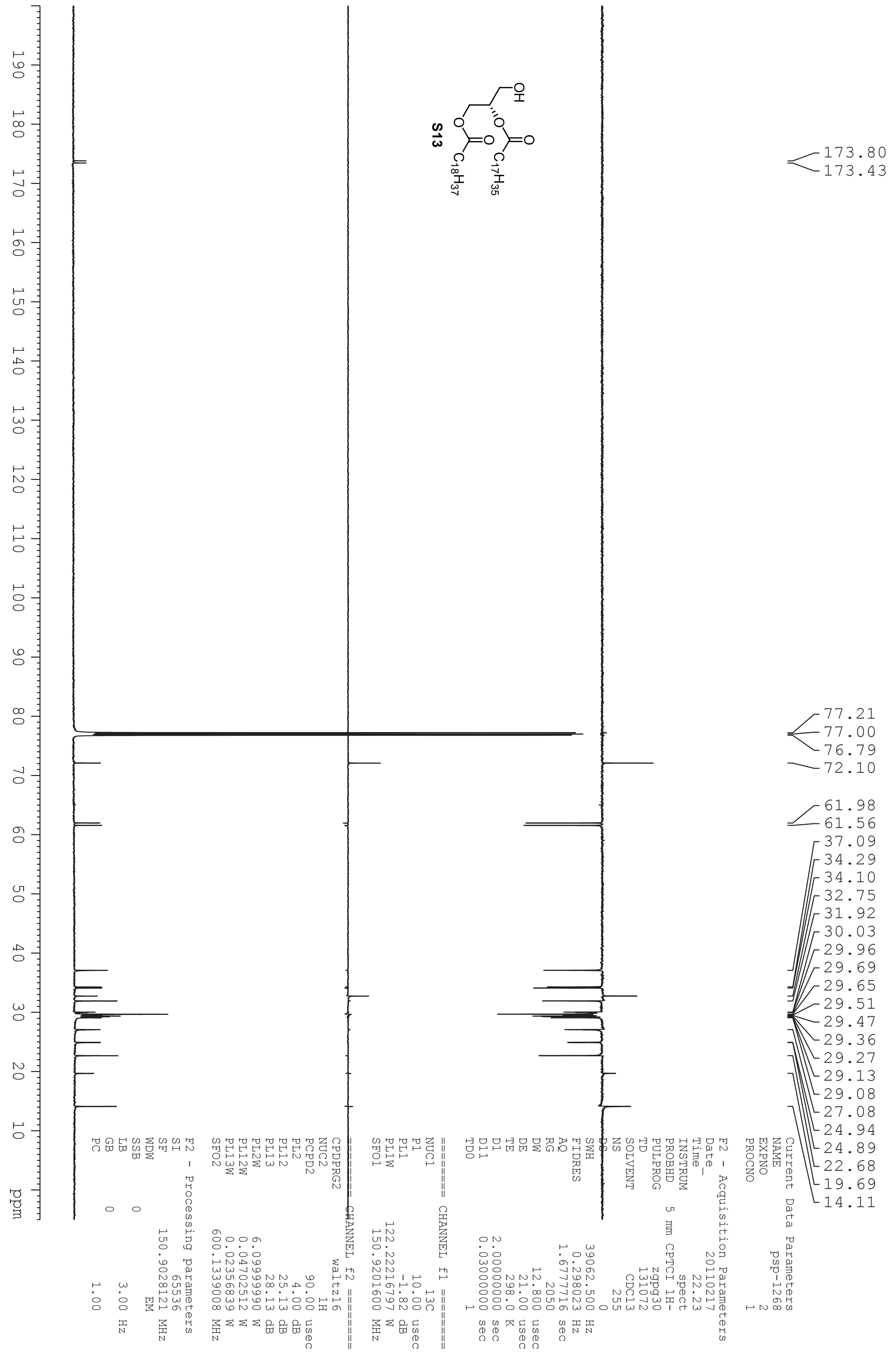

Supplementary Figure 76. ^{13}C and DEPT NMR spectra of compound S13.

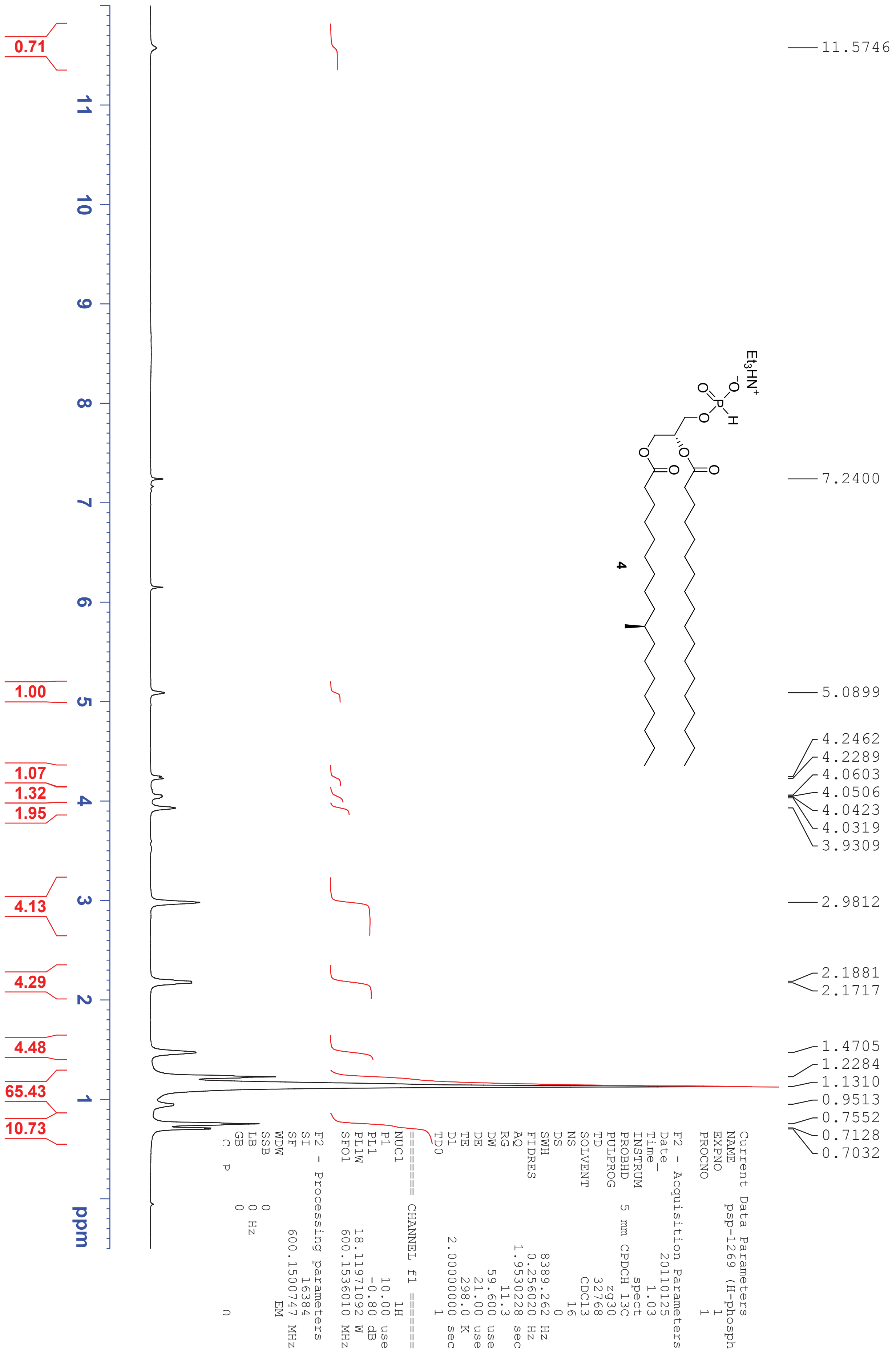

Supplementary Figure 77. ^{1}H NMR spectrum of compound 4.

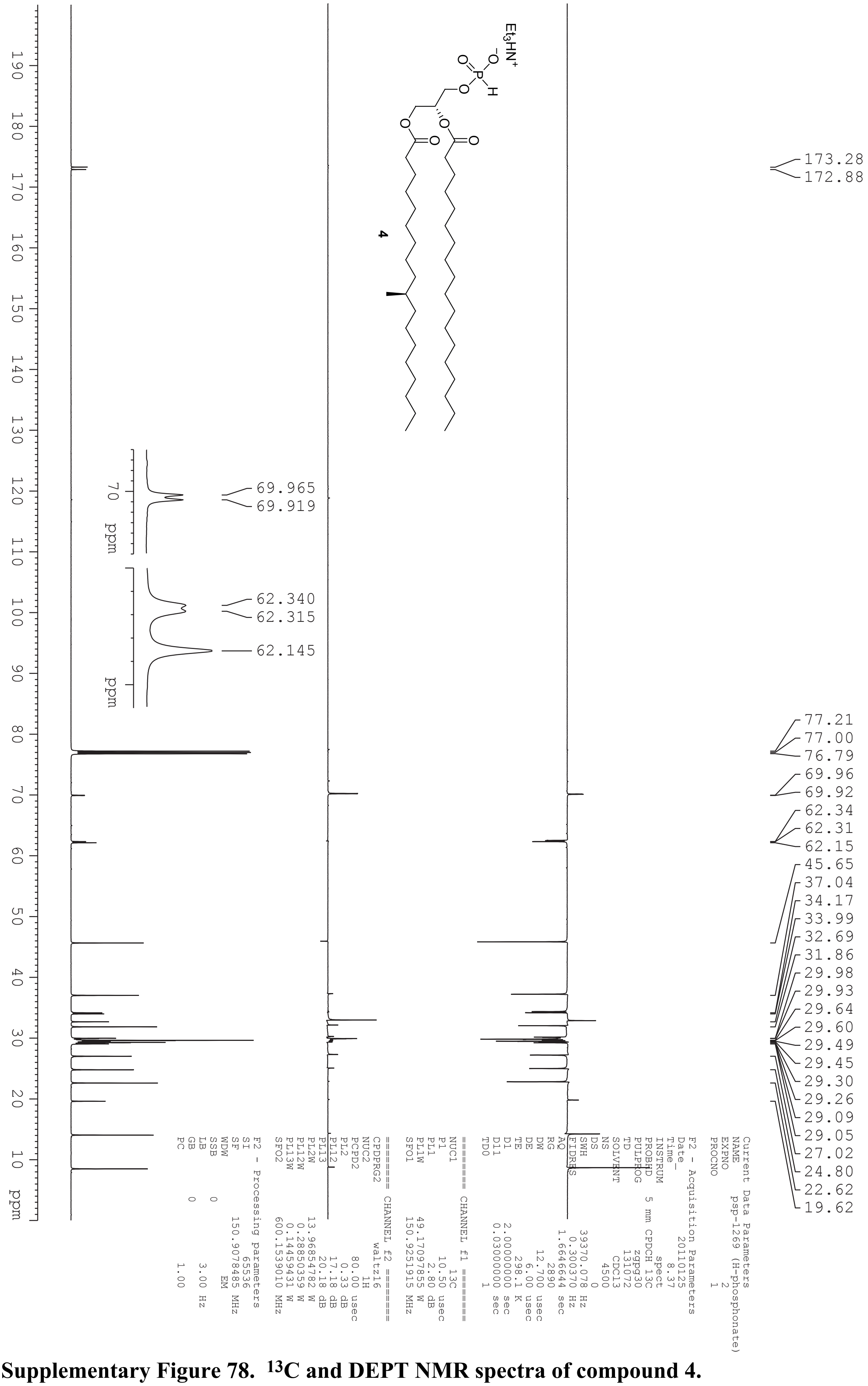

Supplementary Figure 78. ^{13}C and DEPT NMR spectra of compound 4.

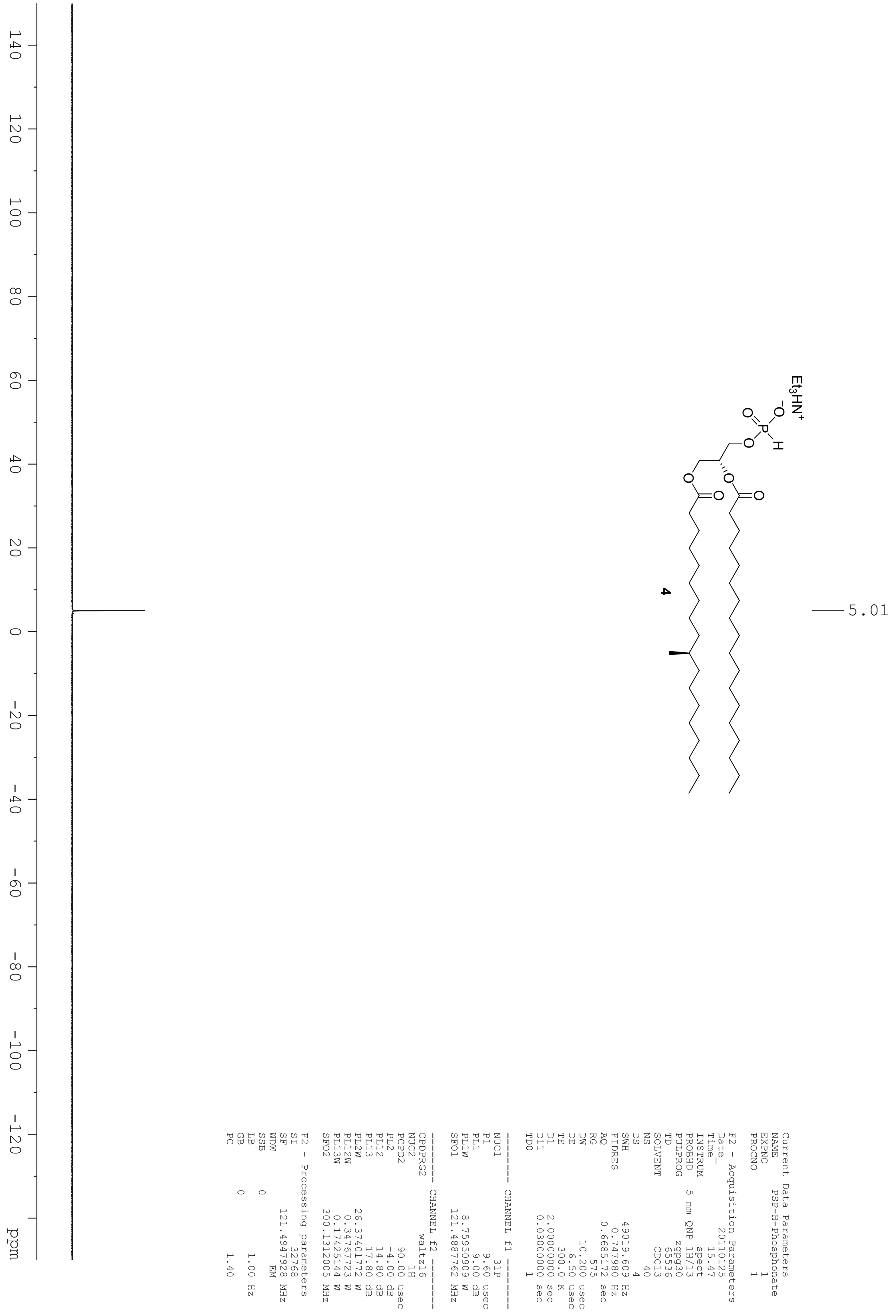

Supplementary Figure 79. ^{31}P NMR spectrum of compound 4.

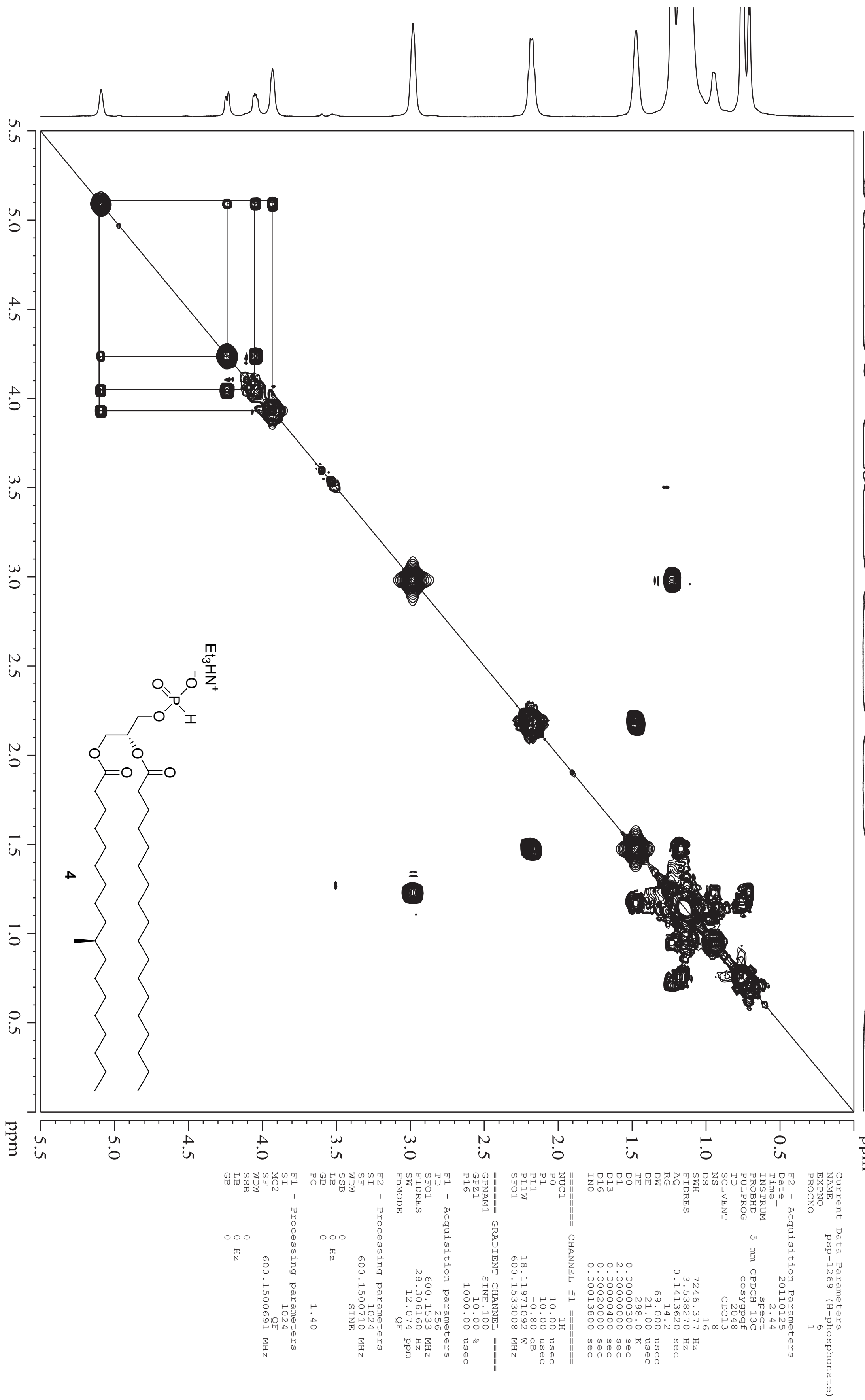

Supplementary Figure 80. COSY NMR spectrum of compound 4.

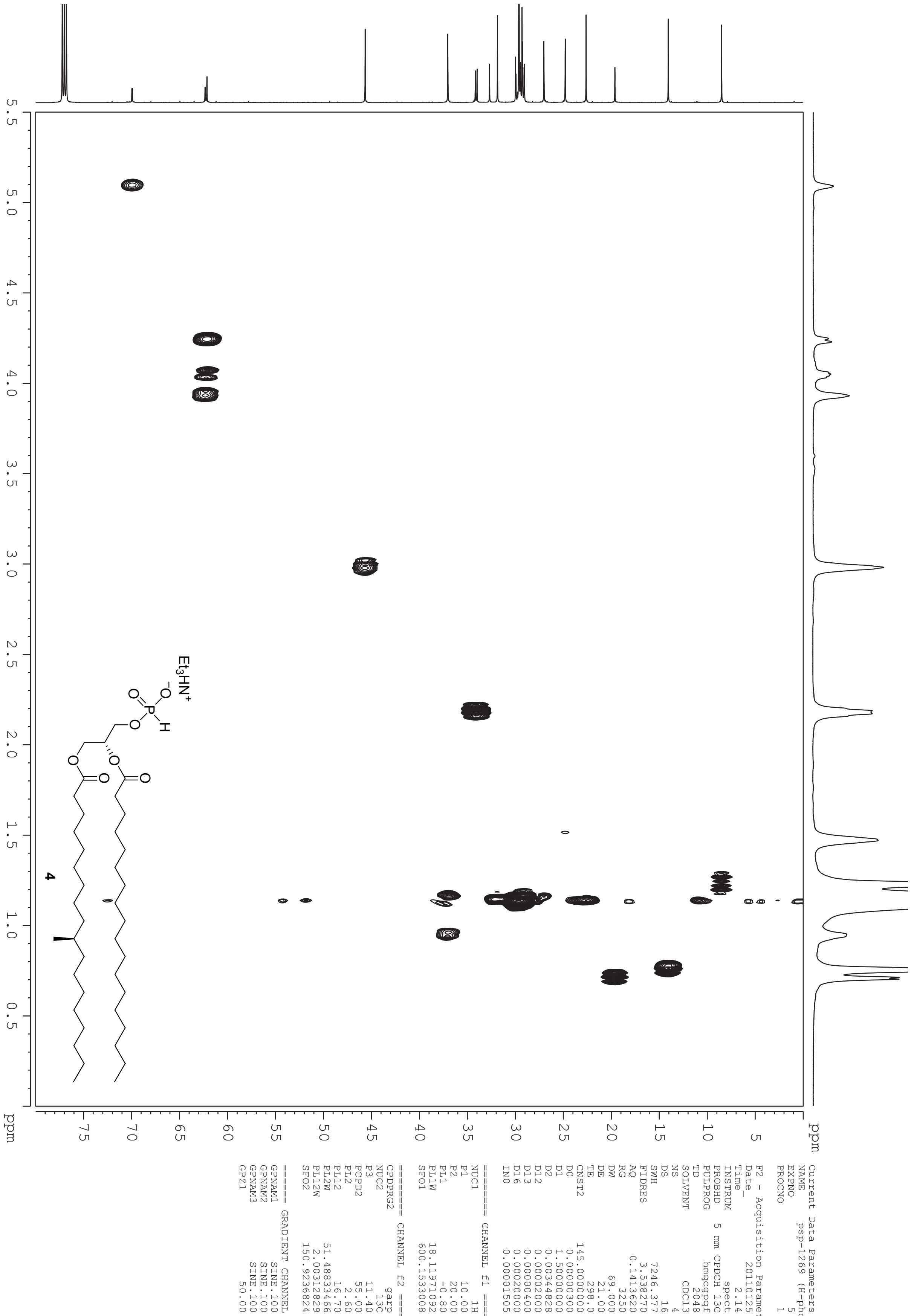

Supplementary Figure 81. HMQC NMR spectrum of compound 4.

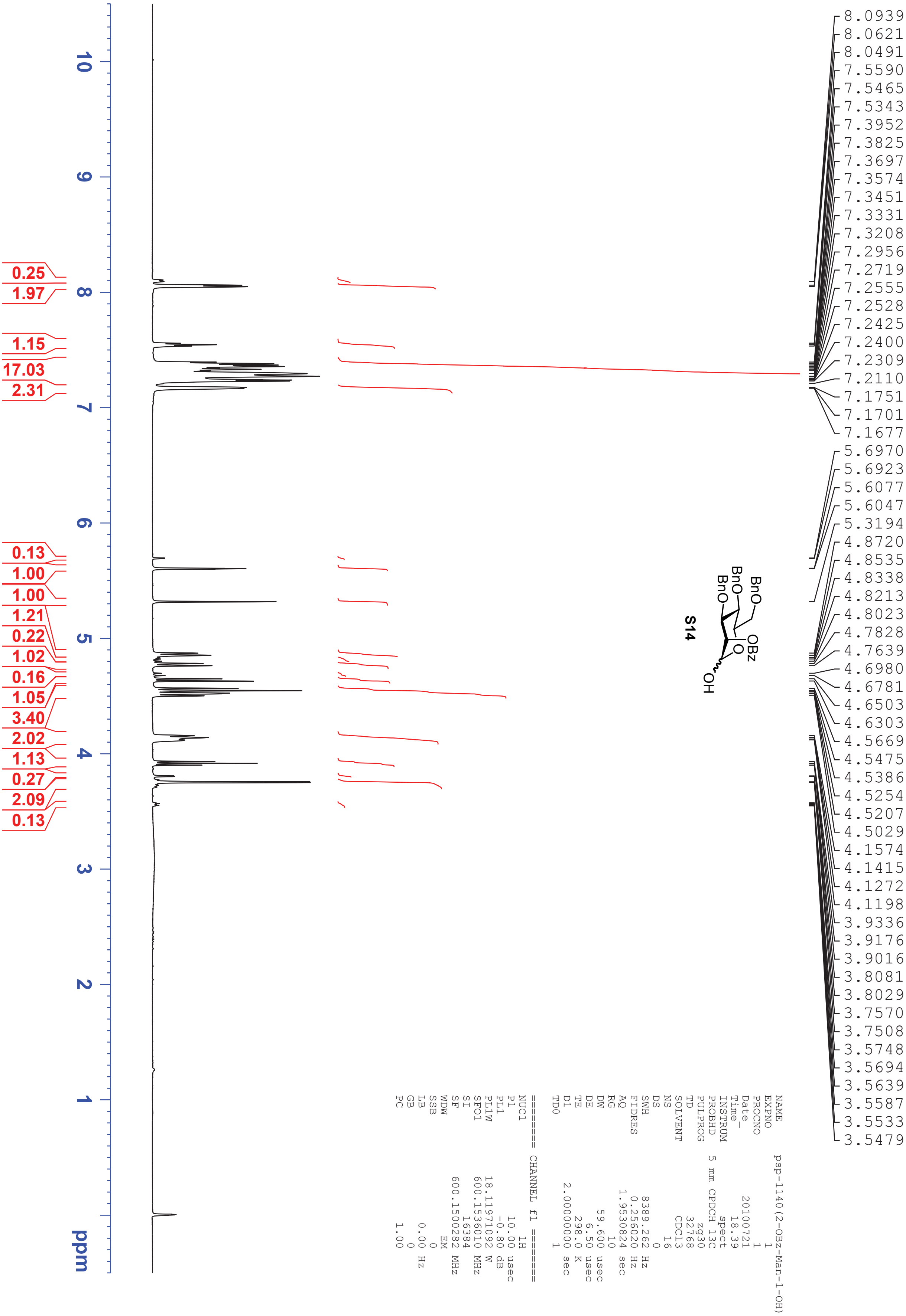

Supplementary Figure 82. ^{1}H NMR spectrum of compound S14.

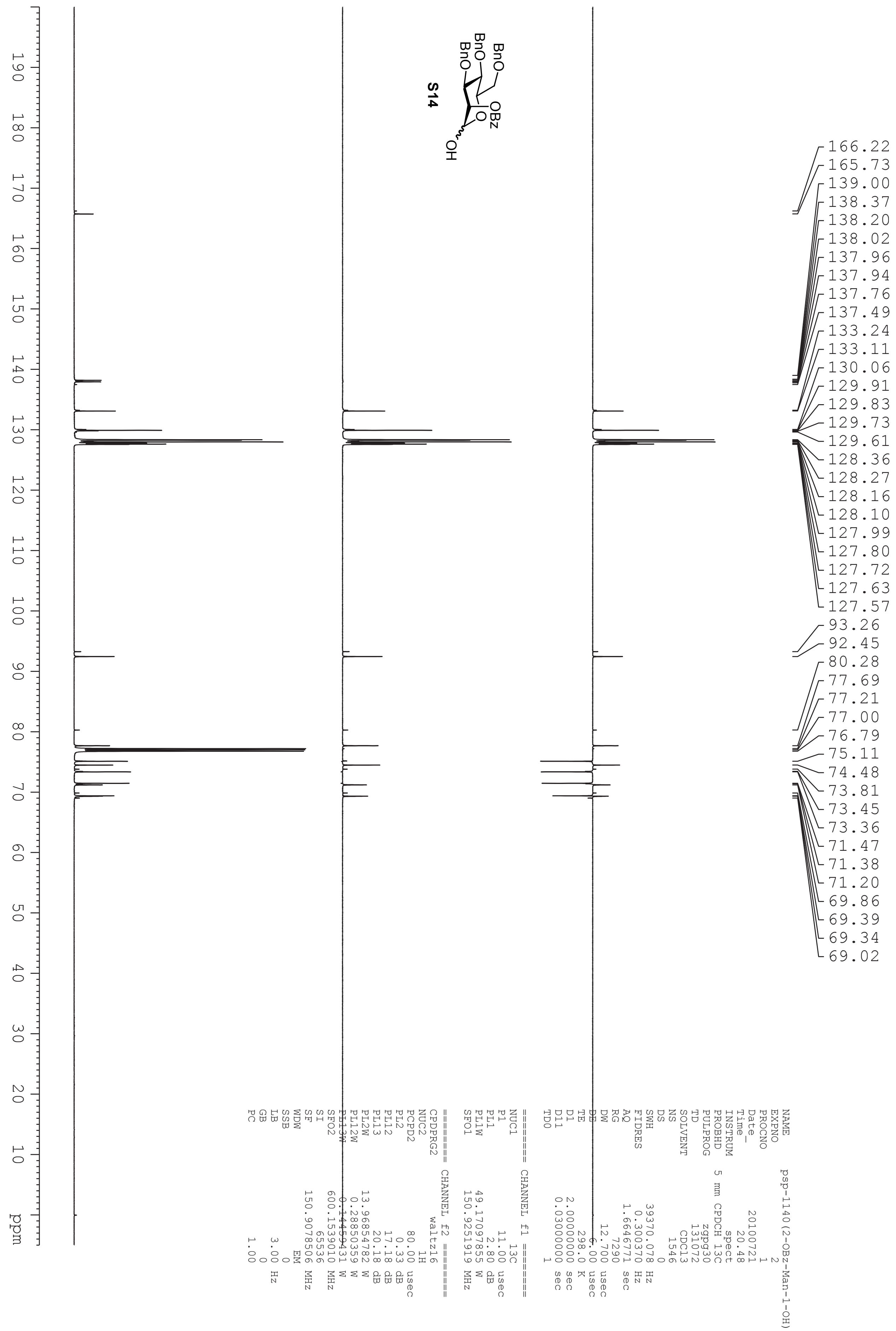

Supplementary Figure 83. ^{13}C and DEPT NMR spectra of compound S14.

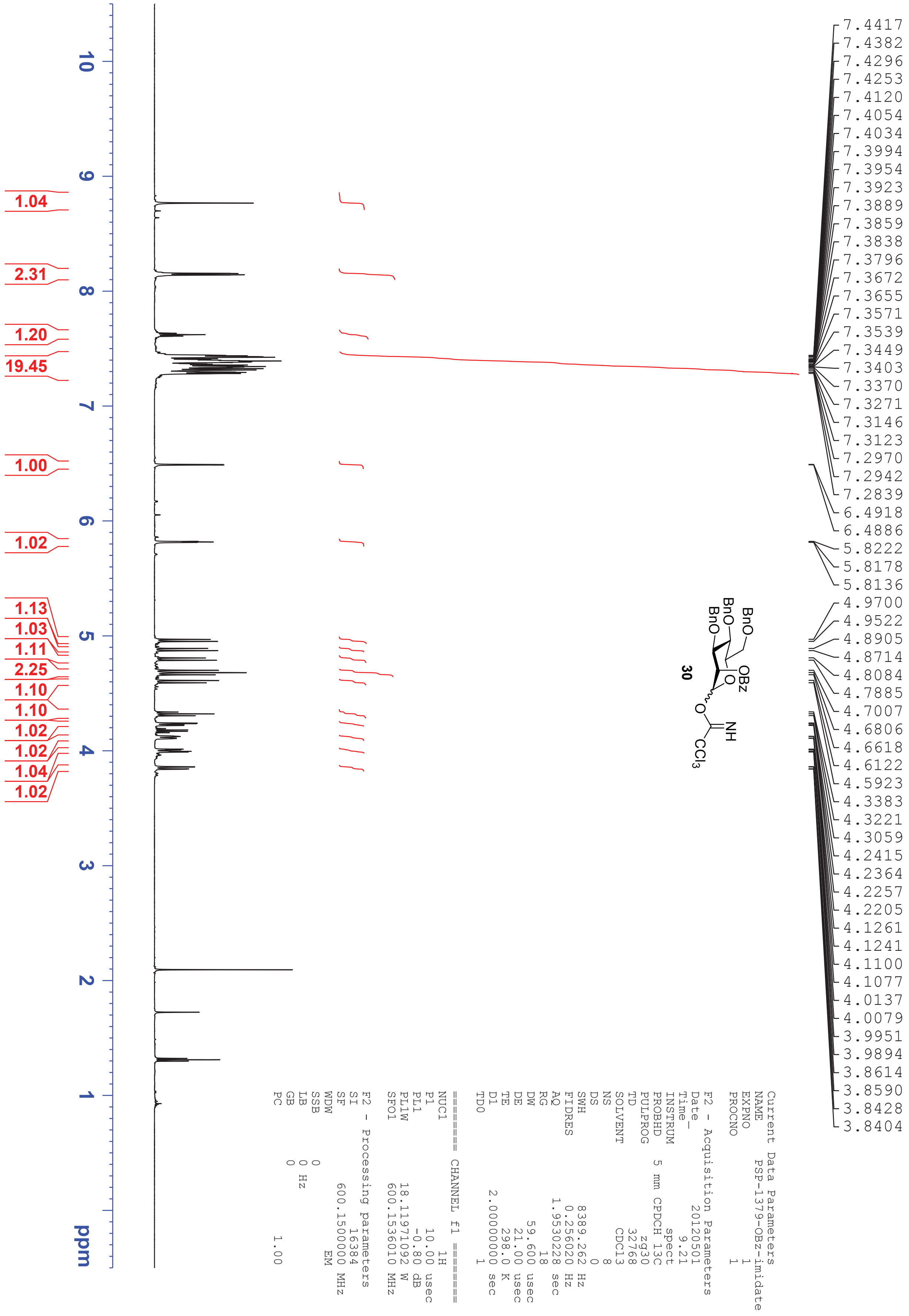

Supplementary Figure 84. ^{1}**H NMR spectrum of compound 30.**

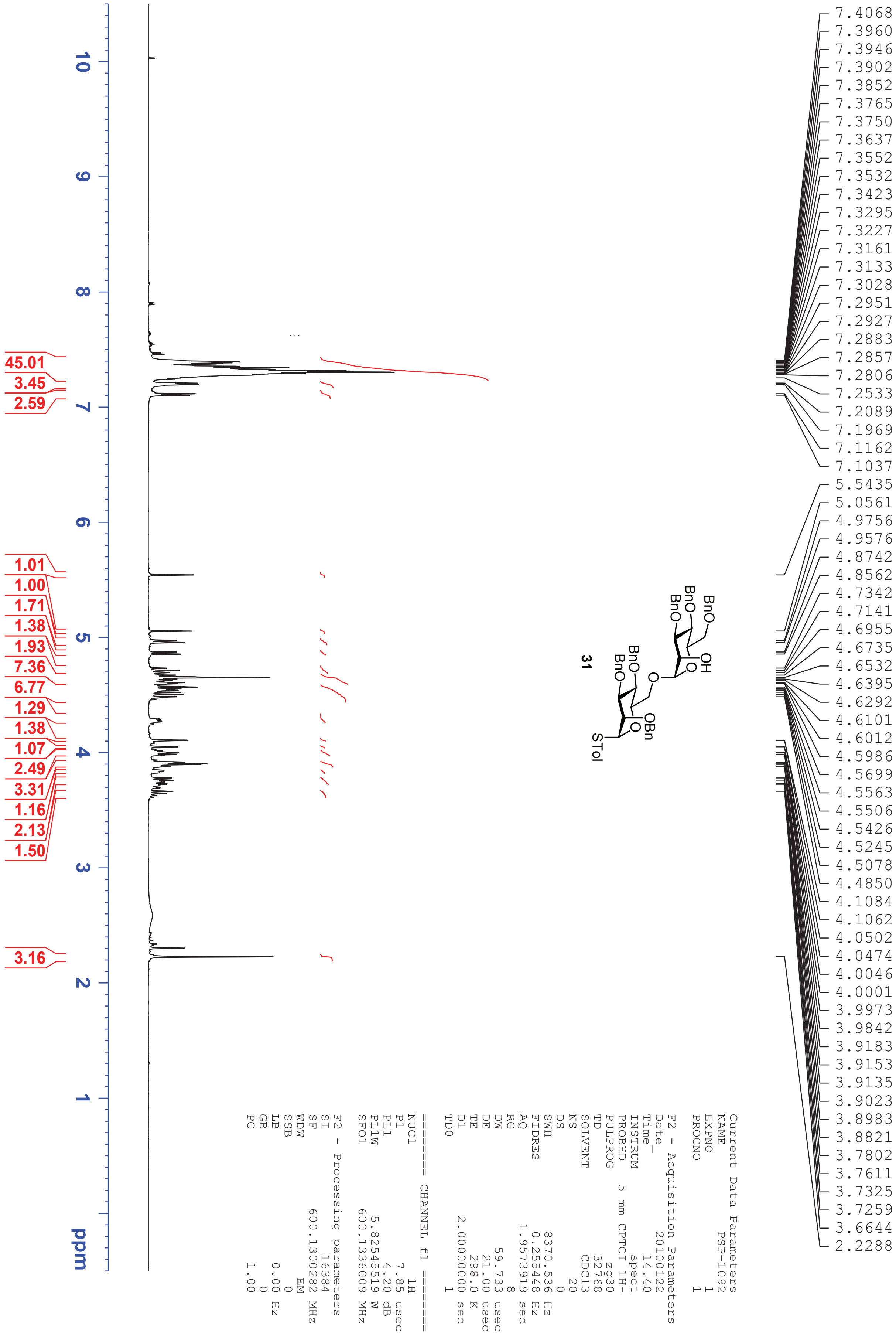

Supplementary Figure 85. ^{1}**H NMR spectrum of compound 31.**

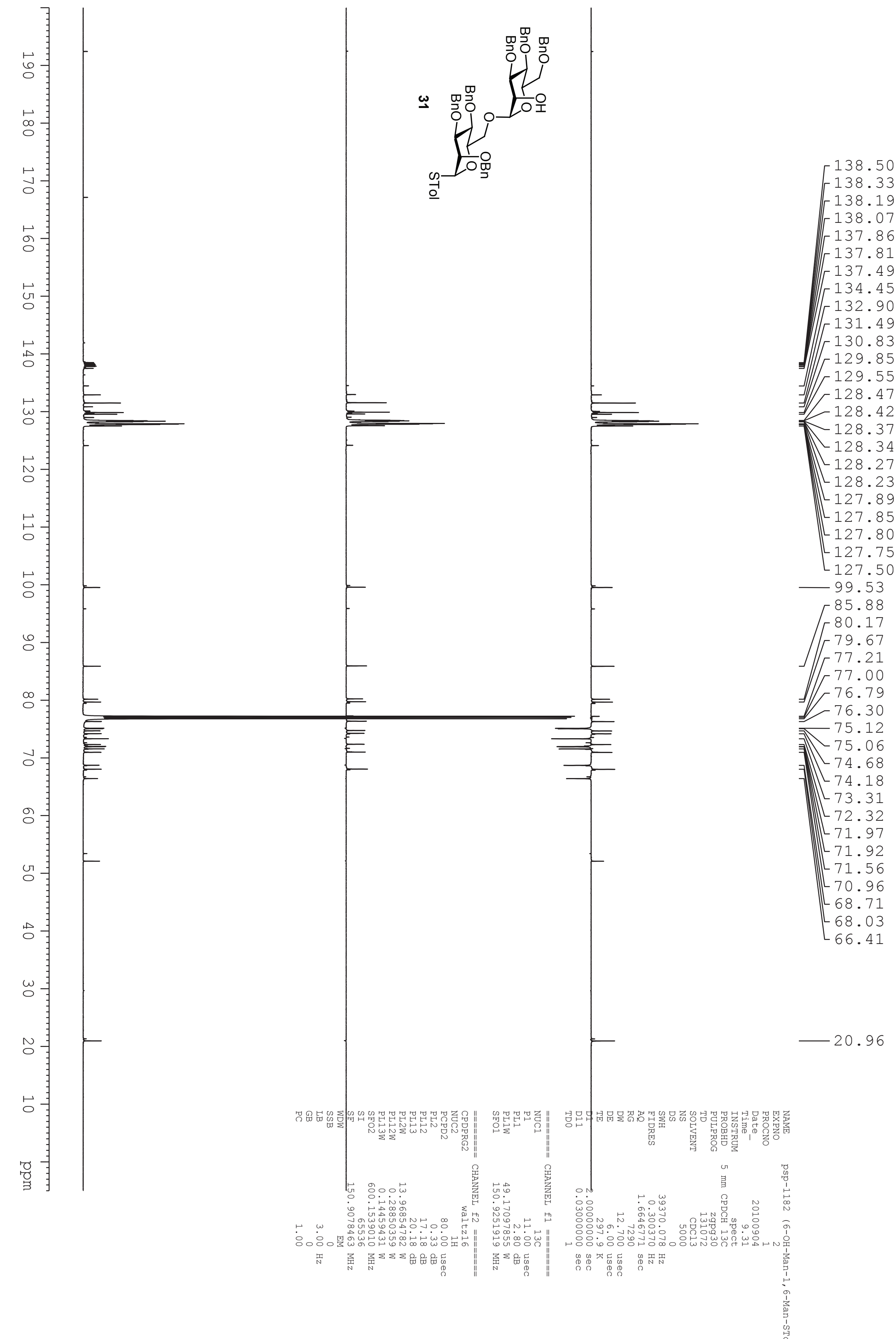

Supplementary Figure 86. ^{13}C and DEPT NMR spectra of compound 31.

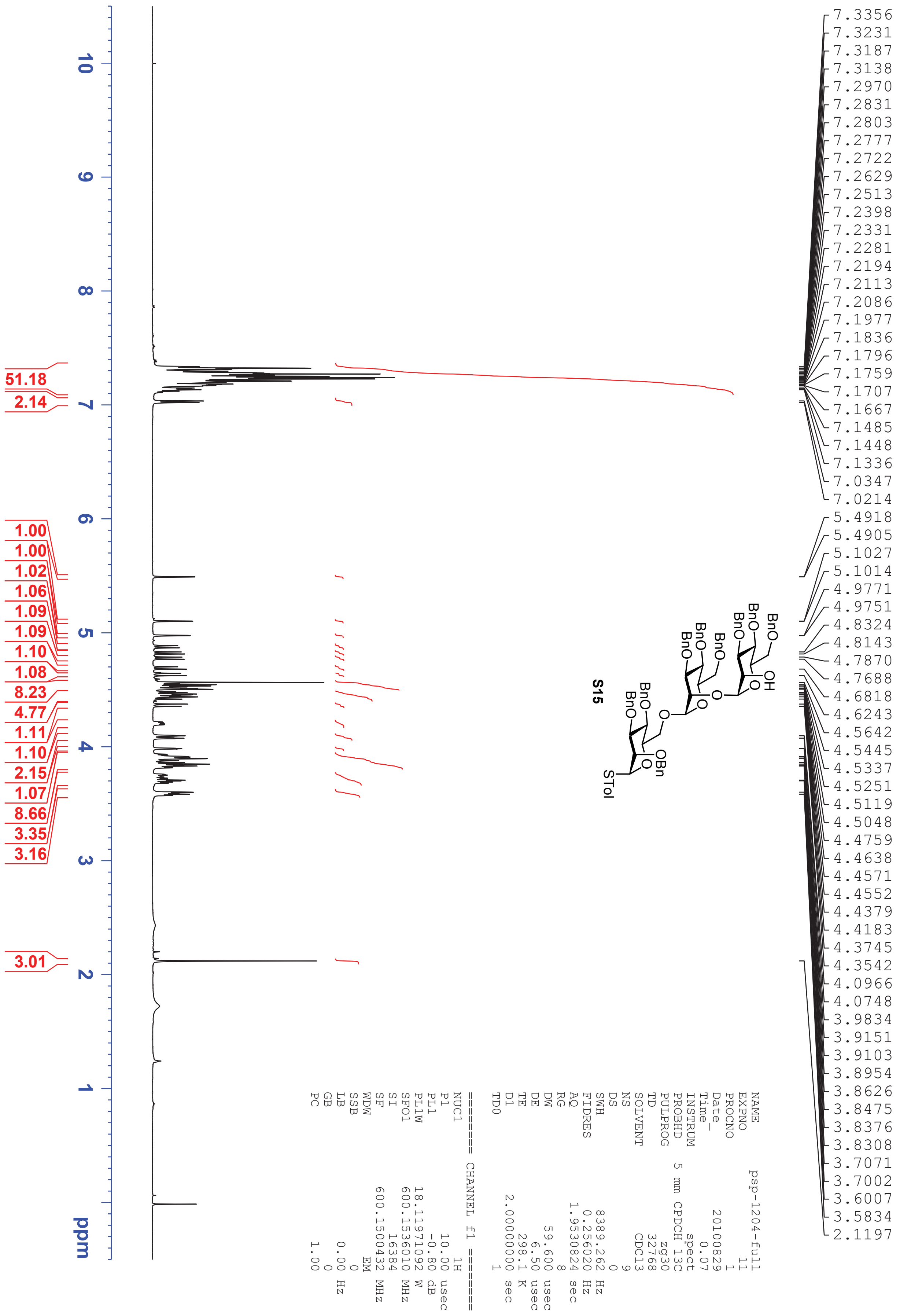

Supplementary Figure 87. ^{1}H NMR spectrum of compound S15.

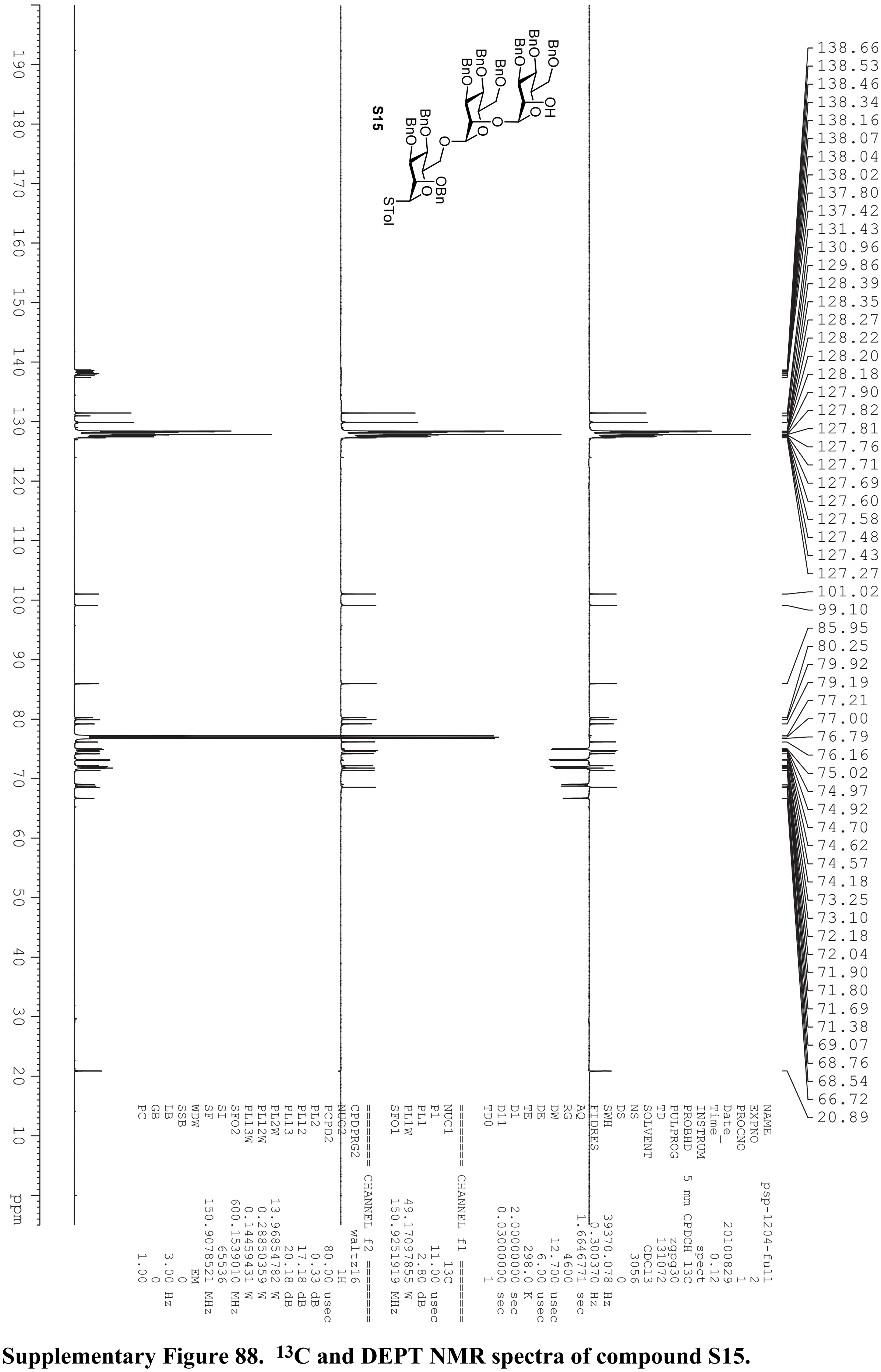

Supplementary Figure 88. ^{13}C and DEPT NMR spectra of compound S15.

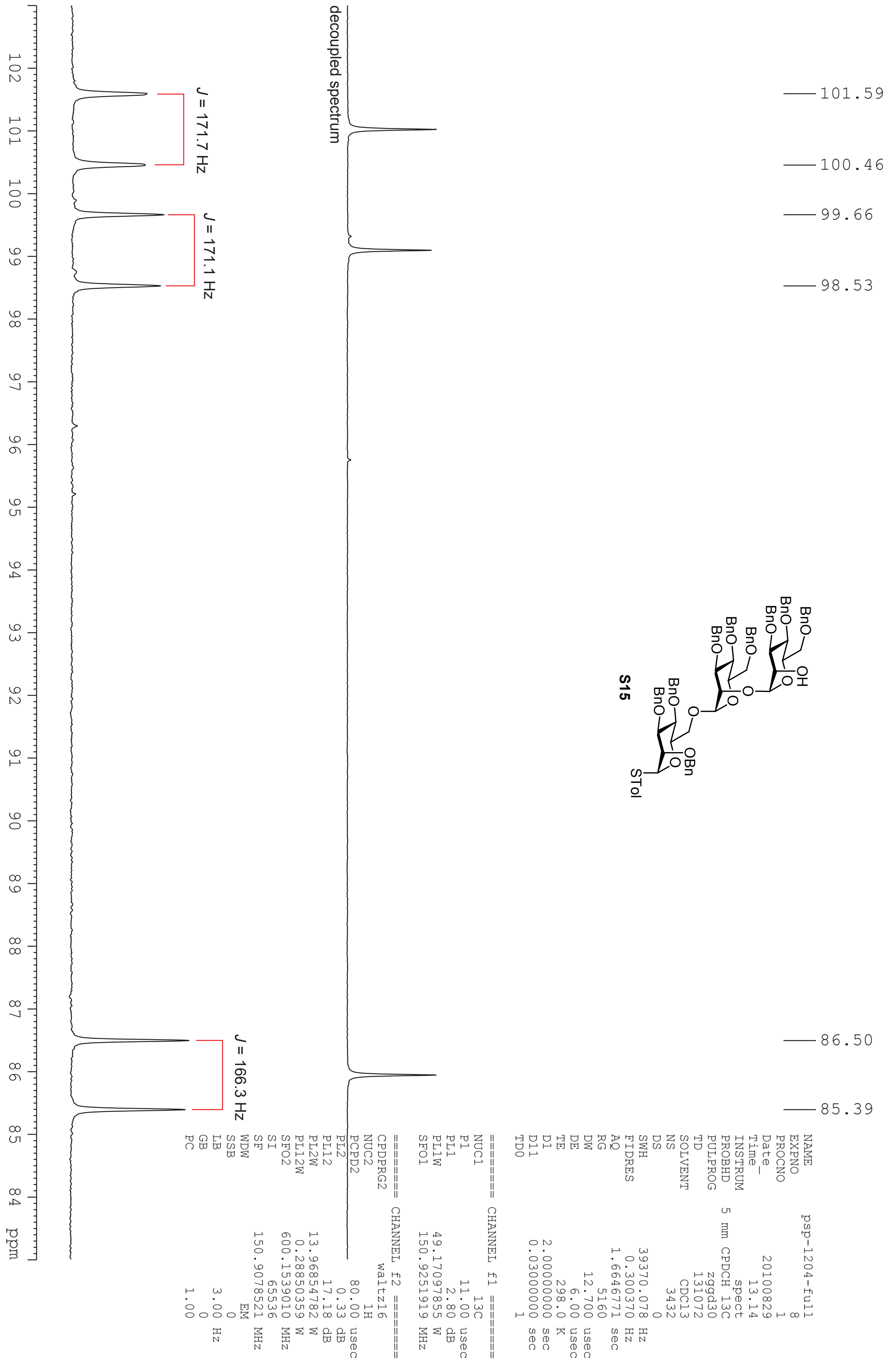

Supplementary Figure 89. Non-decoupled ^{13}C NMR spectrum of compound S15.

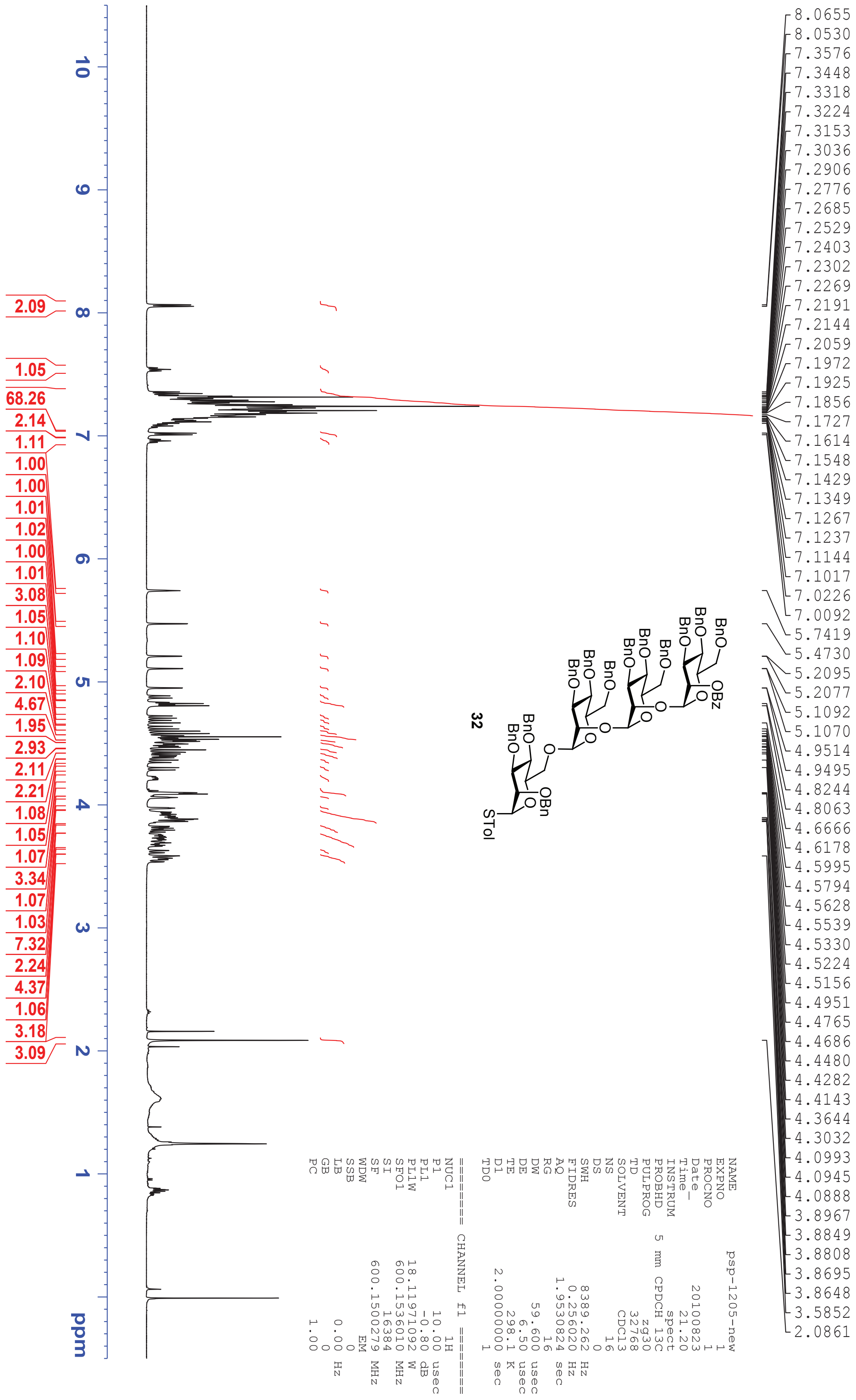

Supplementary Figure 90. ^{1}H NMR spectrum of compound 32.

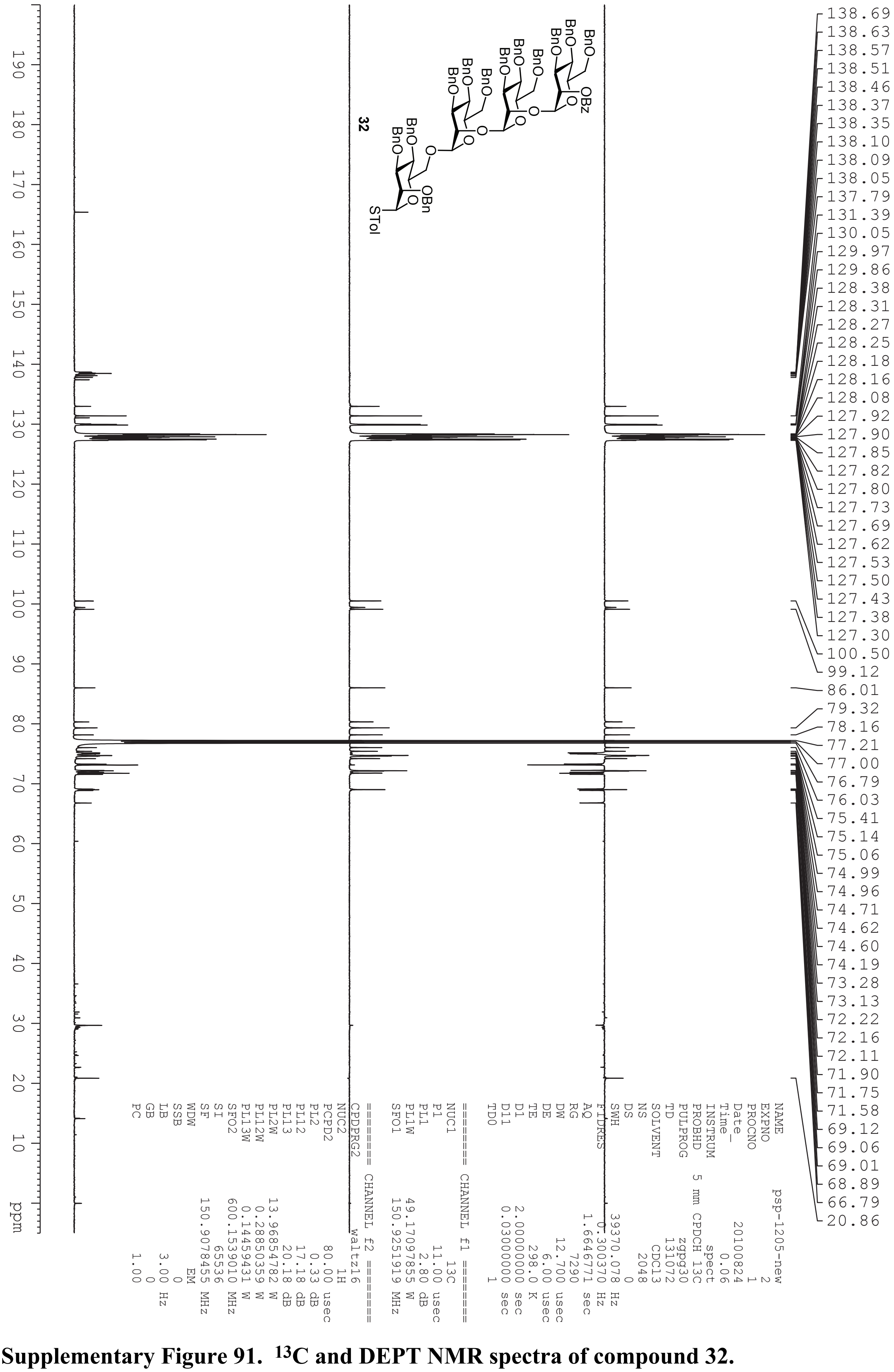

Supplementary Figure 91. ^{13}C and DEPT NMR spectra of compound 32.

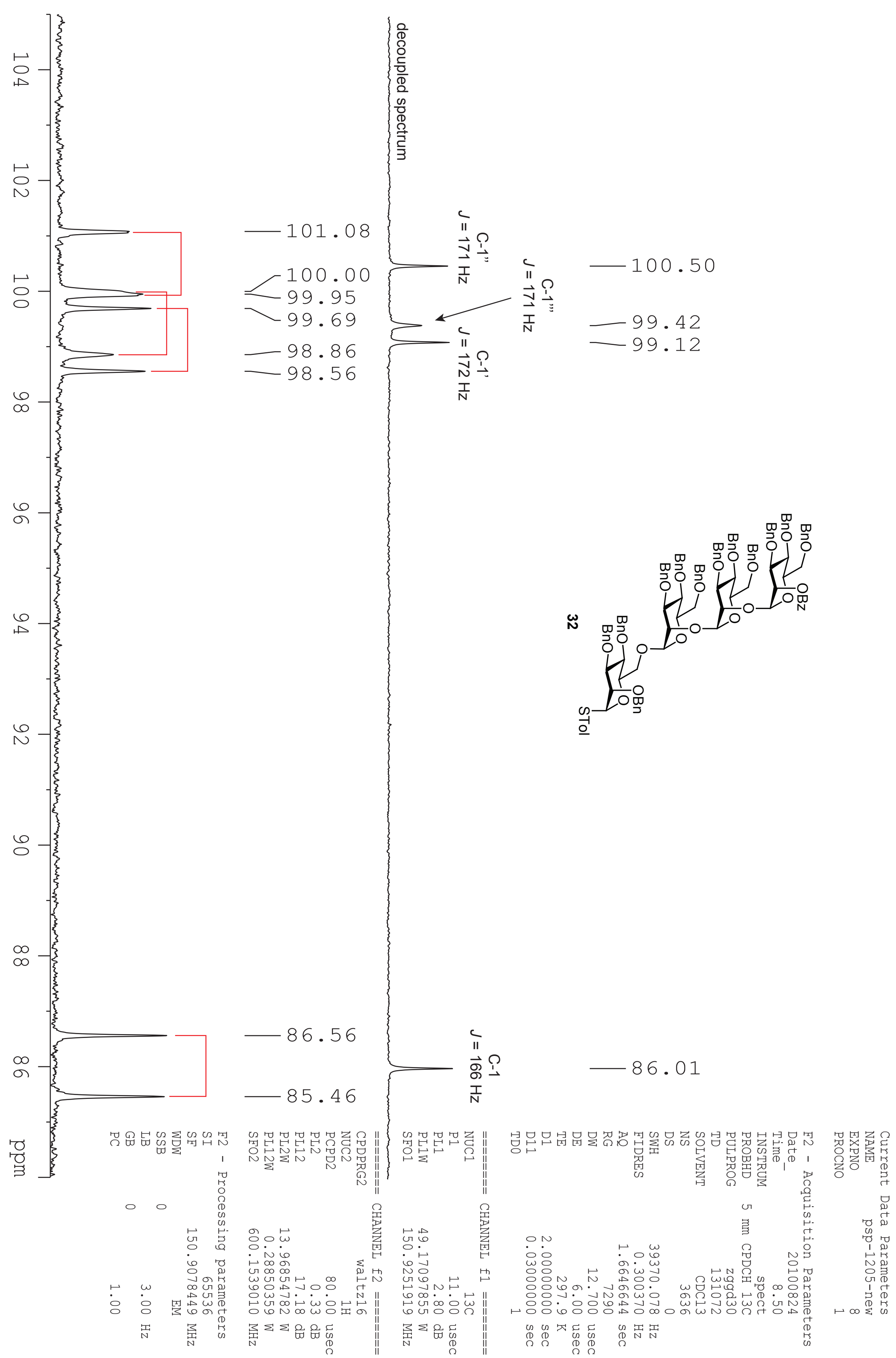

Supplementary Figure 92. Non-decoupled ^{13}C NMR spectrum of compound 32.

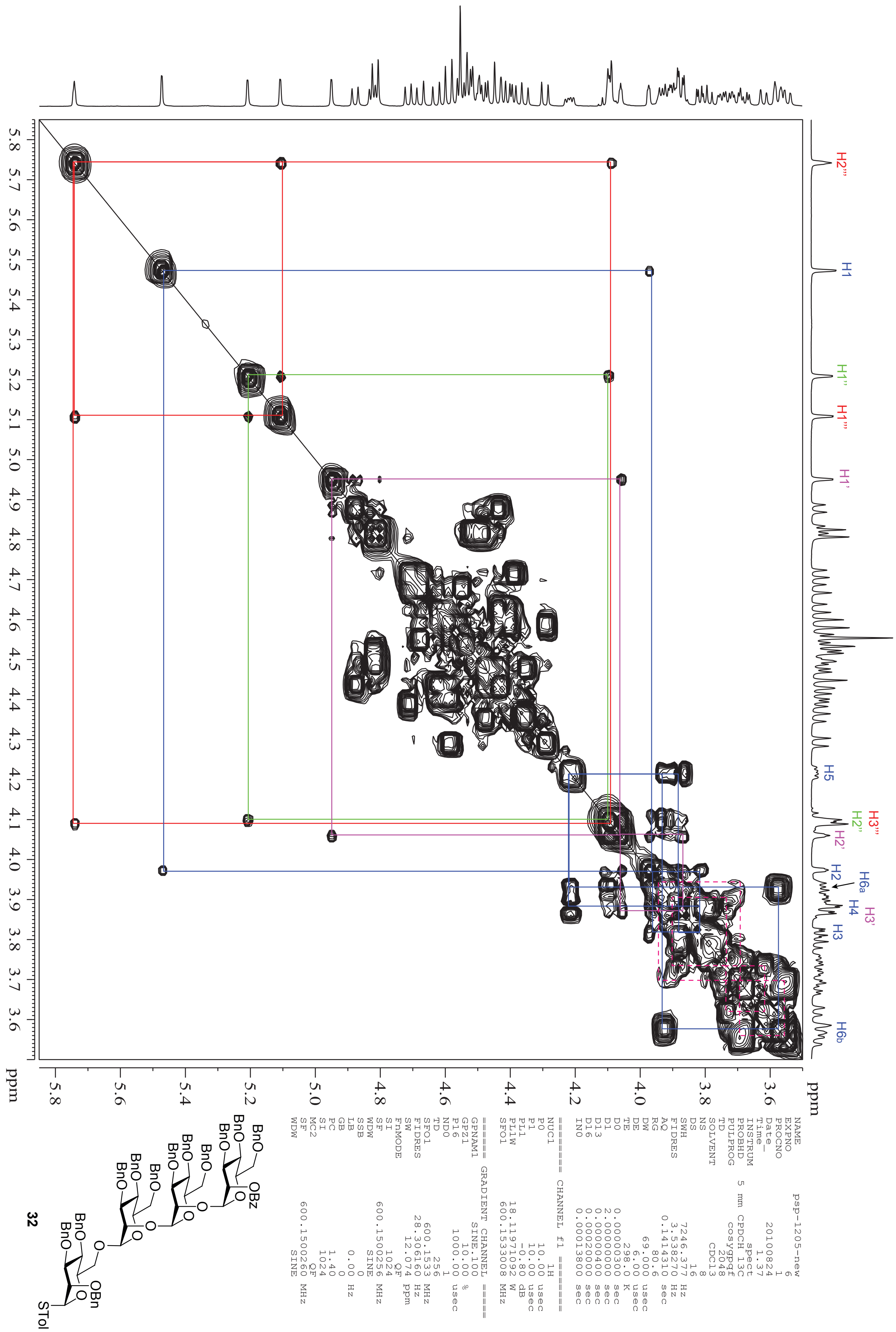

Supplementary Figure 93. COSY NMR spectrum of compound 32.

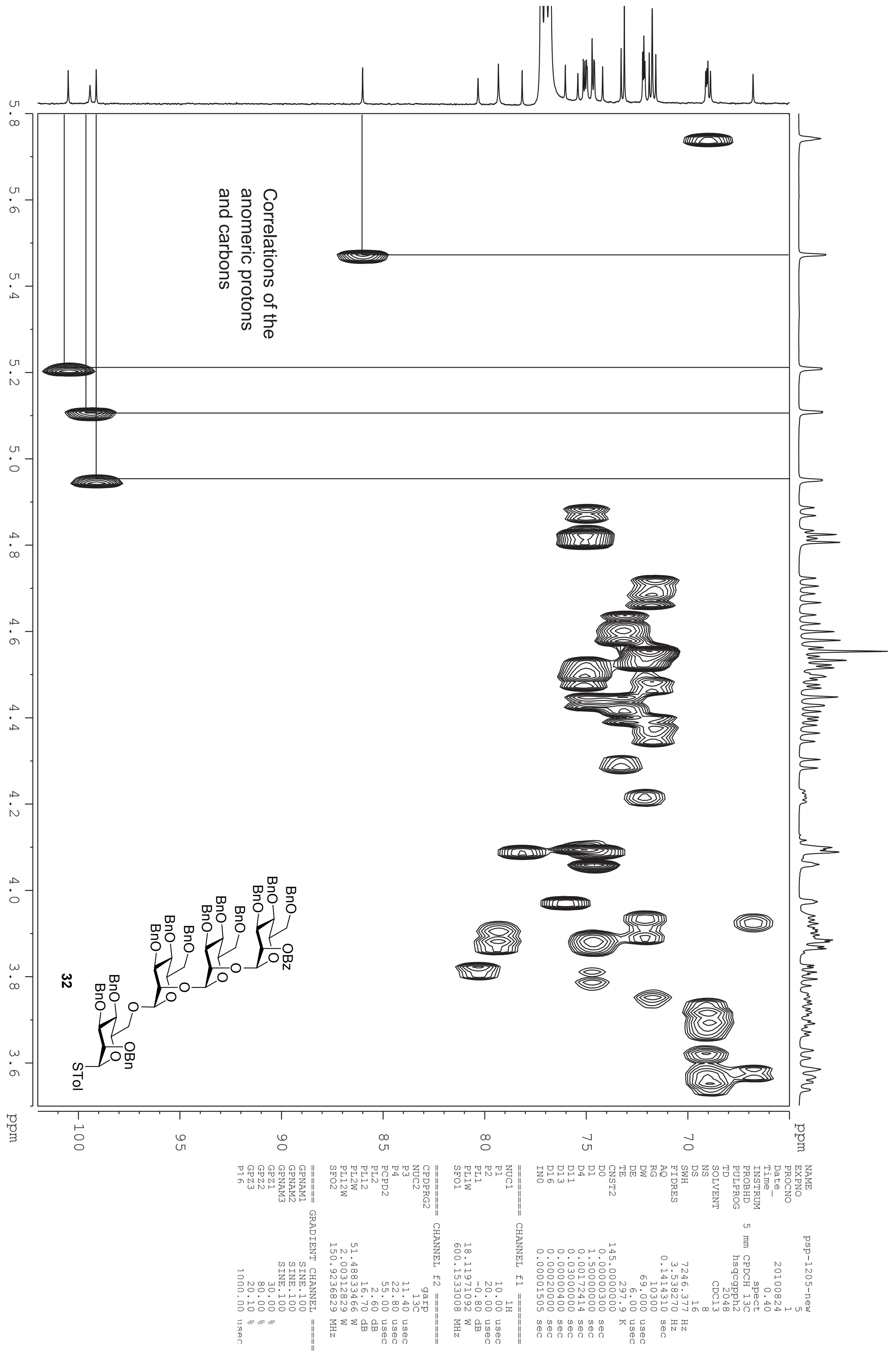

Supplementary Figure 94. HMQC NMR spectrum of compound 32.

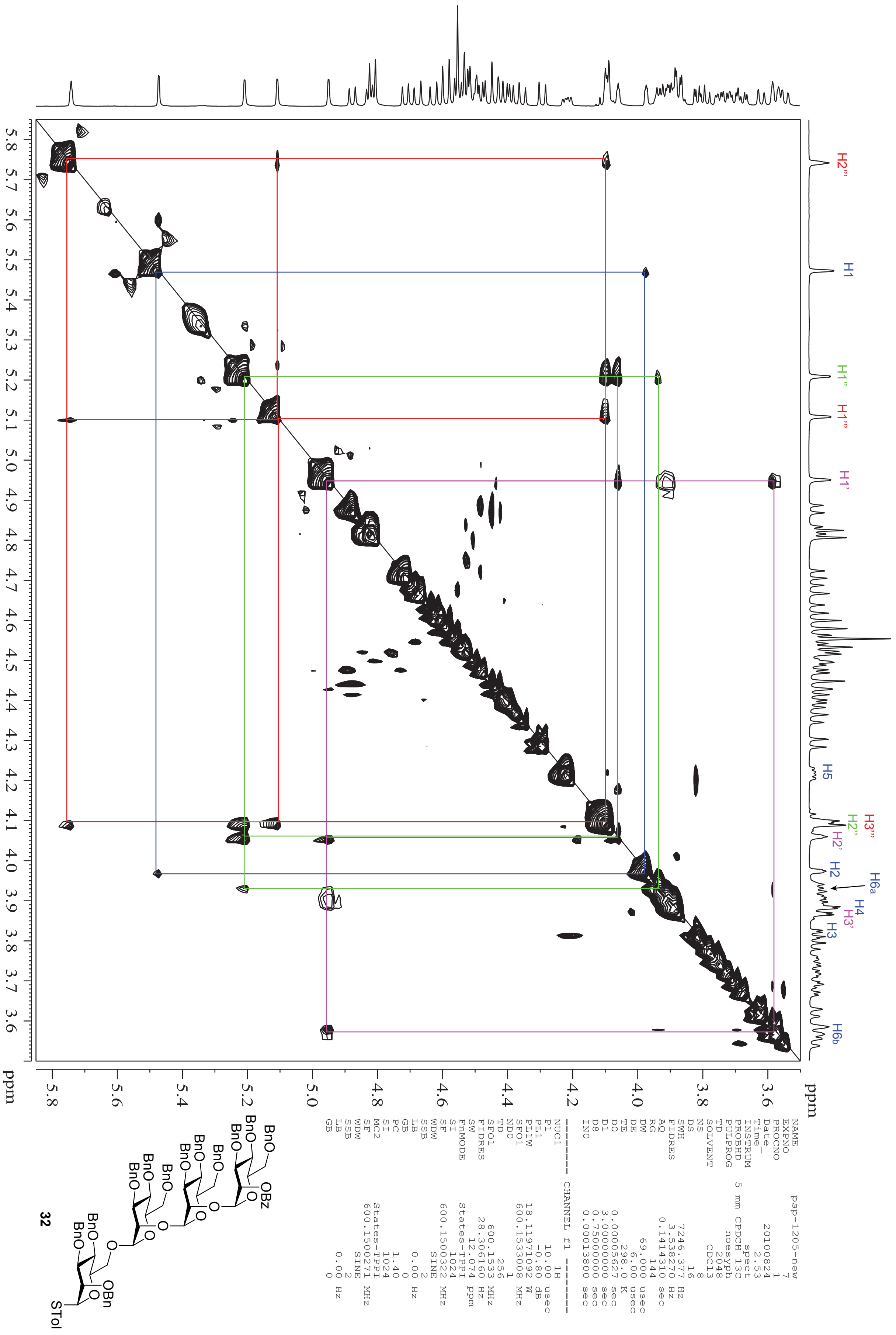

Supplementary Figure 95. NOESY NMR spectrum of compound 32.

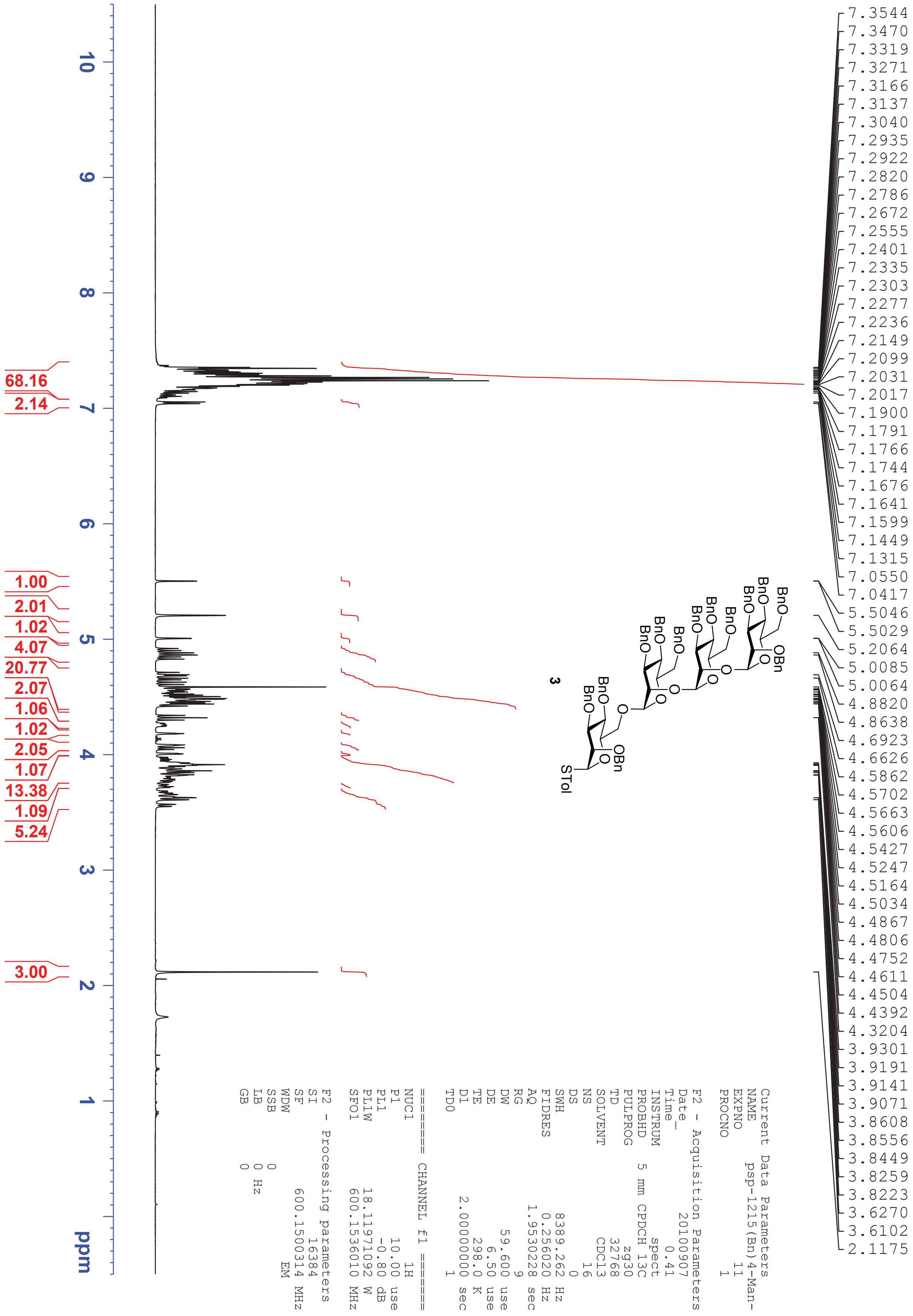

Supplementary Figure 96. ^{1}**H NMR spectrum of compound 3.**

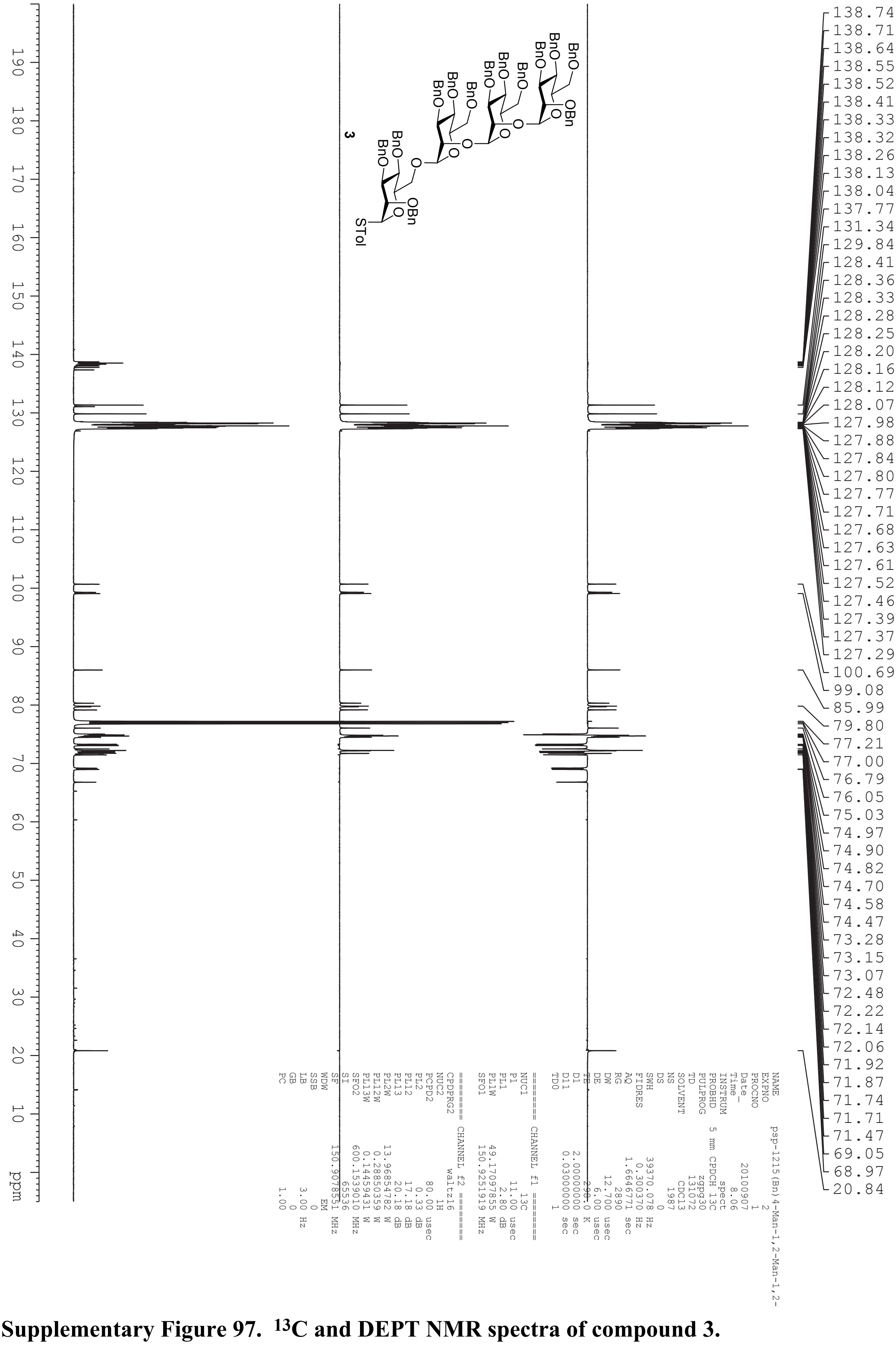

Supplementary Figure 97. ^{13}C and DEPT NMR spectra of compound 3.

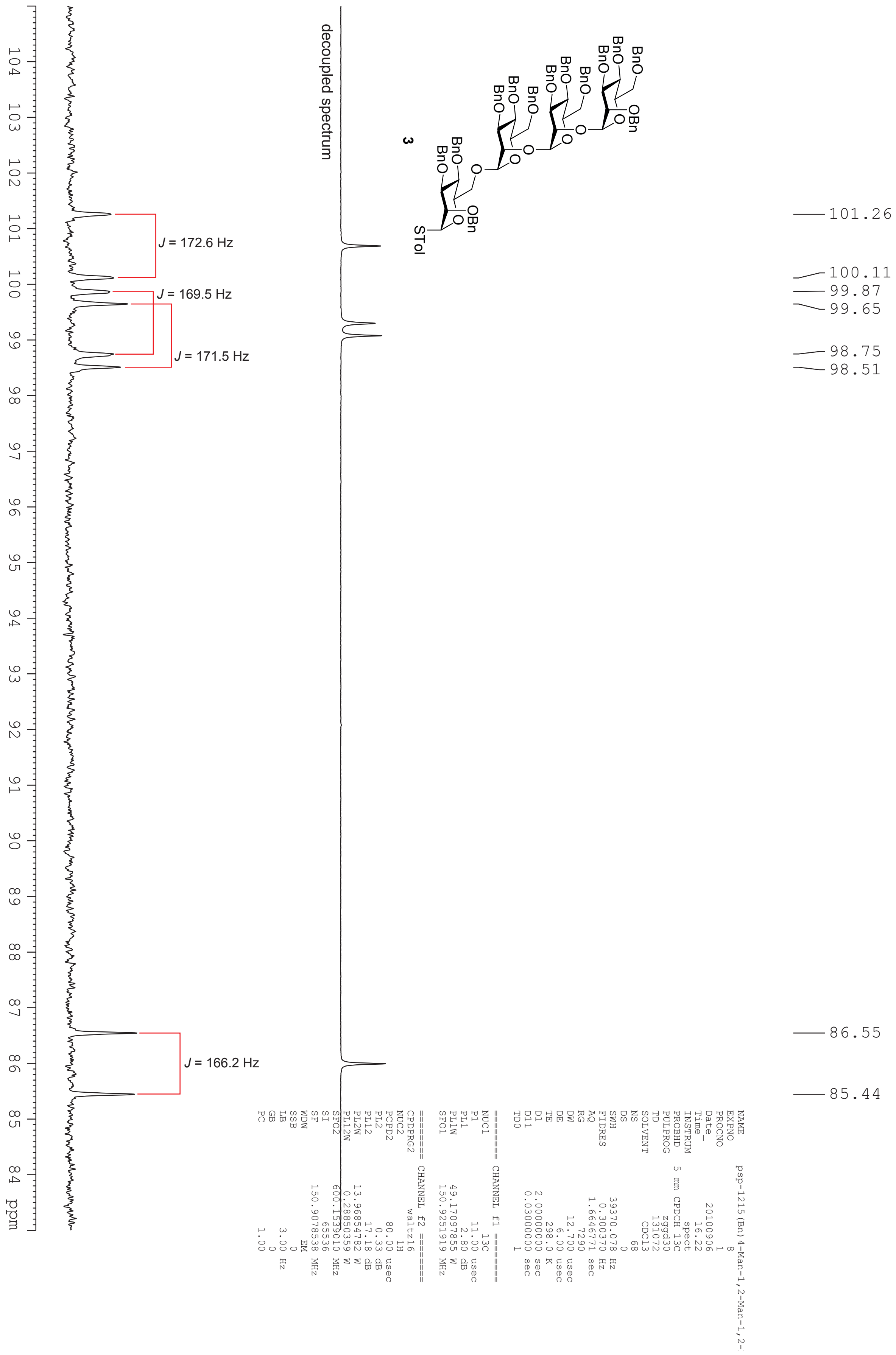

Supplementary Figure 98. Non-decoupled ^{13}C NMR spectrum of compound 3.

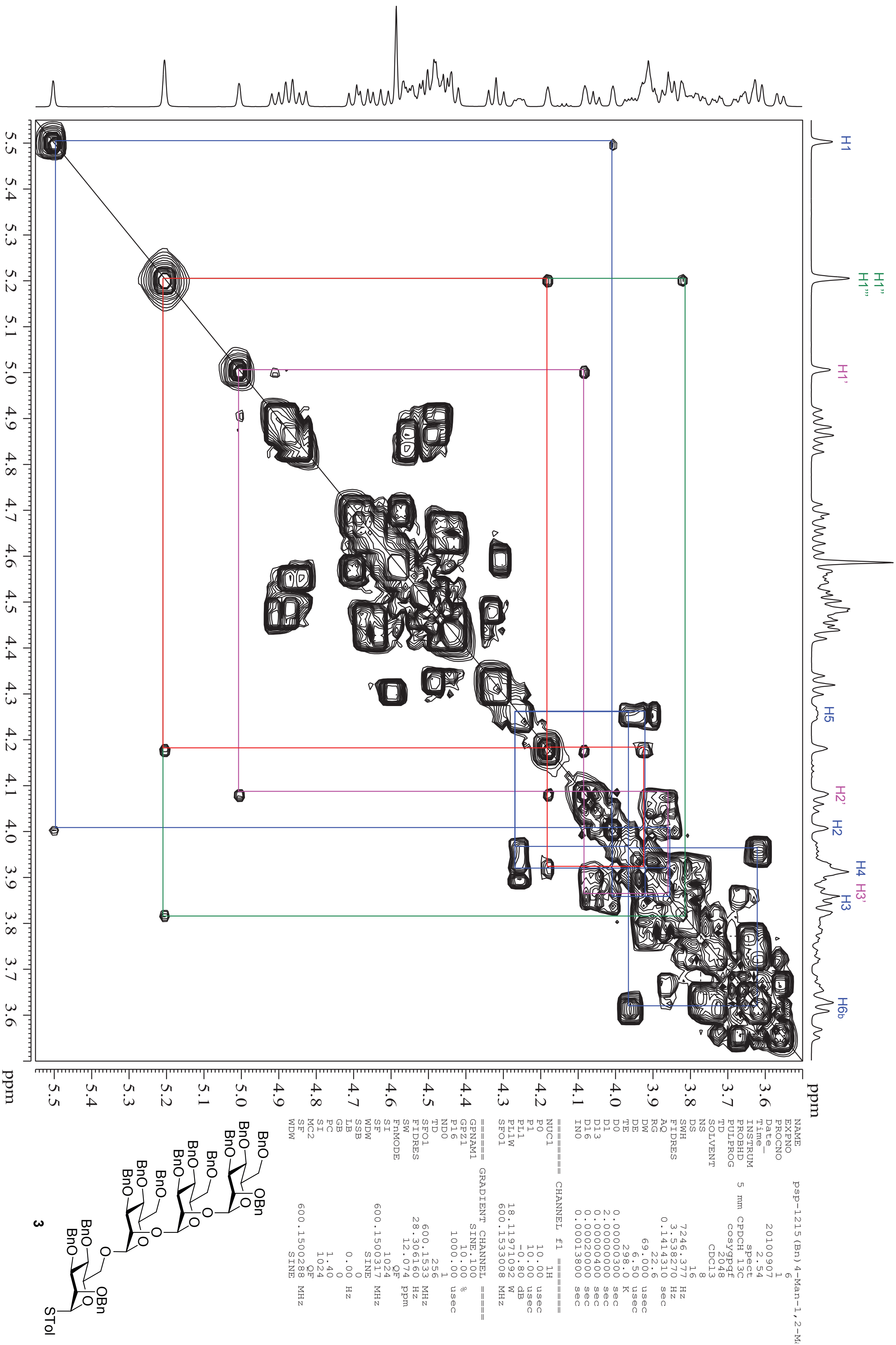

Supplementary Figure 99. COSY NMR spectrum of compound 3.

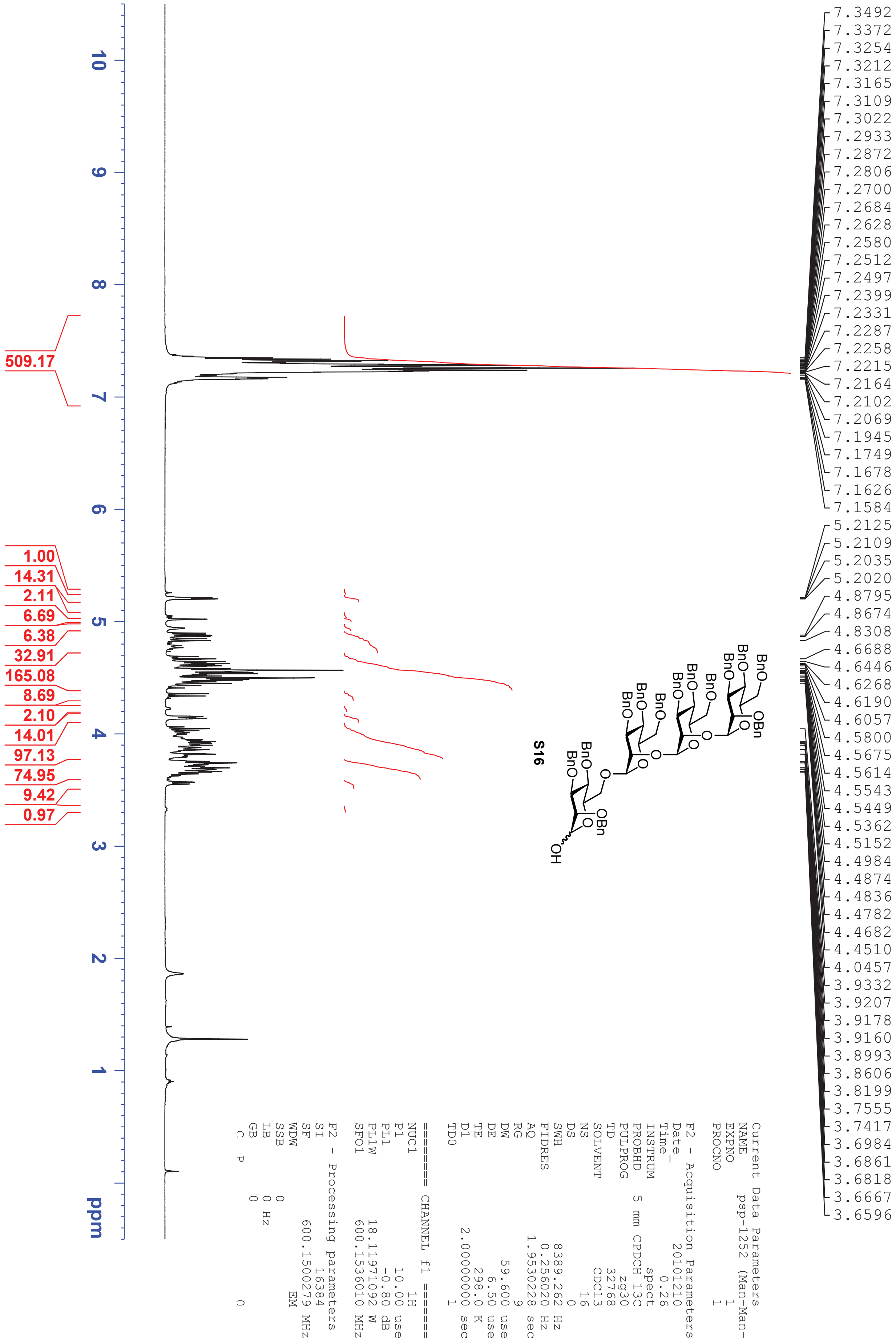

Supplementary Figure 100. ^{1}H NMR spectrum of compound S16.

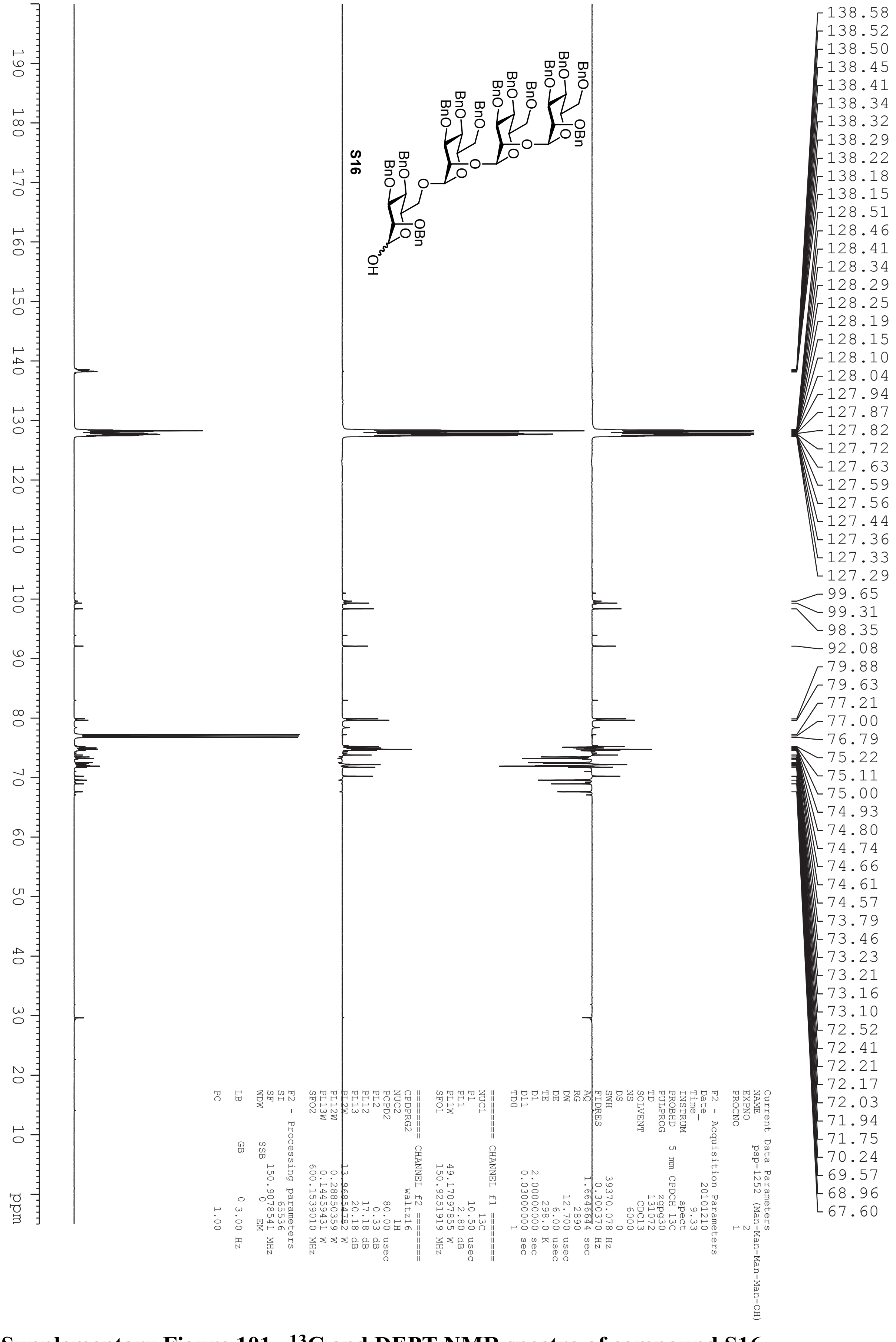

Supplementary Figure 101. ^{13}**C and DEPT NMR spectra of compound S16.**

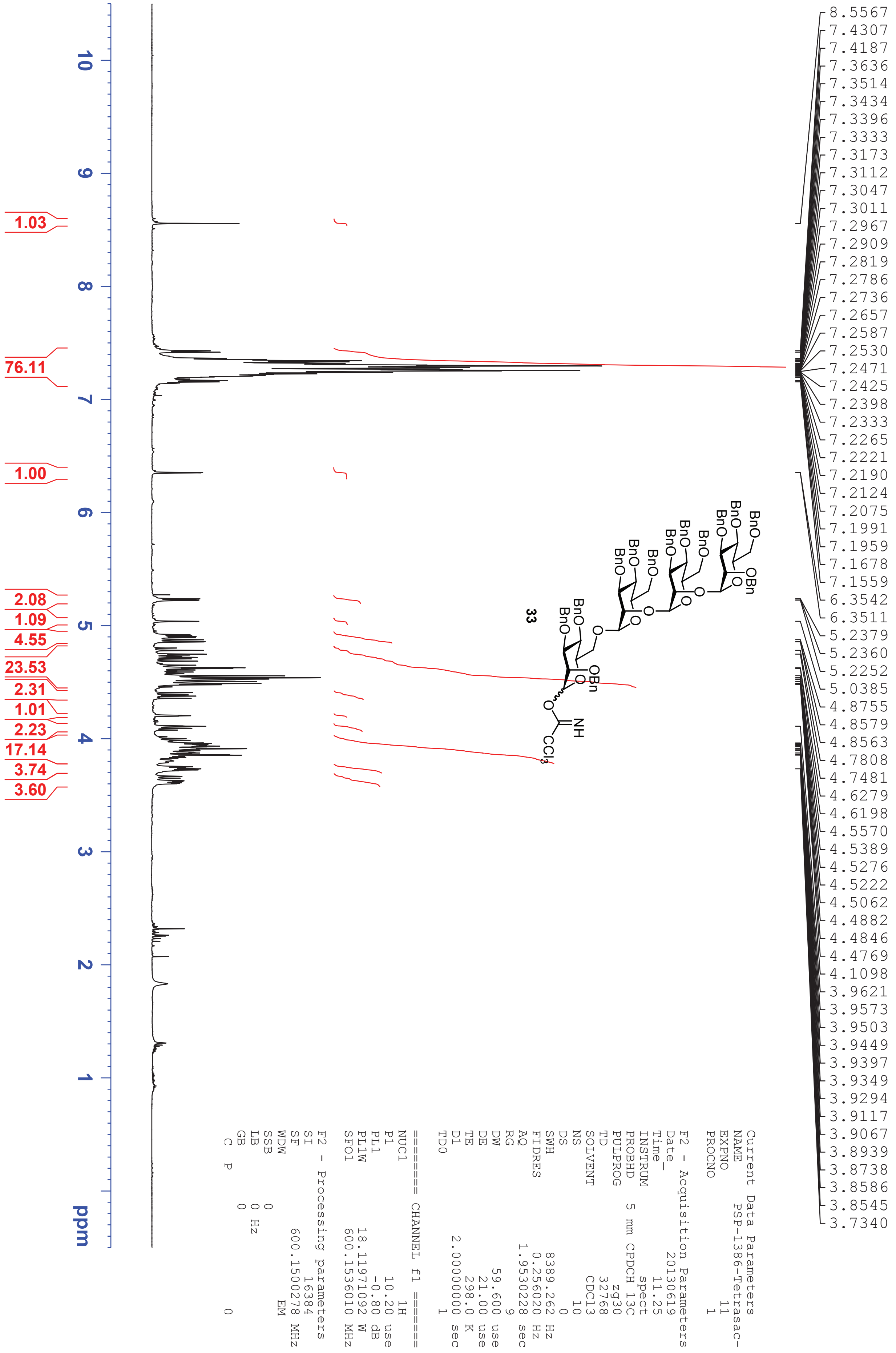

Supplementary Figure 102. **^{1}H NMR spectrum of compound 33.**

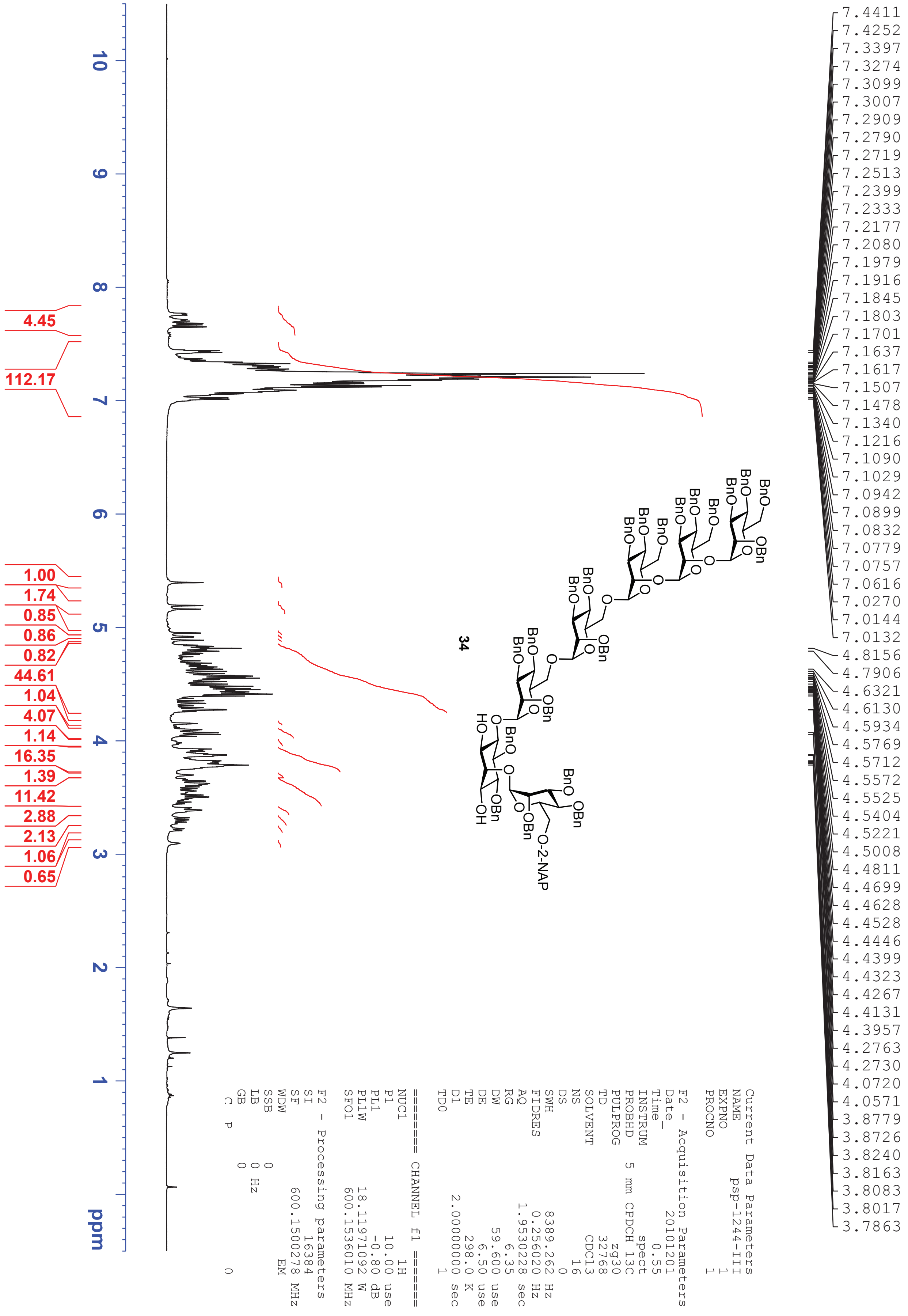

Supplementary Figure 103. ¹H NMR spectrum of compound 34.

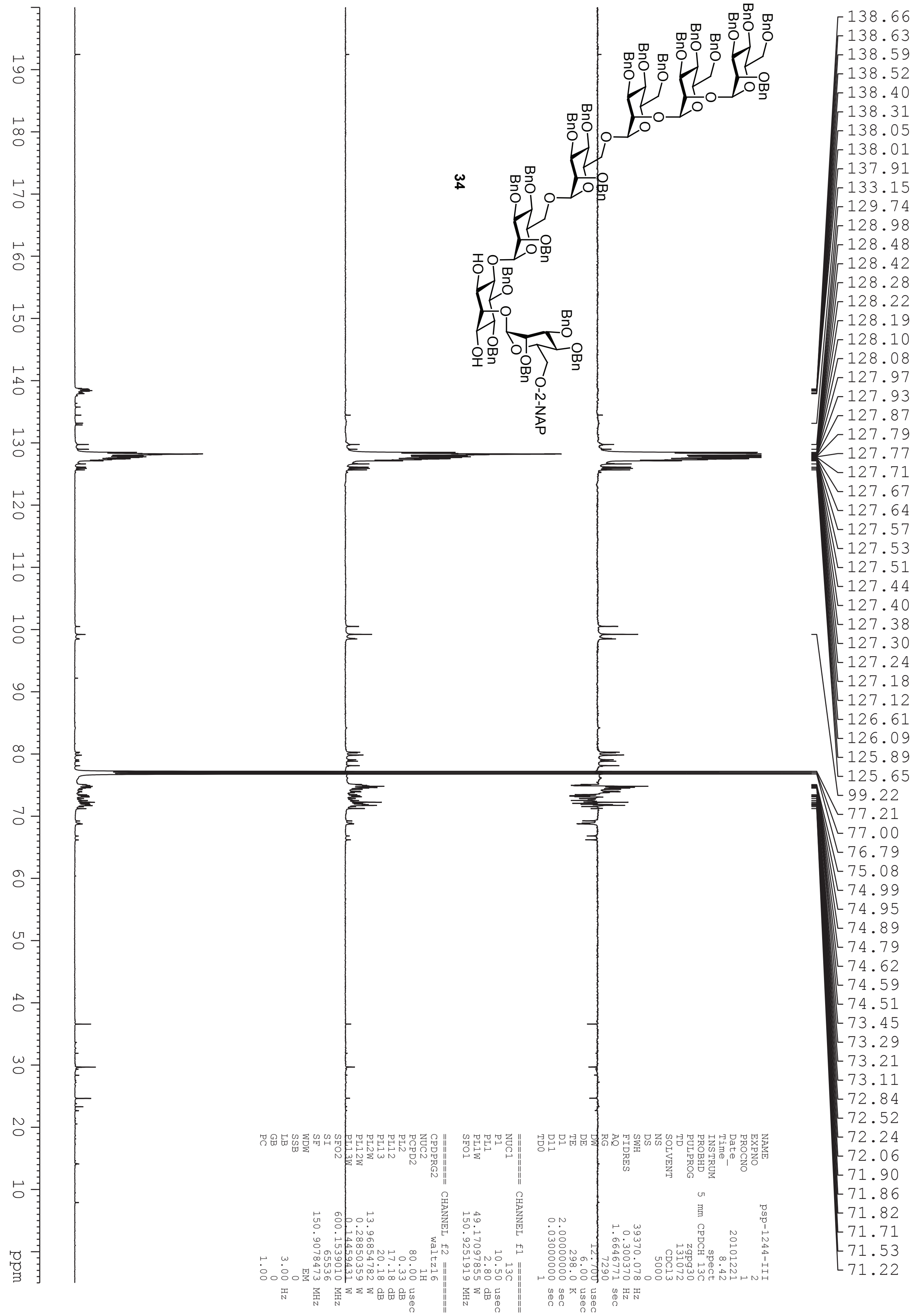

Supplementary Figure 104. ^{13}C and DEPT NMR spectra of compound 34.

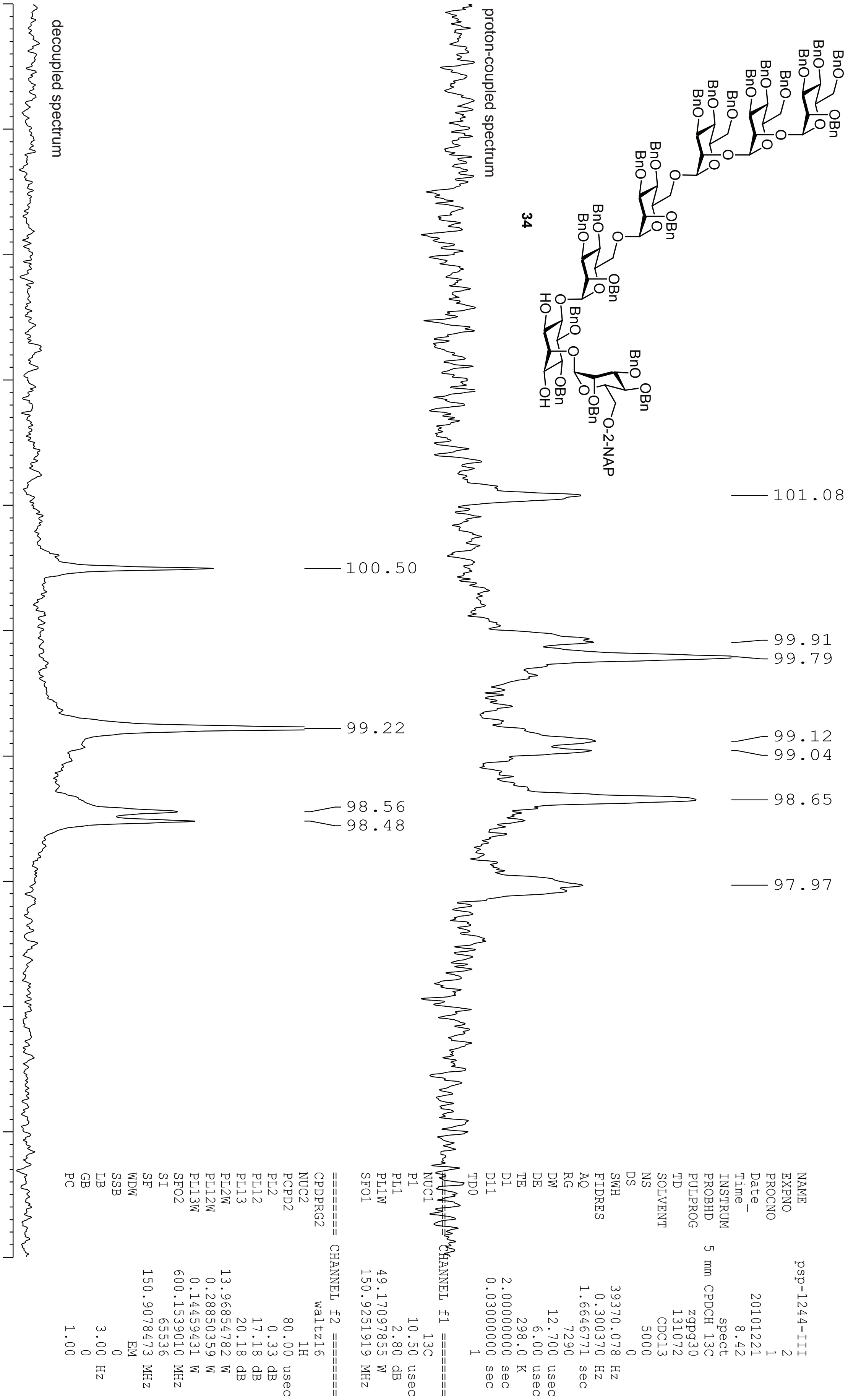

Supplementary Figure 105. Non-decoupled ^{13}C NMR spectrum of compound 34.

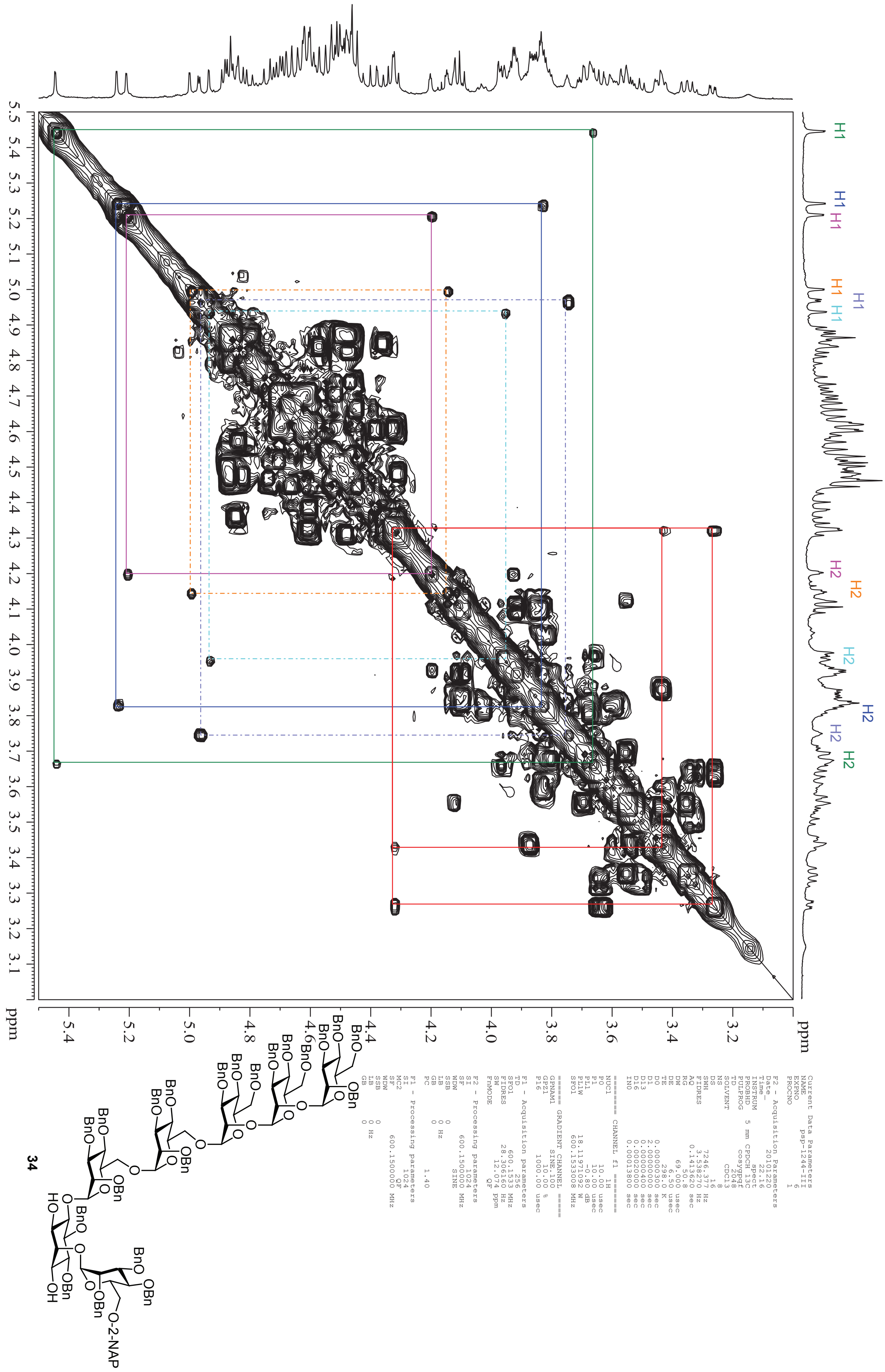

Supplementary Figure 106. COSY NMR spectrum of compound 34.

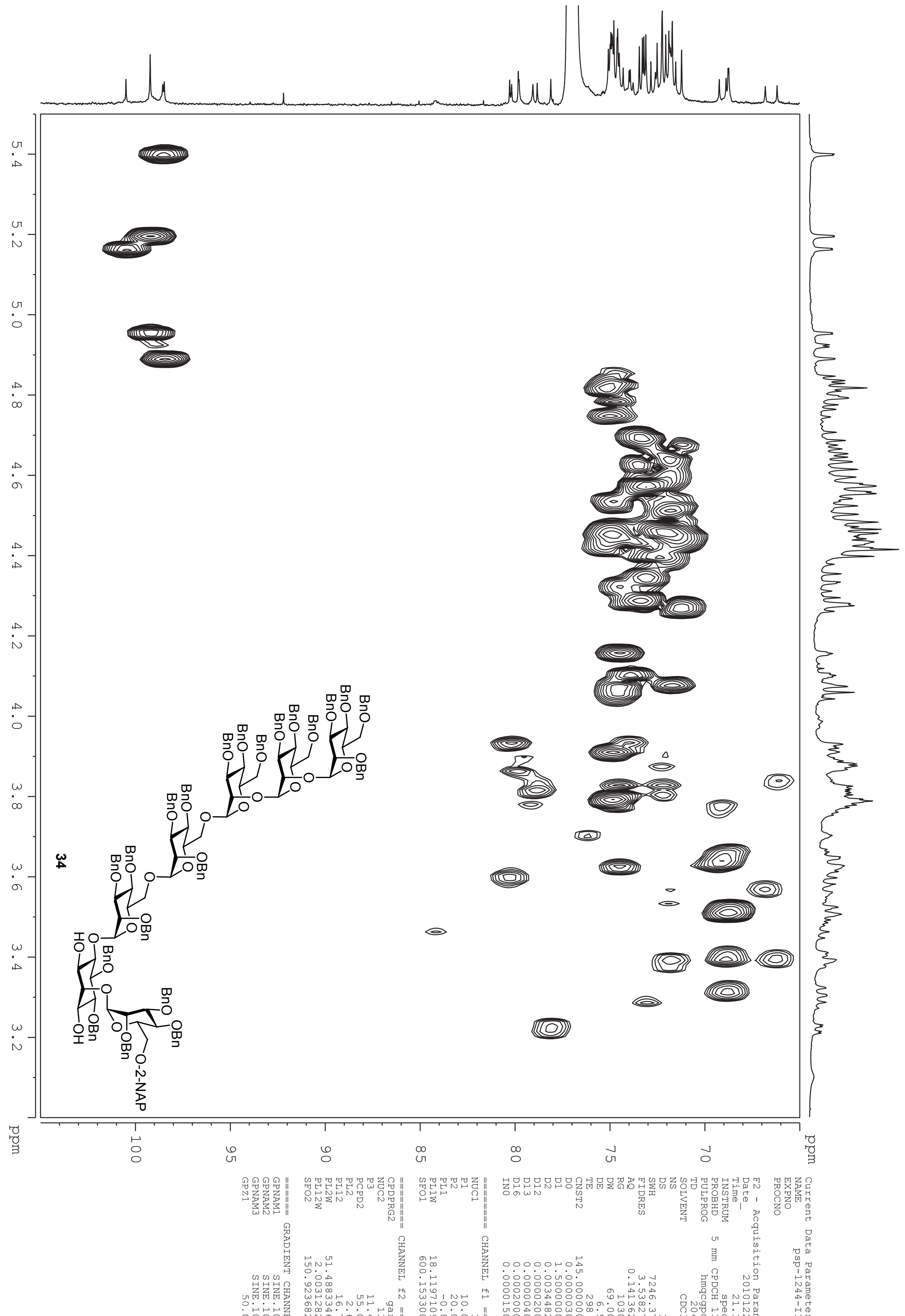

Supplementary Figure 107. HMQC NMR spectrum of compound 34.

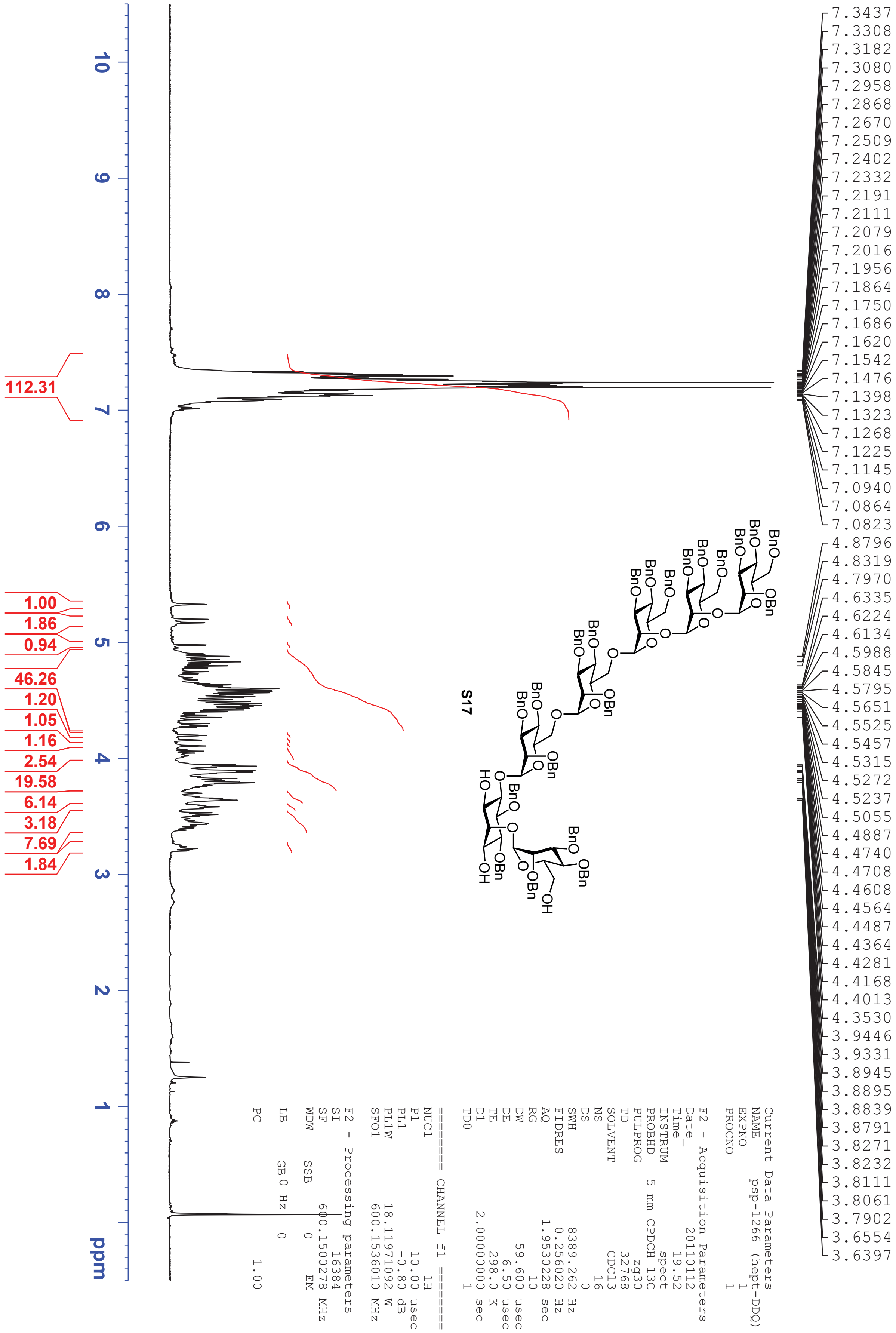

Supplementary Figure 108. ^{1}H NMR spectrum of compound S17.

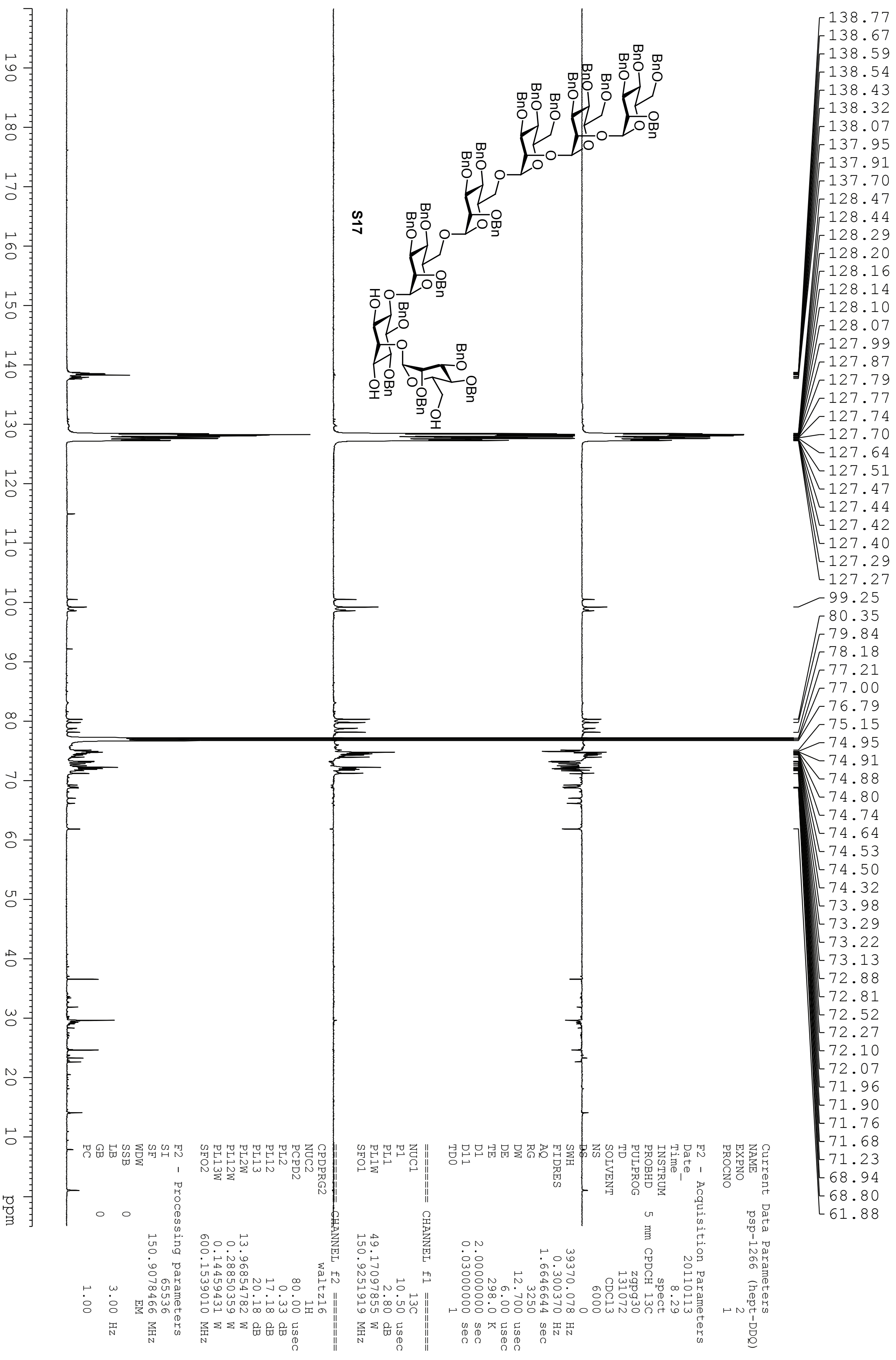

Supplementary Figure 109. ^{13}C and DEPT NMR spectra of compound S17.

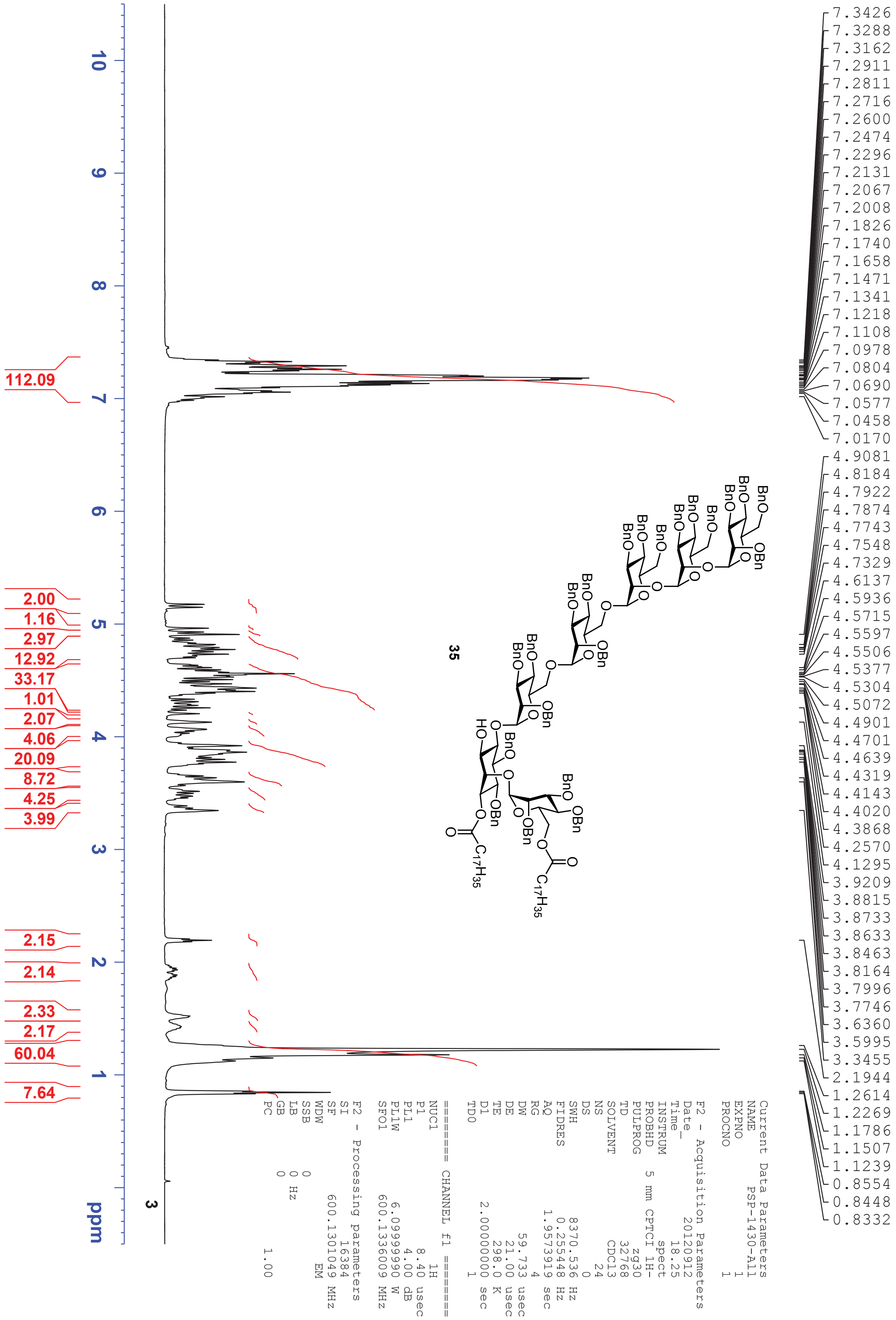

Supplementary Figure 110. ^{1}H NMR spectrum of compound 35.

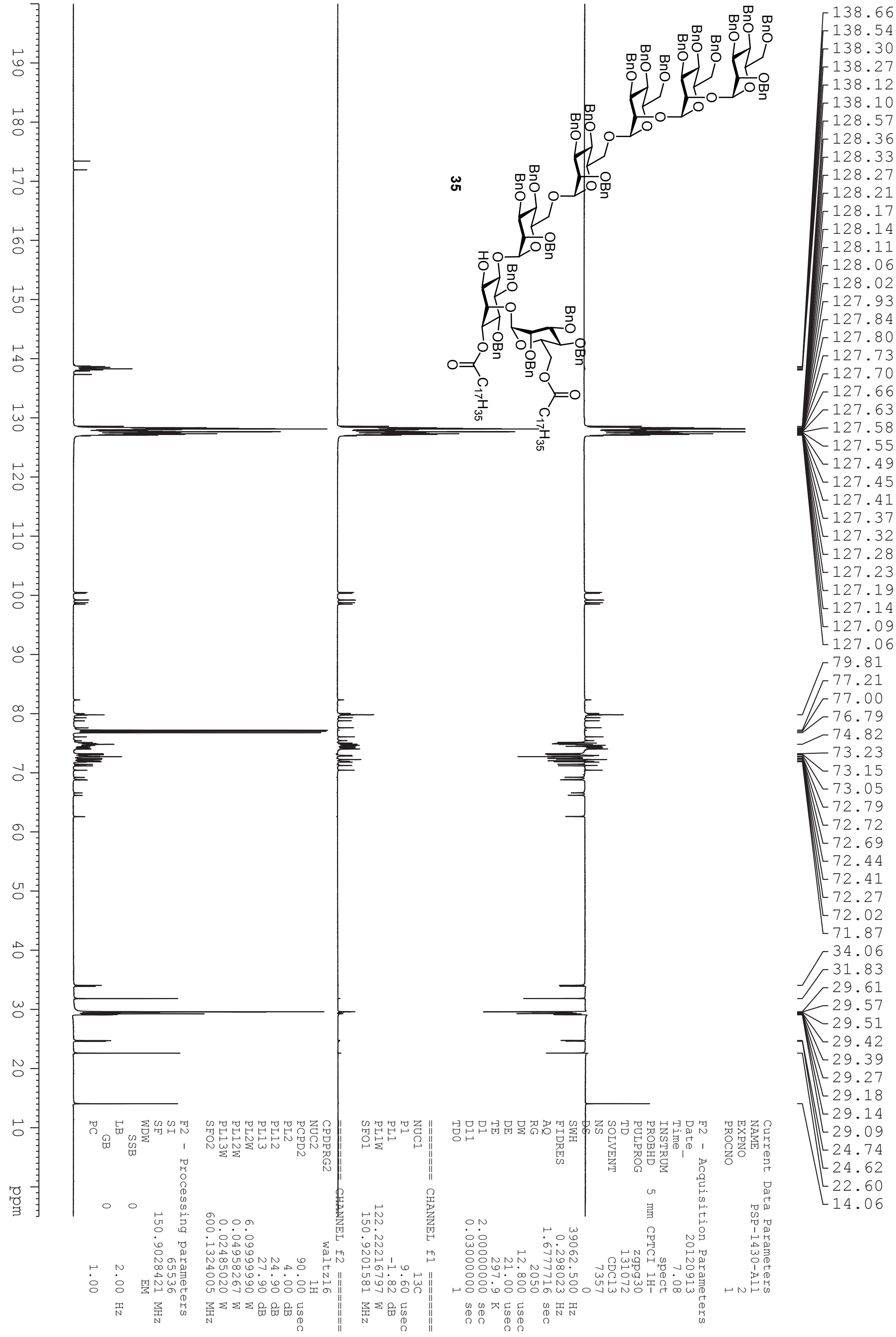

Supplementary Figure 111. ^{13}C and DEPT NMR spectra of compound 35.

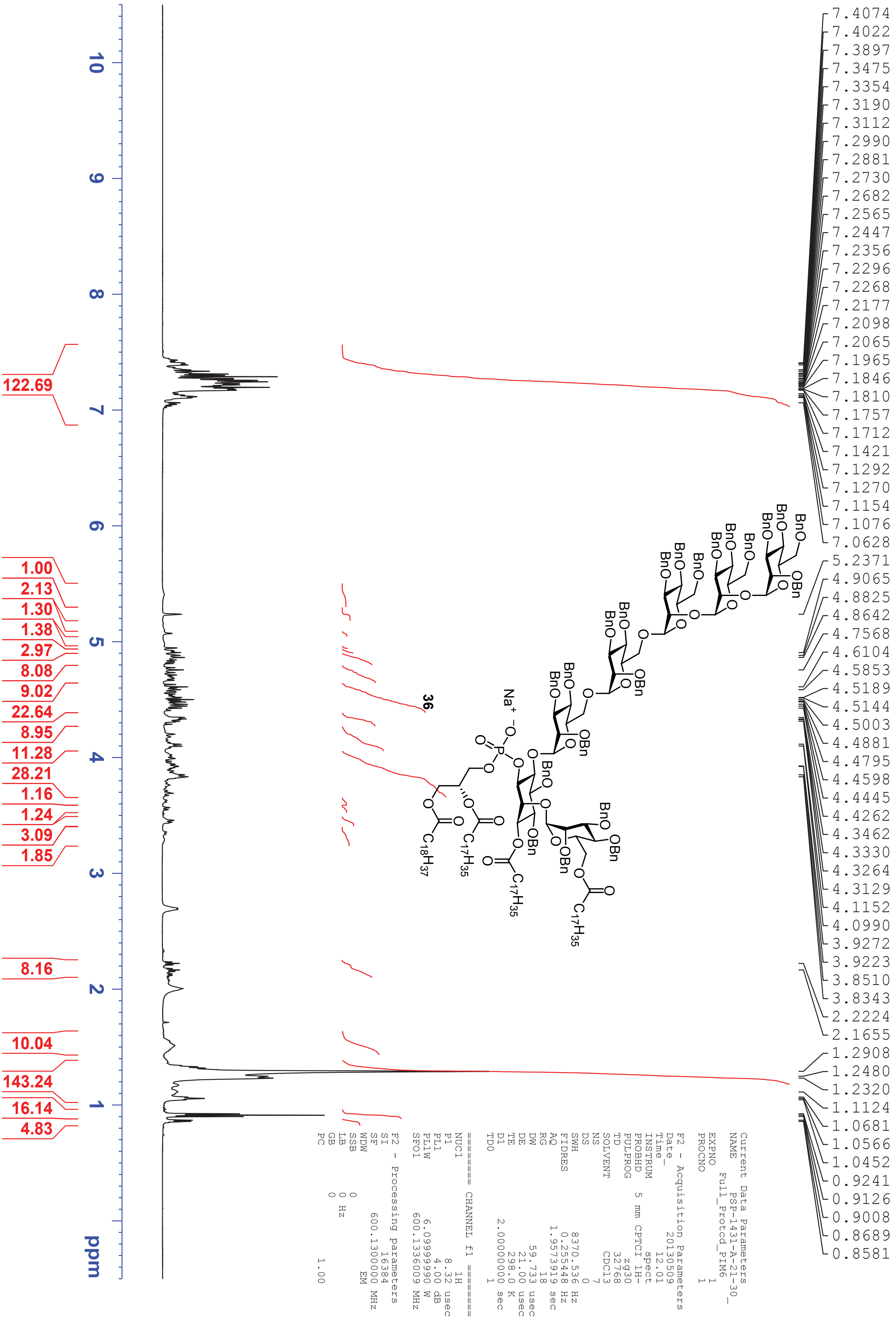

Supplementary Figure 112. ^{1}**H NMR spectrum of compound 36.**

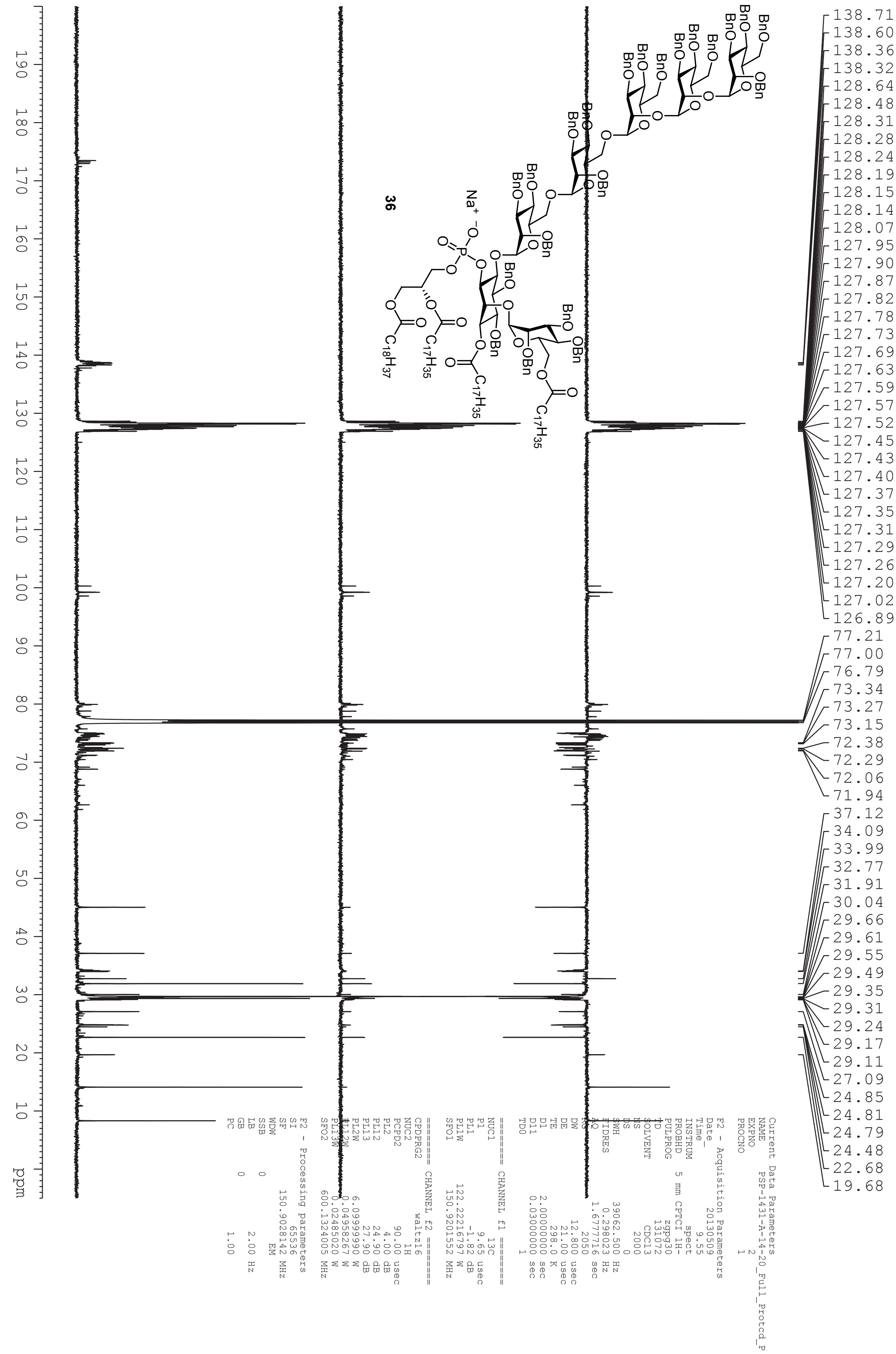

Supplementary Figure 113. ^{13}C and DEPT NMR spectra of compound 36.

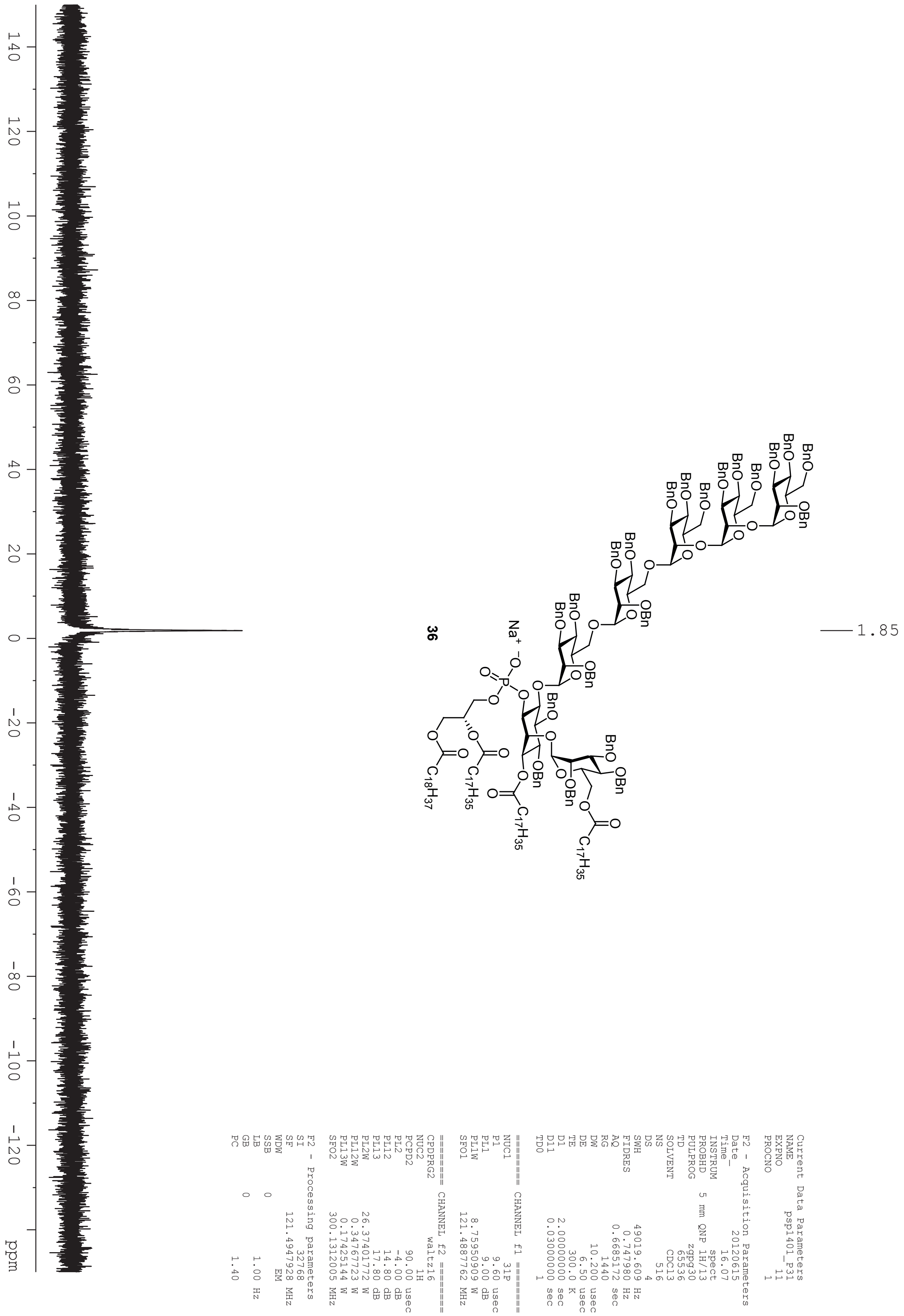

Supplementary Figure 114. ^{31}P NMR spectrum of compound 36.

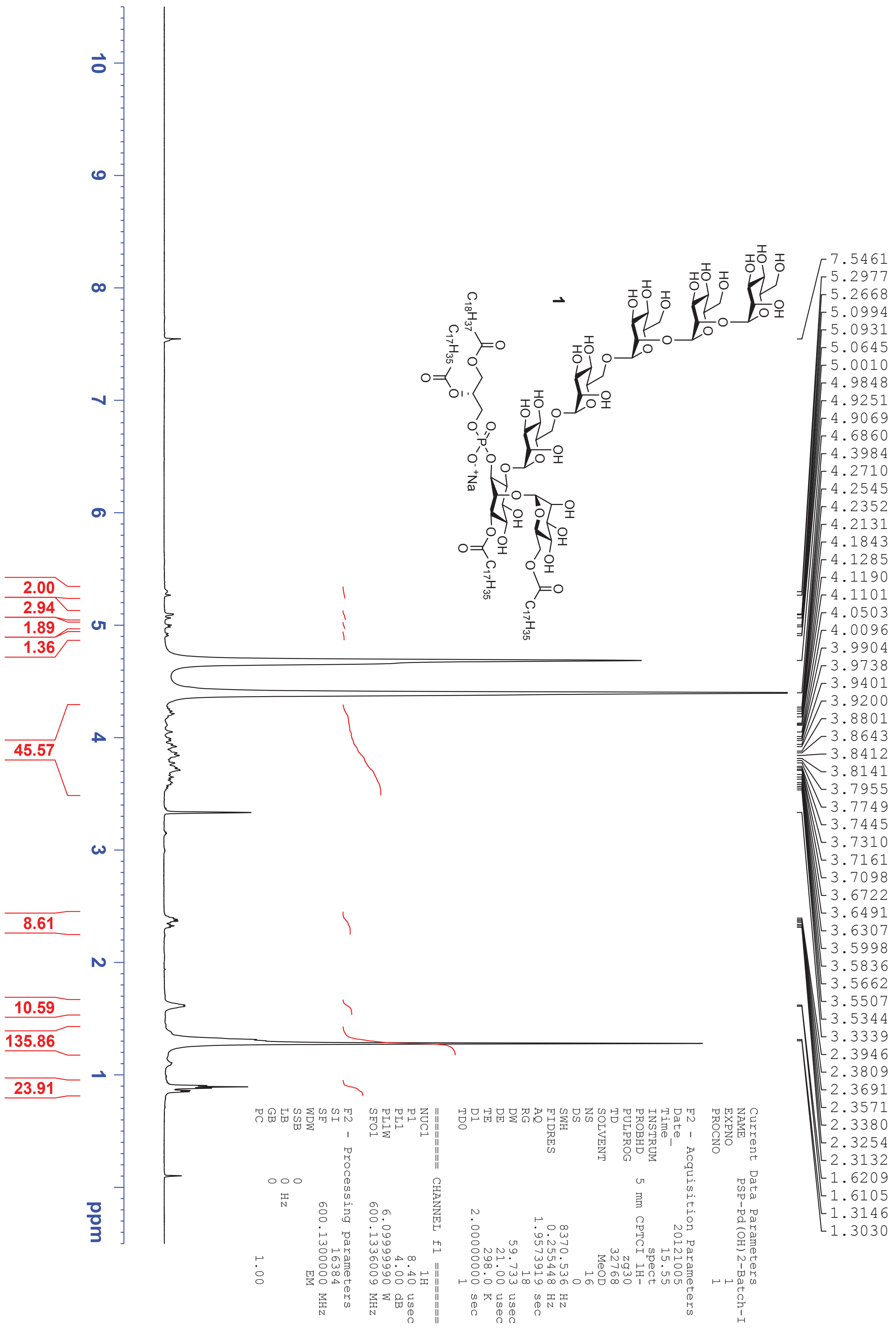

Supplementary Figure 115. ¹H NMR spectrum of compound 1.

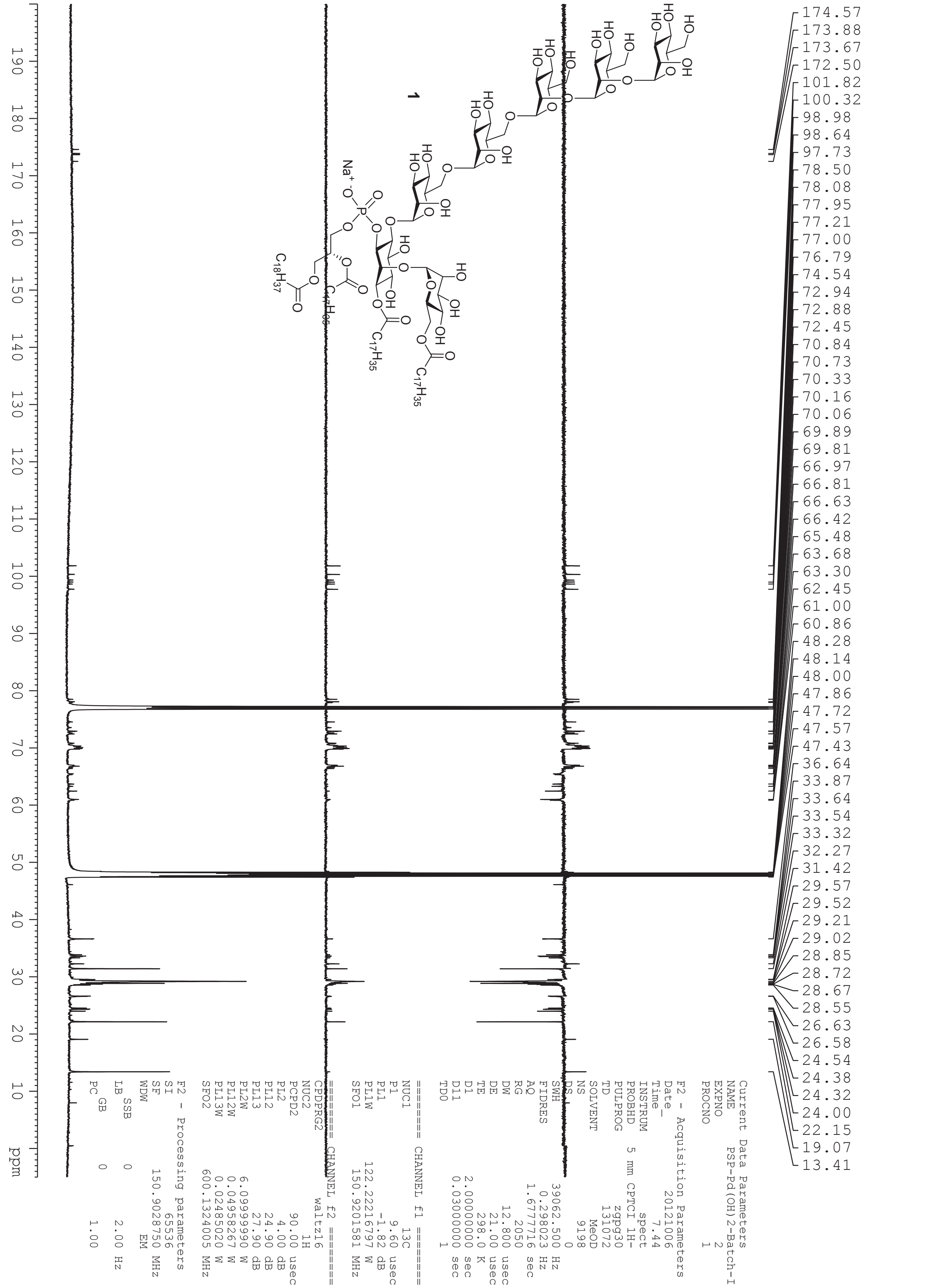

Supplementary Figure 116. ^{13}C and DEPT NMR spectra of compound 1.

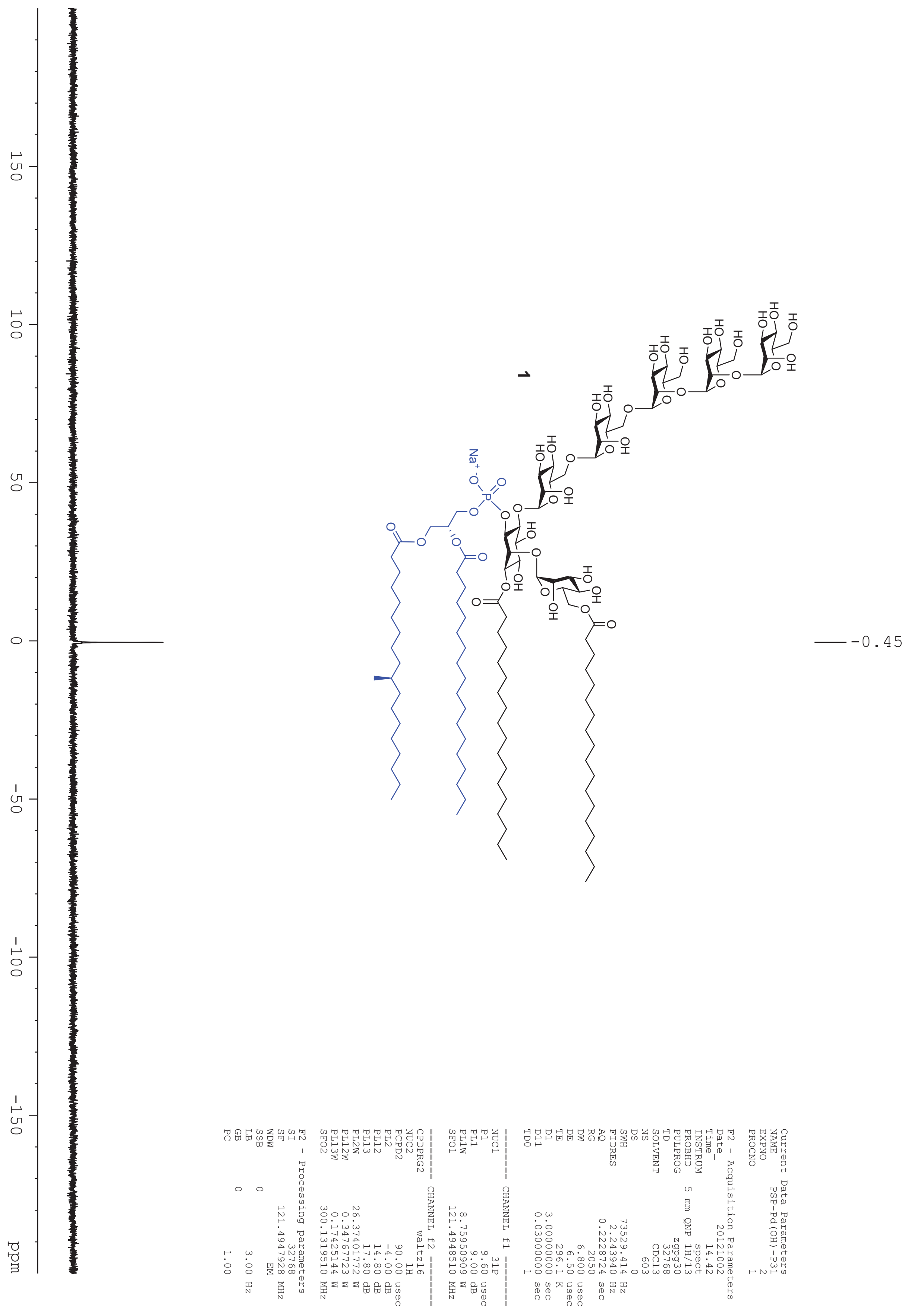

Supplementary Figure 117. ^{31}P NMR spectrum of compound 1.

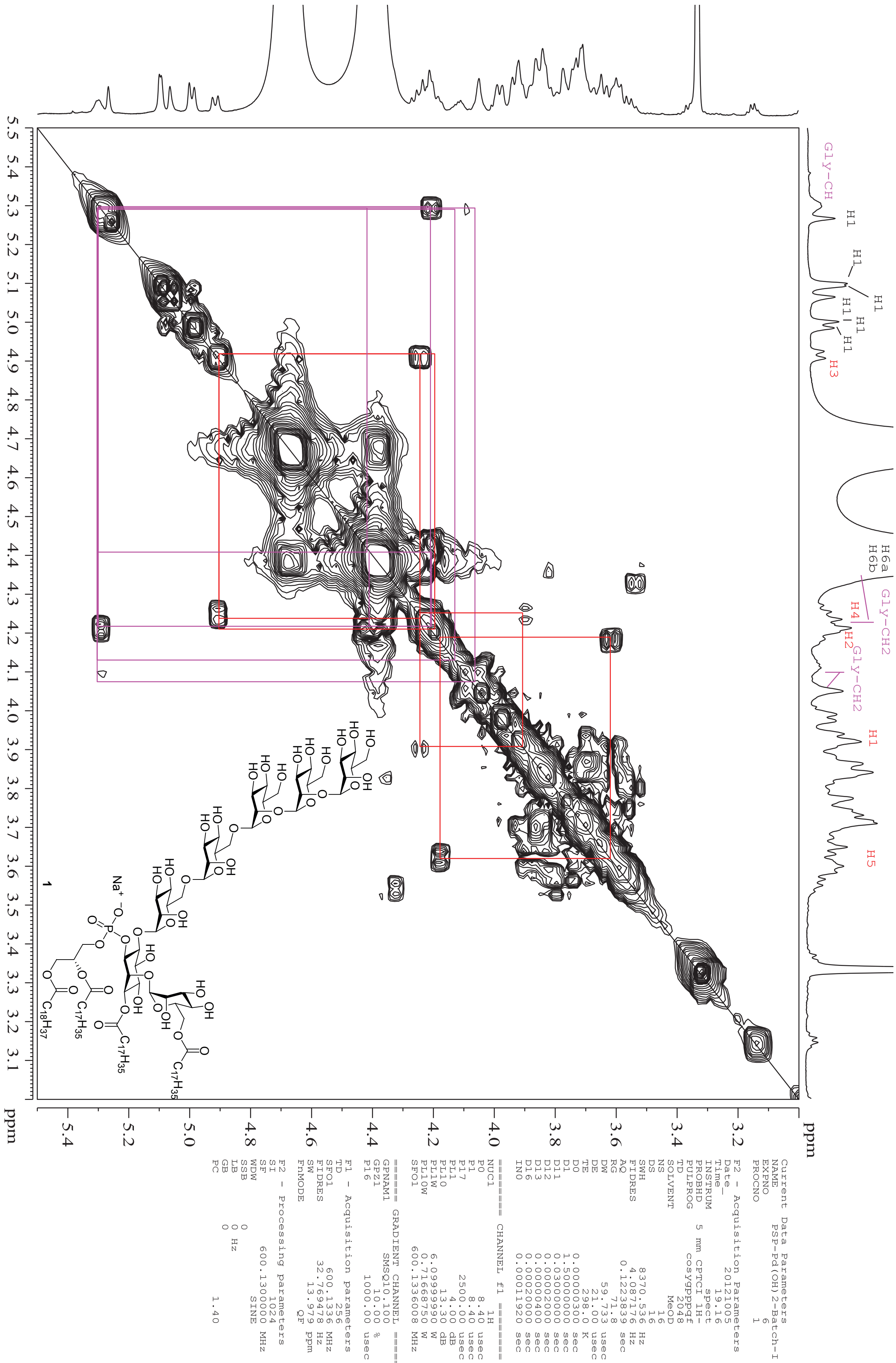

Supplementary Figure 118. COSY NMR spectrum of compound 1.

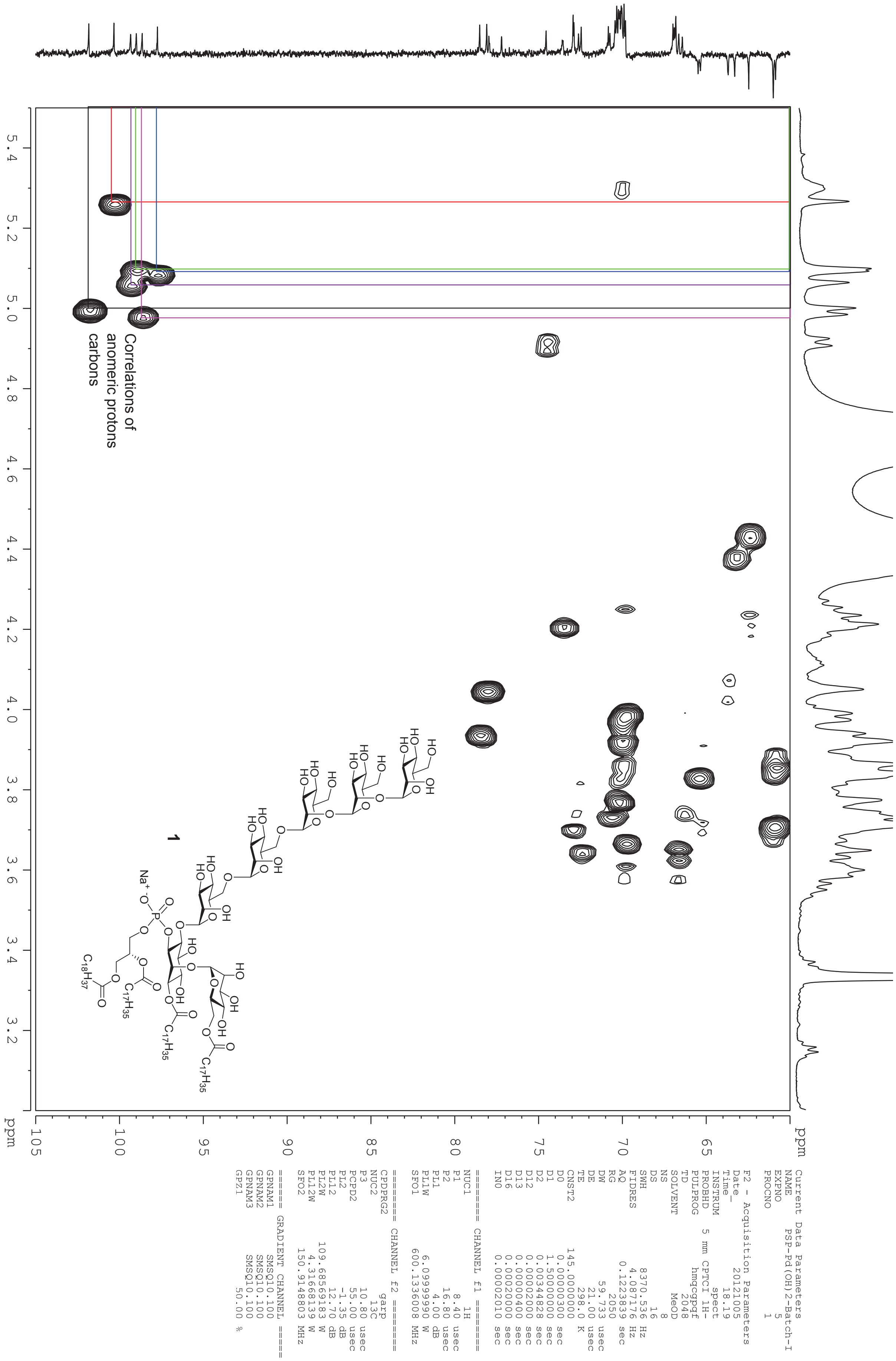

Supplementary Figure 119. HMQC NMR spectrum of compound 1.

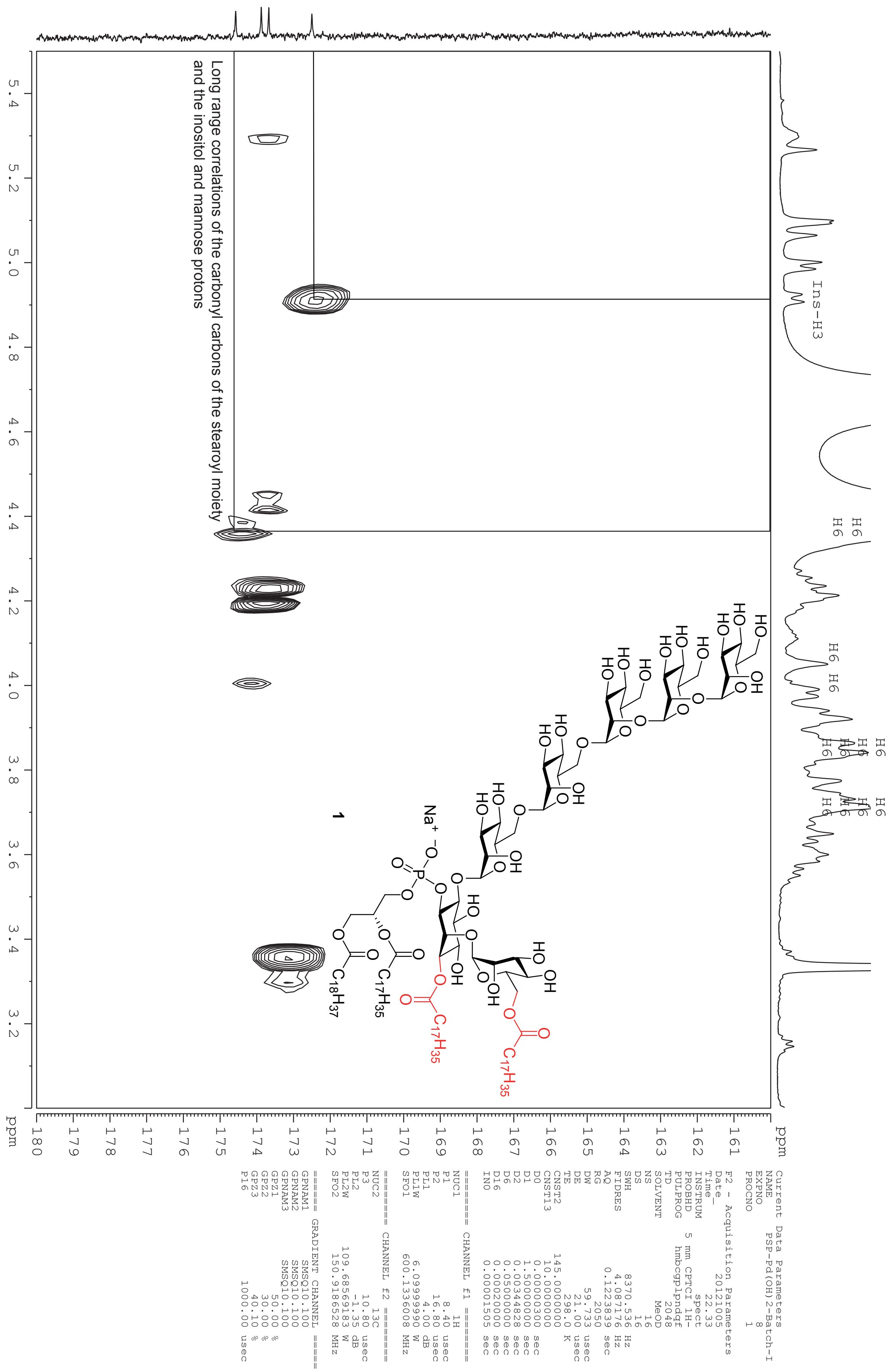

Supplementary Figure 120. HMBC NMR spectrum of compound 1.

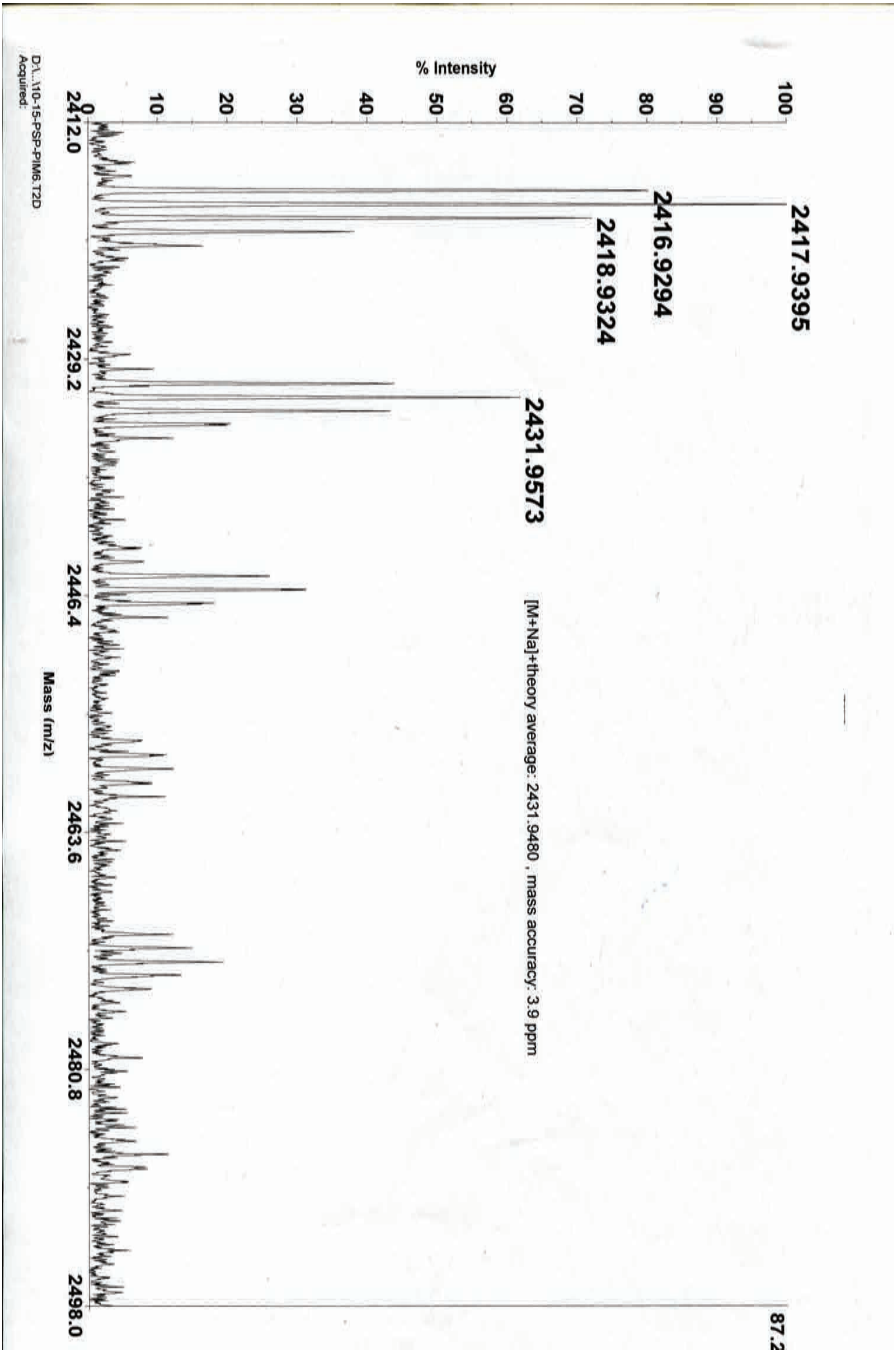

Supplementary Figure 121. Electrospray ionisation mass spectrum of compound 1.

Supplementary Table 1. Desymmetisation of the *myo*-inositol derived diol **16**.

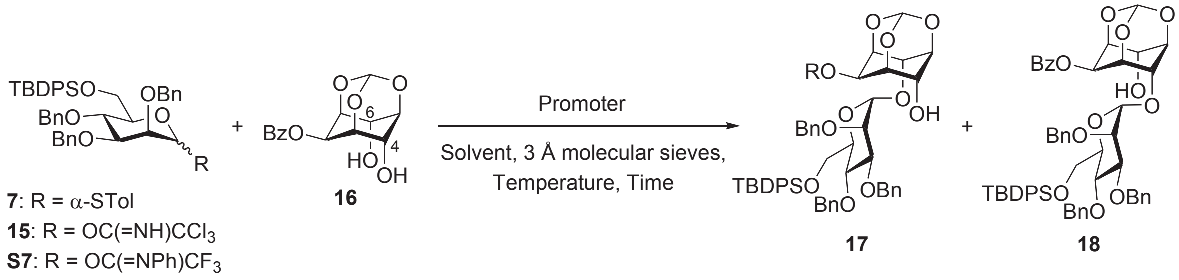

Entry	Donor	Promoter	Solvent	Temperature (°C)	Time (h)	Yield (%) 17	18
1	**7**	NIS/TMSOTf	Dioxane/CH_2Cl_2 (1/3)	–40 to –20	5	0	0
2	**15**	$BF_3 \cdot Et_2O$	Dioxane/CH_2Cl_2 (1/3)	–40 to –20	3	33	11
3	**15**	$BF_3 \cdot Et_2O$	THF	–40 to –20	3	37	14
4	**15**	TMSOTf	Dioxane/CH_2Cl_2 (1/3)	–40 to –20	4	20	9
5	**15**	AgOTf	Dioxane/CH_2Cl_2 (1/3)	–40 to –20	4	37	10
6	**15**	AgOTf	Dioxane/CH_2Cl_2 (1/3)	–78 to –20	5	52	23
7	**15**	AgOTf	Dioxane/CH_2Cl_2 (1/3)	–40	5	54	16
8	**15**	AgOTf	Dioxane/CH_2Cl_2 (10/1)	rt	3	68	20
9	**15**	AgOTf	Dioxane/CH_2Cl_2 (1/3)	0	3	0	0
10	**S3**	AgOTf	Dioxane/CH_2Cl_2 (10/1)	rt	8	8	0
11	**S7**	$BF_3 \cdot Et_2O$	Dioxane/CH_2Cl_2 (10/1)	rt	8	8	0

THF, tetrahydrofuran.

Supplementary Table 2. Preparation of the pseudoheptasaccharide **34**.

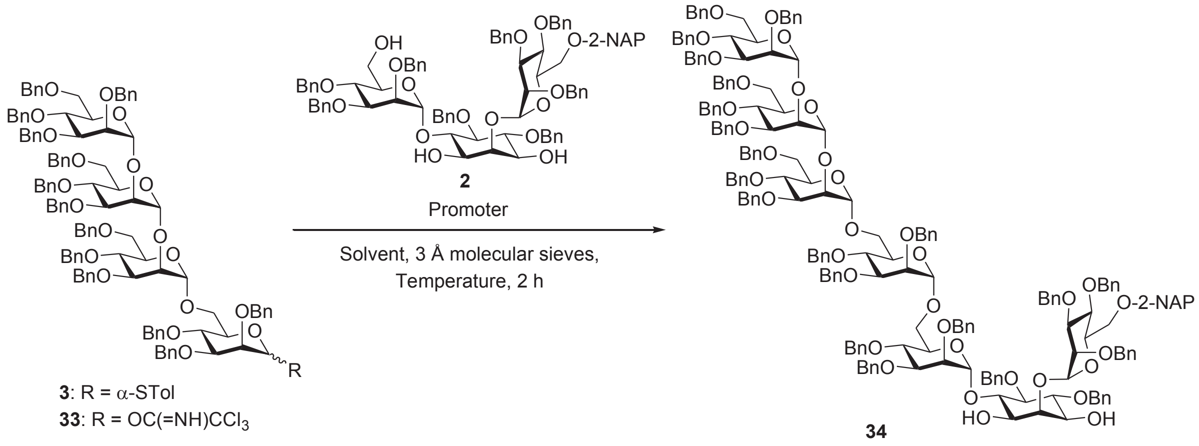

Entry	Donor	Promoter	Solvent	Temperature (°C)	Yield (%)
1	**3**	NIS/TMSOTf	CH_2Cl_2	–78	0
2	**3**	NIS/TMSOTf	CH_2Cl_2	–60	10
3	**3**	NIS/TMSOTf	CH_2Cl_2	–40 to –20	–[a]
4	**33**	TfOH	CH_2Cl_2	–60	0
5	**33**	AgOTf	CH_2Cl_2	0	0
6	**33**	TMSOTf	CH_2Cl_2	–60	24
7	**33**	TMSOTf	CH_2Cl_2	–40	40
8	**33**	TMSOTf	Et_2O	–40	52 (89[b])

[a]Mixture of inseparable products was obtained. [b]Yield is based on the recovered acceptor **2**. TfOH, trifluoromethanesulfonic acid.

Supplementary Methods

I. General methods

CH_2Cl_2, Et_2O and tetrahydrofuran (THF) were purified and dried from a safe purification system. Anhydrous 1,4-dioxane, and pyridine were purchased from Aldrich and directly used for the reactions. Flash column chromatography was carried out on Silica Gel 60 (230–400 mesh, E. Merk). TLC was performed on pre-coated glass plates of Silica Gel 60 F_{254} (0.25 mm, E. Merck); detection was executed by spraying with a solution of $Ce(NH_4)_2(NO_3)_6$, $(NH_4)_6Mo_7O_{24}$, and H_2SO_4 in water and subsequent heating on a hot plate. Specific rotations were taken at ambient conditions and reported in $10^{-1}\cdot deg\cdot cm^2\cdot g^{-1}$; the sample concentrations are in $g\cdot dL^{-1}$. ^1H, ^{13}C and ^{31}P NMR spectra were recorded on 400 and 600 MHz spectrometers. Chemical shifts are in ppm from Me_4Si, calibrated using the residual proton and carbon of the deuterated solvent. Proton peak assignments were performed using two-dimensional NMR techniques (^1H-^1H COSY, HMQC and NOESY). The hydrogen multiplicities of carbon peaks were determined using DEPT-90 and DEPT-135 experiments, the spectra of which were herein provided together with the power-gated-decoupled ^{13}C NMR spectrum.

II. Synthetic methods and characterisation data

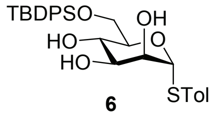

6

4-Methylphenyl 6-*O*-*tert*-butyldiphenylsilyl-1-thio-*α*-D-mannopyranoside (6). A solution of the thioglycoside **5**[3] (50 g, 0.18 mol), *N,N*-dimethylaminopyridine (DMAP, 4.27 g, 0.03 mol) and Et_3N (145 mL, 1.05 mol) in *N,N*-dimethylformamide (DMF, 500 mL) was cooled at 0 °C and *tert*-butyldiphenylchlorosilane (90.6 mL, 0.35 mol) was added dropwise under nitrogen. The reaction mixture was gradually warmed up to room temperature and stirred for 24 h. Then, the reaction flask was immersed in ice bath and quenched with a saturated solution of ammonium chloride. The whole mixture was transferred to a separatory funnel and extracted with ethyl acetate. The combined organic layer was washed with cold water and brine. After $MgSO_4$ drying, the organic layer was concentrated under reduced pressure and the residue was purified by flash column chromatography (ethyl acetate/hexanes = 1/4 to 1/1) to obtain the desired product **6** (86 g, 93%). ^1H NMR (400 MHz, $CDCl_3$): δ 7.69 (t, J = 6.0 Hz, 4H, Ar-H), 7.40–7.34 (m, 6H, Ar-H), 7.31 (d, J = 8.2 Hz, 2H, Ar-H), 6.98

(d, J = 8.2 Hz, 2H, Ar-H), 5.41 (s, 1H, 1-H), 4.27–4.22 (m, 1H, 4-H), 4.16 (bs, 1H, 2-H), 3.99 (dd, 1H, J = 4.4, 11.0 Hz, 6-H_a), 3.92–3.811 (m, 3H, 6-H_b, 3-H, 5-H), 2.28 (s, CH_3), 1.06 (s, 9H, *t*Bu); ^{13}C NMR (100 MHz, $CDCl_3$): δ 137.3 (C), 135.6 (CH), 135.5 (CH), 133.1 (C), 132.9 (C), 132.0 (CH), 130.3 (C), 129.71 (CH), 129.67 (CH), 127.70 (CH), 127.68 (CH), 88.4 (CH), 72.4 (CH), 72.1 (CH), 72.0 (CH), 69.8 (CH), 64.8 (CH_2), 26.8 (CH_3), 21.0 (CH_3), 19.1 (C); HRMS (ESI): *m*/*z* calcd for $C_{29}H_{36}O_5NaSSi$ ($[M + Na]^+$): 547.1950, found: 547.1948.

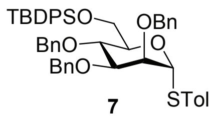

7

4-Methylphenyl 2,3,4-tri-*O*-benzyl-6-*O*-*tert*-butyldiphenylsilyl-1-thio-*α*-D-mannopyranoside (7). The mixture of the triol **6** (60 g, 0.12 mol) and benzyl bromide (44.9 mL, 0.38 mol) in DMF (600 mL) was cooled to 0 °C in an ice-bath. NaH (60% oil dispersion, 16.5 g, 0.69 mol) was then added in five portions over 1 h. After gradually warming up to room temperature, the solution was stirred for an additional 2 h. The reaction mixture was poured carefully in ice-water with vigorous shaking, followed by extraction with ethyl acetate. The combined organic layer was washed with cold water and brine. After drying over $MgSO_4$, the solvent was evaporated *in vacuo*. The crude compound was purified by flash column chromatography (ethyl acetate/hexanes = 1/20) to obtain the desired product **7** (86 g, 94%). $[\alpha]^{27}_D$ +41.3 (*c* 1.6, $CHCl_3$); IR (thin film): v 3073, 2929, 2857, 1495, 1428, 1104, 811, 738 cm^{-1}; ^1H NMR (400 MHz, $CDCl_3$): δ 7.72 (ddd, J = 1.2, 7.9, 14.8 Hz, 4H, Ar-H), 7.41–7.20 (m, 23H, Ar-H), 7.03 (d, J = 7.9 Hz, 2H, Ar-H), 5.56 (d, J = 1.4 Hz, 1H, 1-H), 4.96 (d, J = 10.8 Hz, 1H, PhCH_2), 4.70–4.63 (m, 5H, PhCH_2), 4.19–4.18 (m, 2H, 4-H, 5-H), 4.06–4.01 (m, 2H, 2-H, 6-H_a), 3.93–3.87 (m, 2H, 3-H, 6-H_b), 2.31 (s, 3H, CH_3); ^{13}C NMR (100 MHz, $CDCl_3$): δ 138.5 (C), 138.3 (C), 138.1 (C), 137.2 (C), 136.0 (CH), 135.6 (CH), 133.9 (C), 133.3 (C), 131.5 (CH), 131.2 (C), 129.7 (CH), 129.5 (CH), 129.4 (CH), 128.4 (CH), 128.3 (CH), 128.0 (CH), 127.9 (CH), 127.71 (CH), 127.67 (CH), 127.6 (CH), 127.54 (CH), 127.46 (CH), 86.0 (CH), 80.3 (CH), 76.8 (CH), 75.3 (CH_2),74.8 (CH), 74.0 (CH), 72.2 (CH_2), 71.9 (CH_2), 63.1 (CH_2), 26.8 (CH_3), 21.1 (CH_3), 19.3 (CH); HRMS (ESI): *m*/*z* calcd for $C_{50}H_{54}O_5NaSSi$ ($[M + Na]^+$): 817.3359, found: 817.3362.

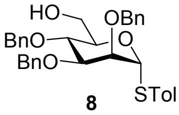

8

4-Methylphenyl 2,3,4-tri-*O*-benzyl-1-thio-*α*-D-mannopyranoside (8). Method A: To the solution of TBDPS compound **7** (22 g, 27.67 mmol) in CH_2Cl_2/MeOH (1/2, 275ml), *p*-toluenesulfonic acid (PTSA, 5.79g, 30.44 mmol) was added and the solution was stirred at room temperature for 12 h. After the completion of reaction, triethylamine was added to quench the reaction and the solvent was evaporated under reduced pressure. The resulting residue was dissolved in EtOAc and washed successively with saturated solution of $NaHCO_{3(aq)}$, water, and brine. After drying over $MgSO_4$, the organic layer was concentrated under reduced pressure and the residue was purified by column chromatography (ethyl acetate/hexanes = 1/7) to furnish the 6-alcohol **8** (14.2 g, 92%). Method B: Compound **12** (1 g, 1.64 mmol) and 2-naphthaldehyde (0.27 g, 1.73 mmol) were dissolved in CH_2Cl_2 (20 mL) and cooled to –78 °C under nitrogen atmosphere. After 5 min, Et_3SiH (0.39 mL, 2.49 mmol) and TMSOTf (60 μL, 0.33 mmol) were added to the reaction mixture and the reaction temperature was gradually raised to –40 °C over a period of 1 h. After stirring at –40 °C for another hour, the reaction flask was directly moved to an ice-water bath. DMF (15 mL), benzyl bromide (0.58 mL, 4.91 mmol), and sodium hydride (60% oil dispersion, 0.24 g, 10.0 mmol) were subsequently added to the stirring mixture. The reaction mixture was gradually warmed up to room temperature and stirred for 4 h. The temperature was again lowered to 0 °C and water (30 mL) was added slowly. After 5 mins, the aqueous layer was removed by cannulation, DDQ (1.86 g, 8.19 mmol) was introduced to the reaction flask, and the reaction was stirred at room temperature for 15 h. The resulting mixture was filtered through a Celite plug and the filtrate was washed successively with saturated $NaHCO_{3(aq)}$ and brine. After drying with anhydrous $MgSO_4$, the solvent was removed under reduced pressure. The resulting crude mixture was purified by flash column chromatography (ethyl acetate/hexanes = 1/7) to furnish the 6-alcohol **8** (0.66 g, 73%). $[\alpha]^{27}_{D}$ +82.9 (*c* 2.4, $CHCl_3$); IR (thin film): *v* 3478, 3034, 2918, 2870, 1740, 1495, 1454, 1366, 1209, 1088, 1028, 811, 736 cm^{-1}; ^{1}H NMR (400 MHz, $CDCl_3$): *δ* 7.34–7.25 (m, 17H, Ar-H), 7.09 (d, *J* =8.0 Hz, 2H, Ar-H), 5.42 (d, *J* =1.5 Hz, 1H, 1-H), 4.94 (d, *J* = 10.9 Hz, 1H, PhCH_2), 4.71–4.60 (m, 5H, PhCH_2), 4.14–4.10 (m, 1H, 5-H), 4.01 (t, *J* = 9.5 Hz, 4-H), 3.98–3.97 (m, 1H, 2-H), 3.88 (dd, *J* = 3.0, 9.3 Hz, 1H, 3-H), 3.81–3.77 (m, 2H, 6-H × 2), 2.31 (s, 3H, CH_3); ^{13}C NMR (100 MHz, $CDCl_3$): *δ* 138.3 (C), 138.1 (C), 137.9 (C), 137.7 (C), 132.4 (CH), 130.0 (C), 129.9 (CH), 128.4 (CH), 128.0(CH), 127.9 (CH), 127.8 (CH), 127.7 (CH), 86.3 (CH), 80.0 (CH), 76.3 (CH), 75.23

(CH_2), 74.7 (CH), 73.1 (CH), 72.3 (CH_2), 72.2 (CH_2), 62.1 (CH_2), 21.1 (CH_3); HRMS (ESI): *m*/*z* calcd for $C_{34}H_{36}O_5NaS$ ([M + Na]$^+$): 579.2181, found: 579.2181.

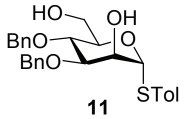

11

4-Methylphenyl 3,4-di-*O*-benzyl-1-thio-*α*-D-mannopyranoside (11). A mixture of the tetrakis-trimethylsilyl ether **9**[4] (1.0 g, 1.74 mmol) and benzaldehyde (0.371 mL, 3.65 mmol) in CH_3CN (10 mL) was stirred at 0 °C under nitrogen atmosphere. Trimethylsilyl trifluoromethanesulfonate (TMSOTf, 9 μL, 0.052 mmol) was added to the solution and the mixture was kept stirring at the same temperature for 30 min. A small portion of precipitated white solid was separated for the recrystallization and X-ray analysis of the dibenzylidene compound **10**. The single crystal of compound **10** was obtained by vapor diffusion method using ethyl acetate and hexane. The original reaction was then neutralized with Et_3N and the solvent was removed under reduced pressure. The white solid obtained was dissolved in dichloromethane and $BH_3{\cdot}THF$ (1 M in THF, 17.3 mL, 17.3 mmol) and copper(II) trifluoromethanesulfonate (32 mg, 0.09 mmol) were sequentially added under nitrogen atmosphere at room temperature. After 15 h, the reaction was quenched with MeOH and neutralized with Et_3N. The solvent was evaporated under reduced pressure. The resulting residue was dissolved in ethyl acetate and washed successively with saturated $NaHCO_{3(aq)}$, water and brine. The organic layer was dried over $MgSO_4$ and concentrated in *vacuo*. The residue was purified by flash chromatography (acetone/CH_2Cl_2 =1/15) to obtain the desired diol **11** (0.71 g, 87%). $[\alpha]^{17}_{D}$ +213.7 (*c* 2.83, $CHCl_3$); IR (thin film): *ν* 3395, 3031, 2918, 2870, 1498, 1449, 1101, 1036, 819, 763, 709 cm^{-1}; ^{1}H NMR (600 MHz, $CDCl_3$): *δ* 7.37–7.28 (m, 12H, Ar-H), 7.06 (d, *J* = 7.9 Hz, 2H, Ar-H), 5.48 (bs, 1H, 1-H), 4.22 (dd, *J* = 1.2, 3.0 Hz, 1H, 2-H), 4.14 (dt, *J* = 2.4, 9.6 Hz, 1H, 5-H), 3.94 (t, *J* = 9.6 Hz, 1H, 4-H), 3.88 (dd, *J* = 3.0, 9.6 Hz, 1H, 3-H), 3.81–3.76 (m, 2H, 6-H × 2), 3.40 (bs, 1H, OH), 2.62 (bs, 1H, OH), 2.24 (s, 3H, CH_3); ^{13}C NMR (150 MHz, $CDCl_3$): *δ* 138.1 (C), 137.9 (C), 137.6 (C), 132.4 (CH), 129.9 (CH), 129.5 (C), 128.6 (CH), 128.4 (CH), 128.10 (CH), 128.05 (CH), 128.0 (CH), 127.8 (CH), 87.6 (CH), 80.0 (CH), 75.23 (CH_2),74.0 (CH), 72.6 (CH), 72.2 (CH_2), 69.8 (CH), 61.6 (CH_2), 21.0 (CH_3); HRMS (ESI): *m*/*z* calcd for $C_{27}H_{30}O_5NaS$ ([M + Na]$^+$): 489.1712, found: 489.1706.

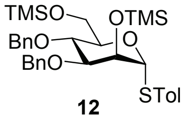

12

4-Methylphenyl 2,6-di-*O*-trimethylsilyl-3,4-di-*O*-benzyl-1-thio-*α*-D-mannopyranoside (12). The diol **11** (17.0 g, 0.036 mol) was dissolved in CH_2Cl_2 (170 mL) and the reaction flask was immersed in an ice bath. After the addition of Et_3N (30.5 mL, 0.219 mol), trimethylchlorosilane (TMSCl, 18.5 mL, 0.146 mol) was slowly added to the solution, and the mixture was gradually warmed up to room temperature. After stirring for 12 h, the solution was concentrated *in vacuo*, the obtained residue was suspended in hexane (100 mL), and the whole mixture was filtered through Celite. The filtrate was concentrated under reduced pressure to afford compound **12** (22.2 g, quantitative). $[\alpha]^{20}_{D}$ +125.3 (*c* 2.5, $CHCl_3$); IR (thin film): *v* 2955, 1639, 1494, 1249, 1101, 867, 749 cm^{-1}; ^1H NMR (600 MHz, $CDCl_3$): *δ* 7.36 (d, *J* = 7.8 Hz, 2H, Ar-H), 7.35–7.25 (m, 10H, Ar-H), 7.08 (d, *J* = 7.8 Hz, Ar-H), 5.27 (d, *J* = 1.8 Hz, 1H, 1-H), 4.89 (d, *J* = 11.4 Hz, 1H, $PhCH_2$), 4.72, 4.65 (ABq, *J* = 12.0, 11.4 Hz, 2H, $PhCH_2$), 4.61 (d, *J* =11.4 Hz, 1H, $PhCH_2$), 4.25 (dd, *J* = 2.4, 2.7 Hz, 1H, 2-H), 4.10 (ddd, *J* = 2.4, 4.8, 9.4 Hz, 1H, 5-H), 3.88 (t, *J* = 9.4 Hz, 1H, 4-H), 3.84 (dd, *J* = 5.4, 11.4 Hz, 1H, 6-H_a), 3.81 (dd, *J* = 2.4, 11.4 Hz, 1H, 6-H_b), 3.76 (dd, *J* =, 2.4, 9.4 Hz, 1H, 3-H); ^{13}C NMR (150 MHz, $CDCl_3$): *δ* 138.63 (C), 138.20 (C), 137.38 (C), 132.13 (CH), 130.86 (C) 129.63 (CH), 128.31 (CH), 127.87 (CH), 127.82 (CH), 127.58 (CH), 127.54 (CH), 89.56 (CH), 80.38 (CH), 74.78 (CH_2), 74.60 (CH), 74.01 (CH), 72.41 (CH_2), 71.11 (CH), 62.10 (CH_2), 21.08 (CH_3), 0.35 (CH_3), –0.26 (CH_3); HRMS (FAB): *m*/*z* calcd for $C_{33}H_{46}O_5NSi_2S$ ($[M]^+$): 610.2600, found: 610.2605.

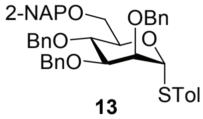

13

4-Methylphenyl 2,3,4-tri-*O*-benzyl-6-*O*-(2-naphthylmethyl)-1-thio-*α*-D-mannopyranoside (13). Compound **12** (11 g, 18.0 mmol) and 2-naphthaldehyde (2.95 g, 18.9 mmol) were dissolved in CH_2Cl_2 (150 mL) and cooled to –78 °C under nitrogen atmosphere. After 5 min, Et_3SiH (4.31 mL, 27.0 mmol) and TMSOTf (651 µL, 3.60 mmol) were added to the reaction mixture and the temperature was gradually raised to –40 °C for a period of 1 h. After stirring at –40 °C for an additional 1 h, the reaction flask was directly moved to an ice-bath and DMF (150 mL), benzyl bromide (6.42 mL, 54.0 mmol), and NaH (60% oil dispersion, 2.59 g, 108.0 mmol, in 3 portions) were added to the reaction. The

reaction was gradually warmed up to room temperature. After 4 h of stirring, the reaction was again cooled to 0 °C and quenched with water until the effervescence ceased. The crude compound was extracted with CH_2Cl_2 and the combined organic layer was washed successively with water and brine, dried over $MgSO_4$ and concentrated under reduced pressure. The resulting residue was purified by flash column chromatography (ethyl acetate/hexanes = 1/7) to obtain the desired product **13** (10.1 g, 81%). $[\alpha]^{23}_{D}$ +78.3 (*c* 2.1, $CHCl_3$); IR (thin film): *v* 3056, 3026, 2866, 1600, 1495, 1454, 1366, 1204, 1097, 813 cm^{-1}; ^{1}H NMR (400 MHz, $CDCl_3$): δ 7.94–7.88 (m, 4H, Ar-H), 7.62–7.28 (m, 24H, Ar-H), 7.14 (d, *J* = 8.0 Hz, 2H, Ar-H), 5.77 (d, *J* = 1.3 Hz, 1H, 1-H), 5.08 (d, *J* = 10.9 Hz, 1H, ArCH_2), 4.94 (d, *J* = 12.1 Hz, 1H, ArCH_2), 4.87 (d, *J* = 12.3 Hz, 1H, ArCH_2), 4.79–4.75 (m, 4H, ArCH_2), 4.69 (d, *J* = 10.9 Hz, 1H, ArCH_2), 4.54 (ddd, *J* = 1.4, 4.9, 9.7 Hz, 1H, 5-H), 4.27 (t, *J* = 9.5 Hz, 4-H), 4.18 (dd, *J* = 1.8, 2.8 Hz, 1H, 2-H), 4.09–4.03 (m, 2H, 3-H, 6-H_a), 3.96 (dd, *J* = 1.3, 11.0 Hz, 1H, 6-H_b), 2.38 (s, 3H, CH_3); ^{13}C NMR (100 MHz, $CDCl_3$): δ 138.2 (C), 138.0 (C), 137.7 (C), 137.3 (C), 135.6 (C), 133.0 (C), 132.7 (C), 131.9 (CH), 130.3 (C), 129.6 (CH), 128.2 (CH), 128.1 (CH), 128.0 (CH), 127.8 (CH), 127.7 (CH), 127.62 (CH), 127.56 (CH), 127.4 (CH), 127.3 (CH), 126.2 (CH), 125.7 (CH), 125.5 (CH), 85.8 (CH), 80.0 (CH), 76.0 (CH), 74.9 (CH_2), 74.8 (CH), 73.1 (CH_2), 72.5 (CH), 71.8 (CH_2), 71.6 (CH_2), 69.0 (CH_2), 20.8 (CH_3); HRMS (ESI): *m*/*z* calcd for $C_{451}H_{44}O_5NaS$ ($[M + Na]^+$): 719.2807, found: 719.2802.

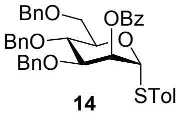

14

4-Methylphenyl 2-*O*-benzoyl-3,4,6-tri-*O*-benzyl-1-thio-*α*-D-mannopyranoside (14). Compound **12** (20 g, 32.74 mmol) was dissolved in CH_2Cl_2 (300 mL) and cooled to –78 °C under nitrogen atmosphere. After the addition of benzaldehyde (3.49 mL, 34.37 mmol) and Et_3SiH (7.84, 49.10 mmol), the reaction mixture was stirred at –78 °C for 5 min. TMSOTf (1.18 mL, 1.46 mmol) was added dropwise and the reaction was stirred at –78 °C further for 1.5 h. After the complete consumption of starting material, acetonitrile (20 mL) and $BF_3 \cdot OEt_2$ (2.07 mL, 19.37 mmol) were added to the reaction and the stirring was continued while gradually warming the reaction temperature to –20 °C for a period of 30 min. Benzoic anhydride (22.2 g, 98.2 mmol), Et_3N (16.6 mL, 163.7 mmol) and DMAP (0.80 g, 6.55 mmol) were then introduced to the reaction and the reaction was warmed gradually to room temperature. After 12 h of stirring, the solvents were evaporated under reduced pressure.

The residue was dissolved in ethyl acetate and washed consecutively with water, saturated $NaHCO_{3(aq)}$ and brine. The organic layer was dried over $MgSO_4$, filtered and concentrated under reduced pressure. The crude material was purified by flash column chromatography (ethyl acetate/hexanes = 1/8) to afford compound **14** (20.2 g, 93%). $[\alpha]^{24}_{D}$ +76.9 (*c* 5.6, $CHCl_3$); IR (thin film): *ν* 3088, 2920, 2865, 1722, 1601, 1584, 1494, 1452, 1265, 1090, 909, 737, 697 cm^{-1}; ^{1}H NMR (600 MHz, $CDCl_3$): *δ* 8.16 (dd, *J* = 1.0, 8.3 Hz, 2H, Ar-H), 7.59 (m, 1H, Ar-H), 7.49–7.31 (m, 19H, Ar-H), 7.13 (d, *J* = 8.3 Hz, 2H, Ar-H), 5.97 (dd, *J* = 1.8, 3.0 Hz, 1H, 2-H), 5.68 (d, *J* = 1.8, 1H, 1-H), 5.00 (d, *J* = 10.8 Hz, 1H, $PhCH_2$), 4.90 (d, *J* = 11.4 Hz, 1H, $PhCH_2$), 4.79 (d, *J* = 12.0 Hz, 1H, $PhCH_2$), 4.69 (d, *J* = 11.4 Hz, 1H, $PhCH_2$), 4.67 (d, *J* = 10.8 Hz, 1H, $PHCH_2$), 4.58 (d, *J* = 12 Hz, 1H, $PhCH_2$), 4.51 (ddd, *J* = 1.6, 4.0, 9.8 Hz, 1H, 5-H), 4.62 (t, *J* = 9.6 Hz, 1H, 4-H), 4.17 (dd, *J* =3.0, 9.6 Hz, 1H, 3-H), 4.04 (dd, *J* = 4.2, 10.8 Hz, 1H, 6-H_a), 3.88 (dd, *J* = 1.8, 10.8 Hz, 1H, 6-H_b), 2.36 (s, 3H, CH_3); ^{13}C NMR (100 MHz, $CDCl_3$): *δ* 165.5 (C=O), 138.3 (C), 138.2 (C), 137. 8 (C), 137.5 (C), 133.1 (CH), 132.3 (CH), 129.8 (CH), 129.8 (C), 129.73 (CH), 129.68 (C), 128.3 (CH), 128.2 (CH), 128.2 (CH), 128.1 (CH), 128.0 (CH), 127.9 (CH), 127.7 (CH), 127.6 (CH), 127.41 (CH), 127.36 (CH), 86.6 (CH), 78.5 (CH), 75.2 (CH_2), 74.4 (CH), 73.3 (CH_2), 72.5 (CH), 71.5 (CH_2), 70.5 (CH), 68.9 (CH_2), 21.0 (CH_3); HRMS (ESI): *m*/*z* calcd for $C_{41}H_{40}O_6NaS$ ($[M + Na]^+$): 683.2443, found: 683.2441.

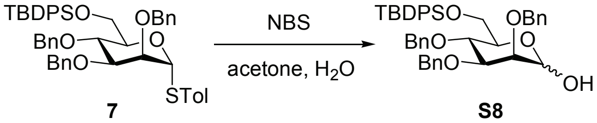

7 **S8**

2,3,4-Tri-*O*-benzyl-6-*O*-*tert*-butyldiphenylsilyl-D-mannopyranose (S8). Water (5 ml, 0.30 mol) and *N*-bromosuccinimide (NBS, 27 g, 0.15 mol) were added to a solution of thioglycoside **7** (80.0 g, 0.10 mol) in acetone (1.0 L) at 0 °C. The solution was stirred at the same temperature for 30 min. The reaction was quenched with 10% $Na_2S_2O_{3(aq)}$ and the solvent was evaporated under reduced pressure. The residue was dissolved in ethyl acetate and washed successively with 10% $Na_2SO_{3(aq)}$ solution and brine, dried over $MgSO_4$ and concentrated under reduced pressure. The crude product was purified by flash chromatography (ethyl acetate/hexanes =1/4) to obtain the hemiacetal **S8** (66 g, 95%; *α*/*β* 1/0.11) as a colorless thick syrup. ^{1}H NMR (400 MHz, $CDCl_3$): *δ* 7.75–7.67 (m, 4.5H, Ar-H), 7.41–7.16 (m, 23.3 H, Ar-H), 5.22 (bs, 1H), 5.15 (d, *J* = 11.5 Hz, 0.11H), 4.94–4.89 (m, 1.11H), 4.83–4.75 (m, 1.11H), 4.68–4.55 (m, 4.44H), 4.20–4.10 (m, 1.11H), 4.03–3.94 (m, 2.22H), 3.91–3.79 (m, 3.11H), 3.64 (dd, 0.11H), 3.59 (d, 0.11H), 2.36 (s, 1H, OH), 1.04 (s,

9H); ^{13}C NMR (100 MHz, $CDCl_3$): δ 138.6 (C), 138.4 (C), 135.9 (CH), 135.6 (CH), 133.9 (C), 133.4 (C), 129.5 (CH), 128.5 (CH), 128.3 (CH), 128.5 (CH), 127.8 (CH), 127.7 (CH), 127.6 (CH), 127.6 (CH), 127.5 (CH), 127.5 (CH), 93.4 (CH), 92.7 (CH), 82.9 (CH), 79.6 (CH), 76.0 (CH), 75.5 (CH), 75.1 (CH_2), 74.7 (CH_2), 74.6 (CH), 74.15 (CH), 73.14 (CH), 72.7 (CH_2), 72.2 (CH_2), 63.4 (CH_2), 62.9 (CH_2), 26.8 (CH_3), 19.3 (C); HRMS (ESI): *m*/*z* calcd for $C_{43}H_{48}O_6NaSi$ ($[M + Na]^+$): 711.3118, found: 711.3112.

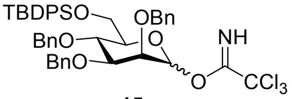

15

2,3,4-Tri-*O*-benzyl-6-*O*-*tert*-butyldiphenylsilyl-D-mannopyranosyl trichloroacetimidate (15). K_2CO_3 (3.31 g, 24.0 mmol) was added to the solution of hemiacetal **S8** (3.3 g, 4.79 mmol) and CCl_3CN (4.81 mL, 47.90 mmol) in CH_2Cl_2 while maintaining the temperature at 0 °C under nitrogen atmosphere. The reaction was warmed up to room temperature and stirred for 12 h. The whole mixture was filtered through Celite, and the solution was washed successively with water and brine, dried over $MgSO_4$, and concentrated under reduced pressure to obtain the trichloroacetimidate **15** (3.84 g, 99%; α/β = 4/1), which was used for the next step without any further purification. ^{1}H NMR (400 MHz, $CDCl_3$): δ 8.6 (s, 1H), 8.5 (s, 4H), 7.75–7.69 (m, 24H), 7.47–7.20 (m, 101H), 6.39 (s, 4H), 5.86 (s, 1H), 5.02–4.57 (m, 30H), 4.33–4.15 (m, 7H), 4.05–3.87 (m, 24H), 3.82–3.70 (m, 2H), 3.65–3.54 (m, 2H), 1.05 (s, 36H), 1.01 (s, 9H).

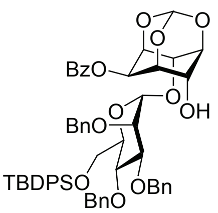

17

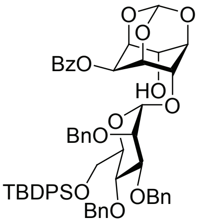

18

2-*O*-Benzoyl-6-*O*-(2,3,4-tri-*O*-benzyl-6-*O*-*tert*-butyldiphenylsilyl-*α*-D-mannopyranosyl)-D-*myo*-inositol-1,3,5-orthoformate (17) and 2-*O*-benzoyl-4-*O*-(2,3,4-tri-*O*-benzyl-6-*O*-*tert*-butyldiphenylsilyl-*α*-D-mannopyranosyl)-D-*myo*-inositol-1,3,5-orthoformate (18). A mixture of mannosyl trichloroacetimidate **15** (1.5 g, 1.80 mmol), 4,6-diol **16**[2] (0.530 g, 1.80 mmol) and freshly dried 3 Å molecular sieves (5.0 g) was stirred in dioxane (100 mL) and CH_2Cl_2 (5 mL) at room temperature for 1 h under nitrogen atmosphere. Silver trifluoromethanesulfonate (2.31g, 9.01 mmol) was then added to the

reaction mixture. After 1 h of stirring, another solution of the imidate **15** (1.5 g, 1.80 mmol) in CH_2Cl_2 (5 mL) was added to the reaction and the stirring was continued at the same temperature for an additional 2 h. The reaction was quenched by adding Et_3N and the whole mixture was filtered through Celite and concentrated under reduced pressure. The obtained residue was dissolved in ethyl acetate/hexanes (1/2) and filtered through a short plug of silica gel followed by washing with the same solvent. The filtrate was consecutively washed with saturated $NaHCO_{3(aq)}$, water and brine, dried over $MgSO_4$, and concentrated under reduced pressure. The crude compound was purified by flash column chromatography (ethyl acetate/hexanes = 1/4 to 1/3) to obtain the pseudodisaccharides **17** (1.21 g, 68%) and **18** (0.310 g, 20%). For **17**: $[\alpha]^{26}_{D}$ +33.1 (*c* 1.0, $CHCl_3$); ^1H NMR (400 MHz, $CDCl_3$): δ 8.18 (d, *J* = 1.0, 8.4 Hz, 2H, Ar-H), 7.69–7.11 (m, 28H, Ar-H), 5.52 (d, *J* = 1.04 Hz, 1H, orthoformate-H), 5.31 (d, *J* = 1.4 Hz, 1H, 2-H), 4.98 (d, *J* = 2.3 Hz, 1H, 1′-H), 4.18 (d, *J* = 12.4 Hz, 1H, PhC*H*2), 4.80 (d, *J* = 10.0 Hz, 1H, PhC*H*2), 4.68–4.59 (m, 4H, PhCH*H* × 3, 6-H), 4.49–4.43 (m, 2H, PhC*H*2, 4-H), 4.40–4.37 (m, 1H, 3-H), 4.30–4.28 (m, 1H, 5-H), 3.93 (t, *J* = 8.7 Hz, 1H, 4′-H), 3.88–3.87 (m, 2H, 6′-H × 2), 3.75–3.73 (m, 2H, 3′-H, 5′-H), 3.66 (t, *J* = 2.8 Hz, 1H, 2′-H), 3.18 (bs, 1H, OH), 1.05 (s, 9H, CH_3); ^{13}C NMR (100 MHz, $CDCl_3$): δ 165.1 (C), 139.0 (C × 3), 138.0 (C × 2), 135.8 (CH), 135.6 (CH), 133.2 (C), 130.0 (CH), 129.7 (CH), 128.5 (CH), 128.41 (CH), 128.37 (CH), 128.3 (CH), 128.1 (CH), 128.0 (CH), 127.9 (CH), 127.8 (CH), 127.7 (CH), 127.6 (CH), 127.5 (CH), 102.7 (CH), 97.6 (CH, *J* = 170.9 Hz, C-1′), 78.5 (CH), 75.6 (CH), 74.8 (CH_2), 74.4 (CH), 74.3 (CH), 73.2 (CH_2), 72.7 (CH_2), 72.2 (CH), 71.5 (CH), 69.0 (CH), 68.8 (CH), 67.8 (CH), 63.4 (CH), 63.3 (CH_2), 26.8 (CH_3), 19.2 (C); HRMS (ESI): *m*/*z* calcd for $C_{57}H_{60}O_{12}NaSi$ ($[M + Na]^+$): 987.3752, found: 987.3759. For **18**: $[\alpha]^{27}_{D}$ +42.2 (*c* 2.0, $CHCl_3$); ^1H NMR (400 MHz, $CDCl_3$): δ 8.07 (dd, *J* = 1.3,7.08 Hz, 2H, Ar-H), 7.65–7.18 (m, 28H, Ar-H), 5.52 (bs, 1H, orthoformate-H), 5.37 (s, 1H, 2-H), 4.91 (d, *J* = 2.2 Hz, 1H, 1′-H), 4.79 (d, *J* = 11.5 Hz, 1H, PhC*H*2), 4.76 (d, *J* = 12.3 Hz, 1H, PhC*H*2), 4.72 (d, *J* = 11.8 Hz, 1H, PhC*H*2), 4.61–4.49 (m, 5H, PhCH*H* × 3, 4-H, 6-H), 4.39–4.36 (m, 1H, 3-H), 4.24–4.21 (m, 1H, 5-H), 4.02 (t, *J* = 8.9 Hz, 1H, 4′-H), 3.89 (dd, *J* = 4.2, 11.2 Hz, 6′-H_a), 3.85–3.82 (m, 1H, 6′-H_b), 3.78 (dd, *J* = 2.6, 8.9 Hz, 1H, 3′-H), 3.75–3.73 (m, 1H, 5′-H), 3.54 (t, *J* = 2.6 Hz, 1H, 2′-H), 1.0 (s, 9H, CH_3); ^{13}C NMR (100 MHz, $CDCl_3$): δ 165.7 (C), 138.1 (C), 138.1 (C), 137.9 (C), 135.8 (CH), 135.6 (CH), 133.6 (C), 133.3 (CH), 133.2 (CH), 129.9 (CH), 129.5 (CH), 128.5 (CH), 128.4 (CH), 128.3 (CH), 128.1 (CH), 127.92 (CH), 127.89 (CH), 127.6 (CH), 127.5 (CH), 102.7 (CH), 98.7 (CH, *J* = 171.2 Hz, C-1′), 78.4 (CH), 75.9 (CH), 74.5 (CH_2), 74.4 (CH), 74.2 (CH), 73.2 (CH_2), 72.9

(CH_2), 72.2 (CH), 72.1 (CH), 70.5 (CH), 67.9 (CH), 67.3 (CH), 63.3 (CH), 62.3 (CH_2), 26.7 (CH_3), 19.2 (C); HRMS (ESI): *m/z* calcd for $C_{57}H_{60}O_{12}NaSi$ ($[M + Na]^+$): 987.3752, found: 987.3754.

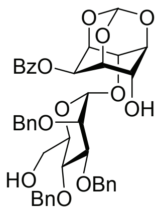

S1

2-*O*-Benzoyl-6-*O*-(2,3,4-tri-*O*-benzyl-*α*-D-mannopyranosyl)-D-*myo*-inositol-1,3,5-orthoformate (S1). To a solution of disaccharide **17** (50 mg, 0.052 mmol) in CH_2Cl_2 (1 mL), tetrabutylammonium fluoride (TBAF, 1 M in THF, 1 mL, 1.036 mmol) and acetic acid (59 μL, 1.036 mmol) were added at room temperature. After stirring for 24 h, the reaction was diluted with ethyl acetate and washed successively with water and brine. The resulting solution was dried over $MgSO_4$, filtered and concentrated under reduced pressure. The residue was purified by flash column chromatography (ethyl acetate/hexanes = 1/2) to afford the diol **S1** (32 mg, 86%). $[\alpha]^{23}_D$ +43.1 (*c* 2.5, $CHCl_3$); IR (thin film): ν 3478, 3030, 2931, 1720, 1456, 1273, 1168, 1076, 1000, 957, 802 cm^{-1}; ^1H NMR (400 MHz, $CDCl_3$): δ 8.18 (dd, *J* = 1.2, 8.3 Hz, 2H, Ar-H), 7.63–7.59 (m, 1H, Ar-H), 7.51–7.47 (m, 2H, Ar-H), 7.39–7.21 (m, 15H, Ar-H), 5.53 (d, *J* = 1.2 Hz, 1H, orthoformate-H), 5.28 (d, *J* = 2.3 Hz, 1H, 2-H), 4.97 (d, *J* = 2.3 Hz, 1H, 1′-H), 4.86 (d, *J* = 10.9 Hz, 1H, PhC*H*2), 4.80 (d, *J* = 12.2 Hz, 1H, PhC*H*2), 4.67–4.57 (m, 5H, PhCH*H* × 4, 5-H), 4.52 (bs, 1H, 4-H), 4.41–4.39 (m, 1H, 3-H), 4.37–4.35 (m, 1H, 1-H), 4.31–4.29 (m, 1H, 6-H), 3.92 (t, *J* = 9.5 Hz, 1H, 4′-H), 3.81 (dd, *J* = 2.0, 9.5 Hz, 1H, 3′-H), 3.76–3.67 (m, 4H, 5′-H, 2′-H, 6′-H × 2); ^{13}C NMR (100 MHz, $CDCl_3$): δ 166.0 (C=O), 137.9 (C), 137.8 (C), 137.7 (C), 133.4 (CH), 129.9 (CH), 129.5 (C), 128.5 (CH), 128.44 (CH), 128.38 (CH), 128.1 (CH), 128.02 (CH), 127.98 (CH), 127.8 (CH), 102.6 (CH), 98.1 (CH), 78.4 (CH), 75.2 (CH), 74.8 (CH_2), 74.5 (CH), 73.4 (CH), 73.2 (CH_2), 72.5 (CH_2), 72.00 (CH), 71.96 (CH), 69.1 (2 × CH), 67.7 (CH), 63.3 (CH), 62.0 (CH_2): HRMS (ESI): *m/z* calcd for $C_{41}H_{42}O_{12}Na$ ($[M +Na]^+$): 749.2574, found: 749.2567.

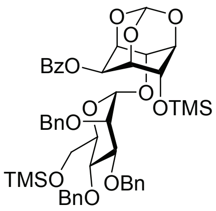

S2

2-*O*-Benzoyl-6-*O*-(2,3,4-tri-*O*-benzyl-6-*O*-trimethylsilyl-*α*-D-mannopyranosyl)-4-*O*-trimethylsilyl-D-*myo*-inositol-1,3,5-orthoformate (S2). To a solution of the diol **S1** (0.50 g, 0.69 mmol) in CH_2Cl_2 at 0 °C, Et_3N (0.58 mL, 4.13 mmol) and trimethylchlorosilane (0.350 mL, 2.75 mmol) were added under nitrogen atmosphere. The reaction was gradually warmed up to room temperature and stirred for 15 h. Afterwards, the solvents were evaporated under reduced pressure, and the residue was suspended in hexane, stirred for 5 min and then filtered through a Celite plug. The combined filtrates were concentrated under reduced pressure to obtain compound **S2** (0.567 g, 94%). $[\alpha]^{27}_{D}$ +10.1 (*c* 1.0, $CHCl_3$); ^{1}H NMR (600 MHz, $CDCl_3$): δ 8.18 (d, J = 7.0 Hz, 2H, Ar-H), 7.59 (m, 1H, Ar-H), 7.47 (m, 2H, Ar-H), 7.38–7.25 (m, 15 H, Ar-H), 5.54 (bs, 1H, orthoformate-H), 5.46 (bs, 1H, 2-H), 4.96 (bs, 1H, 1′-H), 4.93 (d, J = 11.5 Hz, 1H, PhCH_2), 4.78 (d, J = 12.4 Hz, 1H, PhCH_2), 4.67 (d, J = 12.4 Hz, 1H, PhCH_2), 4.61–4.56 (m, 4H, PhCH*H* × 3, 4-H), 4.51–4.47 (m, 1H, 6-H), 4.41–4.38 (m, 1H, 5-H), 4.30–4.28 (m, 1H, 1-H), 4.26–2.23 (m, 1H, 3-H), 3.90–3.73 (m, 6H, 2′-H, 3′-H, 4′-H, 5′-H, 6′-H × 2), 0.15 (s, 9H, Si(CH_3)$_3$), 0.11 (s, 9H, Si(CH_3)$_3$); ^{13}C NMR (150 MHz, $CDCl_3$): δ 166.1 (C=O), 138.8 (C), 138.4 (C), 138.2 (C), 133.3 (CH), 129.9 (CH), 129.8 (C), 128.4 (CH), 128.33 (CH), 128.29 (CH), 128.1 (CH), 127.9 (CH), 127.7 (CH), 127.6 (CH), 127.5 (CH), 127.3 (CH), 102.8 (CH), 99.3 (CH), 80.0 (CH), 75.1 (CH), 74.9 (CH), 74.7 (CH_2), 73.6 (CH), 73.5 (CH), 72.9 (CH_2), 72.4 (CH), 72.3 (CH_2), 71.0 (CH), 70.4 (CH), 68.0 (CH), 64.0 (CH), 62.3 (CH_2), –0.2 (CH_3), –0.4 (CH_3); HRMS (ESI): *m/z* calcd for $C_{47}H_{58}O_{12}NaSi_2$ ([M + Na]$^+$): 893.3365, found: 893.3356.

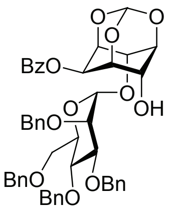

S3

2-*O*-Benzoyl-6-*O*-(2,3,4,6-tetra-*O*-benzyl-*α*-D-mannopyranosyl)-D-*myo*-inositol-1,3,5-orthoformate (S3). To the solution of compound **S2** (50 mg, 0.057 mmol) in CH_2Cl_2 (2 mL) at –78 °C, benzaldehyde (6.4 µL, 0.066 mmol) and Et_3SiH (14µL, 0.086 mmol) were

added under nitrogen atmosphere. The resulting solution was stirred for 5 min before introducing TMSOTf (2 μL, 0.014 mmol) to the reaction followed by stirring at –78 °C for 2 h. The reaction was quenched with TBAF (1 M solution in THF, 69 μL, 0.069 mmol) and placed in an ice-water bath for 5 min. Ethyl acetate was then added and the mixture was washed thoroughly with water and brine. The organic layer was dried over $MgSO_4$ and concentrated under reduced pressure. The crude residue was purified by flash column chromatography (ethyl acetate/hexanes = 1/3) to furnish compound **S3** (41 mg, 87%).

^{1}H NMR (600 MHz, $CDCl_3$) (*δ*, ppm)	
Obtained	Literature[2]
8.09 (dd, *J* = 8.0, 1.0 Hz, 2H, Ar-H)	8.09 (dd, *J* = 8.0, 1.0 Hz, 2H, Ar-H)
7.51 (t, *J* = 7.4 Hz, 1H, Ar-H)	7.52 (td, *J* = 7.4, 7.4, 1.0 Hz, 1H, Ar-H)
7.39 (t, *J* = 7.9 Hz, 2H, Ar-H)	7.40 (t, *J* = 8.0 Hz, 2H, Bz-H)
7.33-7.06 (m, 20H, Ar-H)	7.53-7.16 (m, 20H, Ar-H)
5.44 (bs, 1H, orthoformate-H)	5.46 (d, *J* = 1.3 Hz, 1H, orthoformate-H)
5.26 (bs, 1H, 2-H)	5.29 (d, *J* = 1.5 Hz, 1H, 2-H)
4.96 (d, *J* = 3.1 Hz, 1H, 1′-H)	4.97 (d, *J* = 2.9 Hz, 1H, 1′-H)
4.67 (d, *J* = 12.2 Hz, 1H, PhC*H*$_2$)	4.68 (d, *J* = 12.2 Hz, 1H, PhC*H*$_2$)
4.61 (d, *J* = 11.9 Hz, 2H, PhCH$_2$)	4.63 (d, *J* = 8.9 Hz, 1H, PhC*H*$_2$)
	4.61 (d, *J* = 12.2 Hz, 1H, PhC*H*$_2$)
4.55–4.48 (m, 4H, Ins-H + 3 PhCH*H*),	4.95–4.56 (m, 4H, Ins-H + 3 PhCH*H*)
4.43 (d, *J* = 11.6 Hz, 1H, PhC*H*$_2$)	4.44 (d, *J* = 11.7 Hz, 1H, PhC*H*$_2$)
4.31-4.23 (m, 5H, 4 Ins-H + PhC*H*$_2$)	4.32–4.26 (m, 5H, 4 Ins-H + PhC*H*$_2$),
3.81–3.79 (m, 1H, 5′-H)	3.82 (m, 1H, 5′-H)
3.71–3.64 (m, 2H)	3.72-3.68 (m, 2H, 3′-H + 4′-H)
3.64–3.62 (m, 1H, 2′-H)	3.66 (t, *J* = 2.9 Hz, 1H, 2′-H)
3.62–3.60 (m, 1H, 6′-H$_a$)	3.62 (dd, *J* = 10.2, 2.2 Hz, 1H, 6′-H$_a$)
3.56 (dd, *J* = 10.3 Hz, 6.5 Hz, 1H, 6′-Hb)	3.59 (dd, *J* = 10.2, 6.5 Hz, 1H, 6′-H$_b$)

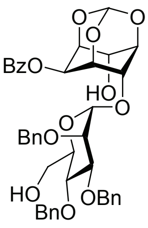

S4

2-*O*-Benzoyl-4-*O*-(2,3,4-tri-*O*-benzyl-*α*-D-mannopyranosyl)-D-*myo*-inositol-1,3,5-orthoformate (S4). Compound **S4** was prepared from compound **18** by using the same procedure applied in generating compound **S1**. $[\alpha]^{23}_{D}$ +63.9 (*c* 1.2, $CHCl_3$); IR (thin film): *ν* 3500, 3034, 2922, 1720, 1454, 1364, 1273, 1165, 1073, 959 cm^{-1}; ^{1}H NMR (600 MHz, $CDCl_3$): *δ* 8.13 (d, *J* = 7.3 Hz, 2H, Ar-H), 7.58 (t, *J* = 7.4 Hz, 1H, Ar-H), 7.45 (t, *J* = 7.8 Hz, 3H, Ar-H), 7.36–7.26 (m, 15H, Ar-H), 5.52 (s, 1H, orthoformate-H), 5.38 (s, 1H, 2-H), 4.92 (d, *J* = 2.3 Hz, 1H, 1′-H), 4.82 (d, *J* = 11.1 Hz, 1H, $PhCH_2$), 4.75 (d, *J* = 12.0 Hz, 1H, $PhCH_2$), 4.71 (d, *J* = 11.8 Hz, 1H, $PhCH_2$), 4.61 (d, *J* = 11.8 Hz, 1H, $PhCH_2$), 4.60 (d, *J* = 11.1 Hz, 1H, $PhCH_2$), 4.56 (d, *J* = 12.0 Hz, 1H, $PhCH_2$), 4.55–4.53 (m, 1H, 4-H), 4.51–4.47 (m, 2H, 3-H, 6-H), 4.40–4.39 (m, 1H, 1-H), 4.23–4.20 (m, 1H, 5-H), 3.90 (t, *J* = 8.7 Hz, 1H, 4′-H), 3.84 (dd, *J* = 1.8, 11.9 Hz, 1H, 6′-H_a), 3.80–3.77 (m, 2H, 3′-H, 5′-H), 3.73 (dd, *J* = 4.6, 11.9 Hz, 1H, 6′-H_b), 3.73 (t, *J* = 2.6 Hz, 1H, 2′-H); ^{13}C NMR (150 MHz, $CDCl_3$): *δ* 166.5 (C=O), 138.0 (C), 137.9 (C), 137.7 (C), 133.5 (CH), 130.0 (CH), 129.4 (C), 128.6 (CH), 128.5 (CH), 128.4 (CH), 128.1 (CH), 128.1 (CH), 128.0 (CH), 127.9 (CH), 127.8 (CH), 102.6 (CH), 99.9 (CH), 78.2 (CH), 75.5 (CH), 74.6 (CH_2), 74.5 (CH), 73.9 (CH), 73.5 (CH), 73.3 (CH_2), 72.9 (CH_2), 72.0 (CH), 70.4 (CH), 67.7 (CH), 67.57 (CH × 2), 62.3 (CH_2); HRMS (ESI): *m/z* calcd for $C_{41}H_{42}O_{12}Na$ ($[M + Na]^+$): 749.2574, found: 749.2575.

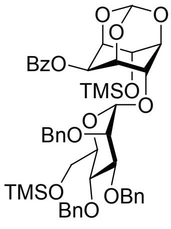

S5

2-*O*-Benzoyl-4-*O*-(2,3,4-tri-*O*-benzyl-6-*O*-trimethylsilyl-*α*-D-mannopyranosyl)-6-*O*-trimethylsilyl-D-*myo*-inositol-1,3,5-orthoformate (S5). Compound **S5** was prepared from compound **S4** by using the same procedure applied in generating compound **S2**. $[\alpha]^{27}_{D}$ +31.8 (*c* 2.6, $CHCl_3$); ^{1}H NMR (600 MHz, $CDCl_3$): *δ* 8.14 (dd, *J* = 7.8, 0.8 Hz, 2H, Ar-H), 7.57 (t, *J* = 7.8 Hz, 1H, Ar-H), 7.45 (t, *J* = 7.8 Hz, 2H, Ar-H), 7.35–7.25 (m, 15H, Ar-H), 5.54 (d, *J* =

0.9 Hz, 1H, orthoformate-H), 5.48 (d, *J* = 1.4 Hz, 1H, 2-H), 5.05 (d, *J* = 4.6 Hz, 1H, 1′-H), 4.89 (d, *J* = 11.2 Hz, 1H, PhC*H*$_2$), 4.81 (d, *J* = 12.1 Hz, 1H, PhC*H*$_2$), 4.68 (d, *J* = 11.5 Hz, 1H, PhC*H*$_2$), 4.66 (d, *J* = 11.5 Hz, 1H, PhC*H*$_2$), 4.64 (d, *J* = 12.1 Hz, 1H, PhC*H*$_2$), 4.60 (d, *J* = 11.2Hz, 1H, PhC*H*$_2$), 4.56–4.55 (m , 1H, 1-H), 4.55–4.53 (m, 1H, 6-H), 4.42–4.41 (m, 1H, 3-H), 4.27–4.26 (m, 1H, 1-H), 4.24–4.22 (m, 1H, 5-H), 3.95 (t, *J* = 9.3 Hz, 1H, 3′-H), 3.90 (dd, *J* = 9.3 Hz, 3.0 Hz, 1H, 4-H), 3.80–3.78 (m, 2H, 6′-H × 2), 3.77–3.76 (m, 1H, 2′-H), 3.67 (dt, *J* = 9.3, 3.0 Hz, 1H, 5′-H), 0.11 (s, 9H, Si(C*H*$_3$)$_3$) 0.05 (s, 9H, Si(CH$_3$)$_3$); ^{13}C NMR (150 MHz, CDCl$_3$): δ 165.7 (C=O), 138.5 (C), 138.2 (C), 138.2 (C), 133.0 (CH), 129.7 (CH), 129.7 (C), 128.2 (CH), 128.09 (CH), 128.07 (CH), 127.54 (CH), 127.51 (CH), 127.47 (CH), 127.4 (CH), 127.34 (CH), 127.29 (CH), 127.2 (CH), 102.8 (CH), 97.2 (CH), 80.0 (CH), 75.3 (CH), 74.5 (CH$_2$), 74.3 (CH), 73.7 (CH), 72.7 (CH$_2$), 72.4 (CH$_2$), 72.1 (CH), 71.3 (CH), 70.9 (CH), 69.1 (CH), 67.7 (CH), 63.8 (CH), 61.8 (CH$_2$), –0.2 (CH$_3$), –0.6 (CH$_3$); HRMS (ESI): *m*/*z* calcd for $C_{47}H_{58}O_{12}NaSi_2$ ([M + Na]$^+$): 893.3365, found: 893.3358.

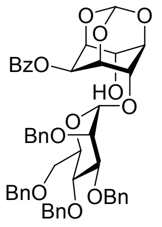

S6

2-*O*-Benzoyl-4-*O*-(2,3,4,6-tetra-*O*-benzyl-*α*-D-mannopyranosyl)-D-*myo*-inositol-1,3,5-orthoformate (S6). Compound **S6** was prepared from compound **S5** by using the same procedure applied in generating compound **S3**.

^{1}H NMR (600 MHz, CDCl$_3$) (δ, ppm)	
Obtained	Literature[2]
8.12 (dd, *J* = 8.3, 1.1 Hz, 2H, Ar-H)	8.12 (dd, *J* = 8.2, 0.9 Hz, 2H, Ar-H)
7.56 (t, *J* = 7.5 Hz, 1H, Ar-H)	7.58–7.55 (m, 1H, Ar-H)
7.44 (t, *J* = 7.9 Hz, 2H, Ar-H)	7.44 (t, *J* = 7.9 Hz, 2H, Ar-H)
7.34–7.16 (m, 20H, Ar-H)	7.58–7.17 (m, 20 H, Ar-H)
5.52 (d, *J* = 0.8 Hz, 1H)	5.53 (d, *J* = 1.0 Hz, 1H, orthoformate-H)
5.40 (d, *J* = 1.1 Hz, 1H)	5.41 (d, *J* = 1.3 Hz, 1H, 2-H),
4.97 (d, *J* = 2.6 Hz, 1H)	4.98 (d, *J* = 2.6 Hz, 1H, 1′-H)
4.73 (d, *J* = 11.0 Hz, 1H, PhC*H*$_2$)	4.74 (d, *J* = 10.9 Hz, 1H, PhC*H*$_2$)
4.72 (d, *J* = 12.1 Hz, 1H, PhC*H*$_2$)	4.72 (d, *J* = 12.1 Hz, 1H, PhC*H*$_2$)

4.68 (d, *J* = 11.8 Hz, 1H, PhC*H*$_2$)	4.68 (d, *J* = 12.4 Hz, 1H, PhC*H*$_2$)
4.61–4.60 (m, 1H)	4.62–4.61 (m, 1H, Ins-H)
4.58 (d, *J* = 11.8 Hz, 1H, PhC*H*$_2$)	4.59 (d, *J* = 11.8 Hz, 1H, PhC*H*$_2$)
4.56 (d, *J* = 12.1 Hz, 1H, PhC*H*$_2$)	4.57 (d, *J* = 12.1 Hz, 1H, PhC*H*$_2$)
4.54 (d, *J* = 12.2 Hz, 1H, PhC*H*$_2$)	4.55 (d, *J* = 12.2 Hz, 1H, PhC*H*$_2$)
4.50–4.49 (m, 2H, 2 Ins-H)	4.51–4.49 (m, 2H, 2 Ins-H)
4.44 (d, *J* = 11.9 Hz, 1H, PhC*H*$_2$)	4.45 (d, *J* = 12.9 Hz, 1H, PhC*H*$_2$)
4.42 (d, *J* = 12.2 Hz, 1H, PhC*H*$_2$)	4.43 (d, *J* = 12.2 Hz, 1H, PhC*H*$_2$)
4.40–4.39 (m, 1H, Ins-H)	4.41–4.39 (m, 1H, Ins-H)
4.23–4.22 (m, 1H, Ins-H)	4.24–4.23 (m, 1H, Ins-H)
3.93 (dd, *J* = 9.0, 8.0 Hz, 1H, 4′-H)	3.94 (t, *J* = 8.1 Hz, 1H, 4′-H)
3.83 (ddd, *J* = 9.0, 4.9, 1.8 Hz, 1H, 5′-H)	3.84 (ddd, *J* = 8.1, 4.9, 1.8, Hz, 1H, 5′-H)
3.76 (dd, *J* = 8.0, 2.8 Hz, 1H, 3′-H)	3.77 (dd, *J* = 8.1, 2.6 Hz, 1H, 3′-H)
3.69 (dd, *J* = 10.8, 4.9 Hz, 1H, 6′-H_a)	3.67 (dd, *J* = 10.8, 4.9 Hz, 1H, 6′-H_a)
3.63 (dd, *J* = 10.8, 1.8 Hz, 1H, 6′-H_b)	3.64 (dd, *J* = 10.8, 1.8 Hz, 1H, 6′-H_b)
3.57 (t, *J* = 2.8 Hz, 1H, 2′-H)	3.58 (t, *J* = 2.6 Hz, 1H, 2′-H)
2.83 (d, *J* = 9.3 Hz, 1H, OH)	

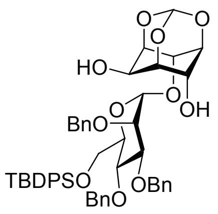

19

6-*O*-(2,3,4-Tri-*O*-benzyl-*α*-D-mannopyranosyl)-D-*myo*-inositol-1,3,5-orthoformate (19). NaOMe was added to a solution of compound **17** (1 g, 1.036 mmol) in a CH_2Cl_2/MeOH (1/1, 10 mL) mixed solvent. After stirring for 16 h, the reaction was neutralized by Dowex-IR resin and filtered. The filtrate was concentrated under reduced pressure, and the residue was purified by flash column chromatography (ethyl acetate/hexanes = 1/1.5) to get the diol **19** (0.892 mg, quant.). $[\alpha]^{25}_{D}$ +42.4 (*c* 3.7, $CHCl_3$); IR (thin film): *ν* 3448, 3065, 2927, 2862, 1589, 1494, 1454, 1305, 1213, 1164, 1008, 992, 807, 742, 699, 611 cm^{-1}; ^1H NMR (600 MHz, $CDCl_3$): *δ* 7.69 (t, *J* = 7.5 Hz, 4H, Ar-H), 7.43 (t, *J* = 7.5 Hz, 2H, Ar-H), 7.39–7.30 (m, 14H, Ar-H), 7.26–7.25 (m, 3H, Ar-H), 7.13–7.12 (m, 2H, Ar-H), 5.43 (s, 1H, orthoformate-H), 4.79–4.76 (m, 3H, 1′-H, PhC*H*$_2$), 4.71 (d, *J* = 11.8 Hz, 1H, PhC*H*$_2$), 4.61 (d, *J* = 11.8 Hz, 1H, PhC*H*$_2$), 4.57–4.53 (m, 2H, 6-H, PhC*H*$_2$), 4.47 (d, *J* = 10.8 Hz, 1H, PhC*H*$_2$), 4.37–4.42

(m, 1H, 4-H), 4.25–4.23 (m, 1H, 5-H), 4.17 (m, 1H, 3-H), 4.07–4.05 (m, 1H, 1-H), 3.92 (m, 4H, 2-H, 4′-H, 6′-H × 2), 3.75–3.72 (m, 1H, 5′-H), 3.70 (dd, J = 2.5, 8.1 Hz, 1H, 3′-H), 3.49 (t, J = 2.5 Hz, 1H, 2′-H), 1.07 (s, 3H, CH_3); ^{13}C NMR (150 MHz, $CDCl_3$): δ 137.9 (C), 137.8 (C), 137.7 (C), 135.7 (CH), 135.6 (CH), 133.3 (C), 133.0 (C), 129.7 (CH), 128.6 (CH), 128.5 (CH), 128.4 (CH), 128.1 (CH), 128.0 (CH), 127.9 (CH), 127.8 (CH), 127.7 (CH), 127.6 (CH), 102.7 (CH), 98.1 (CH), 78.3 (CH), 75.8 (CH), 74.6 (CH_2), 74.38 (CH), 74.35 (CH), 74.3 (CH), 73.4 (CH_2), 73.0 (CH_2), 71.7 (CH), 71.6 (CH), 68.3 (CH), 67.5 (CH), 63.3 (CH_2), 60.5 (CH), 26.7 (CH_3), 19.2 (C); HRMS (ESI): m/z calcd for $C_{50}H_{56}O_{11}SiNa$ ($[M + Na]^+$): 883.3490, found: 883.3496.

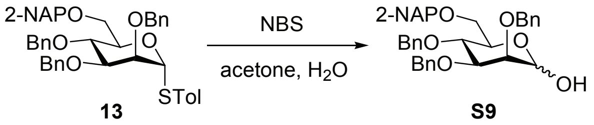

2,3,4-tri-*O*-benzyl-6-*O*-(2-naphthylmethyl)-D-mannopyranose (S9). The thioglycoside **13** (5.00 g, 7.17 mmol) was dissolved in acetone (70 mL) and the flask was immersed in an ice-water bath. NBS (1.92 g, 10.76 mmol) and water (0.78 mL, 43.06 mmol) were then added. After stirring in ice bath for 1 h, the reaction was quenched with 10% $Na_2S_2O_{3(aq)}$ (50 mL) and the solvents were evaporated under reduced pressure. The residue was dissolved in ethyl acetate and washed successively with 10% $Na_2S_2O_{3(aq)}$ and brine. The organic layer was dried over $MgSO_4$, filtered and concentrated reduced pressure. The residue was purified by flash column chromatography (ethyl acetate/hexanes = 1/3) to give the hemiacetal **S9** (3.89 g, 92%; α/β = 7/1). ^1H NMR (600 MHz, $CDCl_3$): δ 7.82–7.77 (32H), 7.50–7.09 (m, 144H), 5.23 (s, 7H), 5.07 (d, J = 11.4 Hz, 1H), 4.91–4.48 (m, 64H), 4.22–4.12 (m, 14H), 3.99–3.71 (m, 32H), 3.53–3.47 (m, 2H). ^{13}C NMR (150 MHz, $CDCl_3$): δ 138.42 (C), 138.31 (C), 138.19 (C), 138.11 (C), 137.99 (C), 135.55 (C), 135.38 (C), 133.17 (C), 132.96 (C), 128.50 (CH), 128.47 (CH), 128.30 (CH), 128.24 (CH), 128.19 (CH), 128.13 (CH), 127.87 (CH), 127.81 (CH), 127.66 (CH), 127.59 (CH), 127.50 (CH), 126.74 (CH), 126.07 (CH), 126.01 (CH), 125.81 (CH), 93.75 (CH), 92.70 (CH), 83.04 (CH), 79.70 (CH), 75.91 (CH), 75.16 (CH), 75.11 (CH_2), 75.00 (CH_2), 74.73 (CH), 74.58 (CH_2), 74.49 (CH), 73.60 (CH_2), 73.34 (CH_2), 72.67 (CH_2), 72.60 (CH_2), 72.08 (CH_2), 71.40 (CH), 69.58 (CH_2), 69.00 (CH_2); HRMS (ESI): m/z calcd for $C_{38}H_{38}O_6Na$ ($[M + Na]^+$): 613.2566, found: 613.2565.

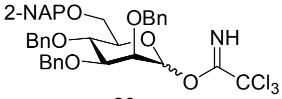

20

2,3,4-tri-*O*-benzyl-6-*O*-(2-naphthylmethyl)-*α*-D-mannopyranosyl trichloroacetimidate (20). To the solution of compound **S9** (0.95 g, 1.61 mmol) in CH_2Cl_2 (20 mL), CCl_3CN (1.61 mL, 16.1 mmol) and K_2CO_3 (1.11 g, 8.04 mmol) were sequentially added at room temperature under nitrogen atmosphere. After continuously stirring for 24 h, the reaction was filtered through Celite, the residue was washed with CH_2Cl_2, and the resulting organic solution was washed with water and brine. After drying with $MgSO_4$, the solution was concentrated under reduced pressure to afford the trichloroacetimidate **20** (1.16 g, 98%; *α*/*β* = 5/1), which was directly used for the next reaction. ^1H NMR (400 MHz, $CDCl_3$): *δ* 8.63 (s, 0.20 H), 8.53 (s, 1H), 7.81–7.07 (m, 25H), 6.38 (d, *J* = 1.72 Hz, 1H), 5.81 (d, *J* = 0.6 Hz, 1H), 4.89–4.51 (m, 9.2H), 4.18–4.13 (m, 1.20H), 4.10–3.66 (m, 6H).

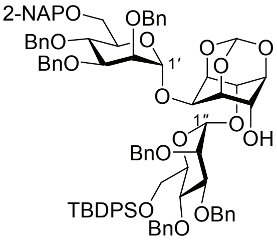

21

6-*O*-(2,3,4-Tri-*O*-benzyl-6-*O*-*tert*-butyldiphenylsilyl-*α*-D-mannopyranosyl)-2-*O*-[2,3,4-tri-*O*-benzyl-6-*O*-(2-naphthylmethyl)-*α*-D-mannopyranosyl]-D-*myo*-inositol-1,3,5-orthoformate (21). A mixture of diol **19** (0.908 g, 1.05 mmol), imidate **20** (0.775 g, 1.05 mmol) and 3 Å molecular sieves (3 g) in CH_2Cl_2 (50 mL) was stirred for 1 h at room temperature under nitrogen atmosphere. The reaction mixture was cooled to –60 °C, $BF_3 \cdot OEt_2$ (40 μL, 0.317 mmol) was then added, and the reaction temperature was gradually raised to –20 °C. After stirring for 1 h, an additional solution of imidate **20** (0.775 g, 1.05 mmol) in CH_2Cl_2 (5 mL) and $BF_3 \cdot OEt_2$ (40 μL, 0.317 mmol) were consecutively introduced and the resulting mixture was stirred at the same temperature for another 2 h. Et_3N was added to quench the reaction and the whole mixture was filtered through Celite. The filtrate was washed successively with saturated $NaHCO_{3(aq)}$ and brine. After drying over $MgSO_4$, the organic layer was concentrated under reduced pressure. The crude product was purified by flash column chromatography (ethyl acetate/hexanes = 1/3) to furnish the pseudotrisaccharide **21** (1.09 g, 72%). $[\alpha]^{23}_D$ +40.8 (*c* 1.6, $CHCl_3$); IR ($CHCl_3$): *ν* 3530, 3034, 2931, 2862, 1727,

1602, 1454, 1364, 1166, 1110, 1003, 951, 822, 742, 699 cm^{-1}; ^{1}H NMR (400 MHz, $CDCl_3$): δ 7.77–7.67 (m, 8H, Ar-H), 7.47–7.04 (m, 39H, Ar-H), 5.46 (d, J = 1.6 Hz, 1H, orthoformate-H), 5.11 (d, J = 2.9 Hz, 1′-H), 4.89–4.23 (m, 17H, 1″-H, ArCH*H* × 14, 3-H, 6-H), 4.37–4.32 (m, 1H, 4-H), 4.27–4.25 (m, 1H, 5-H), 4.10–4.00 (m, 6H, 1-H, 2-H, 2′-H, 3′-H, 4′-H, 5′-H), 3.90–3.71 (m, 7H, 3″-H, 4″-H, 5″-H, 6′-H × 2, 6″-H × 2), 3.59 (t, J = 4.2 Hz, 1H, 2″-H), 3.04 (s, 1H, OH), 1.06 (s, 9H); ^{13}C NMR (100 MHz, $CDCl_3$): δ 138.5 (C), 138.4 (C), 138.2 (C), 138.0 (C × 2), 137.8 (C), 135.7 (CH), 135.6 (C, CH), 133.3 (C), 133.2 (C), 133.0 (C), 132.9 (C), 129.6 (CH), 128.5 (CH), 128.4 (CH), 128.3 (CH), 128.2 (CH), 128.0 (CH), 127.9 (CH), 127.8 (CH), 127.72 (CH), 127.66 (CH), 127.6 (CH), 127.5 (CH), 126.3 (CH), 126.0 (CH), 125.8 (CH), 125.7 (CH), 102.6 (CH), 98.6 (CH), 98.1 (CH), 80.2 (CH), 78.6 (CH), 76.0 (CH), 75.6 (CH), 75.0 (CH), 74.9 (CH_2), 74.5 (CH_2), 74.4 (CH), 74.3 (CH), 73.3 (CH_2 × 2), 73.1 (CH_2), 72.9 (CH), 72.8 (CH_2), 72.5 (CH_2), 72.4 (CH), 72.2 (CH), 69.3 (CH_2), 69.1 (CH), 68.9 (CH), 67.7 (CH), 66.0 (CH), 63.4 (CH_2), 26.7 (CH_3), 19.1 (C); HRMS (ESI): *m*/*z* calcd for $C_{88}H_{92}O_{16}NaSi$ ($[M + Na]^+$): 1455.6052, found: 1455.6057.

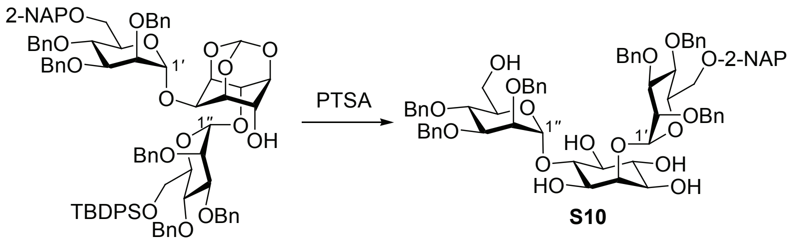

21

6-*O*-(2,3,4-tri-*O*-Benzyl-*α*-D-mannopyranosyl)-2-*O*-[2,3,4-tri-*O*-benzyl-6-*O*-(2-naphthylmethyl)-*α*-D-mannopyranosyl]-D-*myo*-inositol (S10). To a solution of compound **21** (1.23 g, 0.86 mmol) in a CH_2Cl_2/MeOH (1/1, 20 mL) mixed solvent at room temperature, *p*-toluenesulfonic acid monohydrate (PTSA·H_2O, 491 mg, 2.58 mmol) was added. After stirring for 20 h, Et_3N (0.3 mL) was added to quench the reaction and the solution was concentrated under reduced pressure. Purification of the crude residue via flash column chromatography (ethyl acetate/hexanes = 2/1) gave the pentaol **S10** (854 g, 84%). $[\alpha]^{23}_{D}$ +33.1 (*c* 3.3, $CHCl_3$); IR (thin film): *ν* 3448, 3030, 2862, 2927, 1495, 1456, 1088, 1047, 745, 697 cm^{-1}; ^{1}H NMR (600 MHz, $CDCl_3$): δ 7.73–7.67 (m, 4H, Ar-H), 7.40–7.37 (m, 3H, Ar-H), 7.28–7.18 (m, 24H, Ar-H), 7.13–7.06 (m, 4H, Ar-H), 7.02–7.01 (m, 2H, Ar-H), 5.07 (d, J = 2.7 Hz, 1H, 1′-H), 5.01 (d, J = 3.0 Hz, 1H, 1″-H), 4.68–4.51 (m, 11H, ArC*H*$_2$), 4.49 (d, J = 11.8 Hz, 1H, ArC*H*$_2$), 4.44 (d, J = 11.2 Hz, 1H, ArC*H*$_2$), 4.35 (d, J = 11.0 Hz, 1H, ArC*H*$_2$), 4.06–3.96 (m, 1H, 5″-H), 3.95 (t, J = 2.4 Hz, 1H, 2-H), 3.93–3.90 (m, 1H, 5′-H), 3.81 (dd, J =

2.8, 7.1 Hz, 1H, 3″-H), 3.78–3.73 (m, 4H, 2″-H, 3″-H, 4″-H, 6″-H_a), 3.69–3.67 (m, 2H, 2′-H, 4′-H), 3.64–3.58 (m, 3H, 6′-H × 2, 6″-H_b), 3.53–3.50 (m, 1H, 4-H), 3.45–3.42 (m, 1H, 6-H), 3.71–3.36 (m, 1H, 1-H), 3.20–3.17 (m, 2H, 3-H, 5-H), 2.94 (bs, 1H, OH), 2.40 (bs, 1H, OH), 2.18 (bs, 2H, OH); ^{13}C NMR (150 MHz, $CDCl_3$): δ 138.2 (C × 2), 137.93 (C), 137.86 (C), 137.78 (C), 137.75 (C), 135.3 (C), 133.2 (C), 132.9 (C), 128.4 (CH), 128.24 (CH), 128.15 (CH), 128.04 (CH), 127.96 (CH), 127.95 (CH), 127.9 (CH), 127.82 (CH), 127.79 (CH), 127.7 (CH), 127.6 (CH), 126.7 (CH), 126.0 (CH), 125.9 (CH), 125.8 (CH), 99.9 (CH), 99.8 (CH), 83.2 (CH), 80.0 (CH), 78.0 (CH × 2), 76.1 (CH), 75.4 (CH), 75.2 (CH), 75.0 (CH), 74.4 (CH_2), 74.1 (CH_2), 73.4 (CH_2), 73.23 (CH), 73.19 (CH), 73.15 (CH), 72.6 (CH_2), 72.3 (CH_2), 72.2 (CH_2 × 2), 72.1 (CH), 72.0 (CH), 70.8 (CH), 69.3 (CH_2), 62.4 (CH_2); HRMS (ESI): *m*/*z* calcd for $C_{71}H_{76}O_{16}Na$ ($[M + Na]^+$): 1207.5031, found: 1207.5035.

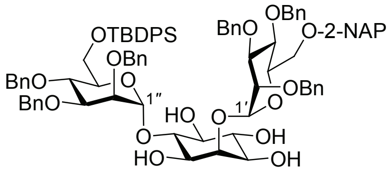

22

6-*O*-(2,3,4-tri-*O*-Benzyl-6-*O*-*tert*-butyldiphenylsilyl-*α*-D-mannopyranosyl)-2-*O*-[2,3,4-tri-*O*-benzyl-6-*O*-(2-naphthylmethyl)-*α*-D-mannopyranosyl]-D-*myo*-inositol (22). Compound **S10** (1.40 g, 1.81 mmol) and DMAP were dissolved in CH_2Cl_2 (20 mL), and the mixture was cooled in an ice-water bath. Et_3N (987 μL, 7.08 mmol) and *t*-butyldiphenylchlorosilane (613 μL, 2.36 mmol) were added under nitrogen atmosphere. The ice-water bath was removed and stirring was continued at room temperature. After 2 d, the reaction was diluted with CH_2Cl_2 and washed successively with saturated $NH_4Cl_{(aq)}$ and brine. The organic solution was dried over $MgSO_4$ and concentrated under reduced pressure. Purification of the residue by flash column chromatography delivered the tetraol **22** (1.55, 82%). $[\alpha]^{23}_D$ +23.1 (*c* 0.5, $CHCl_3$); ^{1}H NMR (400 MHz, $CDCl_3$): δ 7.81–7.76 (m, 4H, Ar-H), 7.68–7.64 (m, 4H, Ar-H), 7.48–7.11 (m, 35H, Ar-H), 7.07–7.02 (m, 4H, Ar-H), 5.15 (d, *J* = 2.3 Hz, 1H, 1″-H), 5.01 (d, *J* = 3.0 Hz, 1H, 1′-H), 4.78–4.60 (m, 11H, ArC*H*₂), 4.54 (d, *J* = 11.8 Hz, 1H, ArCH_2), 4.41 (d, *J* = 11.0 Hz, 1H, ArCH_2), 4.37 (d, *J* = 11.0 Hz, 1H, ArCH_2), 4.14–4.05 (m, 2H, 5″-H, 6-H), 3.99–3.96 (m, 1H, 5′-H), 3.90–3.77 (m, 6H, 3′-H, 4′-H, 6′-H × 2, 3″-H, 4″-H), 3.73–3.68 (m, 4H, 2′-H, 2″-H, 6″-H × 2), 3.5 (td, *J* = 1.1, 9.1 Hz, 1H, 4-H), 3.56–3.46 (m, 1H, 1-H), 3.43–3.35 (m, 2H, 2-H, 5-H), 3.23 (td, *J* = 1.8, 9.1 Hz, 1H, 3-H); 1.04 (s, 9H, *t*Bu); ^{13}C NMR (150 MHz, $CDCl_3$): δ 138.2 (C), 138.1 (C), 138.0 (C), 137.89

(C), 137.85 (C), 137.8 (C), 135.7 (CH), 135.5 (C, CH), 133.2 (C), 133.1 (C), 132.9 (C), 132.8 (C), 129.7 (CH), 128.4 (CH), 128.3 (CH), 128.2 (CH), 128.1 (CH), 128.0 (CH), 127.94 (CH), 127.91 (CH), 127.9 (CH), 127.8 (CH), 127.7 (CH), 127.5 (CH), 126.6 (CH), 125.9 (CH), 125.7 (CH), 99.8 (CH), 99.4 (CH), 84. 9 (CH), 79.4 (CH), 78.3 (CH), 77.9 (CH), 76.4 (CH), 75.3 (CH), 75.0 (CH), 74.7 (CH), 74.4 (CH_2), 74.34 (CH), 74.25 (CH_2), 73.4 (CH_2), 73.3 (CH), 73.0 (CH), 72.7 (CH_2), 72.5 (CH_2), 72.4 (CH_2), 72.2 (CH_2, CH), 71.2 (CH), 70.8 (CH), 69.2 (CH_2), 63.8 (CH_2), 26.7 (CH_3), 19.1 (CH); HRMS (ESI): *m*/*z* calcd for $C_{87}H_{94}O_{16}NaSi$ ($[M + Na]^+$): 1445.6209, found: 1445.6201.

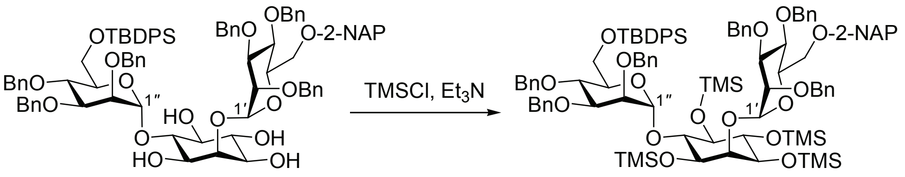

22 **S11**

6-*O*-(2,3,4-Tri-*O*-benzyl-6-*O*-*tert*-butyldiphenylsilyl-*α*-D-mannopyranosyl)-2-*O*-[2,3,4-tri-O-benzyl-6-*O*-(2-naphthylmethyl)-*α*-D-mannopyranosyl]-1,3,4,5-tetrakis-*O*-trimethylsilyl-D-*myo*-inositol (S11). Et_3N (2.17 mL, 23 mmol) was added to a solution of the tetraol **22** (1 g, 0.62 mmol) in CH_2Cl_2 (20 mL) at room temperature under nitrogen. The reaction flask was immersed in an ice-water bath, TMSCl (1.6 mL, 12.5 mmol) was slowly added to the solution, and the mixture was gradually warmed up to room temperature. After stirring for 36 h, the solution was concentrated under reduced pressure, the resulting mass was suspended in hexane (30 mL), and the whole mixture was filtered through Celite. The filtrate was concentrated under reduced pressure to afford compound **S11** (1.06 g, quant.). $[\alpha]^{23}_D$ +21.1 (*c* 12.1, $CHCl_3$); IR ($CHCl_3$): *ν* 3030, 2953, 2850, 1634, 1497, 1454, 1363, 1252, 1111, 1028, 918, 843, 746, 697 cm^{-1}; ^1H NMR (600 MHz, $CDCl_3$): δ 7.78–7.76 (m, 1H, Ar-H), 7.71–7.54 (m, 7H, Ar-H), 7.43–7.07 (m, 39H, Ar-H), 5.51 (s, 1H, 1′-H), 5.41 (s, 1H, 1″-H), 4.99 (d, *J* = 12.0 Hz, 1H, ArCH_2), 4.92 (d, *J* = 11.6 Hz, 1H, ArCH_2), 4.87 (d, *J* = 11.8 Hz, 1H, ArCH_2), 4.81 (d, *J* = 12.2 Hz, 1H, ArCH_2), 4.73–4.64 (m, 5H, ArCH_2), 4.57 (d, *J* = 12.2 Hz, 1H, ArCH_2), 4.47 (d, *J* = 12.0 Hz, 1H, ArCH_2), 4.40 (d, *J* = 11.8 Hz, 1H, ArCH_2), 4.34 (d, *J* = 9.5 Hz, 1H, ArCH_2), 4.27 (d, *J* = 11.8 Hz, 1H, ArCH_2), 4.18–4.10 (m, 4H, 3″-H, 4″-H, 5″-H, 5′-H), 4.00 (dd, *J* = 11.1, 3.1 Hz, 1H, 6″-H_a), 3.94–3.85 (m, 7H, 2″-H, 6″-H_b, 2′-H, 3′-H, 4′-H, 2-H, 6-H), 3.78 (dd, *J* = 10.7, 3.2 Hz, 1H, 6′-H_a), 3.65 (d, *J* = 10.7 Hz, 1H, 6′-H_b), 3.57–3.54 (m, 2H, 1-H, 4-H); 3.21 (dd, *J* = 9.4, 2.2 Hz, 1H, 5-H), 3.12 (t, *J* = 9.4 Hz, 1H, 3-H), 0.95 (s, 3H), 0.14 (s, 9H, Si(CH_3)$_3$), 0.12 (s, 9H, Si(CH_3)$_3$), 0.06 (s, 9H,

Si(CH_3)$_3$), –0.002 (s, 9H, Si(CH_3)$_3$); ^{13}C NMR (100 MHz, $CDCl_3$): δ 139.7 (C × 2), 138.9 (C), 138.8 (C), 138.4 (C), 138.0 (C), 136.1 (C), 135.8 (CH), 135.6 (CH), 133.8 (C), 133.6 (C), 133.2 (C), 132.9 (C), 129.3 (CH), 128.3 (CH), 128.2 (CH), 128.1 (CH), 127.9 (CH), 127.9 (CH), 127.8 (CH), 127.8 (CH), 127.7 (CH), 127.6 (CH), 127.5 (CH), 127.4 (CH), 127.3 (CH), 127.2 (CH), 126.7 (CH), 126.3 (CH), 126.0 (CH), 125.8 (CH), 125.5 (CH), 99.1 (CH), 98.0 (CH), 79.7 (CH), 78.4 (CH), 77.5 (CH), 76.3 (CH), 75.8 (CH), 75.6 (CH), 74.9 (CH × 2), 74.3 (CH_2), 74.1 (CH), 73.9 (CH), 73.6 (CH_2), 73.5 (CH_2), 73.0 (CH_2), 72.6 (CH_2, CH × 2), 72.3 (CH_2), 71.8 (CH), 71.7 (CH), 71.1 (CH_2), 69.5 (CH_2), 63.2 (CH_2), 26.8 (CH_3), 19.3 (CH), 1.3 (CH_3), 1.2 (CH_3), 0.35 (CH_3), 0.07 (CH_3); HRMS (ESI): *m*/*z* calcd for $C_{99}H1_{26}O_{16}NaSi_5$ ([M + Na]$^+$): 1733.7790, found: 1733.7797.

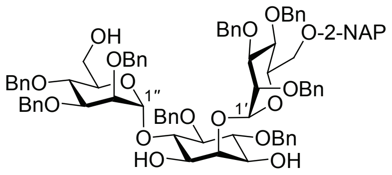

2

3,4-Di-*O*-benzyl-6-*O*-(2,3,4-tri-*O*-benzyl-α-D-mannopyranosyl)-2-*O*-[2,3,4-tri-*O*-benzyl-6-*O*-(2-naphthylmethyl)-*α*-D-mannopyranosyl]-D-*myo*-inositol (2). Compound **S11** (500 mg, 0.29 mmol) and freshly flame dried 3 Å molecular sieves (500 mg) in CH_2Cl_2 (10 mL) was stirred at room temperature for 1 h under nitrogen. The reaction flask was cooled down to –40 °C, and benzaldehyde (62 µL, 0.61 mmol) was added to the mixture. After stirring for 5 min, Et_3SiH (117 µL, 0.73 mmol) and TMSOTf (19 µL, 85 µmol) were consecutively added and the resulting solution was continuously stirred for 48 h. TBAF (1 M in THF, 0.9 mL, 0.9 mmol) was added to the mixture, the reaction flask was warmed up to room temperature, and the solution was stirred for 12 h. The whole mixture was filtered through Celite, the filtrate was diluted with CH_2Cl_2 (10 mL) and washed with water and brine. The mixture was dried over $MgSO_4$ and concentrated under reduced pressure. The residue was purified by flash column chromatography (ethyl acetate/hexanes = 1/2) to get the desired compound **2** (311 mg, 72%). $[\alpha]^{23}_D$ +53.9 (*c* 2.2, $CHCl_3$); IR (thin film): *ν* 3461, 3065, 2931, 2866, 1495, 1453, 1362, 1208, 1116, 735, 699 cm^{-1}; ^{1}H NMR (600 MHz, $CDCl_3$): δ 7.78–7.69 (m, 4H, Ar-H), 7.46–7.12 (m, 41H, Ar-H), 7.04 (d, *J* = 7.3 Hz, 2H, Ar-H), 5.42 (s, 1H, 1′-H), 4.99 (d, *J* = 3.7 Hz, 1H, 1″-H), 4.90 (d, *J* = 11.1 Hz, 1H, ArCH_2), 4.85 (d, *J* = 10.8 Hz, ArCH_2), 4.77–4.51 (m, 15H, ArCH_2), 4.49 (d, *J* = 12.2 Hz, 1H, ArCH_2), 4.44 (d, *J* = 10.8 Hz, 1H, ArCH_2), 4.30 (t, *J* = 2.2 Hz, 1H, 2-H), 4.15–4.12 (m, 1H, 5″-H), 4.08 (t. *J* = 9.5 Hz, 1H,

4″-H), 3.98–3.95 (m, 1H, 5′-H), 3.88 (dd, *J* = 2.7, 6.2 Hz, 1H, 3′-H), 3.83–3.76 (m, 4H, 2″-H, 3″-H, 4′-H, 6′-H_a), 3.72–3.69 (m, 2H, 2′-H, 6′-H_b), 3.60 (t, *J* = 9.2 Hz, 1H, 4-H), 3.57 (dd, *J* = 3.9, 10.8 Hz, 1H, 6″-H_a), 3.90 (t, *J* = 9.2 Hz, 1H, 6-H), 3.41–3.38 (m, 2H, 1-H, 6″-H_b), 3.34 (t, *J* = 9.2 Hz, 1H, 5-H), 3.27 (dd, *J* = 2.2, 9.2 Hz, 1H, 3-H); ^{13}C NMR (150 MHz, $CDCl_3$): δ 138.6 (C), 138.44 (C), 138.41 (C), 138.1 (C), 138.03 (C), 137.96 (C), 137.9 (C), 137.6 (C), 135.7 (C), 133.2 (C), 132.9 (C), 128.5 (CH), 128.5 (CH), 128.4 (CH), 128.4 (CH), 128.3 (CH), 128.3 (CH), 128.1 (CH), 128.1 (CH), 128.0 (CH), 127.92 (CH), 127.88 (CH), 127.85 (CH), 127.6 (CH), 127.6 (CH), 127.4 (CH), 127.3 (CH), 127.1 (CH), 126.7 (CH), 126.1 (CH), 125.9 (CH), 125.7 (CH), 100.0 (CH), 98.6 (CH), 83.1 (CH), 80.3 (CH), 79.0 (CH), 78.2 (CH), 76.6 (CH), 75.4 (CH), 75.3 (CH_2), 75.0 (CH_2), 74.6 (CH), 74.2 (CH), 73.8 (CH_2), 73.7 (CH), 73.5 (CH_2), 73.2 (CH), 73.1 (CH), 72.6 (CH_2), 72.5 (CH), 72.4 (CH_2), 72.1 (CH_2), 71.8 (CH_2), 71.7 (CH), 71.6 (CH_2), 68.8 (CH_2), 62.7 (CH_2); HRMS (ESI): *m*/*z* calcd for $C_{85}H_{88}O_{16}Na$ ([M + Na]$^+$): 1387.5970, found: 1387.5983.

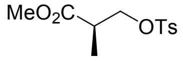

24

Methyl 2-*(R)*-methyl-3-[(4-methylbenzenesulfonyl)oxy]propanoate (24). $[\alpha]^{23}_D$ +3.3 (*c* 6.8, $CHCl_3$); IR ($CHCl_3$): ν 2956, 1740, 1598, 1463, 1362, 1179, 1097, 974, 817, 750, 665, 572, 555 cm^{-1}; ^{1}H NMR (400 MHz, $CDCl_3$): δ 7.72 (d, *J* = 8.4 Hz, 2H, Ar-H), 7.30 (d, *J* = 8.4 Hz, 2H, Ar-H), 4.13 (dd, *J* = 6.8, 9.6 Hz, 1H, CH_2), 4.01 (dd, *J* = 6.0, 9.6 Hz, 1H, CH_2), 3.57 (s, 3H, -$COOCH_3$), 2.78–2.71 (m, 1H, CH), 2.39 (s, 3H, CH_3), 1.11 (d, *J* = 7.2 Hz, 3H, CH_3); ^{13}C NMR (100 MHz, $CDCl_3$): δ 172.9 (C=O), 144.8 (C), 132.6 (C), 129.7 (CH), 127.8 (CH), 70.7 (CH_2), 51.9 (CH_3), 39.0 (CH), 21.5 (CH_3), 13.4 (CH_3); HRMS (ESI): *m*/*z* calcd for $C_{12}H_{17}O_5S$ ([M + H]$^+$): 273.0797, found: 273.0805.

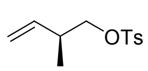

25

3-*(S)*-Methyl-4-[(4-methylbenzenesulfonyl)oxy]but-1-ene (25). To a solution of compound **23** (20 g, 0.17 mol) in CH_2Cl_2 at 0 °C, Et_3N (28 mL, 0.20 mol), DMAP (4 g, 0.03 mol), and tosyl chloride (39 g, 0.20 mol) were successively added. The mixture was stirred overnight at room temperature. The reaction was quenched with water, followed by extraction with CH_2Cl_2. The combined organic layers were washed with $NaHCO_{3(aq)}$ and brine, dried over $MgSO_4$, filtered through a pad of silica and concentrated under reduced

pressure to afford compound **24** (43.3 g, 94%) as a colorless oil.

To a solution of compound **24** (10 g, 36.72 mmol) in toluene (100 mL) at –78 °C, diisobutylaluminium hydride (1.2 M in toluene, 36.7 mL, 44.1 mmol) was added dropwise over a period of 30 min under nitrogen atmosphere. After stirring at the same temperature for 1 h, the reaction was quenched by adding ethyl acetate, and then warmed up to room temperature. A saturated aqueous solution of Rochelle salt (100 mL) was added and the biphasic mixture was vigorously stirred until the mixture became clear. The subsequent extraction with ethyl acetate led to an organic solution that was washed successively with water and brine, dried over $MgSO_4$, filtered and concentrated under reduced pressure to afford the aldehyde product, which was used in the next step without further purification.

The *n*-butyllithium (1.6 M in hexanes, 34.4 mL) was added to the suspension of methyltriphenylphosphonium bromide (15.7 g, 44.1 mmol) in THF (250 mL) at 0 °C under nitrogen atmosphere. After 30 min of stirring, a solution of the synthesized aldehyde in THF (20 mL) was added to the reaction mixture through a syringe pump for over 30 min. Then, the reaction was warmed up to room temperature and the stirring was continued overnight. The reaction was quenched with aqueous ammonium chloride, followed by extraction with ethyl acetate. The organic layer was washed with water and brine, dried over $MgSO_4$, and concentrated under reduced pressure. Purification by flash column chromatography supplied the olefin **25** (5.94 g, 72%). $[\alpha]^{23}_{D}$ +3.4 (*c* 2.5, $CHCl_3$) [lit.[5] $[\alpha]^{23}_{D}$ +4.0 (*c* 2.17, $CHCl_3$)]; IR (thin film): *ν* 2983, 1600, 1361, 1178, 1094, 967, 815, 663, 573, 555 cm^{-1}; ^{1}H NMR (400 MHz, $CDCl_3$): δ 7.76 (d, *J* = 8.4 Hz, 2H, Ar-H), 7.32 (d, *J* = 8.4 Hz, 2H, Ar-H), 5.61 (ddd, *J* = 7.0, 10.4, 17.3 Hz, 1H, C*H*=CH$_2$), 5.03 (dt, *J* = 1.5, 8.9 Hz, 1H, CH=C*H*$_2$), 5.01–4.99 (m, 1H, CH=C*H*$_2$), 3.90 (dd, *J* = 6.3, 9.4 Hz, 1H, CH_2), 3.82 (dd, *J* = 6.9, 9.4 Hz, 1H, CH_2), 2.53–2.46 (m, 1H, CH), 2.43 (s, 3H, CH_3), 0.99 (d, *J* = 6.7 Hz, 3H, CH_3); ^{13}C NMR (100 MHz, $CDCl_3$): δ 144.6 (C), 138.2 (CH), 132.9 (C), 129.7 (CH), 127.7 (CH), 115.8 (CH_2), 73.8 (CH_2), 36.8 (CH), 21.4 (CH_3), 15.8 (CH_3); HRMS (EI): *m*/*z* calcd for $C_{12}H_{16}O_3SNa$ ([M + Na]$^+$): 263.0710, found: 263.0718.

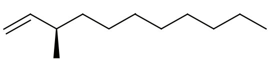

26

3-*(R)*-Methylundec-1-ene (26). To a solution of the tosylate **25** (3 g, 12.48 mmol) in THF (30 mL) at –78 °C, the *n*-heptylmagnesium bromide (30.6 mL, 43.7 mmol) was added dropwise followed by Li_2CuCl_4 (0.1 M in THF, 12.5 mL, 1.25 mmol). The reaction was gradually warmed up to 0 °C over a period of 1 h and stirred at 0 °C for an additional 12 h.

The resulting mixture was then carefully poured into an ice-cooled saturated solution of ammonium chloride (50 mL) and the target compound was extracted with ethyl acetate. The combined organic layer was washed with water and brine, dried over $MgSO_4$, and concentrated under reduced pressure. Purification by flash column chromatography (hexane) furnished the olefin **26** (1.9 g, 92%). $[\alpha]^{27}_{D}$ –3.5 (*c* 4.0, $CHCl_3$); IR (thin film) *ν* 2926, 2116, 1464, 1378, 907, 718 cm^{-1}; ^1H NMR (600 MHz, $CDCl_3$): *δ* 5.67 (ddd, *J* = 7.8, 9.9, 17.3 Hz, 1H, C*H*=CH_2), 4.94–4.90 (m, 1H, CH=CH_2), 4.88–4.86 (m, 1H, CH=CH_2), 2.10–2.06 (m, 1H, CH), 1.29–1.23 (m, 14H), 0.95 (d, *J* = 6.72 Hz, 3H), 0.86 (t, *J* = 7.0 Hz, 3H); ^{13}C NMR (150 MHz, $CDCl_3$): *δ* 145.1 (CH), 112.2 (CH_2), 37.8 (CH), 36.7 (CH_2), 31.9 (CH_2), 29.8 (CH_2), 29.70 (CH_2), 29.66 (CH_2), 29.6 (CH_2), 29.4 (CH_2), 29.3 (CH_2), 27.3 (CH_2), 22.7 (CH_2), 20.2 (CH_3), 14.1 (CH_3); HRMS (EI): *m*/*z* calcd for $C_{12}H_{24}$ ($[M]^+$): 168.1878, found: 168.1879.

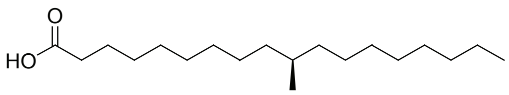

28

10-*(R)*-Methylundecanoic acid or tuberculostearic acid (28). Grubbs' 2nd generation catalyst (100 mg, 0.12 mmol) was added to the mixture of compound **26** (2.52 g, 14.9 mmol) and 8-nonenoic acid (**27**, 0.78 g, 4.98 mmol) in CH_2Cl_2, and the mixture was refluxed for 2 d. The solvent was evaporated under reduced pressure and the resulting crude residue was purified by flash column chromatography (ethyl acetate/hexanes = 1/5) to afford a long chain *E*/*Z* olefinic acid mixture.

The synthesized olefinic acid was then dissolved in ethanol (50 mL), palladium on charcoal (Pd/C) (100 mg, 10% Pd content) was added, and the resulting mixture was stirred under hydrogen atmosphere at room temperature for 12 h. The whole mixture was filtered through Celite and the filtrate was concentrated under reduced pressure. The residue was dissolved in CH_2Cl_2 (30 mL) and passed through a short plug of silica gel to afford compound **28** (3.32 g, 75%) as a colorless oil. $[\alpha]^{23}_{D}$ –0.35 (*c* 1.5, $CHCl_3$) [lit[6] $[\alpha]^{23}_{D}$ –0.02 (*c* 10.5, $CHCl_3$)]; IR (thin film): *ν* 3000, 2923, 1714, 1464, 1286, 723 cm^{-1}; ^1H NMR (600 MHz, $CDCl_3$): *δ* 2.33 (t, *J* = 7.6 Hz, 2H), 1.64–1.59 (m, 2H), 1.32–1.22 (m, 25H), 1.06–1.03 (m, 2H), 0.86 (t, *J* = 6.9 Hz, 3H), 0.81 (d, *J* = 6.6 Hz, 3H); ^{13}C NMR (150 MHz, $CDCl_3$): *δ* 180.3 (C=O), 37.1 (CH_2 × 2), 34.1 (CH_2), 32.7 (CH), 31.9 (CH_2), 30.0 (CH_2), 29.9 (CH_2), 29.7 (CH_2), 29.5 (CH_2), 29.4 (CH_2), 29.3 (CH_2), 29.1 (CH_2), 27.1 (CH_2), 27.0 (CH_2), 24.7

(CH_2), 22.7 (CH_2), 19.7 (CH_3), 14.1 (CH_3); HRMS (EI): *m*/*z* calcd for $C_{19}H_{38}O_2$ ($[M]^+$): 298.2875, found: 298.2872.

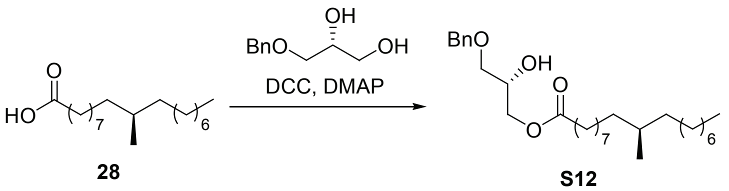

28 S12

3-*O*-Benzyl-1-*O*-[(*R*)-10-methyloctadecanoyl]-*sn*-glycerol (S12). DMAP (11 mg, 0.09 mmol) and dicyclohexylcarbodiimide (DCC, 377 mg, 1.83 mmol) were added to a solution of compound **28** (271 mg, 0.92 mmol) and 3-*O*-benzyl-*sn*-glycerol (200 mg, 1.10 mmol) in CH_2Cl_2 (10 mL) at 0 °C. The reaction was warmed up to room temperature and stirred for an additional 12 h. The mixture was then filtered through Celite, washed with saturated $NaHCO_{3(aq)}$ and brine, dried over $MgSO_4$ and concentrated under reduced pressure. The residue was purified by flash column chromatography (ethyl acetate/hexanes = 1/4) to give compound **S12** (317 mg, 75%). $[\alpha]^{23}_{D}$ +9.5 (*c* 3.3, $CHCl_3$); IR (thin film): *ν* 3456, 2918, 2853, 1737, 1494, 1453, 1378, 1247, 1174, 1094, 1028,731, 697, cm^{-1}; ^{1}H NMR (600 MHz, $CDCl_3$): δ 7.35–7.27 (m, 5H, Ar-H), 4.54 (s, 2H, PhCH_2), 4.17 (dd, J = 11.6, 4.2 Hz, 1H), 4.12 (dd, J = 11.6, 6.6 Hz, 1H), 4.03–4.00 (m, 1H), 3.53 (dd, J = 9.6, 4.4 Hz, 1H), 3.47 (dd, J = 9.6, 6.2 Hz, 1H), 2.57 (s, 1H, OH), 2.30 (t, J = 7.2 Hz, 2H), 1.61–1.56 (m, 2H), 1.33–1.24 (m, 25H), 1.06–1.03 (m, 2H), 0.86 (t, J = 7.2 Hz, 3H), 0.82 (d, J = 6.6 Hz, 3H); ^{13}C NMR (150 MHz, $CDCl_3$): δ 173.9 (C=O), 137.6 (C), 128.5 (CH × 2), 127.9 (CH), 127.7 (CH × 2), 73.5 (CH_2), 70.8 (CH_2), 68.9 (CH), 65.3 (CH_2), 37.1 (CH_2), 34.1 (CH_2), 32.7 (CH), 31.9 (CH_2), 30.0 (CH_2), 29.9 (CH_2), 29.7 (CH_2), 29.5 (CH_2), 29.34 (CH_2), 29.25 (CH_2), 29.1 (CH_2), 27.1 (CH_2), 27.0 (CH_2), 24.9 (CH_2), 22.7 (CH_2), 19.7 (CH_3), 14.1 (CH_3) HRMS (EI): *m*/*z* calcd for $C_{29}H_{50}O_4Na$ ($[M + Na]^+$): 485.3607, found: 485.3600.

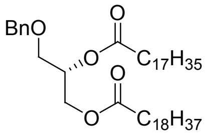

29

3-*O*-Benzyl-1-*O*-[(*R*)-10-methyloctadecanoyl]-2-*O*-stearoyl-*sn*-glycerol (29). To a solution of alcohol **S12** (255 mg, 0.55 mmol) and stearic acid (235 mg, 0.83 mmol) in CH_2Cl_2 (10 mL) at 0 °C, DMAP (14 mg, 0.12 mmol) and DCC (228 mg, 1.11 mmol) were added. The mixture was warmed up to room temperature and stirred for an additional 12 h. After

filtration through Celite, the organic solution was washed with saturated $NaHCO_{3(aq)}$ and brine, dried over $MgSO_4$, and concentrated under reduced pressure. The residue was purified by flash column chromatography (ethyl acetate/hexanes = 1/10) to give compound **29** (354 g, 88%). $[\alpha]^{23}_{D}$ +10.9 (*c* 4.5, $CHCl_3$); IR (thin film): *v* 2853, 2926, 1742, 1376, 1460, 1163, 1114, 1023, 735, 697 cm^{-1}; ^1H NMR (600 MHz, $CDCl_3$): δ 7.34–7.26 (m, 5H, Ar-H), 5.24–5.20 (m, 1H), 4.53, 4.50 (AB_q, J = 12.0 Hz, 2H, $PhCH_2$), 4.32 (dd, J = 11.8, 3.8 Hz, 1H), 4.17 (dd, J = 11.8, 6.5 Hz, 1H), 3.59–3.55 (m, 2H), 2.30 (t, J = 7.5 Hz, 2H), 2.26 (t, J = 7.5 Hz, 2H), 1.62–1.54 (m, 4H), 1.37–1.03 (m, 55H,) 0.86 (t, J = 7.0 Hz, 6H), 0.81 (d, J = 6.7 Hz, 3H); ^{13}C NMR (150 MHz, $CDCl_3$): δ 173.4 (C=O), 173.1 (C=O), 137.7 (C), 128.4 (2 × CH), 127.8 (CH), 127.6 (2 × CH), 73.3 (CH_2), 70.0 (CH), 68.2 (CH_2), 62.6 (CH_2), 37.1 (CH_2), 34.3 (CH_2), 34.1 (CH_2), 32.8 (CH), 31.9 (CH_2), 30.03 (CH_2), 29.98 (CH_2), 29.8 (CH_2), 29.69 (CH_2), 29.66 (CH_2), 29.6 (CH_2), 29.53 (CH_2), 29.48 (CH_2), 29.4 (CH_2), 29.3 (CH_2), 29.1 (CH_2), 29.1 (CH_2), 27.1 (CH_2), 25.0 (CH_2), 24.9 (CH_2), 22.7 (CH_3), 14.1 (CH_3 × 2); HRMS (EI): *m*/*z* calcd for $C_{47}H_{84}O_5Na$ ($[M + Na]^+$): 751.6216, found 751.6208.

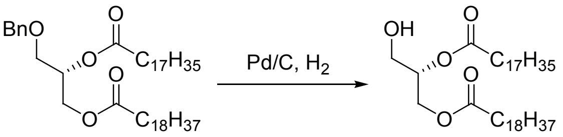

29 **S13**

1-*O*-[(*R*)-10-methyloctadecanoyl]-2-*O*-stearoyl-*sn*-glycerol (S13). A solution of compound **29** (200 mg, 0.27 mmol) in ethanol (5 mL) were mixed with acetic acid (0.5 mL) and Pd/C (15 mg, 10% Pd content) at room temperature and stirred for 4 h before being filtered through a pad of Celite. The solvent was removed *in vacuo* at 25 °C. The residue was purified immediately by flash column chromatography (ethyl acetate/hexanes = 1/4) to afford the alcohol **S13** (162 mg, 92%). $[\alpha]^{23}_{D}$ –1.5 (*c* 1.5, $CHCl_3$); IR (thin film): *v* 3469, 2922, 2840, 1739, 1462, 1376, 1168, 1116, 718 cm^{-1}; ^1H NMR (600 MHz, $CDCl_3$): δ 5.08–5.05 (m, 1H), 4.30 (dd, J = 12.0, 4.2 Hz, 1H), 4.22 (dd, J = 12.0, 5.4 Hz, 1H), 3.74–3.3.68 (m, 2H), 2.32 (t, J = 7.2 Hz, 2H), 2.30 (t, J = 7.2 Hz, 2H), 2.01 (s, 1H, OH), 1.63–1.56 (m, 4H), 1.32–1.13 (m, 55H), 0.86 (t, J = 7.2 Hz, 6H), 0.81 (d, J = 6.6 Hz, 3H); ^{13}C NMR (150 MHz, $CDCl_3$): δ 173.8 (C=O), 173.4 (C=O), 72.1 (CH), 62.0 (CH_2), 61. 6 (CH_2), 37.1 (CH_2), 34.3 (CH_2), 34.1 (CH_2), 32.8 (CH), 31.9 (CH_2), 30.03 (CH_2), 29.96 (CH_2), 29.69 (CH_2), 29.65 (CH_2), 29.51 (CH_2), 29.47 (CH_2), 29.4 (CH_2), 29.3 (CH_2), 29.13 (CH_2), 29.08 (CH_2), 27.1 (CH_2), 24.94 (CH_2), 24.89 (CH_2), 24.7 (CH_2), 19.7 (CH_3), 14.1 (CH_3 × 2); HRMS (ESI): *m*/*z* calcd for $C_{40}H_{78}O_5Na$ ($[M + Na]^+$): 661.5747, found: 661.5745.

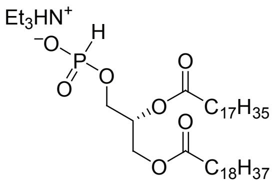

4

Triethylammonium 1-*O*-[(*R*)-10-methyloctadecanoyl]-2-*O*-stearoyl-*sn*-glycero-3-*H*-phosphonate (4). Imidazole (150 mg, 0.24 mmol) was dissolved in toluene, which was later vaporized to enable moisture coevaporation. After further keeping under vacuum overnight, the dry imidazole was dissolved in toluene (5 mL) and cooled to 0 °C. A solution of PCl_3 (62 μL, 0.71 mmol) in toluene (1 mL) and Et_3N (262 μL, 1.878 mmol) were then added to the imidazole solution. After 1 h, the reaction temperature was lowered to –10 °C and a solution of the alcohol **S13** (150 mg, 0.24 mmol) in a mixture of toluene (2 mL) and CH_2Cl_2 (1 mL) was added via syringe pump for over a period of 1 h. The reaction was stirred for an additional 1 h before quenching with pyridine/water (1/4, 5 mL). The crude phosphonate was extracted with $CHCl_3$, and the combined organic layer was washed with triethylammonium bicarbonate buffer and dried over Na_2SO_4. Concentration *in vacuo* gave the crude residue, which was purified by flash column chromatography with Et_3N-containing silica gel ($MeOH/CHCl_3$ = 1/5) to afford the *H*-phosphonate **4** (132 mg, 69%). $[\alpha]^{23}_{D}$ –1.5 (*c* 1.5, $CHCl_3$); ^1H NMR (600 MHz, $CDCl_3$): δ 5.09–5.19 (m, 1H), 4.24 (d, J = 11.0 Hz, 1H), 4.05 (dd, J = 11.0, 5.8 Hz, 1H), 4.00–3.94 (m, 2H), 3.01–2.95 (m, 4H), 2.20–2.16 (m, 4H), 1.47–0.70 (m, 80H); ^{13}C NMR (150 MHz, $CDCl_3$): δ 173.3 (C=O), 172.9 (C=O), 69.9 (d, $^{31P\text{-}13C}J$ = 6.9 Hz, CH), 62.3 (d, $^{31P\text{-}13C}J$ = 3.8 Hz, CH_2), 62.2 (CH_2), 45.7 (CH_2), 37.0 (CH_2), 34.2 (CH_2), 34.0 (CH_2), 32.7 (CH), 31.9 (CH_2), 30.0 (CH_2), 29.9 (CH_2), 29.7 (CH_2), 29.6 (CH_2), 29.49 (CH_2), 29.45 (CH_2), 29.3 (CH_2), 29.3 (CH_2), 29.09 (CH_2), 29.05 (CH_2), 27.0 (CH_2), 24.8 (CH_2), 22.6 (CH_2), 19.6 (CH_3), 14.1 (CH_3), 8.5 (2 × CH_3); ^{31}P NMR (121 MHz, $CDCl_3$): δ 5.08; HRMS (EI): *m*/*z* calcd for $C_{46}H_{95}O_7NP$ ($[M + H]^+$): 804.6846, found: 804.6849.

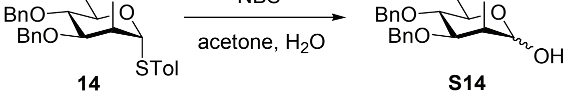

2-*O*-Benzoyl-3,4,6-tri-*O*-benzyl-D-mannopyranose (S14). To the solution of the thioglycoside **14** (10 g, 15.13 mmol) in acetone (1.0 L) at 0 °C, water (5 ml, 0.30 mol) and NBS (4.04 g, 22.7 mmol) were added and the resulting mixture was stirred at the same

temperature for 30 min. The reaction was quenched with 10% $Na_2S_2O_{3(aq)}$ and the solvent was evaporated under reduced pressure. The residue obtained was dissolved in ethyl acetate and washed successively with 10% $Na_2SO_{3(aq)}$ and brine. The resulting solution was dried over $MgSO_4$ and concentrated under reduced pressure. The crude product was purified by using a short silica gel column (ethyl acetate/hexanes = 1/4) to furnish the hemiacetal **S14** (8.21 g, 98%; α/β = 8/1). ^{1}H NMR (600 MHz, $CDCl_3$): δ 8.10 (d, J = 7.9 Hz, 0.26H, Ar-H), 8.06 (d, J = 7.8 Hz, 2H, Ar-H), 7.55 (t, J = 7.5 Hz, 1.13 H, Ar-H), 7.40–7.17 (m, 19H, Ar-H), 5.70 (d, J = 2.8 Hz, 0.13H), 5.61 (d, J = 1.8 Hz, 1H), 5.32 (s, 1H), 4.86 (d, J = 10.8 Hz, 1H, PhCH_2), 4.83 (s, 0.13H), 4.81 (d, J = 11.4 Hz, 0.26H, PhCH_2), 4.77 (d, J = 11.4 Hz, 1H, PhCH_2), 4.69 (d, J = 12.0 Hz, 0.13H, PhCH_2), 4.64 (d, J = 12.0 Hz, 1H, PhCH_2), 4.57–4.50 (m, 3.39H, PhCH_2), 4.16–4.12 (m, 1H), 3.92 (t, J = 9.6 Hz, 1.13H), 3.81 (d, J = 3.1 Hz, 0.26H), 3.75 (d, J = 3.7 Hz, 2H), 3.58–3.55 (m, 0.13H); ^{13}C NMR (150 MHz, $CDCl_3$): δ 166.2 (C=O), 165.7 (C=O), 138.2 (C), 138.02 (C), 137.96 (C), 137.9 (C), 137.8 (C), 137.5 (C), 133.2 (CH), 133.1 (CH), 130.1 (CH), 129.9 (CH), 129.8 (C), 129.6 (C), 128.4 (CH), 128.3 (CH), 128.2 (CH), 128.1 (CH), 128.0 (CH), 127.8 (CH), 127.7 (CH), 127.63 (CH), 127.57 (CH), 93.3 (CH), 92.5 (CH), 80.28 (CH), 77.7 (CH), 75.1 (CH, CH_2), 74.5 (CH), 73.8 (CH), 73.5 (CH_2), 73.4 (CH_2), 71.5 (CH_2), 71.4 (CH_2), 71.2 (CH), 69.9 (CH), 69.4 (CH_2), 69.3 (CH), 69.0 (CH_2); HRMS (ESI): m/z calcd for $C_{34}H_{34}O_7Na$ ($[M + Na]^+$): 577.2202, found: 577.2204.

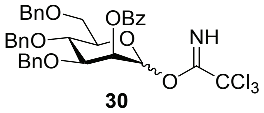

30

2-*O*-Benzoyl-3,4,6-tri-*O*-benzyl-*α*-D-mannopyranosyl trichloroacetimidate (30). The solution of compound **S14** (3 g, 5.438 mmol) in CH_2Cl_2 (30 mL) was cooled in an ice-water bath and CCl_3CN (5.43 mL, 54.1 mmol) and diazabicycloundec-7-ene (162 μL, 1.08 mmol) were sequentially added under nitrogen atmosphere. The reaction was gradually warmed up to room temperature and stirred for 5 h. Then, the mixture was diluted with CH_2Cl_2 (20 mL) and washed with water and brine. After drying over $MgSO_4$, the solvent was removed under reduced pressure. The residue was passed through a short silica gel column (ethyl acetate/hexanes = 1/4) to obtain compound **30** (3.71 g, 98%). ^{1}H NMR (600 MHz, $CDCl_3$): δ 8.69 (s, 1H, NH), 8.62 (s, 0.16H, NH), 8.07 (d, J = 7.2 Hz, 2.6H, Ar-H), 7.57–7.54 (m, 1.3H, Ar-H), 7.37–7.20 (m, 21.8H), 6.40 (d, J = 1.2 Hz, 1H), 6.09 (d, J = 3 Hz, 0.16H), 5.97 (s, 0.16H), 5.74–5.73 (m, 1H), 4.88–4.86 (m, 1.16H), 4.85–4.79 (m, 1.16H),

4.62–4.52 (m, 3.64H), 4.25 (t, J = 9.0 Hz, 1H), 4.15 (dd, J = 3.0, 9.0 Hz, 1H), 4.10–4.02 (m, 1.32H), 3.93–3.88 (m, 1.64H), 3.78–3.71 (m, 1.32H).

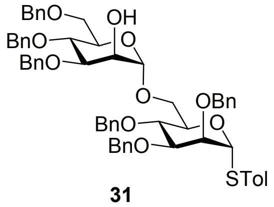

31

4-Methylphenyl-(3,4,6-tri-*O*-benzyl-*α*-D-mannopyranosyl)-(1→6)-2,3,4-tri-*O*-benzyl-1-thio-D-mannopyranoside (31). A mixture of 6-alcohol **8** (0.98 g, 1.76 mmol), trichloroacetimidate **30** (1.85 g, 2.64 mmol) and 3 Å molecular sieves (3 g) was stirred in CH_2Cl_2 (60 mL) for 1 h, under nitrogen atmosphere before cooling the reaction flask to –78 °C. TMSOTf (64 μL, 0.35 mmol) was added and the mixture was continuously stirred at –78 °C for 3 h. Afterwards, the reaction mixture was diluted with methanol (60 ml) and then warmed up to room temperature. NaOMe (475 mg, 8.80 mmol) was then added at room temperature and the resulting mixture was stirred for 18 h. The mixture was filtered through Celite and the filtrate was neutralized by Dowex-IR resin, filtered and concentrated under reduced pressure. The crude residue was purified by flash column chromatography (ethyl acetate/hexanes = 1/2.5) to furnish the alcohol **31** (1.52 g, 87%.). $[\alpha]^{27}_{D}$ +57.2 (c 1.5, $CHCl_3$); IR (thin film): ν 3466, 3030, 2918, 1496, 1366, 1209, 1104, 738, 695 cm^{-1}; ^1H NMR (600 MHz, $CDCl_3$): δ 7.41–7.25 (m, 30H, Ar-H), 7.20 (d, J = 7.2 Hz, 2H, Ar-H), 7.11 (d, J = 7.2 Hz, 1H, Ar-H), 5.54 (s, 1H, 1-H), 5.06 (s, 1H, 1′-H), 4.97 (d J = 10.8 Hz, 1H, PhCH_2), 4.87 (d, J = 10. Hz, 1H, PhCH_2), 4.73–4.46 (m, 10H, PhCH_2), 4.92 (dd, J = 9.8, 4.1 Hz, 1H, 5-H), 4.11 (d, J = 1.3 Hz, 1H, 2′-H), 4.05 (d, J =1.7 Hz, 1H, 2-H), 4.02–4.98 (m, 2H, 4-H, 6-H_a), 3.92–3.90 (m, 3H, 3-H, 3′-H, 4′-H), 3.85–3.83 (m, 1H, 5′-H), 3.78–3.61 (m, 3H, 6-H_b, 6′-H × 2), 2.23 (s, 3H, CH_3); ^{13}C NMR (150 MHz, $CDCl_3$): δ 138.5 (C), 138.3 (C), 138.2 (C), 138.1 (C), 137.9 (C), 137.8 (C), 137.49 (C), 134.45 (C), 132.9 (CH), 131.5 (CH), 130.8 (C), 129.9 (CH), 129.6 (CH), 128.5 (CH), 128.42 (CH), 128.37 (CH), 128.34 (CH), 128.27 (CH), 128.2 (CH), 127.89 (CH), 127.85 (CH), 127.80 (CH), 127.75 (CH), 127.5 (CH), 99.5 (CH), 85.9 (CH), 80.2 (CH), 79.7 (CH), 76.3 (CH), 75.1 (CH_2), 75.1 (CH_2), 74.7 (CH), 74.2 (CH), 73.3 (CH_2), 72.3 (CH), 72.0 (CH_2), 71.9 (CH_2), 71.7 (CH_2), 71.0 (CH), 68.7 (CH_2), 68.0 (CH), 66.4 (CH_2), 21.0 (CH_3); HRMS (ESI): m/z calcd for $C_{61}H_{64}O_{10}NaS$ ($[M + Na]^+$): 1011.4118, found: 1011.4109.

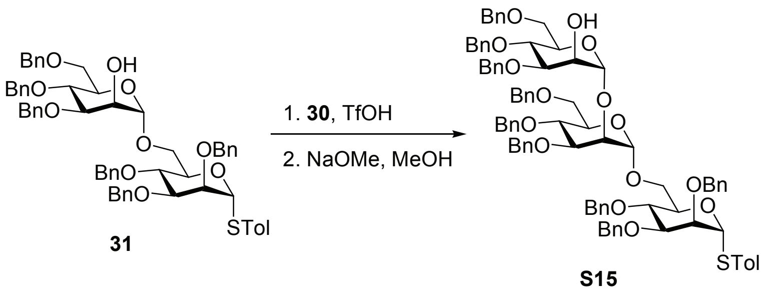

4-Methylphenyl (3,4,6-tri-*O*-benzyl-*α*-D-mannopyranosyl)-(1→2)-(3,4,6-tri-*O*-benzyl-*α*-D-mannopyranosyl)-(1→6)-2,3,4-tri-*O*-benzyl-1-thio-*α*-D-mannopyranoside (S15). A mixture of the trichloroacetimidate **30** (1 g, 1.43 mmol), the 2′-alcohol **31** (1.42 g, 1.43 mmol) and 3 Å molecular sieves (4 g) in CH_2Cl_2 (70 mL) was stirred at room temperature for 1 h under nitrogen. The reaction mixture was cooled down to –60 °C, TfOH (26μL, 0.29 mmol) was added, and the solution was stirred continuously while gradually warming up to –40 °C over a period of 1 h. Further, trichloroacetimidate **30** (0.5 g, 0.72 mmol) in CH_2Cl_2 (5 mL) and TfOH (26 μL, 0.29 mmol) were added to the reaction and the resulting mixture was stirred at the same temperature for another 3 h. After the completion of glycosylation, methanol (80 mL) was added and the reaction was gradually warmed up to room temperature. NaOMe (775 mg, 14.35 mmol) was added and the solution was stirred at room temperature for 18 h. The reaction was filtered through Celite and neutralized with Dowex 50 WX2-200 IR-resin. The mixture was filtered through a sintered glass and the filtrate was concentrated under reduced pressure. Purification of the crude compound by flash column chromatography (ethyl acetate/hexanes = 1/3) furnished the 2-alcohol **S15** (1.43 g, 70%). $[\alpha]^{25}_{D}$ +36.3 (*c* 6.0, $CHCl_3$); IR (thin film): *ν* 3448, 3026, 2918, 1600, 1497, 1456, 1364, 1208, 1104, 747, 697 cm^{-1}; ^{1}H NMR (600 MHz, $CDCl_3$): *δ* 7.34–7.11 (m, 49H, Ar-H), 7.03 (d, *J* = 7.8 Hz, 2H, Ar-H), 5.49 (d, *J* = 0.8 Hz, 1H, 1-H), 5.10 (d, *J* = 0.8 Hz, 1H, 1″-H), 4.98 (d, *J* = 1.2 Hz, 1H, 1′-H), 4.88 (d, *J* = 11.2 Hz, 1H, PhCH_2), 4.82 (d, *J* =10.9 Hz, 1H, PhCH_2), 4.78 (d, *J* = 10.9 Hz, PhCH_2), 4.69 (d, *J* = 12.3 Hz, 1H, PhCH_2), 4.64 (d, *J* = 12.2 Hz, 1H, PhCH_2), 4.56–4.51 (m, 12H, PhCH_2), 4.36 (d, *J* = 12.2 Hz, 1H, PhCH_2), 4.22–4.19 (m, 1H, 5-H), 4.10 (t, *J* = 2.0 Hz, 1H, 2″-H), 4.08 (t, *J* = 2.3 Hz, 1H, 2′-H), 3.98 (t, *J* = 2.1 Hz, 1H, 2-H), 3.94–3.82 (m, 8H, 3-H, 4-H, 6-H_a, 3′-H, 4′-H, 3″-H, 4″-H, 5″-H), 3.76–3.68 (m, 3H, 5′-H, 6′-H_a, 6″-H_a), 3.60–3.57 (m, 3H, 6-H_b, 6′-H_b, 6″-H_b), 2.12 (s, 3H, CH_3); ^{13}C NMR (150 MHz, $CDCl_3$): *δ* 138.7 (C), 138.5 (C), 138.5 (C), 138.3 (C), 138.2 (C), 138.1 (C), 138.04 (C), 138.02 (C), 137.8 (C), 137.4 (C), 131.4 (CH), 131.0 (C), 129.9 (CH), 128.5 (CH), 128.4 (CH), 128.3 (CH), 128.2 (CH), 128.20 (CH), 128.18 (CH), 127.9 (CH), 127.82 (CH), 127.81 (CH), 127.76 (CH), 127.71 (CH), 127.69 (CH), 127.60 (CH), 127.58 (CH), 127.5

(CH), 127.4 (CH), 127.3 (CH), 101.0 (CH, *J* = 171.7 Hz), 99.1 (CH, *J* = 171.1 Hz), 86.0 (CH, *J* = 166.3 Hz), 80.3 (CH), 79.9 (CH), 79.2 (CH), 76.2 (CH), 75.02 (CH_2), 74.97 (CH_2), 74.9 (CH_2), 74.7 (CH), 74.62 (CH), 74.57 (CH), 74.2 (CH), 73.3 (CH_2), 73.1 (CH_2), 72.2 (CH), 72.0 (CH_2), 71.9 (CH_2), 71.8 (CH_2, CH), 71.7 (CH_2), 71.4 (CH), 69.1 (CH_2), 68.8 (CH_2), 68.5 (CH), 66.7 (CH_2), 20.9 (CH_3); HRMS (ESI): *m*/*z* calcd for $C_{88}H_{92}O_{15}NaS$ ($[M + Na]^+$): 1443.6055, found: 1443.6044.

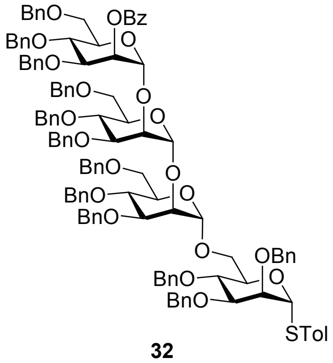

32

4-Methylphenyl (2-*O*-benzoyl-3,4,6-tri-*O*-benzyl-*α*-D-mannopyranosyl)-(1→2)-(3,4,6-tri-*O*-benzyl-*α*-D-mannopyranosyl)-(1→2)-(3,4,6-tri-*O*-benzyl-*α*-D-mannopyranosyl)-(1→6)-2,3,4-tri-*O*-benzyl-1-thio-*α*-D-mannopyranoside (32). A mixture of the trichloroacetimidate **30** (541 mg, 0.78 mmol), the 2-alcohol **S15** (1.10 g, 0.77 mmol) and 3 Å molecular sieves (2 g) in CH_2Cl_2 was stirred at room temperature for 1 h under nitrogen. The reaction flask was cooled to –60 °C, TfOH (14 μL, 0.15 mmol) was added and the solution was stirred continuously while gradually warming up to –40 °C for over a period of 1 h. Further, trichloroacetimidate **30** (270 mg, 0.39 mmol) and TfOH (14 μL, 0.15 mmol) were added to the reaction and stirred at the same temperature for another 3 h. After quenching with Et_3N, the reaction was filtered through Celite, and the combined filtrate was successively washed with saturated $NaHCO_{3(aq)}$, water and brine. The organic solution was dried over $MgSO_4$ and concentrated under reduced pressure. The crude compound was purified by flash column chromatography (ethyl acetate/hexanes = 1/6.5) to get the tetrasaccharide **32** (1.12 g, 74%). $[\alpha]^{25}_{D}$ +12.7 (*c* 1.1, $CHCl_3$); IR (thin film): *ν* 3030, 2918, 2862, 1727, 1602, 1495, 1361, 1267, 1104, 1026, 736, 695 cm^{-1}; ^1H NMR (600 MHz, $CDCl_3$): *δ* 8.06 (d, *J* = 7.5 Hz, 2H, Ar-H), 7.54 (t, *J* = 7.5 Hz, 1H, Ar-H), 7.36–7.07 (m, 63H, Ar-H), 7.02 (d, *J* = 8.0 Hz, 2H, Ar-H), 6.96 (t, *J* = 7.4 Hz, 1H, Ar-H), 5.74 (bs, 1H, 2‴-H), 5.47 (s, 1H, 1-H), 5.21 (d, *J* = 1.1 Hz, 1″-H), 5.11 (d, *J* = 1.3 Hz, 1H, 1‴-H), 4.95 (d, *J* = 1.1 Hz, 1H, 1′-H), 4.88 (d, *J* = 11.1 Hz, 1H, PhC*H*$_2$), 4.83 (d, *J* = 10.9 Hz, 1H, PhC*H*$_2$), 4.82 (d, *J*

= 10.9 Hz, 2H, PhCH_2), 4.74 (d, J = 11.1 Hz, 1H, PhCH_2), 4.68 (d, J = 12.4 Hz, 1H, PhCH_2), 4.63 (d, J = 12.3 Hz, 1H, PhCH_2), 4.59 (d, J = 12.0 Hz, 2H, PhCH_2), 4.58–4.43 (m, 10H, PhCH_2), 4.40 (d, J = 12.2 Hz, 1H, PhCH_2), 4.39 (d, J = 11.1 Hz, 1H, PhCH_2), 4.36 (d, J = 11.6 Hz, 1H, PhCH_2), 4.29 (d, J = 12.0 Hz, 1H, PhCH_2), 4.23–4.21 (m, 1H, 5-H), 4.13–4.09 (m, 3H), 4.06 (t, J = 2.0 Hz, 1H, 2′-H), 3.97 (t, J = 2.0 Hz, 1H, 2-H), 3.94–3.83 (m, 7H), 3.83–3.66 (m, 6H), 3.63–3.54 (m, 4H), 2.09 (s, 3H, CH_3); ^{13}C NMR (150 MHz, $CDCl_3$): δ 165.4 (C=O), 138.7 (C), 138.63 (C), 138.57 (C), 138.51 (C), 138.46 (C × 3), 138.37 (C), 138.35 (C), 138.11 (C), 138.09 (C), 138.05 (C), 137.8 (C), 137.4 (C), 133.0 (CH), 131.4 (CH), 131.1 (C), 130.1 (CH), 130.0 (CH), 129.9 (CH), 128.4 (CH), 128.31 (CH), 128.27 (CH), 128.25 (CH), 128.18 (CH), 128.16 (CH), 128.1 (CH), 127.92 (CH), 127.90 (CH), 127.85 (CH), 127.82 (CH), 127.80 (CH), 127.73 (CH), 127.69 (CH), 127.6 (CH), 127.53 (CH), 127.50 (CH), 127.43 (CH), 127.38 (CH), 127.3 (CH), 100.5 (CH, J = 171 Hz, C-1″), 99.4 (CH, J = 171 Hz, C-1‴), 99.1 (CH, J = 172 Hz, C-1′), 86.0 (CH, J = 166 Hz, C-1), 80.3 (CH), 79.3 (CH × 2), 78.2 (CH), 76.0 (CH), 75.4 (CH), 75.2 (CH_2), 75.1 (CH_2), 74.99 (CH_2), 74.96 (CH_2), 74.7 (CH × 2), 74.62 (CH), 74.60 (CH), 74.2 (CH), 73.3 (CH_2), 73.1 (CH_2 × 3), 72.22 (CH_2), 72.16 (2 × CH), 72.1 (CH), 71.9 (CH_2), 71.8 (CH, CH_2), 71.6 (CH_2), 69.12 (CH_2), 69.06 (CH_2), 69.01 (CH), 68.89 (CH_2), 66.8 (CH_2), 20.9 (CH_3); HRMS (ESI): m/z calcd for $C_{122}H_{124}O_{21}NaS$ ($[M + Na]^+$): 1979.8254, found: 1979.8293.

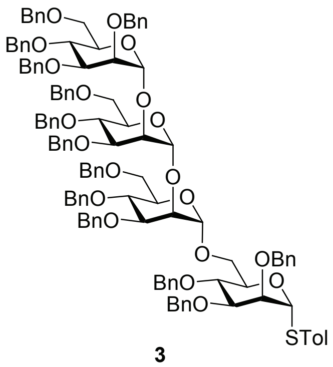

3

4-Methylphenyl (2,3,4,6-tetra-*O*-benzyl-*α*-D-mannopyranosyl)-(1→2)-(3,4,6-tri-*O*-benzyl-*α*-D-mannopyranosyl)-(1→2)-(3,4,6-tri-*O*-benzyl-*α*-D-mannopyranosyl)-(1→6)-2,3,4-tri-*O*-benzyl-1-thio-*α*-D-mannopyranoside (3). The solution of the benzoate **32** (2.40 g, 1.23 mmol) in DMF (20 mL) was placed in an ice bath and NaH (60% oil dispersion, 74 mg, 3.08 mmol) was added. After 10 min of stirring, a second portion of NaH (60% oil dispersion, 74 mg, 3.08 mmol) and benzyl bromide (292 μL, 2.45 mmol) were added, and the reaction was stirred continuously with gradually warming up to room temperature for 2 h.

The mixture was, then, cooled to 0 °C, diluted with ethyl acetate and quenched with water. After extraction with ethyl acetate, the combined organic layer was washed with water and brine, dried over $MgSO_4$, filtered, and concentrated under reduced pressure. The crude residue was purified by flash column chromatography (ethyl acetate/hexanes = 1/5) to afford the tetrasaccharide **3** (2.34 g, 98%). $[\alpha]^{27}_{D}$ +19.7 (*c* 0.5, $CHCl_3$); ^1H NMR (600 MHz, $CDCl_3$): δ 7.35–7.13 (m, 67H, Ar-H), 7.05 (d, *J* = 8.0 Hz, 2H, Ar-H), 5.50 (d, *J* = 1.0 Hz 1H, 1-H), 5.21 (s, 2H, 1″-H, 1‴-H), 5.01 (d, *J* = 1.3 Hz, 1H, 1′-H), 4.91 (d, *J* =11.1 Hz, 1H, $PhCH_2$), 4.87 (d, *J* = 10.9 Hz, 2H, $PhCH_2$), 4.84 (d, *J* = 10.9 Hz, 1H, $PhCH_2$), 4.70 (d, *J* = 12.4 Hz, 1H, $PhCH_2$), 4.67 (d, *J* = 12.2 Hz, 1H, $PhCH_2$), 4.64 (d, *J* = 12.2 Hz, 1H, $PhCH_2$), 4.61–4.42 (m, 20H, $PhCH_2$), 4.33 (d, *J* = 12.1 Hz, 1H, $PhCH_2$), 4.31 (d, *J* = 12.4 Hz, 1H, $PhCH_2$), 4.27–4.25 (m, 1H, 5-H), 4.18 (t, *J* = 2.3 Hz, 1H), 4.09–4.06 (m, 2H), 4.01 (t, *J* = 2.3 Hz, 1H, 2-H), 3.98–3.79 (m, 12H), 3.77 (dd, *J* = 4.4, 11.3 Hz, 1H), 3.73 (dd, *J* = 4.5, 11.1 Hz, 1H), 3.69–3.61 (m, 4H), 3.56 (dd, *J* = 3.6, 10.6 Hz, 1H), 2.12 (s, 3H, CH_3); ^{13}C NMR (150 MHz, $CDCl_3$): δ 138.8 (C), 138.73 (C), 138.66 (C), 138.6 (C), 138.5 (C × 2), 138.42 (C), 138.35 (C × 2), 138.3 (C), 138.14 (C), 138.07 (C), 137.8 (CH), 131.4 (C), 129.9 (CH), 128.43 (CH), 128.38 (CH), 128.36 (CH), 128.31 (CH), 128.28 (CH), 128.23 (CH), 128.18 (CH), 128.15 (CH), 128.1 (CH), 128.0 (CH), 127.92 (CH), 127.87 (CH), 127.8 (CH), 127.74 (CH), 127.71 (CH), 127.69 (CH), 127.66 (CH), 127.63 (CH), 127.55 (CH), 127.5 (CH), 127.43 (CH), 127.40 (CH), 127.3 (CH), 100.7 (CH, *J* = 172.6 Hz), 99.3 (CH, *J* = 169.5 Hz), 99.09 (CH, *J* = 171.5 Hz, C-1′), 86.0 (CH, *J* = 166.2 Hz, C-1), 80.3 (CH), 79.8 (CH), 79.7 (CH), 79.2 (CH), 76.1 (CH), 75.1 (CH_2), 75.0 (CH_2), 74.9 (CH_2), 74.8 (CH × 2), 74.7 (CH × 2), 74.6 (CH), 74.5 (CH), 73.3 (CH_2), 73.2 (CH_2), 73.1 (CH_2), 72.5 (CH_2), 72.23 (CH × 2), 72.16 (CH), 72.1 (CH_2), 72.0 (CH_2), 71.9 (CH_2), 71.8 (CH_2), 71.7 (CH), 71.5 (CH_2), 69.2 (CH_2), 69.1 (CH_2), 69.0 (CH_2), 20.9 (CH_3); HRMS (ESI): *m/z* calcd for $C_{122}H_{126}O_{20}NaS$: ($[M + Na]^+$): 1965.8461, found: 1965.8475.

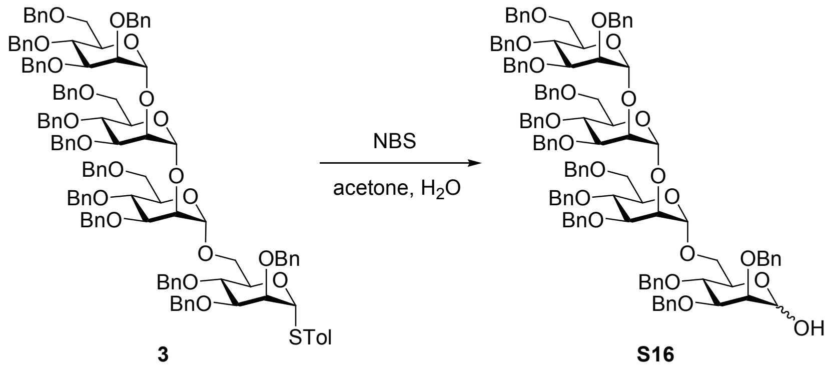

(2,3,4,6-Tetra-*O*-benzyl-*α*-D-mannopyranosyl)-(1→2)-(3,4,6-tri-*O*-benzyl-*α*-D-mannopyranosyl)-(1→2)-(3,4,6-tri-*O*-benzyl-*α*-D-mannopyranosyl)-(1→6)-2,3,4-tri-*O*-benzyl-D-mannopyranose (S16). A solution of the thioglycoside **3** (2.44 g, 1.26 mmol) in acetone (30 mL) was cooled down to 0 °C. NBS (335 mg, 1.89 mmol) was then added and the mixture was stirred continuously at the same temperature for 3 h. After concentration under reduced pressure, the residue was dissolved in ethyl acetate and washed successively with 10% $Na_2S_2O_{3(aq)}$ and brine. After drying over $MgSO_4$, the organic layer was under reduced pressure and the crude product was purified by flash column chromatography (ethyl acetate/hexanes = 1/2) to furnish the 1-alcohol **S16** (2.13 g, 92%, α/β = 1/7). ^{1}H NMR (600 MHz, $CDCl_3$): δ 7.35–7.16 (m, 520H, Ar-H), 5.21 (d, J = 1.0 Hz, 1H), 5.21–5.20 (m 14H), 5.05–5.02 (m, 9H), 4.94 (s, 7H), 4.90–4.73 (m, 32H), 4.70–4.41 (m, 165H), 4.36–4.33 (m, 8H), 4.23–4.22 (m, 2H), 4.16–4.13 (m, 14H), 4.06–3.78 (m, 97H), 3.76–3.60 (m, 74H), 3.57–3.55 (m, 9H), 3.34–3.32 (m, 1H); ^{13}C NMR (150 MHz, $CDCl_3$): δ 138.7, 138.64, 138.58, 138.52, 138.50, 138.5, 138.4, 138.34, 138.32, 138.29, 138.22, 138.18, 138.15, 138.01, 137.95, 137.9, 128.51, 128.46, 128.4, 128.34, 128.29, 128.25, 128.19, 128.15, 128.1, 128.0, 127.94, 127.87, 127.8, 127.6, 127.7, 127.59, 127.56, 127.44, 127.36, 127.33, 127.29, 101.0, 99.7, 99.3, 99.2, 98.4, 93.9, 92.1, 83.0, 79.9, 79.8, 79.7, 79.6, 78.4, 75.4, 75.2, 75.1, 75.0, 74.9, 74.8, 74.74, 74.66, 74.61, 74.57, 74.4, 74.1, 73.8, 73.5, 73.3, 73.21, 73.16, 73.1, 72.5, 72.4, 72.21, 72.17, 72.0, 71.94, 71.85, 71.8, 71.0, 70.2, 69.6, 69.2, 69.1, 69.0, 67.6, 67.1; HRMS (ESI): m/z calcd for $C_{115}H_{120}O_{21}Na$ ($[M + Na]^+$): 1859.8220, found: 1859.8219.

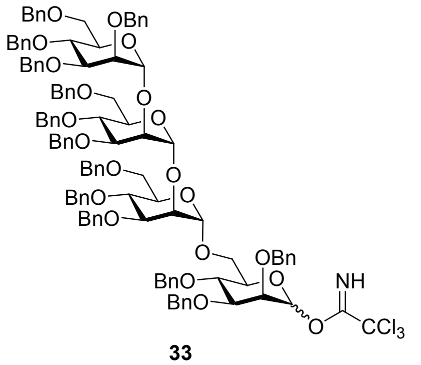

33

(2,3,4,6-Tetra-*O*-benzyl-*α*-D-mannopyranosyl)-(1→2)-(3,4,6-tri-*O*-benzyl-*α*-D-mannopyranosyl)-(1→2)-(3,4,6-tri-*O*-benzyl-*α*-D-mannopyranosyl)-(1→6)-2,3,4-tri-*O*-benzyl-D-mannopyranosyl trichloroacetimidate (33). DBU (12 μL, 0.41 mmol) was added to a solution of the 1-alcohol **S16** (283 mg, 0.15 mmol) and CCl_3CN (154 μL, 1.54 mmol) in CH_2Cl_2 at 0 °C under nitrogen atmosphere. The ice-water bath was then removed and the

mixture was stirred continuously for 12 h. The reaction was washed with water and brine, dried over $MgSO_4$, filtered and concentrated under reduced pressure. The crude product was passed through a short column of Et_3N-neutralized silica gel (ethyl acetate/hexanes = 1/4) to afford the trichloroacetimidate donor **33** (284 mg, 93%). ^{1}H NMR (600 MHz, $CDCl_3$): δ 8.56 (s, 1H, NH), 7.43–7.15 (m, 65H, Ar-H), 6.35 (d, J = 1.9 Hz, 1H, anomeric-H), 5.24 (d, J = 1.1 Hz, 1H, anomeric-H), 5.22 (d, J = 1.3 Hz, 1H, anomeric-H), 5.04 (d, J = 1.5 Hz, 1H, anomeric-H), 4.91 (d, J = 11.0 Hz, 1H, PhCH_2), 4.90 (d, J = 10.9 Hz, 1H, PhCH_2), 4.87 (d, J = 10.6 Hz,1H, PhCH_2), 4.86 (d, J = 22.5 Hz, 1H, PhCH_2), 4.79, 4.74 (ABq, J = 12.4 Hz, 2H, PhCH_2), 4.73 (d, J = 12.2 Hz, 1H, PhCH_2), 4.70 (d, J = 12.2 Hz, 1H, PhCH_2), 4.65–4.46 (m, 16H, PhCH_2), 4.40 (d, J = 11.8 Hz, 1H, Bn-H), 4.37 (d, J = 12.2 Hz, 1H, Bn-H), 4.20 (t, J = 2.0 Hz, 1H), 4.11–4.08 (m, 2H), 4.02–3.60 (m, 21H); ^{13}C NMR (150 MHz, $CDCl_3$): δ 160.0 (C=NH), 138.73 (C), 138.71 (C), 138.62 (C), 138.61 (C), 138.59 (C), 138.55 (C), 138.4 (C), 138.32 (C), 138.26 (C), 138.2 (C), 138.1 (C), 138.0 (C), 137.7 (C), 128.5 (CH), 128.4 (CH), 128.3 (CH), 128.22 (CH), 128.19 (CH), 128.17 (CH), 128.14 (CH), 128.11 (CH), 128.07 (CH), 128.0 (CH), 127.9 (CH), 127.83 (CH), 127.77 (CH), 127.72 (CH), 127.70 (CH), 127.68 (CH), 127.63 (CH), 127.58 (CH), 127.56 (CH), 127.52 (CH), 127.49 (CH), 127.45 (CH), 127.41 (CH), 127.37 (CH), 127.30 (CH), 127.28 (CH), 127.25 (CH), 127.2 (CH), 100.8 (CH), 99.3 (CH), 98.9 (CH), 95.7 (CH), 90.9 (CCl_3), 79.8 (CH), 79.7 (CH), 79.1 (CH), 79.0 (CH), 75.0 (CH_2), 74.9 (3 × CH_2, CH), 74.8 (CH), 74.73 (CH), 74.71 (CH), 74.54 (CH), 74.46 (CH), 74.0 (CH), 73.9 (CH), 73.5 (CH), 73.3 (CH_2), 73.2 (CH_2), 73.1 (CH_2), 72.53 (CH_2), 72.46 (CH_2), 72.22 (CH), 72.15 (CH_2), 72.1 (CH_2), 72.0 (CH_2), 71.7 (CH), 71.3 (CH_2), 69.4 (CH_2), 69.1 (CH_2), 69.0 (CH_2), 66.2 (CH_2).

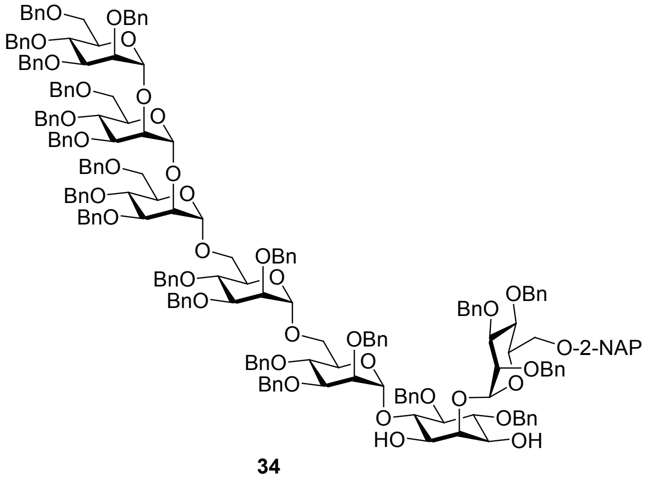

34

4,5-Di-*O*-benzyl-6-*O*-[(2,3,4,6-tetra-*O*-benzyl-*α*-D-mannopyranosyl)-(1→2)-(3,4,6-

tri-*O*-benzyl-*α*-D-mannopyranosyl)-(1→2)-(3,4,6-tri-*O*-benzyl-*α*-D-mannopyranosyl)-(1→6)-(2,3,4-tri-*O*-benzyl-*α*-D-mannopyranosyl)-(1→6)-2,3,4-tri-*O*-benzyl-*α*-D-mannopyranosyl]-2-*O*-[2,3,4-tri-*O*-benzyl-6-*O*-(2-naphthylmethyl)-*α*-D-mannopyranosyl]-D-*myo*-inositol (34). A mixture of the pseudotrisaccharide **2** (129 mg, 0.095 mmol) and 3 Å molecular sieves in Et_2O (10 mL) was stirred at room temperature for 1 h under nitrogen. The reaction flask was cooled to –40 °C, and TMSOTf (3 μL, 0.02 mmol) was added. A solution of the trichloroacetimidate **33** (225 mg, 0.11 mmol) in Et_2O (2 mL) was then added through the syringe pump for over a period of 30 min at the same temperature. After 2 h, Et_3N (10 μL) was added to quench the reaction and the whole mixture was filtered through Celite. The filtrate was concentrated under reduced pressure to furnish a crude residue, which was purified by flash column chromatography (ethyl acetate/hexanes = 1/2.5) to provide the pseudoheptasaccharide **34** (158 mg, 52%), together with the recovered acceptor **2** (53 mg, 41%). $[\alpha]^{27}_{D}$ +30.8 (*c* 2.2, $CHCl_3$); ^1H NMR (600 MHz, $CDCl_3$): δ 7.78–7.65 (m, 4H, Ar-H), 7.45–7.43 (m, 3H, Ar-H), 7.37–7.00 (m, 105H, Ar-H), 5.40 (s, 1H, Man 1-H), 5.19 (s, 1H, Man 1-H), 5.16 (s, 1H, Man 1-H), 4.95 (s, 1H, Man 1-H), 4.92 (s, 1H, Man 1-H), 4.89 (s, 1H, Man 1-H), 4.84–4.33 (m, 44H, ArC*H*$_2$), 4.15 (t, *J* = 2.0 Hz, 1H), 4.11–4.04 (m, 4H), 3.98 (t, *J* = 7.2 Hz, 1H), 3.93–3.75 (m, 16H), 3.72–3.70 (m, 1H), 3.68–3.43 (m, 13H), 3.41–3.37 (m, 3H), 3.32–3.27 (m, 2H), 3.22 (dd, *J* = 9.8, 2.3 Hz, 1H), 3.10 (s, 1H); ^{13}C NMR (150 MHz, $CDCl_3$): δ 138.76 (C), 138.66 (C), 138.63 (C), 138.59 (C), 138.5 (C), 138.4 (C), 138.3 (C), 138.10 (C), 138.05 (C), 138.0 (C), 137.9 (C), 137.6 (C), 136.4 (C), 135.8 (C), 134.5 (C), 133.2 (C), 132.9 (C), 129.7 (CH), 129.0 (CH), 128.5 (CH), 128.4 (CH), 128.3 (CH), 128.22 (CH), 128.19 (CH), 128.10 (CH), 128.08 (CH), 128.0 (CH), 127.93 (CH), 127.87 (CH), 127.79 (CH), 127.77 (CH), 127.71 (CH), 127.67 (CH), 127.64 (CH), 127.57 (CH), 127.53 (CH), 127.51 (CH), 127.44 (CH), 127.40 (CH), 127.38 (CH), 127.3 (CH), 127.24 (CH), 127.18 (CH), 100.5 (CH), 99.2 (CH × 3), 98.6 (CH), 98.5 (CH), 80.3 (CH), 80.2 (CH), 79.83 (CH), 79.79 (CH), 79.1 (CH), 78.8 (CH), 78.12 (CH), 75.08 (CH_2), 74.99 (CH_2), 74.95 (CH_2), 74.9 (CH_2), 74.8 (CH), 74.62 (CH), 74.59 (CH), 74.5 (CH), 74.3 (CH), 74.0 (CH), 73.9 (CH), 73.8 (CH), 73.5 (CH_2), 73.3 (CH_2), 73.2 (CH_2), 73.1 (CH_2, CH), 72.8 (CH_2), 72.6 (CH_2), 72.5 (CH_2), 72.2 (CH_2, CH), 72.1 (CH_2), 71.90 (CH_2), 71.86 (CH), 71.8 (CH_2), 71.7 (CH), 71.5 (CH_2), 71.2 (CH_2), 69.2 (CH_2), 68.9 (CH_2), 68.8 (CH_2), 68.7 (CH_2), 66.8 (CH_2), 66.2 (CH_2); HRMS (ESI) m/z calcd for $C_{200}H_{204}O_{36}Na_2$ ($[M + 2Na]^{2+}$): 1614.7042, found: 1614.7046.

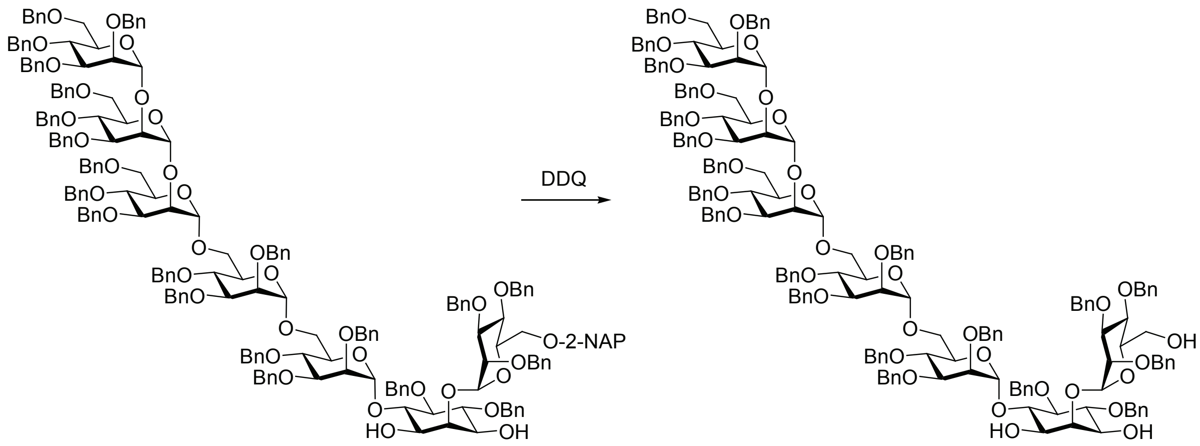

4,5-Di-*O*-benzyl-2-*O*-(2,3,4-tri-*O*-benzyl-*α*-D-mannopyranosyl)-6-*O*-[(2,3,4,6-tetra-*O*-benzyl-*α*-D-mannopyranosyl)-(1→2)-(3,4,6-tri-*O*-benzyl-*α*-D-mannopyranosyl)-(1→2)-(3,4,6-tri-*O*-benzyl-*α*-D-mannopyranosyl)-(1→6)-(2,3,4-tri-*O*-benzyl-*α*-D-mannopyranosyl)-(1→6)-2,3,4-tri-*O*-benzyl-*α*-D-mannopyranosyl]-D-*myo*-inositol (S17). To the solution of compound **34** (75 mg, 24.0 μmol) in a CH_2Cl_2/H_2O (19/1, 4 mL) mixed solvent, 2,3-dichloro-5,6-dicyano-1,4-benzoquinone (DDQ, 16 mg, 71 μmol) was added in three equal portions in half hour intervals at room temperature. After stirring for a total of 2 h, the reaction was filtered through Celite, the filtrate was diluted with CH_2Cl_2 (5 mL) and washed successively with saturated $NaHCO_{3(aq)}$ and brine. After drying over $MgSO_4$, the organic layer was concentrated under reduced pressure, and the residue was purified by flash column chromatography (ethyl acetate/hexanes = 1/1) to afford the triol **S17** (51 mg, 71%). $[\alpha]^{26}_{D}$ +24.7 (*c* 2.0, $CHCl_3$); ^1H NMR (600 MHz, $CDCl_3$): δ 7.34–7.00 (m, 105H, Ar-H), 5.33 (s, 1H, Man 1-H), 3.20 (s, 1H, Man 1-H), 5.17 (s, 1H, Man 1-H), 4.98 (s, 1H, Man 1-H), 4.90–4.73 (m, 10H, Man 1-H × 2, PhCH*H* × 8), 4.69–4.27 (m, 34H, PhC*H*$_2$), 4.20 (t, *J* = 2.0 Hz, 1H, Ins 2-H), 4.16 (t, *J* = 2.0 Hz, 1H, Man 2-H), 4.11 (t, *J* = 2.0 Hz, 1H, Man 2-H), 4.08–4.01 (m, 2H), 3.95–3.75 (m, 19H), 3.70–3.61 (m, 6H), 3.60–3.57 (m, 3H), 3.53–3.38 (m, 7H), 3.25–3.20 (m, 2H, Ins-H × 2); ^{13}C NMR (150 MHz, $CDCl_3$): δ 138.8 (C), 138.7 (C), 138.6 (C), 138.5 (C), 138.4 (C), 138.3 (C), 138.1 (C), 138.0 (C), 137.9 (C), 137.7 (C), 137.6 (C), 128.5 (CH), 128.4 (CH), 128.3 (CH), 128.20 (CH), 128.16 (CH), 128.14 (CH), 128.10 (CH), 128.07 (CH), 128.0 (CH), 127.87 (CH), 127.79 (CH), 127.77 (CH), 127.74 (CH), 127.70 (CH), 127.6 (CH), 127.51 (CH), 127.47 (CH), 127.44 (CH), 127.42 (CH), 127.40 (CH), 127.29 (CH), 127.27 (CH), 100.5 (CH), 99.3 (CH × 2), 98.7 (CH), 98.6 (CH × 2), 80.4 (CH), 79.84 (CH), 79.79 (CH), 78.82 (CH), 78.76 (CH), 78.2 (CH), 75.2 (CH_2), 75.0 (CH_2), 74.91 (CH_2), 74.88 (CH_2), 74.8 (CH), 74.7 (CH_2, CH), 74.6 (CH), 74.53 (CH), 74.50 (CH), 74.3

(CH), 74.2 (CH), 74.0 (CH), 73.3 (CH_2), 73.2 (CH_2), 73.1 (CH_2), 72.9 (CH_2), 72.8 (CH_2), 72.6 (CH_2), 72.52 (CH_2), 72.45 (CH_2), 72.3 (CH), 72.10 (CH_2, CH), 72.07 (CH_2, CH), 72.0 (CH_2), 71.9 (CH_2, CH), 71.8 (CH) 71.7 (CH_2), 71.2 (CH_2, CH), 69.3 (CH_2), 68.9 (CH_2), 68.8 (CH_2), 67.1 (CH_2), 66.2 (CH_2), 61.9 (CH_2); HRMS (MALDI): *m/z* calcd for $C_{189}H_{198}O_{36}Na$ ($[M + Na]^+$): 3068.6306, found: 3068.6387.

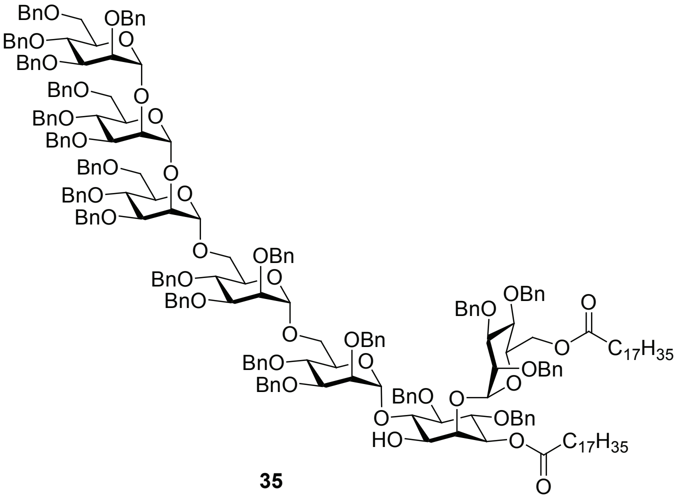

35

4,5-Di-*O*-benzyl-6-*O*-[(2,3,4,6-tetra-*O*-benzyl-*α*-D-mannopyranosyl)-(1→2)-(3,4,6-tri-*O*-benzyl-*α*-D-mannopyranosyl)-(1→2)-(3,4,6-tri-*O*-benzyl-*α*-D-mannopyranosyl)-(1→6)-(2,3,4-tri-*O*-benzyl-*α*-D-mannopyranosyl)-(1→6)-2,3,4-tri-*O*-benzyl-*α*-D-mannopyranosyl]-2-*O*-(2,3,4-tri-*O*-benzyl-6-*O*-stearoyl-*α*-D-mannopyranosyl)-3-*O*-stearoyl-D-*myo*-inositol (35). To the solution of compound **S17** (25 mg, 8.2 μmol) and stearic acid (12 mg, 42.2 μmol) in CH_2Cl_2 (2 mL), DCC (9 mg, 43.6 μmol) and DMAP (5 mg, 42.2 mmol) were added at 0 °C under nitrogen atmosphere. The reaction was then warmed up to room temperature and stirring was continued for 2 d. The whole mixture was filtered through Celite, washed successively with saturated $NaHCO_{3(aq)}$ and brine. The organic layer was dried over $MgSO_4$, concentrated under reduced pressure, and the residue was purified by flash column chromatography to furnish diester **35** (25 mg, 86%). $[\alpha]^{21}_D$ +10.3 (*c* 0.7, $CHCl_3$); IR ($CHCl_3$): *ν* 2849, 1735, 1159, 1458, 1494, 1383, 1260, 1099, 3026, 802, 738, 695 cm^{-1}; ^1H NMR (600 MHz, $CDCl_3$): *δ* 7.36–7.02 (m, 105H), 5.18 (s, 1H, Man 1-H), 5.15 (s, Man 1-H), 5.0 (s, 1H, Man 1-H), 4.91 (m, Man 1-H × 3), 4.88–4.24 (m, 43H, PhCH*H* × 42, Ins 3-H), 4.21 (t, *J* = 1.9 Hz 1H, Ins 2-H), 4.15–4.13 (m, 2H), 4.08–4.01 (m, 5H), 3.95–3.76 (m, 19H), 3.65–3.56 (m, 9H), 3.52–3.45 (m, 3H), 3.38–3.35 (m, 2H), 2.20 (t, *J* = 7.6 Hz, 2H), 1.96–1.86 (m, 2H), 1.46–1.13 (m, 60H), 0.87 (t, J = 7.0 Hz, 6H); ^{13}C NMR (150 MHz, $CDCl_3$): *δ* 173.5 (C=O), 172.0 (C=O), 138.8 (C), 138.72 (C), 138.64 (C), 138.6

(C), 138.53 (C), 138.47 (C), 138.40 (C), 138.35 (C), 138.3 (C), 138.2 (C), 137.9 (C), 137.4 (C), 128.6 (CH), 128.40 (CH), 128.36 (CH), 128.29 (CH), 128.25 (CH), 128.22 (CH), 128.18 (CH), 128.1 (CH), 128.0 (CH), 127.9 (CH), 127.80 (CH), 127.77 (CH), 127.73 (CH), 127.70 (CH), 127.65 (CH), 127.61 (CH), 127.57 (CH), 127.53 (CH), 127.45 (CH), 127.40 (CH), 127.35 (CH), 127.31 (CH), 127.26 (CH), 127.2 (CH), 100.5 (CH), 100.4 (CH), 99.3 (CH), 99.2 (CH), 98.8 (CH), 98.6 (CH), 80.1 (CH), 79.9 (CH), 79.4 (CH), 78.9 (CH), 77.7 (CH), 76.1 (CH), 75.5 (CH), 75.2 (CH_2), 75.03 (CH_2, CH), 74.95 (CH_2), 74.9 (CH_2), 74.7 (CH_2), 74.6 (CH), 74.5 (CH_2), 74.4 (CH), 74.3 (CH), 74.2 (CH), 74.1 (CH), 74.0 (CH), 73.31 (CH_2), 73.2 (CH_2), 73.1 (CH_2), 73.0 (CH), 72.84 (CH_2), 72.79 (CH_2), 72.52 (CH_2), 72.46 (CH_2), 72.33 (CH_2), 72.28 (CH_2), 72.1 (CH_2), 71.93 (CH_2), 71.87 (CH), 71.5 (CH_2), 71.3 (CH_2), 70.5 (CH), 69.3 (CH_2), 68.9 (CH_2), 68.8 (CH_2), 66.7 (CH_2), 66.2 (CH_2), 62.7 (CH_2), 36.6 (CH_2), 34.1 (CH_2), 34.0 (CH_2), 29.7 (CH_2), 29.6 (CH_2), 29.5 (CH_2), 29.4 (CH_2), 29.24 (CH_2), 29.18 (CH_2), 24.8 (CH_2), 24.7 (CH_2), 22.7 (CH_2), 14.1 (CH_3); HRMS (MALDI): *m*/*z* calcd for $C_{225}H_{266}O_{38}Na$ ($[M + Na]^+$): 3601.5688, found: 3601.5737.

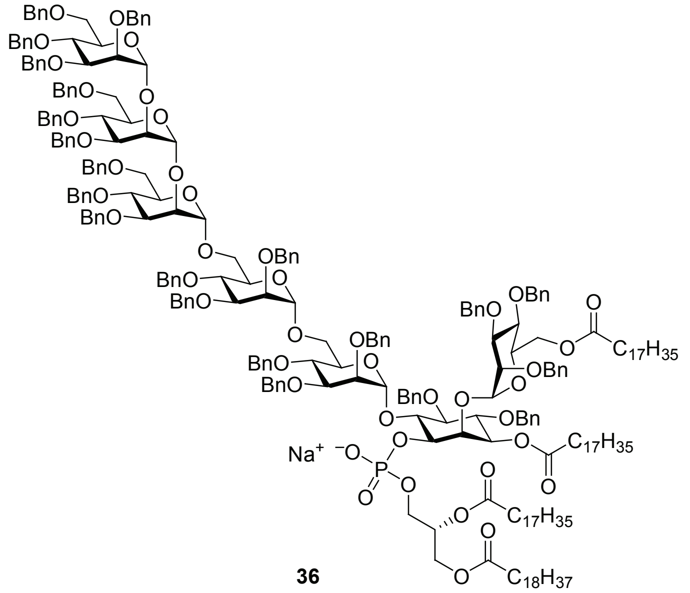

36

4,5-Di-*O*-benzyl-6-*O*-[(2,3,4,6-tetra-*O*-benzyl-*α*-D-mannopyranosyl)-(1→2)-(3,4,6-tri-*O*-benzyl-*α*-D-mannopyranosyl)-(1→2)-(3,4,6-tri-*O*-benzyl-*α*-D-mannopyranosyl)-(1→6)-(2,3,4-tri-*O*-benzyl-*α*-D-mannopyranosyl)-(1→6)-2,3,4-tri-*O*-benzyl-*α*-D-mannopyranosyl]-2-*O*-(2,3,4-tri-*O*-benzyl-6-*O*-stearoyl-*α*-D-mannopyranosyl)-1-*O*-{1-*O*-[(*R*)-10-methyloctadecanoyl]-2-*O*-stearoyl-*sn*-glycerylphosphonato}-3-*O*-stearoyl-D-*myo*-inositol sodium salt (36). The moisture in compound **35** (11 mg, 3.1 μmol) and the *H*-phosphonate **4** (25 mg, 31.1 μmol) were coevaporated with pyridine and the resulting mixture

was further dried under vacuum for 1 h before dissolving in dry pyridine. Pivaloyl chloride (8 μL, 58 μmol) was then added. After 5 h of stirring at room temperature, a freshly prepared solution of iodine (8 mg, 32 μmol) in pyridine/water (50/1, 1 mL) was added. After 3 h, the reaction was diluted with $CHCl_3$, washed with saturated $Na_2SO_{3(aq)}$ and triethylammonium bicarbonate buffer solution, and then dried over $MgSO_4$. The crude product obtained after the removal of the solvent *in vacuo* was purified by flash column chromatography using a Et_3N-containing silica gel (MeOH/$CHCl_3$ 1/20) to give a triethylammonium salt, which was subjected to Na^+ cation-exchange using Dowex 50WxNa^+ IR-resin in $CHCl_3$/MeOH (1/1, 1 mL) for 3 h to provide compound **36** (10 mg, 77%) as a yellowish oil. $[\alpha]^{24}_{D}$ +24.2 (*c* 4.0, $CHCl_3$); ^{1}H NMR (600 MHz, $CDCl_3$): δ 7.41–7.06 (m, 105H, Ar-H), 5.41 (bs, 1H, gly-H), 5.24 (s, 2H, Man 1-H × 2), 5.07 (s, 1H, Man 1-H), 4.95 (s, 1H, Man 1-H), 4.92 (s, 1H, Man 1-H), 4.91 (s, 1H, Man 1-H), 4.90–4.29 (m, 43H), 4.20–3.66 (m, 34H), 3.63–3.28 (m, 7H), 2.24–2.11 (m, 8H), 1.56–1.47 (m, 8H),1.29–1.05 (m, 111H), 0.91 (t, *J* = 7.0 Hz, 12H), 0.86 (d, *J* = 6.5 Hz, 3H); ^{13}C NMR (150 MHz, $CDCl_3$): δ 173.5 (C=O), 173.2 (C=O), 173.0 (C=O), 172.4 (C=O), 139.0 (C), 138.8 (C), 138.73 (C), 138.71 (C). 138.65 (C), 138.6 (C), 138.51 (C), 138.46 (C), 138.42 (C), 138.37 (C), 138.3 (C), 138.2 (C), 137.8 (C) 128.6 (CH), 128.5 (CH), 128.32 (CH), 128.28 (CH), 128.24 (CH), 128.19 (CH), 128.15 (CH), 128.14 (CH), 128.07 (CH), 128.0 (CH), 127.90 (CH), 127.87 (CH), 127.82 (CH), 127.78 (CH), 127.73 (CH), 127.69 (CH), 127.63 (CH), 127.59 (CH), 127.7 (CH), 127.52 (CH), 127.45 (CH), 127.43 (CH), 127.40 (CH), 127.37 (CH), 127.35 (CH), 127.31 (CH), 127.29 (CH), 127.26 (CH), 127.2 (CH), 127.0 (CH), 126.9 (CH), 100.3 (CH), 99.24 (2 × CH), 99.20 (2 × CH), 98.6 (CH), 80.1 (CH), 79.9 (CH), 79.8 (CH), 79.5 (CH), 78.8 (CH), 77.8 (CH), 75.7 (CH), 75.04 (CH_2), 75.00 (CH_2), 74.95 (CH_2), 74.87 (CH, CH_2), 74.8 (CH_2), 74.7 (CH), 74.6 (CH), 74.5 (CH), 74.42 (CH), 74.36 (CH_2), 74.2 (CH), 74.0 (CH), 73.8 (CH), 73.4 (CH_2), 73.3 (CH_2), 73.2 (CH_2), 72.99 (CH_2), 72.96 (CH_2), 72.8 (CH), 72.5 (CH_2), 72.4 (CH_2, CH), 72.3 (CH_2, CH) 72.1 (CH_2), 71.94 (CH_2, CH), 71.89 (CH), 71.7 (CH_2), 71.6 (CH), 71.2 (CH_2), 71.14 (CH_2), 71.1 (CH), 70.5 (CH), 69.2 (CH_2), 68.8 (CH_2), 66.0 (CH_2), 62.7 (CH_2), 61.9 (CH_2), 37.1 (CH_2), 34.2 (CH_2), 34.1 (CH_2), 34.0 (CH_2), 32.3 (CH), 31.9 (CH_2), 30.0 (CH_2), 29.7 (CH_2), 29.61 (CH_2), 29.55 (CH_2), 29.5 (CH_2), 29.4 (CH_2), 29.3 (CH_2), 29.24 (CH_2), 29.18 (CH_2), 29.1 (CH_2), 27.1 (CH_2), 24.9 (CH_2), 24.81 (CH_2), 24.79 (CH_2), 24.5 (CH_2), 22.7 (CH_2), 19.7 (CH_3), 14.1 (3 × CH_3); ^{31}P NMR (121.5 MHz, $CDCl_3$): δ 1.85; HRMS (EI): *m*/*z* calcd for $C_{265}H_{342}Na_2O_{45}P$ ($[M+H]^+$): 4324.5752, found: 4324.5933.

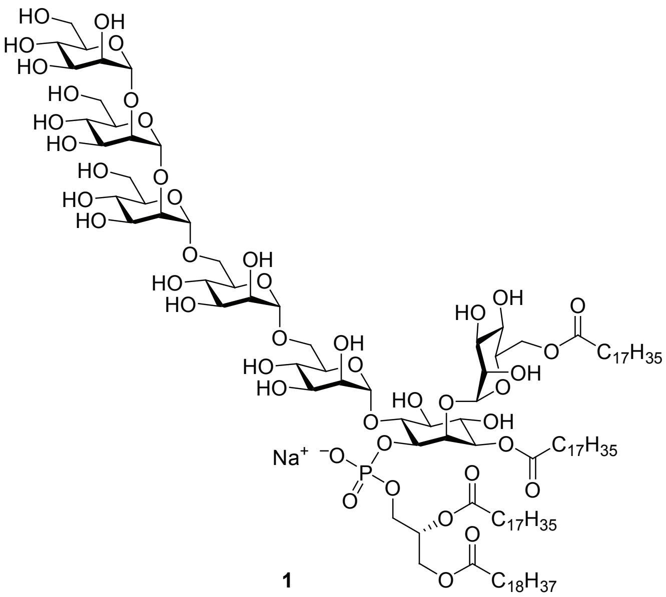

1

6-*O*-[(*α*-D-mannopyranosyl)-(1→2)-(*α*-D-mannopyranosyl)-(1→2)-(*α*-D-mannopyranosyl)-(1→6)-(*α*-D-mannopyranosyl)-(1→6)-*α*-D-mannopyranosyl]-1-*O*-{1-*O*-[(*R*)-10-methyloctadecanoyl]-2-*O*-stearoyl-*sn*-glycerylphosphonato}-3-*O*-stearoyl-2-*O*-(6-*O*-stearoyl-*α*-D-mannopyranosyl)-D-*myo*-inositol sodium salt (1). A solution of compound **36** in EtOAc/THF/*n*-propyl alcohol/H_2O (2 mL, 2:1:1:1) mixed solvent with suspended Pd/C (100 mg, 10% Pd content) was stirred under an atmosphere of hydrogen for 20 h. The reaction mixture was filtered through a short Celite plug with 1-PrOH/H_2O (1:1) as eluent. The filtrate was concentrated and lyophilized to give compound **1** (4.9 mg, 88%) as a white solid. ^{1}H NMR (600 MHz, $CDCl_3/CD_3OD/D_2O$ = 60/35/8): δ 5.30 (bs, 1H, glycerol-H), 5.27 (s, Man 1-H), 5.10 (s, 1H, Man 1-H), 5.09 (s, Man 1-H), 5.07 (s, Man 1-H), 5.00 (s, Man 1-H), 4.99 (s, Man 1-H), 4.92 (d, J = 10.9 Hz, 1H, Ins 3-H), 4.40–3.53 (m, 53H), 2.39–2.31 (m, 8H), 1.63–1.58 (m, 8H), 1.33–1.26 (m, 111H), 0.89 (t, J = 7.0 Hz, 12 H), 0.86 (t, J = 6.4 Hz, 3H); ^{13}C NMR (150 MHz, $CDCl_3/CD_3OD/D_2O$ = 60/35/8): δ 174.6 (C=O), 173.9 (C=O), 173.7 (C=O), 172.5 (C=O), 101.8 (CH), 100.3 (CH), 99.3 (CH), 99.0 (CH), 98.7 (CH), 97.7 (CH), 78.5 (CH), 78.1 (CH), 78.0 (CH), 77.9 (CH), 74.6 (CH), 73.6 (CH), 72.94 (CH), 72.88 (CH), 72.6 (CH), 72.5 (CH), 70.8 (CH), 70.7 (CH), 70.4 (CH), 70.3 (CH × 2), 70.2 (CH), 70.20 (CH), 70.19 (CH), 70.14 (CH × 2), 70.05 (CH), 70.0 (CH), 69.9 (CH × 2), 69.8 (CH), 67.0 (CH), 66.9 (CH), 66.8 (CH × 2), 66.6 (CH), 66.4 (CH), 65.5 (CH_2), 65.3 (CH_2), 63.7 (CH_2), 63.3 (CH_2), 62.5 (CH_2), 61.0 (CH_2 × 2), 60.9 (CH_2), 36.6 (CH_2), 33.9 (CH_2), 33.6 (CH_2), 33.5 (CH_2), 33.3 (CH), 31.4 (CH_2), 29.6 (CH_2), 29.5 (CH_2), 29.2 (CH_2), 29.0 (CH_2), 28.9 (CH_2), 28.72 (CH_2), 28.66 (CH_2), 28.55 (CH_2), 26.63 (CH_2), 26.58 (CH_2), 24.5 (CH_2), 24.4 (CH_2), 24.3 (CH_2), 24.0 (CH_2), 22.2 (CH_2), 19.1 (CH_3), 13.4 (CH_3 × 3); ^{31}P

NMR (121.5 MHz, $CDCl_3/CD_3OD/ D_2O$ = 60/35/8): δ –0.45; HRMS (ESI): *m*/*z* calcd for $C_{118}H_{216}Na_2O_{45}P$ ($[M + Na]^+$): 2431.9480, found: 2431.9573.

Supplementary References

1. Woods, R. J., Andrews, C. W. & Bowen, J. P. Molecular mechanical investigations of the properties of oxocarbenium ions. 2. Application to glycoside hydrolysis. *J. Am. Chem. Soc.* **114**, 859–864 (1992).

2. Patil, P. S. & Hung, S.-C. Total synthesis of phosphatidylinositol dimannoside: a cell-envelope component of Mycobacterium tuberculosis. *Chem. Eur. J.* **15**, 1091–1094 (2009).

3. Watt, J. A. & Williams, S. J. Rapid, iterative assembly of octyl α-1,6-oligomannosides and their 6-deoxy equivalents. *Org. Biomol. Chem.* **3**, 1982 (2005).

4. Patil, P. S., Lee, C.-C., Huang, Y.-W., Zulueta, M. M. L. & Hung, S.-C. Regioselective and stereoselective benzylidene installation and one-pot protection of D-mannose. *Org. Biomol. Chem.* **11**, 2605–2612 (2013).

5. Li, H., Wu, J., Luo, J., & Dai, W.-M. A concise total synthesis of amphidinolide T2. *Chem. Eur. J.* **16**, 11530–11534 (2010).

6. Liu, X., Stocker, B. L. & Seeberger, P. H. Total synthesis of phosphatidylinositol mannosides of *Mycobacterium tuberculosis*. *J. Am. Chem. Soc.* **128**, 3638–3648 (2006).
